# Safety and tolerability of HIV-1 multiantigen pDNA vaccine given with IL-12 plasmid DNA via electroporation, boosted with a recombinant vesicular stomatitis virus HIV Gag vaccine in healthy volunteers in a randomized, controlled clinical trial

**DOI:** 10.1371/journal.pone.0202753

**Published:** 2018-09-20

**Authors:** Marnie L. Elizaga, Shuying S. Li, Nidhi K. Kochar, Gregory J. Wilson, Mary A. Allen, Hong Van N. Tieu, Ian Frank, Magdalena E. Sobieszczyk, Kristen W. Cohen, Brittany Sanchez, Theresa E. Latham, David K. Clarke, Michael A. Egan, John H. Eldridge, Drew Hannaman, Rong Xu, Ayuko Ota-Setlik, M. Juliana McElrath, Christine Mhorag Hay, on behalf of the NIAID HIV Vaccine Trials Network (HVTN) 087 Study Team

**Affiliations:** 1 Vaccine and Infectious Disease Division, Fred Hutchinson Cancer Research Center, Seattle, Washington, United States of America; 2 Division of Pediatric Infectious Diseases, Vanderbilt University Medical Center, Nashville, Tennessee, United States of America; 3 National Institute of Allergy and Infectious Diseases, National Institutes of Health, Bethesda, Maryland, United States of America; 4 Laboratory of Infectious Disease Prevention, New York Blood Center, New York, New York, United States of America; 5 Department of Medicine, University of Pennsylvania Perelman School of Medicine, Philadelphia, Pennsylvania, United States of America; 6 Division of Infectious Diseases, Columbia University Medical Center, New York, New York, United States of America; 7 Profectus BioSciences, Incorporated, Pearl River, New York, United States of America; 8 Ichor Medical Systems, Incorporated, San Diego, California, United States of America; 9 Department of Laboratory Medicine, University of Washington, Seattle, Washington, United States of America; 10 Department of Global Health, University of Washington, Seattle, Washington, United States of America; 11 Department of Medicine, University of Washington, Seattle, Washington, United States of America; 12 Infectious Diseases Division, University of Rochester Medical Center, Rochester, New York, United States of America; George Washington University School of Medicine and Health Sciences, UNITED STATES

## Abstract

**Background:**

The addition of plasmid cytokine adjuvants, electroporation, and live attenuated viral vectors may further optimize immune responses to DNA vaccines in heterologous prime-boost combinations. The objective of this study was to test the safety and tolerability of a novel prime-boost vaccine regimen incorporating these strategies with different doses of *IL-12* plasmid DNA adjuvant.

**Methods:**

In a phase 1 study, 88 participants received an HIV-1 multiantigen (gag/pol, env, nef/tat/vif) DNA vaccine (HIV-MAG, 3000 μg) co-administered with *IL-12* plasmid DNA adjuvant at 0, 250, 1000, or 1500 μg (N = 22/group) given intramuscularly with electroporation (Ichor TriGrid™ Delivery System device) at 0, 1 and 3 months; followed by attenuated recombinant vesicular stomatitis virus, serotype Indiana, expressing HIV-1 Gag (VSV-Gag), 3.4 ⊆ 10^7^ plaque-forming units (PFU), at 6 months; 12 others received placebo. Injections were in both deltoids at each timepoint. Participants were monitored for safety and tolerability for 15 months.

**Results:**

The dose of *IL-12* pDNA did not increase pain scores, reactogenicity, or adverse events with the co-administered DNA vaccine, or following the VSV-Gag boost. Injection site pain and reactogenicity were common with intramuscular injections with electroporation, but acceptable to most participants. VSV-Gag vaccine often caused systemic reactogenicity symptoms, including a viral syndrome (in 41%) of fever, chills, malaise/fatigue, myalgia, and headache; and decreased lymphocyte counts 1 day after vaccination.

**Conclusions:**

HIV-MAG DNA vaccine given by intramuscular injection with electroporation was safe at all doses of IL-12 pDNA. The VSV-Gag vaccine at this dose was associated with fever and viral symptoms in some participants, but the vaccine regimens were safe and generally well-tolerated.

**Trial registration:**

Clinical Trials.gov NCT01578889.

## Introduction

Several approaches to enhance the immunogenicity of DNA vaccination have been investigated since DNA vaccination was first performed in humans. These have included utilizing DNA vaccination as a prime for a heterologous viral vector boost [1, 2], use of plasmid cytokine adjuvants [3] and delivering plasmids with *in vivo* electroporation (EP) [4]. Here we report on the safety and efficacy of combining all 3 approaches to elicit immune responses against HIV.

Prime-boost strategies are routinely used in vaccination regimens to increase the magnitude and duration of the immune response. Heterologous prime-boost regimens are especially promising, and are being studied for a number of infections including HIV, malaria, and tuberculosis. Vesicular stomatitis virus (VSV) has been explored as a potentially useful vector for vaccines because of its well-characterized genome of transcriptional units which express viral proteins, and which can accommodate additional transcriptional units to express foreign proteins [5, 6]. Importantly, in most regions of the world, humans do not commonly encounter VSV, and immunization with an rVSV vaccine is unlikely to either activate and expand VSV-specific CD4+ T-cells, possibly leading to increased risk of HIV infection, or to encounter pre-existing anti-vector immunity that would result in rapid clearance of the vector and reduced immune responses to the target antigens [7]. The clinical safety of a highly attenuated Indiana serotype rVSV vector (rVSV_IN_) was demonstrated in HVTN 090, a phase 1 trial in which doses of 4.6 x 10^3^ to 3.4⊆10^7^ PFU VSV-Gag (Profectus Biosciences, Pearl River, NY) given at 2 timepoints, 2 months apart, were found to be safe and well tolerated [8]. That study also showed that while neutralizing antibody was elicited against the VSV-Gag vector, the second vaccination still induced more frequent HIV Gag-specific immune responses. A less attenuated rVSV vector vaccine, rVSV-ZEBOV (Merck, Kenilworth, NJ), has also been tested in several recent human studies as an Ebola vaccine candidate. While more adverse events were noted compared with those observed with rVSV-Gag in HVTN 090, the safety profile of rVSV-ZEBOV was considered acceptable for a vaccine that would provide protection against Ebola disease, and efficacy was demonstrated in a recent ring-vaccination trial [9–12].

EP is the application of a localized electrical field at the site of injection to facilitate cell uptake of DNA by permeabilizing cell membranes and possibly by increasing local inflammation [13–15]. EP has been shown to be an efficient way to introduce DNA into cells [16] and has been used for more than 3 decades by molecular biologists for cell transfection [17–20]. More recently, clinical applications of EP have been tested in cancer treatment and gene therapy [16, 21–23]. Adding EP to the IM injection procedure has resulted in improved immunogenicity of some DNA vaccines [24–27]. In a recent phase 1 study, HIV Vaccine Trials Network (HVTN) protocol 080, EP using the Cellectra EP device (Inovio Pharmaceuticals, Blue Bell, PA) was used for the DNA vaccine PENNVAX-B (PV-B, 3000 μg), given IM with or without co-administration of IL-12 pDNA (1500 μg). EP demonstrated a significant vaccine dose-sparing effect, and increased the frequency of CD4+ and CD8+ T-cell immune responses compared to IM injection without EP in a previous study, HVTN 070 (PV-B 6000 μg ± IL-12 pDNA 1000 μg) [26]. EP remains investigational and is not yet licensed by the U.S. Food and Drug Administration for clinical use.

Interleukin 12 (IL-12) is a pleiotropic cytokine mainly produced by monocytes, macrophages, and dendritic cells. IL-12 is essential for the differentiation, proliferation, and maintenance of T helper 1 (Th1) type responses that lead to IFN-gamma and IL-2 production. Although *IL-12* pDNA has been shown to enhance immune responses in preclinical vaccine studies [28–30], early studies in humans of DNA vaccines coadministered with *IL-12* pDNA failed to augment immune responses [31]. However, in HVTN 080, in which *IL-12* pDNA was administered with EP, an adjuvant effect of IL-12 was suggested, though not statistically significant [26].

In preclinical studies, the adjuvant activity of *IL-12* pDNA was highly dose dependent, with the highest doses of *IL-12* actually decreasing immunogenicity of the co-administered DNA vaccine when compared to lower doses suggesting the possibility of a J-curve effect of *IL-12* dose level [32, 33]. The optimal dose range of *IL-12* pDNA for adjuvant activity in humans has not yet been identified.

In this phase Ia clinical trial, HVTN 087, we evaluated the safety and immunogenicity of an HIV-1 multi-antigen plasmid DNA (HIV-MAG) vaccine delivered by intramuscular EP, with and without *IL-12* pDNA, and followed by an rVSV HIV-1 Gag (VSV-Gag) boost. This study was designed to determine the optimal dose of *IL-12* pDNA as a cytokine molecular adjuvant to use with the DNA vaccine delivered by EP. In addition, the study evaluated the effects of the different priming regimens on immune responses to a prototype VSV-Gag vaccine boost. The safety and tolerability of the prime-boost vaccine regimen is reported here. Immunogenicity results have been reported in a separate publication [34].

## Materials and methods

### Study design

HVTN 087 (NCT01578889) was a multicenter, randomized, placebo-controlled, double-blind phase Ia trial conducted by the NIAID-funded HVTN at 4 clinical trial sites in the United States: Nashville, TN; New York, NY; Rochester, NY; Philadelphia, PA. The primary objective was to investigate the safety and tolerability of HIV-MAG delivered by intramuscular EP with and without *IL-12* pDNA adjuvant and boosted with VSV-Gag; immunogenicity and *IL-12* dose response were secondary objectives. The study was approved and reviewed regularly by the Fred Hutchinson Cancer Research Center Institutional Review Board (for the New York and Philadelphia sites), the University of Rochester Research Subjects Review Board, and the Vanderbilt University Institutional Review Board. The study opened on May 17, 2012, and concluded all follow-up on September 9, 2016.

After written informed consent, participants were screened for eligibility and willingness to participate. Participants were eligible if between 18 and 50 years of age; in good general health based on history, physical examination and clinical laboratory investigations; considered at low risk for HIV acquisition based on behavioral questionnaires and discussion; and had no history of receiving investigational products, immunosuppressive medication, blood products, immunoglobulin or vaccines within study-defined periods prior to enrollment. Female participants of childbearing potential were not pregnant or planning to become pregnant and agreed to consistently use contraception for 21 days prior to their first vaccination until 3 months after the last vaccination. All participants were counseled at each visit about HIV risk reduction.

Participants were randomized to one of 4 treatment arms or placebo. The study began with Groups 1, 2, and 3. Participants could state a preference to attend more visits (Groups 1 and 3), or fewer visits (Group 2 and later, 4). Groups 1 and 3 were randomized together, in blocks of 12, 12, and 26 each split evenly between the groups, with 2 placebo assignments in each block. Group 2 was randomized as a block of 25 with 2 assigned to placebo. Following a safety review of data through 2 weeks after first vaccination from 12 participants in each of Groups 1 and 3, and 25 participants in Group 2, Group 4 was opened to enrollment, randomized as a block of 25 with 2 assigned to placebo. The randomization allocation sequence was obtained by computer-generated random numbers and provided to each clinical site pharmacist through a web-based randomization system. This pharmacist was charged with maintaining security of the treatment assignments.

Participants either received 3,000 μg HIV-MAG (*gag/pol*, *env*, *nef/tat/vif*) DNA vaccine co-administered with *IL-12* pDNA at 0, 250, 1000, or 1500 μg (N = 22/group) given intramuscularly by EP at 0, 1 and 3 months and boosted by VSV-Gag vaccine at 6 months; or they received placebo given IM by EP at 0, 1, 3 months followed by placebo given IM at 6 months (N = 12) (Table 1). Participants and site staff (except for site pharmacists) were blinded as to participant assignment to active vaccine or placebo, but not to group assignment. The vaccines and placebos were all clear, colorless solutions, and were prepared with identical labels prior to administration.

Participants were assessed for reactogenicity, tolerability, adverse events, and social impacts of participation.

**Table 1:** HVTN 087 study schema.

Study arm	N V/P	*pIL-12* Dose	Month 0	Month 1	Month 3	Month 6
Group 1	22	0 μg	DNA	DNA	DNA	VSV_IN_
	3	0 μg	placebo	placebo	placebo	placebo
Group 2	22	250 μg	DNA + *IL-12*	DNA + *IL-12*	DNA + *IL-12*	VSV_IN_
	3	0 μg	placebo	placebo	placebo	placebo
Group 3	22	1000 μg	DNA + *IL-12*	DNA + *IL-12*	DNA + *IL-12*	VSV_IN_
	3	0 μg	placebo	placebo	placebo	placebo
Group 4	22	1500 μg	DNA + *IL-12*	DNA + *IL-12*	DNA + *IL-12*	VSV_IN_
	3	0 μg	placebo	placebo	placebo	placebo

### Study agents

The HIV-MAG DNA, IL-12 DNA and VSV-Gag vaccine study components were provided by Profectus Biosciences, Pearl River, NY.

#### HIV-1 multiantigen pDNA (HIV-MAG) vaccine

The HIV-MAG vaccine consists of two plasmid DNA expression vectors, ProfectusVax™ HIV-1 *gag/pol* and ProfectusVax™ HIV-1 *nef/tat/vif*, *env*. HIV-1 *gag/pol* expresses an HIV-1 clade B (HXB2) Gag-Pol fusion under the control of a human cytomegalovirus (hCMV) promoter and bovine growth hormone (BGH) polyadenylation signal. HIV-1 *nef/tat/vif*, *env* expresses (i) an HIV-1 clade B (NL43) Nef-Tat-Vif fusion under the control of an hCMV promoter and an SV40 polyadenylation signal; and (ii) an HIV-1 clade B primary isolate 6101 Env gp160 under the control of a simian cytomegalovirus (sCMV) promoter and BGH polyadenylation signal [35, 36]. The dose of HIV-MAG was 3,000 μg.

#### IL-12 plasmid (IL-12 pDNA) adjuvant

The HIV-MAG vaccine was administered with or without GENEVAX® *IL-12*-4532, a plasmid DNA adjuvant encoding the p35 and p40 subunits of human IL-12 [26, 31]. The p35 subunit is under the control of the hCMV promoter/enhancer and the SV40 polyadenylation signal. The p40 subunit is under the control of the sCMV promoter and the BGH polyadenylation signal. The doses of *IL-12* pDNA given were 0, 250, 1000, or 1500 μg. Both the HIV-MAG vaccine and the *IL-12* plasmid adjuvant are formulated in a citrate buffer containing 0.25% bupivacaine-HCl.

#### Administration by electroporation (EP)

The HIV-MAG vaccine, *IL-12* pDNA adjuvant, and placebo injections for the first 3 injection timepoints were delivered by intramuscular (IM) injection with EP using the Ichor Medical Systems TriGrid™ Delivery System (TDS) EP device. Activation of the hand-held integrated applicator, held against the deltoid, results in insertion of an injection needle and array of 4 conductive electrodes into the deltoid muscle, followed by IM injection of the vaccine or placebo, then propagation of a series of rectangular wave, direct current electrical pulses of 200 V/cm amplitude and 40 ms total duration in the area of distribution of the study product.

Each dose was divided and delivered as 2 injections, one into each deltoid.

#### rVSV_IN_N4CT1gag1 (VSV-Gag) vaccine

The VSV-Gag vaccine candidate is an attenuated recombinant vesicular stomatitis virus vector, Indiana serotype (rVSV_IN_), containing the HIV-1 (HXB2) *gag* p55 gene in an expression cassette adjacent to the viral 3’ transcription promoter. To attenuate the vaccine vector, the virus N gene was translocated from the first position in the genome to the fourth position (N4) and the virus G protein cytoplasmic tail (CT) was truncated from 29 amino acids to one amino acid (CT1). The HIV-1 Gag gene was inserted at position one (gag1) in the rVSV genome adjacent to the viral messenger RNA (mRNA) transcription promoter for maximum expression [37]. The vaccine vector was formulated in a phosphate buffer containing gelatin as a virus stabilizer and stored at -80°C. Each 3.4 ⊆ 10^7^ PFU dose was divided and delivered as 2 injections, one into each deltoid, by standard IM injection with needle and syringe [8].

#### Placebo for HIV-MAG vaccine, *IL-12* pDNA adjuvant, and VSV-Gag

The placebo used for all study products was Sodium Chloride for Injection, USP 0.9%. The placebos matched the vaccine injections in volumes and delivery methods.

### Safety and tolerability assessments

Participants first rated their injection site pain on a 10-point visual analog scale (VAS), a 10 centimeter (cm) line with one end marked “No Pain” and the other end marked “Worst Pain”, immediately and 5 and 25–60 minutes following study agent administration. The distance (cm) from the “No Pain” end was recorded as the pain score [38]. Local injection site and systemic reactogenicity signs and symptoms were also assessed around 30 (25–60) minutes after injections, and then self-reported by participants daily for the next 3 days after EP injections, or for 7 days following VSV-Gag/placebo injections. Solicited symptoms included: injection site pain, tenderness, erythema, induration or swelling, malaise and/or fatigue, myalgia, headache, chills, arthralgia, nausea, and vomiting. Adverse events (AEs) were reported for 15 months of participation. Additional contacts at months 24 and 36 solicited information on serious adverse events, other important medical events, new chronic conditions, HIV infection and pregnancy. Reactogenicity symptoms and AEs were scored using the Division of AIDS Table for Grading the Severity of Adult and Pediatric Adverse Events (version 1.0, December 2004; clarification August 2009). As a measure of EP acceptability, 2 weeks following each injection, participants completed a 2-item questionnaire that asked their willingness to undergo electroporation if it were required for a new vaccine against a serious disease if they were at risk for that disease, or if it increased the effectiveness of a currently existing vaccine such as the influenza vaccine.

Routine clinical laboratory tests included: complete blood count with differential and platelets (CBC), T-cell subsets, alanine aminotransferase, aspartate aminotransferase, alkaline phosphatase, creatinine, creatine kinase and urinalysis. For any participant with symptoms of a systemic viral syndrome following VSV-Gag/placebo injection, blood, urine, and saliva were sampled, up to 7 days post-vaccination. Oral swabs were collected from any oral mucosal lesions detected within 2 weeks post-vaccination with VSV-Gag/placebo. The samples were frozen in virus stabilizer, batched, and assessed for the presence of replication-competent rVSV by incubation on Vero cell monolayers, which were examined by microscopy for the presence of VSV-induced cytopathic effect. The presence of rVSV was subject to confirmation by nucleotide sequence analysis and VSV serotyping of virus isolates. Oral swabs were also tested for presence of rVSV by RT-PCR. Serum samples from baseline and from 2 weeks after the third DNA vaccination were tested for IL-12 neutralization activity as previously described to assess any vaccination-induced IL-12 neutralizing antibodies (S1 Methods) [31]. To identify any possible cases of rVSV induced encephalitis, a Mini-Mental State Examination was performed on all participants at baseline and at several timepoints post-VSV-Gag/placebo administration [39].

#### Whole blood phenotyping

Leukocyte populations were enumerated at multiple time-points using whole blood Trucount staining methods (S2 Methods) [40, 41]. Absolute cell counts were assessed on the day of the first HIV-MAG DNA vaccination (day 0) and 1, 3, and 14 days later, as well as on the day of VSV-Gag vaccination (day 168) and 1, 3, 7 and 14 days later.

### Statistical methods

Sample sizes were chosen to provide reasonable precision in the assessment of the primary safety and immunogenicity endpoints. The safety data from all 100 enrolled participants were analyzed according to the initial randomization assignment regardless of how many vaccinations they received. Since enrollment was concurrent with receiving the first vaccination, all participants received at least one vaccination and therefore provided some safety data.

The number and percentage of participants experiencing each type of local and systemic reactogenicity sign or symptom were tabulated by severity and treatment arm and graphically displayed. For a given sign or symptom, each participant’s reactogenicity was counted once under the maximum severity for prime and boost vaccination separately. A Kruskal-Wallis test was used to test for differences in severity across arms.

The mean and 95% CI of VAS pain scores were plotted over 0, 5–7 and 25–60 minutes after each vaccination by treatment arm. The 95% CI was estimated by assuming a t distribution with n-1 degrees of freedom. VAS scores were compared between treatment arms using t-test and between visits using paired t-test.

Differences in the frequency of AEs and frequency of responses indicating the acceptability of EP were compared between treatment arms using Fisher exact tests. All tests were two-sided, and the differences were considered to be statistically significant if *P* < .05 without multiplicity adjustments.

Individual plots were generated to display the trajectories of the blood lymphocyte, neutrophil counts, and cell populations measured by Trucount after the first DNA vaccination and last VSV vaccination. The difference between time points was tested using a Wald test in a linear mixed model that accounted for the correlations between the observations over time within individuals[42].

The data analysis and plots for this paper were generated using R version 3.4.1 for Unix, Copyright 2017, The R Foundation for Statistical Computing (https://www.r-project.org) and SAS software, Version 9.4 for Unix, Copyright 2002–2012, SAS Institute Incorporated. SAS and all other SAS Institute Inc. product or service names are registered trademarks or trademarks of SAS Institute Inc., Cary, NC, USA.

## Results

### Study population characteristics

**Fig 1:**
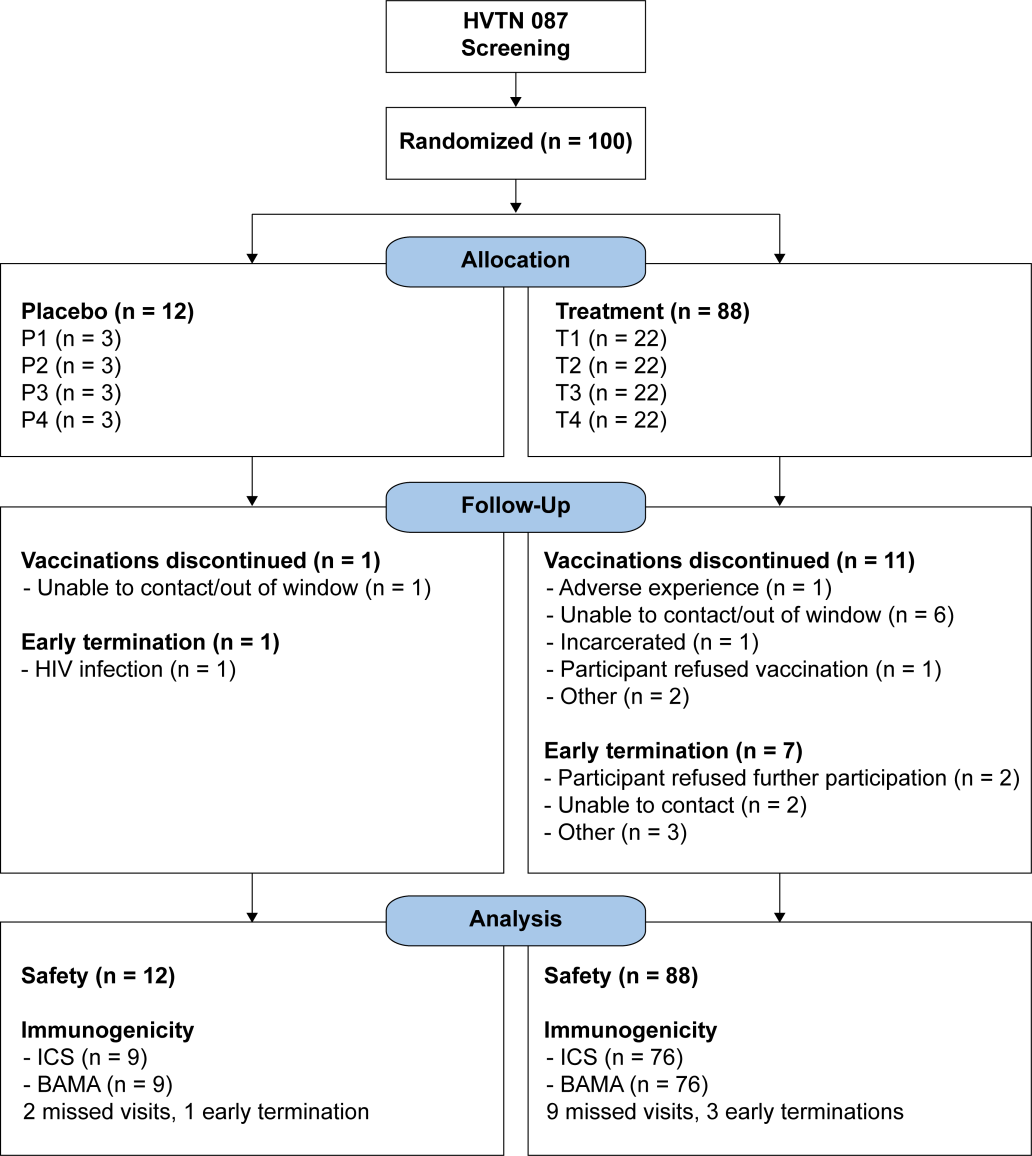
HVTN 087 CONSORT flow diagram.

HVTN 087 enrolled 100 participants—88 vaccine and 12 placebo recipients. The median age of participants was 28 years (range 18–49), and 35% were female. The majority were white (59%) or African-American (24%); 17% identified as Asian, another race, or more than one race; 15% of participants also identified as Hispanic (Table 2). Of the 100 participants, 72 received bilateral vaccinations at all 4 vaccination visits, per protocol. Table 2 shows the vaccination frequency by vaccination visit. Vaccine recipients are indicated as T1, T2, T3 and T4 with the numeral corresponding to their group assignment (Table 1). The 3 placebo recipients in each of the 4 groups were combined for analysis (CTL). The table tallies all participants who received any vaccination at that visit. These counts therefore also include 11 partial vaccinations, in which participants received only one of two scheduled injections at a visit due to technical difficulties with the EP device. Overall, 90% of the 800 expected deltoid injections were administered. Ninety-two (92%) participants completed follow-up. Twelve participants (11 vaccinees and 1 placebo) discontinued vaccinations early (Fig 1). Eight (7 vaccinees and 1 placebo) were terminated from the study early.

**Table 2:** Study population baseline characteristics.

		CTL (n = 12)	T1 (n = 22)	T2 (n = 22)	T3 (n = 22)	T4 (n = 22)	Total (n = 100)
Sex							
	Male	7 (58%)	13 (59%)	14 (64%)	14 (64%)	17 (77%)	65 (65%)
	Female	5 (42%)	9 (41%)	8 (36%)	8 (36%)	5 (23%)	35 (35%)
Ethnicity							
	Hispanic or Latino/a	3 (25%)	6 (27%)	3 (14%)	0 (0%)	3 (14%)	15 (15%)
	Not Hispanic or Latino/a	9 (75%)	16 (73%)	19 (86%)	22 (100%)	19 (86%)	85 (85%)
Race							
	White	8 (67%)	12 (55%)	11 (50%)	14 (64%)	14 (64%)	59 (59%)
	Black/African American	1 (8%)	6 (27%)	8 (36%)	7 (32%)	2 (9%)	24 (24%)
	Asian	0 (0%)	0 (0%)	0 (0%)	1 (5%)	0 (0%)	1 (1%)
	Multiracial	1 (8%)	1 (5%)	2 (9%)	0 (0%)	2 (9%)	6 (6%)
	Other	2 (17%)	3 (14%)	1 (5%)	0 (0%)	4 (18%)	10 (10%)
Age (Years)							
	18–20	0 (0%)	1 (5%)	2 (9%)	0 (0%)	4 (18%)	7 (7%)
	21–30	9 (75%)	7 (32%)	11 (50%)	12 (55%)	13 (59%)	52 (52%)
	31–40	3 (25%)	4 (18%)	4 (18%)	5 (23%)	1 (5%)	17 (17%)
	41–50	0 (0%)	10 (45%)	5 (23%)	5 (23%)	4 (18%)	24 (24%)
	Median	26.5	36.0	28.5	27.5	25.0	28.0
	Range	21–37	20–49	19–49	21–49	18–47	18–49
Vaccination Frequencies							
	Day 0	12 (100%)	22 (100%)	22 (100%)	22 (100%)	22 (100%)	100 (100%)
	Day 28	11 (92%)	20 (91%)	19 (86%)	20 (91%)	22 (100%)	92 (92%)
	Day 84	10 (83%)	20 (91%)	19 (86%)	21 (95%)	21 (95%)	91 (91%)
	Day 168	9 (75%)	19 (86%)	19 (86%)	19 (86%)	18 (82%)	84 (84%)

### Pain scores and reactogenicity

**Fig 2:**
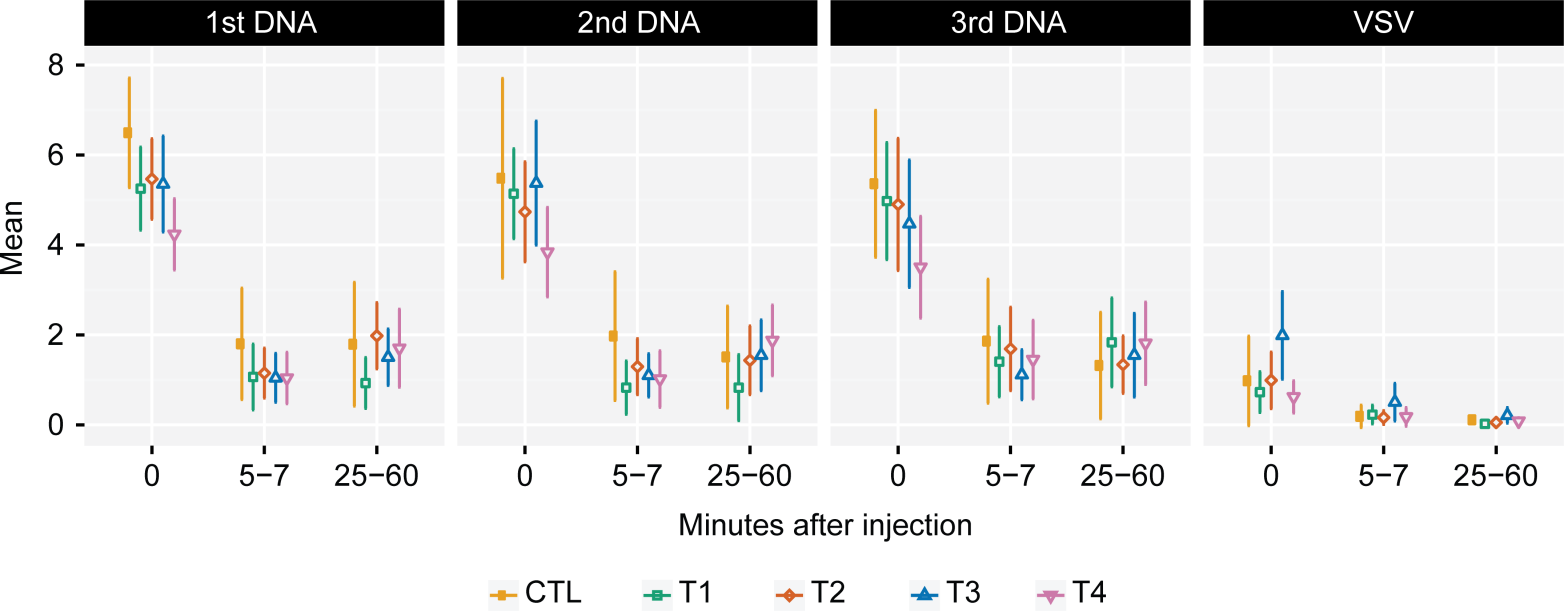
Visual analog scale pain scores after DNA/placebo and VSV-Gag/placebo vaccine delivery. Participants rated their pain between 0 (no pain) and 10 (worst possible pain). The graph shows the mean and 95% CI of VAS scores at 3 timepoints indicating minutes after injection, shown by injection visits and treatment arms. The 95% CI was estimated using t-distribution with n-1 degrees of freedom. Pain scores were maximal at 0 minutes after electroporation, and significantly lower in T4 compared to other treatment arms at that timepoint.

Visual analog scale (VAS) pain scoring after injections with EP showed maximal pain scores immediately after vaccination (i.e. associated with the application of the electrical stimulation) (Fig 2). These initial pain scores after EP were similar across T1-T3 and placebo controls but were significantly lower in T4 (highest dose of *IL-12* pDNA) compared with other treatment arms (p = 0.01, 0.03, 0.04 after the first, the second, and the third DNA vaccination, respectively). The mean pain score after EP rapidly decreased from 4.9 to 1.3 (on a scale of 0–10) at 5 minutes and were subsequently stable (mean 1.5) at 25–60 minutes (median 1.6) after each vaccination. Pain scores at 5 and 25–60 minutes after injections were not significantly different between treatment arms and between EP vaccinations. Standard IM injection, used for VSV-Gag or placebo, produced relatively little pain, with a median pain score of 1.0 immediately after injection, decreasing to 0.3 at 5 minutes and 0.1 at 25–60 minutes (Fig 2).

**Fig 3:**
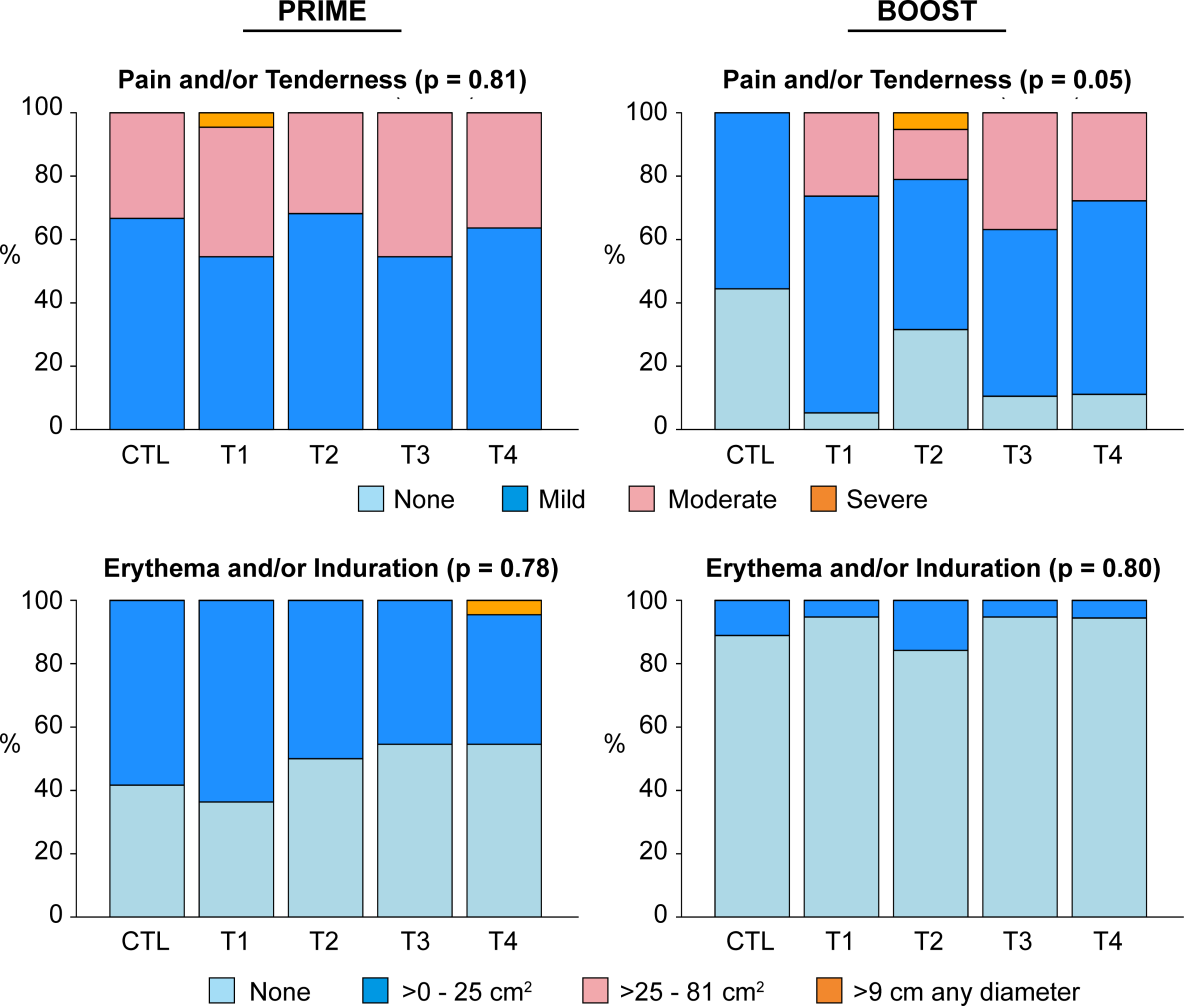
Maximum local reactogenicity, prime vs boost, by treatment group. Bar graphs show the percentage of participants in each treatment group reporting the specified maximum severity during the reactogenicity period. Left panels (Prime) indicate the maximum severity over all 3 priming injections. P values indicated are for comparisons across all treatment arms. The increased reactogenicity of the Prime compared to Boost is significant for T1-T4 (p<0.01), and the increased reactogenicityexperienced by T1-T4 compared to placebo for the VSV-Gag boost, upper right panel, is significant (p = 0.01).

Local injection site reactogenicity (Fig 3, left panels) after HIV-MAG prime with EP was not significantly different across treatment arms (p = 0.81 and 0.78 for pain and/or tenderness, and erythema and/or induration, respectively). Local pain and/or tenderness after VSV-Gag boost (Fig 3, upper right panel) was significantly different across arms (p = 0.05); severity was significantly higher in T1-T4 compared to placebo (p = 0.01) but not significantly different between *IL-12* pDNA dose groups (p = 0.40). Local erythema and/or induration after VSV-Gag boost (Fig 3, lower right panel) was not significantly different across all treatment arms (p = 0.80). Maximal local reactogenicity was significantly higher in severity after HIV-MAG prime with EP compared to after VSV-Gag boost in the T1-T4 groups (p<0.01 for both pain and/or tenderness, and erythema and/or induration). Among placebo recipients there was a trend towards increased reactogenicity between prime (EP) and boost (standard IM) injections (p = 0.063 for both pain and/or tenderness, and erythema and/or induration)—suggesting increased injection site symptoms were an effect of EP as opposed to the presence of HIV-MAG vaccine or *IL-12* pDNA adjuvant.

**Fig 4:**
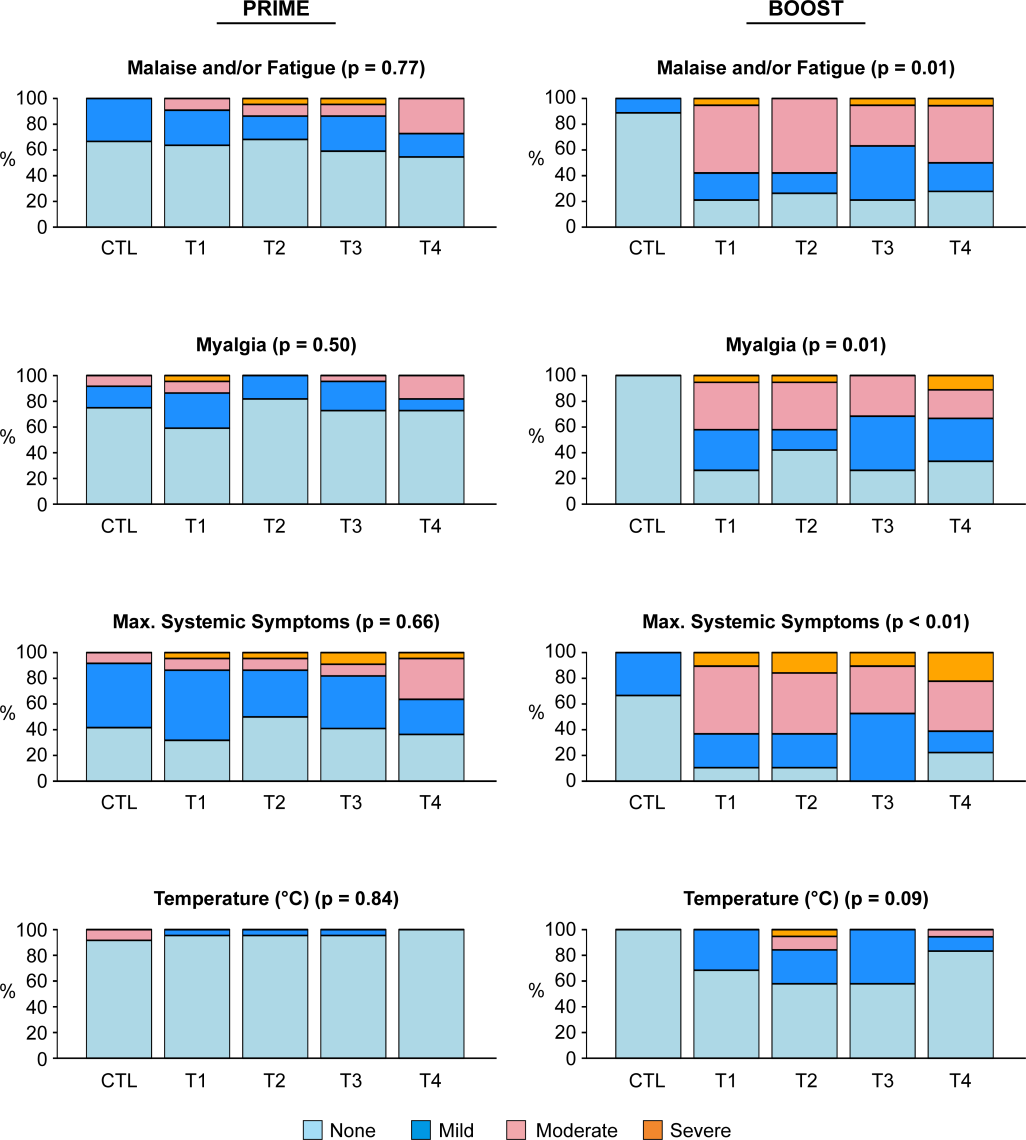
Maximum systemic reactogenicity, prime vs boost, by treatment group. Bar graphs show the percentage of participants in each treatment group reporting the specified maximum severity during the reactogenicity period. Left panels (Prime) indicate the maximum severity over all 3 priming injections. P values indicated are for comparisons across all treatment arms. Maximum systemic symptoms were significantly more severe in T1-T4 groups than the placebo group following the VSV-Gag boost.

Systemic reactogenicity was not significantly different across treatment arms after DNA prime, but was more common and more severe after VSV-Gag boost compared to placebo controls (p<0.01, Fig 4). The majority of participants had mild or moderate systemic symptoms. Malaise and/or fatigue and myalgia were most frequently reported. Eleven of 75 (15%) participants reported at least one severe systemic reactogenicity symptom after VSV-Gag, compared to 5 of 88 (6%) participants who experienced a severe symptom after HIV-MAG. Thirty-one participants out of 75 (41%) who received VSV-Gag experienced a viral syndrome of fever, chills, malaise/fatigue, myalgia, headache during the 7-day reactogenicity period following VSV-Gag injection, and had samples of blood, urine, and saliva tested for rVSV. In addition, 10 participants had swabs of oral lesions tested. All samples were negative for infectious rVSV (culture) and rVSV RNA (RT-PCR).

The dose of *IL-12* pDNA had no significant effect on the maximum severity of systemic reactogenicity symptoms across treatment groups (T1-T4). Placebo controls had similar systemic reactogenicity after EP delivery compared to needle and syringe injections (p = 0.375).

Three participants discontinued IM EP vaccinations for reasons of intolerability: one for injection site reactogenicity symptoms, one for intense pain, and one after an event of presyncope.

### Adverse events

Thirty-nine participants in treatment groups (T1-T4) experienced at least one adverse event that was reported as related to the vaccine, including decreased lymphocyte and neutrophil counts, injection site bruising, elevated aspartate aminotransferase, presyncope and mouth ulceration (Table 3). Sixteen participants had decreases in lymphocyte count, discussed further below. Eight participants had mild or moderate decreased neutrophil counts, which in most cases occurred 3 days after VSV-Gag vaccination and resolved by Day 7 (7 events).

**Table 3:** Related adverse events in treatment groups with or without IL-12 pDNA adjuvant.

	*T1 (without IL-12)*		*T2-T3-T4 (with IL-12)*		
*Preferred Term*	*N*	*%*	*N*	*%*	*P-value*
Participants with one or more AEs	10	45.5%	29	43.9%	1.0
Lymphocyte count decreased*	8	36.4%4	8	36.4%	1.0
Neutrophil count decreased*	2	9.1%	5	22.7%	0.41
Injection site bruising	0	0.0%	4	6.1%	0.57
Presyncope	0	0.0%	3	4.5%	0.57
Aspartate aminotransferase increased	0	0.0%	2	3.0%	1.0
CD4 lymphocytes decreased	2	9.1%	0	0.0%	0.06
Mouth ulceration	1	4.5%	1	1.5%	0.44
White blood cell count decreased*	1	4.5%	1	4.5%	1.0
1 each: Abdominal pain, ALT increased, Anemia, Hemoglobin decreased, Dyspnea, Anxiety, Injection site erythema, Injection site pain, Musculoskeletal stiffness, Night sweats, Oral disorder, Oral herpes, Panic attack, Palpitations, Paresthesia, Photophobia, Tongue ulceration, Viral infection	0	0.0%	1	1.5%	
1 each: Blood creatinine increased, Fatigue, Myalgia, Oral papule	1	4.5%	0	0.0%	

*Comparison between T1 and T3. T2 and T4 did not have blood counts at early post-vaccination time points.

There were no statistically significant differences in adverse events between the group that did not receive *IL-12* pDNA (T1) and the groups that did receive it (T2-T4). There were changes in leukocyte indices related to additional sampling in T1 and T3 at timepoints 1 and 3 days after VSV-Gag injections, which were not assessed in T2 and T4 (Table 3). Mouth ulceration was seen in 5 participants, and considered related to vaccine in 2 cases, although oral swabs were negative for VSV. No arthritis or skin lesions were noted after VSV-Gag boost. There were 5 serious (Grade 3 or 4) adverse events (SAEs) in the study, all deemed not related to study product, including borderline mucinous tumor of the ovary (in a control participant), Grade 4 elevation in CPK, esophageal obstruction, intervertebral disc protrusion, and suicidal ideation. Other Grade 3 or 4 AEs were deemed to be neither SAEs nor related to vaccine: alcoholic hangover, stress, migraine, elevation in CPK, viral infection, headache, and influenza infection.

MMSE testing and clinical observations throughout the study showed no evidence of encephalitis or other mental status changes.

Although the study enrolled individuals at low risk of HIV, 2 individuals were diagnosed with HIV infection during the study. Retrospective testing showed that one individual (in the control group) was already HIV-infected at the time of enrollment; the second individual was diagnosed with HIV infection at the final clinic visit.

No pregnancies were reported.

#### Transient changes in leukocyte trafficking after VSV-Gag

**Fig 5:**
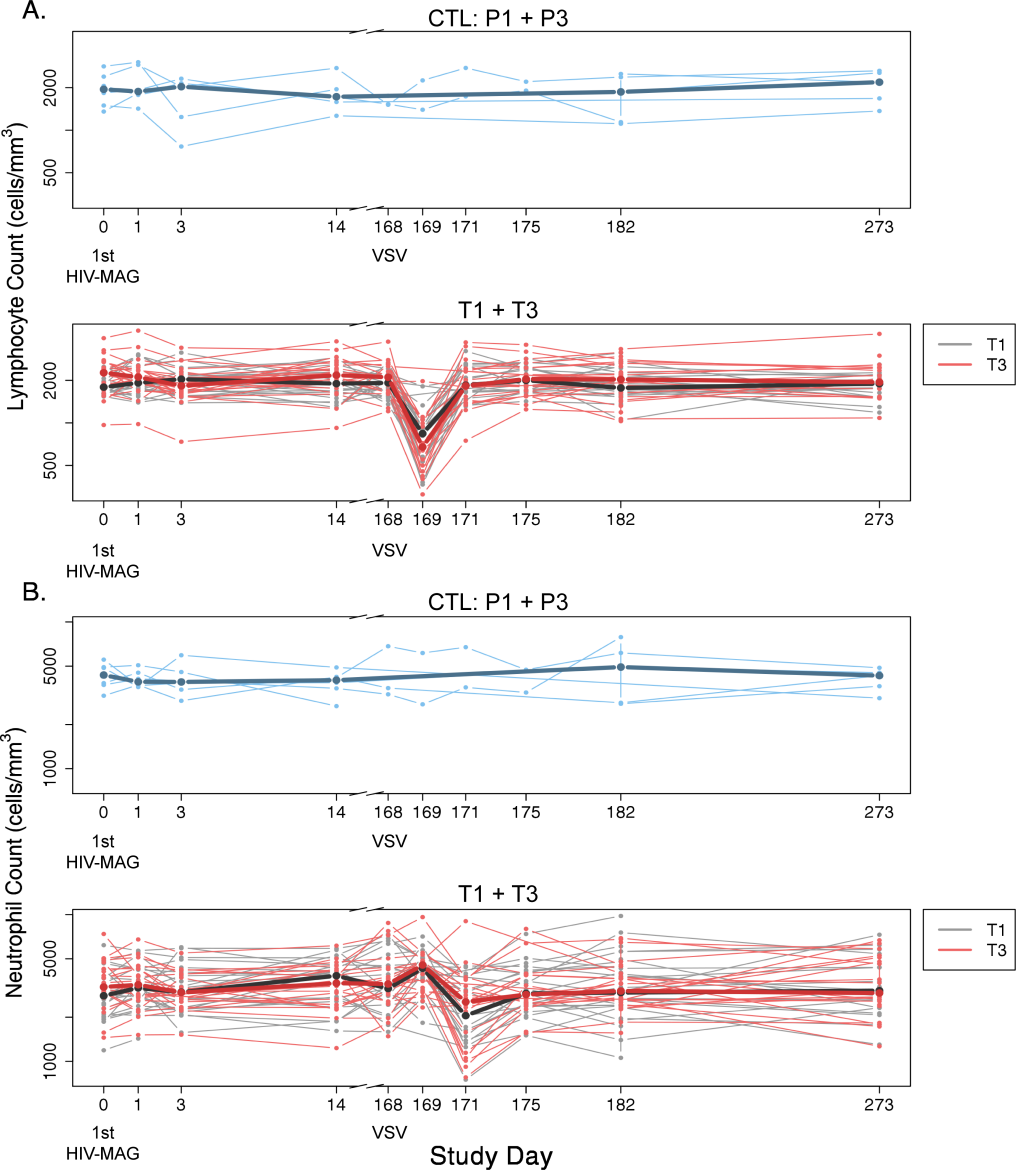
Decreases in peripheral blood absolute lymphocyte counts and absolute neutrophil counts after VSV-Gag. Counts at 1 and 3 days after VSV-Gag boost were assessed for Groups 1 and 3 only. Placebo data from P1 and P3 are pooled, shown in blue. Data from T1 and T3 are displayed together, with T1 values in black and T3 in red. Bold lines represent median values for each treatment group, superimposed on the individual profiles.

In the subset of participants (T1 and T3) who had blood collected for CBC and immunophenotyping at additional early timepoints immediately after vaccination—Days 1 and 3 after the first DNA prime, and at Days 1, 3, and 7 after VSV-Gag boost—changes in leukocyte trafficking were seen. A significant drop in median absolute lymphocyte count (ALC) was detected at Day 1 post-VSV-Gag (day 169, p<0.0001), and a drop in median absolute neutrophil count (ANC) was seen at Day 3 (day 171, p<0.0001) (Fig 5). At Day 1 post-VSV-Gag, decreased lymphocytes were detected in 16 participants: 5 Grade 2 and 3 Grade 3 in T1; 1 Grade 1, 1 Grade 2, 5 Grade 3, and 1 Grade 4 in T3. The median ALC recovered to normal range by the next assessment at Day 3 post-VSV-Gag and remained normal to the last ALC assessment 3.5 months later (Fig 5). Decreased ANCs, Grade 1 or 2, were seen in 7 participants at Day 3 after VSV-Gag boost. The ANCs recovered to the normal range within 4–6 days (Fig 5). On Day 14 after VSV-Gag boost, 2 people had decreased ANCs, including one participant whose ANC had recovered on Day 7 earlier, and one participant from Group 2. Transient decreases in platelet counts were also noted on Day 3 after VSV-Gag boost (median change from baseline, -16,000 cells/mm^3^ in T1 and -29,000 cells/mm^3^ in T3); no participant had a decrease that met criteria for reporting as an AE. These changes were not seen in placebo recipients (median change from baseline, +31,000 cells/mm^3^), although the sample size (2) was small.

#### Other changes to cell populations

**Fig 6:**
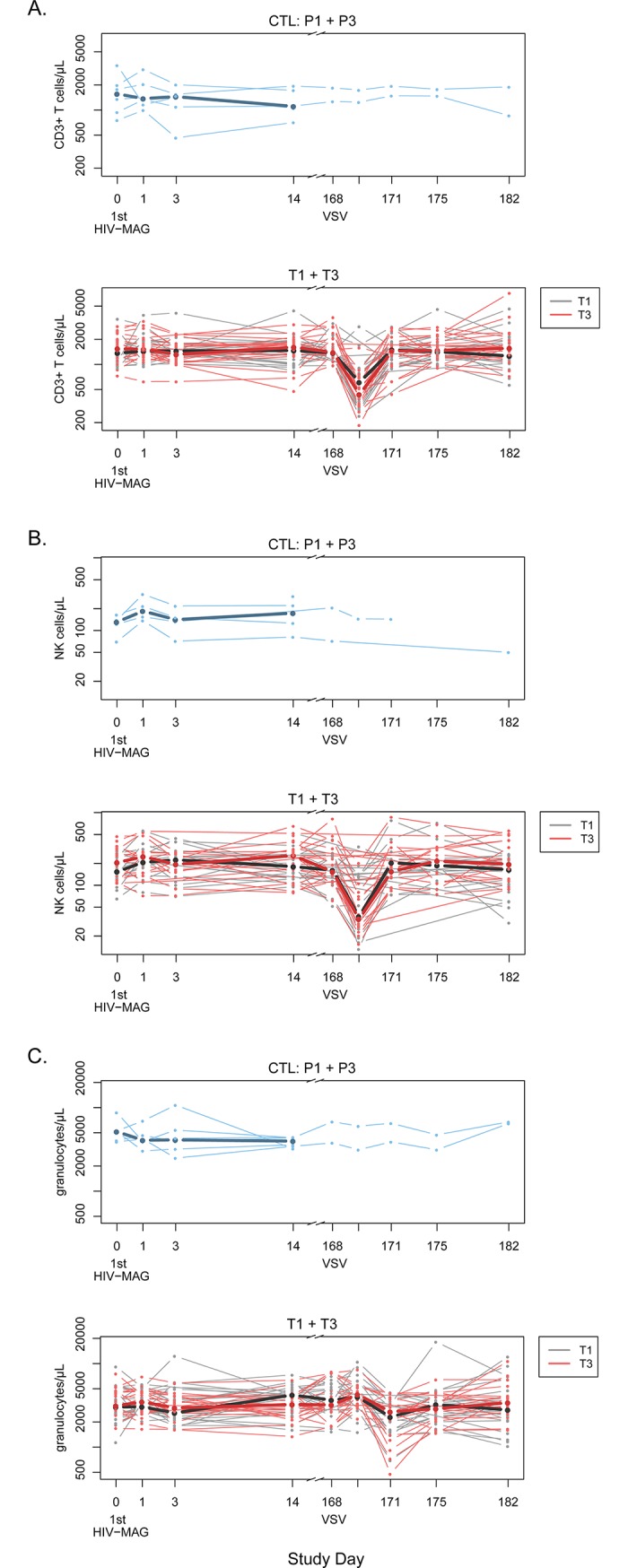
Changes in numbers of cell populations assessed by Trucount™ after vaccination. Absolute counts for CD3+ T cells (A), NK cells (B) and granulocytes (C) are shown. Placebo data from P1 and P3 are pooled, shown in blue. Data from T1 and T3 are displayed together, with T1 values in black and T3 in red. Bold lines represent median values for each treatment group, superimposed on the individual profiles.

Kinetic fluctuations in leukocyte populations after VSV-Gag inoculation were confirmed by Trucount™ whole blood immunophenotyping in vaccine recipients from Groups 1 and 3 (Fig 6). As observed in the clinical monitoring of participants, there was a decline in overall lymphocytes 1 day after VSV-Gag vaccination (day 169, p<0.001 for both groups) that rebounded by 3 days post-vaccination (day 171). This pattern was consistently detected across lymphocyte subsets including total CD3^+^ T cells and NK cells (Fig 6, day 169, p<0.001 for both subsets), CD4^+^ T cells, CD8^+^ T cells and B cells (p<0.001 for all subsets, S1 Table). In contrast, transient increases in the number of monocytes were observed 1 day after VSV-Gag and declines in granulocytes were observed 3 days after VSV-Gag inoculation (day 171, p<0.001). Additionally, there were no differences detected between T1 and T3 at the timepoints studied for any of the cell types evaluated, suggesting that *IL-12* did not impact the global frequencies of leukocytes. Consistent with the CBC results, no significant changes were observed after DNA vaccination with or without *IL-12*.

### IL-12 neutralizing antibody

No vaccination-induced IL-12 neutralizing antibodies were detected in any participant (S1 Methods, and S1 Fig).

### Acceptability of electroporation

**Fig 7:**
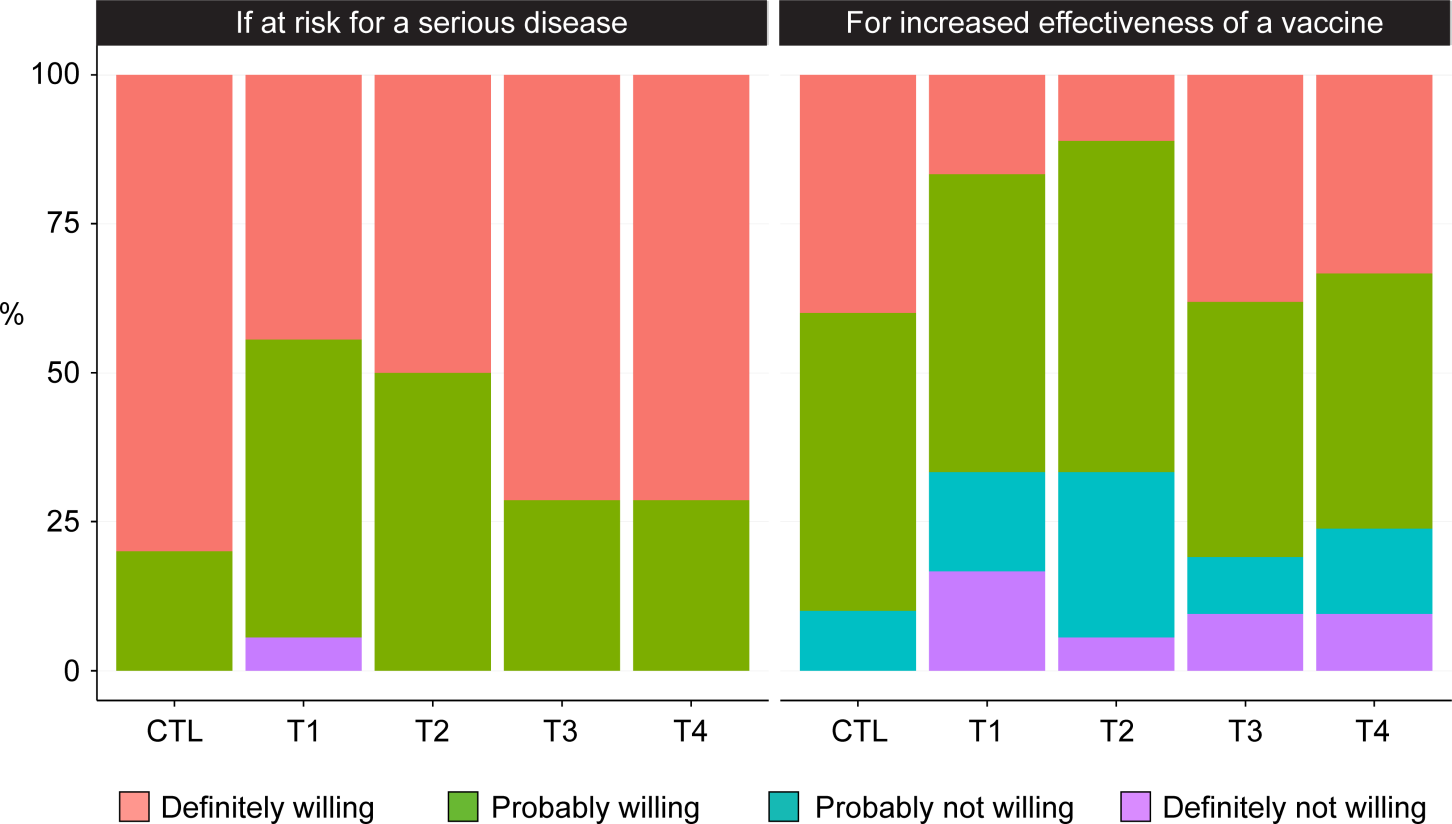
Willingness to undergo electroporation, by treatment group. Bar graphs show the percentage of participants in each treatment group reporting willingness to undergo EP, in response to these questions, as assessed 2 weeks after the last injection with EP: Left panel: How willing would you be to undergo electroporation if it were required for a new vaccine against a serious disease if you were at risk for that disease? Right panel: How willing would you be to undergo electroporation if it increased the effectiveness of a vaccine we already have, such as the influenza vaccine?

EP was generally well accepted. At visit 10 (2 weeks after the last DNA), 88 participants (including 10 placebo recipients) completed the question about their willingness to undergo the procedure for a new vaccine against a serious disease for which they were at risk, and 87 (98.9%) responded “definitely” or “probably willing”; only one (1.1%) responded “not willing”. When asked if they were willing to undergo the procedure in order to increase the effectiveness of a currently available vaccine, most (66/88, or 75%) were still “definitely” or “probably willing” to undergo EP; however, 22 of 88 (25%) reported being “probably not” or “definitely not willing”. There were no differences in willingness between vaccine treatment groups (p = 0.78) or between recipients of vaccines and placebo. (Fig 7).

### Social impacts

During the study, 2 participants (2%) reported social impacts, which affected personal relationships: a misunderstanding with a partner, and a relative who disapproved of participation. The participants reported these events as having a minimal effect on quality of life, and these events resolved.

## Discussion

Vaccination with plasmid DNA has appeared to yield mostly poor immune responses in humans, as detected by standard immunogenicity measures such as the intracellular cytokine staining (ICS) assay. Enhancement of DNA vaccination through electroporation, cytokine adjuvants, and use of heterologous boosts have separately been shown to improve immunogenicity. HVTN 087 has now demonstrated the feasibility, safety, and tolerability of combining these strategies in a vaccine regimen in healthy HIV-uninfected adults. No significant differences in local or systemic reactogenicity were seen between the treatment groups that received *IL-12* pDNA (T2, T3, T4) and the group that received no *IL-12* pDNA (T1), and no dose-related increase in AEs was found, suggesting that administration of *IL-12* pDNA via electroporation is safe and very well tolerated in doses up to 1500 μg. The study did not identify a maximum tolerated dose, which could be higher.

Delivery of HIV-MAG injections via IM EP elicited more local pain and tenderness compared to the VSV-Gag boost which was delivered by standard IM injection. Pain and tenderness following EP injections were observed in placebo controls at the same rate as in vaccinees, suggesting that the delivery method rather than the HIV-MAG vaccine was primarily responsible for these symptoms. Encouragingly, immediate EP-associated pain was transient, generally resolving within 25 minutes, and all but one of the participants indicated a willingness to accept vaccination by IM EP for a serious disease for which they were at risk. The observation of lower pain scores in the high dose IL-12 group was likely due to the increased volume of bupivacaine formulation administered, which may be a potential avenue for reducing the acute discomfort associated with EP mediated delivery.

Participants receiving VSV-Gag injections reported more severe systemic symptoms than those receiving placebo injections, including a viral syndrome in some participants—a finding which has been observed with other vaccine studies utilizing rVSV vectors [8, 9, 12]. Unlike some of those studies, however, we did not see any notable arthralgia, nor a significant increase in oral ulcerations, arthritis or skin lesions in vaccine recipients compared with controls, and we did not detect either replicating rVSV or viral RNA in any samples of blood, urine and saliva. There were no concerning AEs related to the VSV vector.

VSV-Gag injections were associated with transient lymphopenia on Day 1 after injection which resolved by Day 3. Neutrophils similarly decreased on Day 3. The decrease in lymphocyte and neutrophil counts were not associated with any clinical adverse events. Decreased lymphocytes in the peripheral blood were observed both in the clinical safety data (complete blood counts) as well as in the more refined Trucount analysis. Groups 2 and 4 were not assessed at those timepoints, but would be expected to have shown the same effect. A similar decrease was also seen in the Ebola vaccine trials of rVSV-ZEBOV, suggesting a significant innate immune response to rVSV that may influence the trafficking of lymphocytes out of the blood, presumably into lymphoid compartments, and thus impact the ensuing adaptive immune response [9, 43]. Decreased lymphocytes have been rarely reported with other vaccine trials, but few studies measure blood counts at these early timepoints [9, 44]. Decreased neutrophils have been reported as a transient, clinically asymptomatic finding with other vaccines, without known complications [45].

The study was limited in that it did not test higher doses of *IL-12* pDNA which might have been well-tolerated and effective at increasing immunogenicity, and it did not include a comparison to priming with DNA vaccination using conventional IM injection. Importantly, this study has confirmed that the attenuated rVSV_IN_ vector is safe in healthy adults and should be considered as a potential vector platform in further vaccine studies.

## Supporting information

S1 CONSORT Checklist (DOC)

S1 Protocol (PDF)

S1 Methods IL-12 neutralizing antibody assessment. (DOCX)

S2 Methods Whole blood phenotyping. (DOCX)

S1 Fig IL-12 neutralization antibody titers at baseline and after 3 DNA vaccinations. The percentage and frequency of responders is indicated above each plot. Red circles indicate positive responses (responders); blue triangles represent responses below the cutoff for positivity (non-responders).
(TIF)

S1 Table T1 and T3 Trucount cell population changes over time after the VSV vaccination given at Day 0. Details of the decrease in lymphocyte and monocyte counts 1 day after VSV-Gag vaccination (p<0.001 for both groups), which rebounded by 3 days post-vaccination. Days indicated in the table are days following VSV vaccination.
(DOCX)

S1 Dataset (ZIP)
