# Supplementary material for: Safety and tolerability of HIV-1 multiantigen pDNA vaccine given with IL-12 plasmid DNA via electroporation, boosted with a recombinant vesicular stomatitis virus HIV Gag vaccine in healthy volunteers in a randomized, controlled clinical trial
Source: PLoS One. 2018 Sep 20;13(9):e0202753. doi: 10.1371/journal.pone.0202753 (PMC6147413; doi:10.1371/journal.pone.0202753)
Supplement: S1 Protocol — (PDF) [file pone.0202753.s002.pdf]

HIV VACCINE
TRIALS NETWORK

PROTOCOL

HVTN 087

A phase 1 trial to evaluate the safety, tolerability, and immunogenicity of an *IL-12* pDNA enhanced HIV-1 multiantigen pDNA vaccine delivered intramuscularly with electroporation, with an HIV-1 rVSV vaccine boost, in healthy HIV-uninfected adult participants

DAIDS DOCUMENT ID 11673

BB IND [INS # OR DELETE] HELD BY DAIDS

CLINICAL TRIAL SPONSORED BY

Division of AIDS (DAIDS)
National Institute of Allergy and Infectious Diseases (NIAID)
National Institutes of Health (NIH)
Department of Health and Human Services (DHHS)
Bethesda, Maryland, USA

STUDY PRODUCT(S) PROVIDED BY

Profectus Biosciences, Inc.
Tarrytown, New York, USA

December 27, 2011

HVTN 087, Version 1.0

Contents

1 Ethical considerations 5

2 IRB/EC review considerations 7
2.1 Minimized risks to participants 7
2.2 Reasonable risk/benefit balance 7
2.3 Equitable subject selection 7
2.4 Appropriate informed consent 8
2.5 Adequate safety monitoring 8
2.6 Protect privacy/confidentiality 8

3 Overview 9
3.1 Protocol Team 13

4 Background 14
4.1 Rationale for trial concept 14
4.2 HIV-MAG vaccine 15
4.3 *IL-12* pDNA adjuvant 16
4.4 VSV HIV *gag* 17
4.5 Electroporation 18
4.6 Trial design rationale 19
4.7 Plans for future product development and testing 20
4.8 Preclinical safety studies 21
4.9 Preclinical immunogenicity studies 29
4.10 Clinical studies 32
4.11 Potential risks of study products and administration 47

5 Objectives and endpoints 50
5.1 Primary objectives and endpoints 50
5.2 Secondary objectives and endpoints 50
5.3 Exploratory objectives 51

6 Statistical considerations 52
6.1 Accrual and sample size calculations 52
6.2 Randomization 56
6.3 Blinding 57
6.4 Statistical analysis 57

7 Selection and withdrawal of participants 62
7.1 Inclusion criteria 62
7.2 Exclusion criteria 64
7.3 Participant departure from vaccination schedule or withdrawal 67

8 Study product preparation and administration 70
8.1 Vaccine regimen 70
8.2 Study product formulation 73
8.3 Preparation of study products 74
8.4 Administration 83
8.5 Acquisition of study products 84
8.6 Pharmacy records 85
8.7 Final disposition of study products 85

9 Clinical procedures 86
9.1 Informed consent 86

9.2 Pre-enrollment procedures 88
9.3 Enrollment and vaccination visits 89
9.4 Follow-up visits 91
9.5 Month 15 health contact 92
9.6 Annual health contacts 93
9.7 HIV counseling and testing 93
9.8 Contraception status 95
9.9 Urinalysis 95
9.10 Assessments of reactogenicity 95
9.11 Assessment of neurological safety 97
9.12 Assessment for generalized rVSV infection 97
9.13 Visit windows and missed visits 97
9.14 Early termination visit 98
9.15 Pregnancy 98

10 Laboratory 99
10.1 HVTN CRS laboratory procedures 99
10.2 Total blood volume 99
10.3 Primary immunogenicity timepoints 99
10.4 Endpoint assays: cellular 99
10.5 Endpoint assays: humoral 100
10.6 Innate immunity assays 100
10.7 Genotyping 101
10.8 Exploratory studies 101
10.9 Other use of stored specimens 102
10.10 Biohazard containment 102

11 Safety monitoring and safety review 103
11.1 Safety monitoring and oversight 103
11.2 Safety reporting 104
11.3 Safety reviews 106
11.4 Safety pause and prompt PSRT AE review 107
11.5 Review of cumulative safety data 108
11.6 Study termination 108

12 Protocol conduct 109
12.1 Social impacts 109
12.2 Compliance with NIH guidelines for research involving products containing recombinant DNA 110
12.3 Emergency communication with study participants 110

13 Version history 111

14 Document references (other than literature citations) 112

15 Acronyms and abbreviations 114

16 Literature cited 117

Appendix A Sample informed consent form 122

Appendix B Approved birth control methods (for sample informed consent form) 140

Appendix C HVTN VISP registry consent 141

Appendix D Table of procedures (for sample informed consent form) 144

Appendix E Laboratory procedures for Groups 1 and 3 145
Appendix F Laboratory procedures for Groups 2 and 4 146
Appendix G Procedures at HVTN CRS for Groups 1 and 3 147
Appendix H Procedures at HVTN CRS for Groups 2 and 4 148
Appendix I Procedures at CRS for annual health contacts 149
Appendix J Adverse Events of Special Interest (AESI) 150

1 Ethical considerations

Multiple candidate human immunodeficiency virus (HIV) vaccines will need to be studied simultaneously in different populations around the world before a successful HIV preventive vaccine is found. It is critical that universally accepted ethical guidelines are followed at all sites involved in the conduct of these clinical trials. The HIV Vaccine Trials Network (HVTN) has addressed ethical concerns in the following ways:

- HVTN trials are designed and conducted to enhance the knowledge base necessary to find a preventive vaccine, using methods that are scientifically rigorous and valid, and in accordance with Good Clinical Practice (GCP) guidelines.

- HVTN scientists and operational staff incorporate the philosophies underlying major codes [1-3], declarations, and other guidance documents relevant to human subjects research into the design and conduct of HIV vaccine clinical trials.

- HVTN scientists and operational staff are committed to substantive community input—into the planning, conduct, and follow-up of its research—to help ensure that locally appropriate cultural and linguistic needs of study populations are met. Community Advisory Boards (CAB) are required by DAIDS and supported at all HVTN research sites to ensure community input.

- HVTN clinical trial staff counsel study participants routinely on how to reduce HIV risk. Participants who become HIV-infected during the trial are provided counseling on notifying their partners and about HIV infection according to local guidelines. Staff members will also counsel them about reducing their risk of transmitting HIV to others.

- Participants who become HIV-infected during the trial are referred to medical practitioners to manage their HIV infection and to identify potential clinical trials they may want to join.

- The HVTN provides training so that all participating sites similarly ensure fair participant selection, protect the privacy of research participants, and obtain meaningful informed consent. During the study, participants will have their wellbeing monitored, and to the fullest extent possible, their privacy protected. Participants may withdraw from the study at any time.

- Prior to implementation, HVTN trials are rigorously reviewed by scientists who are not involved in the conduct of the trials under consideration.

- HVTN trials are reviewed by local and national regulatory bodies and are conducted in compliance with all applicable national and local regulations.

- The HVTN designs its research to minimize risk and maximize benefit to both study participants and their local communities. For example, HVTN protocols provide enhancement of participants' knowledge of HIV and HIV prevention, as well as counseling, guidance, and assistance with any social impacts that may result from research participation. HVTN protocols also include careful medical

review of each research participant's health conditions and reactions to study products while in the study.

- HVTN research aims to benefit local communities by directly addressing the health and HIV prevention needs of those communities and by strengthening the capacity of the communities through training, support, shared knowledge, and equipment. Researchers involved in HVTN trials are able to conduct other critical research in their local research settings.

- The HVTN recognizes the importance of institutional review and values the role of in country Institutional Review Boards (IRBs) and Ethics Committees (ECs) as custodians responsible for ensuring the ethical conduct of research in each local setting.

2 IRB/EC review considerations

US Food and Drug Administration (FDA) and other US federal regulations require IRBs or ECs to ensure that certain requirements are satisfied on initial and continuing review of research (Title 45, Code of Federal Regulations (CFR), Part 46.111(a) 1-7; 21 CFR 56.111(a) 1-7). The following section highlights how this protocol addresses each of these research requirements. Each HVTN Investigator welcomes IRB/EC questions or concerns regarding these research requirements.

2.1 Minimized risks to participants

45 CFR 46.111 (a) 1 and 21 CFR 56.111 (a) 1: Risks to subjects are minimized.

This protocol minimizes risks to participants by (a) correctly and promptly informing participants about risks so that they can join in partnership with the researcher in recognizing and reporting harms; (b) respecting local/national blood draw limits; (c) performing direct observation of participants postvaccination and collecting information regarding side effects for several days postvaccination; (d) having staff properly trained in administering study procedures that may cause physical harm or psychological distress, such as blood draws, vaccinations, HIV testing and counseling and HIV risk reduction counseling; (e) providing HIV risk reduction counseling and checking on contraception use (for women); and (f) providing safety monitoring.

2.2 Reasonable risk/benefit balance

45 CFR 46.111 (a) 2 and 21 CFR 56 (a) 2: Risks to subjects are reasonable in relation to anticipated benefits, if any, to subjects, and the importance of the knowledge that may reasonably be expected to result.

In all public health research, the risk-benefit ratio may be difficult to assess because the benefits to a healthy participant are not as apparent as they would be in treatment protocols, where a study participant may be ill and may have exhausted all conventional treatment options. However, this protocol is designed to minimize the risks to participants while maximizing the potential value of the knowledge it is designed to generate.

2.3 Equitable subject selection

45 CFR 46.111 (a) 3 and 21 CFR 56.111 (a) 3: Subject selection is equitable

This protocol has specific inclusion and exclusion criteria for investigators to follow in admitting participants into the protocol. Participants are selected because of these criteria and not because of positions of vulnerability or privilege. Investigators are required to maintain screening and enrollment logs to document volunteers who screened into and out of the protocol and for what reasons.

2.4 Appropriate informed consent

45 CFR 46.111 (a) 4 & 5 and 21 CFR 56.111 (a) 4 & 5: Informed consent is sought from each prospective subject or the subject's legally authorized representative as required by 45 CFR 46.416; informed consent is appropriately documented as required by 45 CFR 46.417

The protocol specifies that informed consent must be obtained before any study procedures are initiated and assessed throughout the trial (see Section 9.1). Each site is provided training in informed consent by the HVTN as part of its entering the HVTN. The HVTN requires a signed consent document for documentation, in addition to chart notes or a consent checklist.

2.5 Adequate safety monitoring

45 CFR 46.111 (a) 6 and 21 CFR 56.111 (a) 6: There is adequate provision for monitoring the data collected to ensure the safety of subjects.

This protocol has extensive safety monitoring in place (see Section 11). Safety is monitored daily by clinical affairs staff and routinely by the Protocol Safety Review Team (PSRT). Site staff have 24-hour cell phone access to clinical affairs staff. In addition, the HVTN Safety Monitoring Board (SMB) or a Data and Safety Monitoring Board (DSMB) periodically reviews study data.

2.6 Protect privacy/confidentiality

45 CFR 46.111 (a) 7 and 21 CFR 56.111 (a) 7: There are adequate provisions to protect the privacy of subjects and maintain the confidentiality of data.

Privacy refers to an individual's right to be free from unauthorized or unreasonable intrusion into his/her private life and the right to control access to individually identifiable information about him/her. The term "privacy" concerns research participants or potential research participants as individuals whereas the term "confidentiality" is used to refer to the treatment of information about those individuals. This protocol respects the privacy of participants by informing them about who will have access to their personal information and study data (see Appendix A). The privacy of participants is protected by assigning unique identifiers in place of the participant's name on study data and specimens. In the United States, research participants in HVTN protocols are protected by a Certificate of Confidentiality from the US NIH, which can prevent disclosure of study participation even when that information is requested by subpoena. Participants are told of the use and limits of the certificate in the study consent form. In addition, each staff member at each study site in this protocol signs a Confidentiality Agreement with the HVTN and each study site participating in the protocol is required to have a standard operating procedure on how the staff members will protect the confidentiality of study participants.

3 Overview

Title

A phase 1 trial to evaluate the safety, tolerability, and immunogenicity of an *IL-12* pDNA enhanced HIV-1 multiantigen pDNA vaccine delivered intramuscularly with electroporation, with an HIV-1 rVSV vaccine boost, in healthy HIV-uninfected adult participants

Primary objectives

To evaluate the safety and tolerability of a prime-boost regimen of HIV-MAG vaccine given with and without plasmid human *IL-12*, delivered with electroporation, followed by an $rVSV_{IN}$N4CT1*gag*1 (VSV HIV *gag*) boost in healthy HIV-uninfected adult volunteers.

Study products and routes of administration

- **HIV-1 multiantigen pDNA (HIV-MAG) vaccine**: The HIV-MAG vaccine consists of 2 plasmid DNA expression vectors, HIV-1 *gag*/*pol* and HIV-1 *nef*/*tat*/*vif*, *env*. HIV-1 *gag*/*pol* expresses an HIV-1 clade B Gag-Pol fusion under the control of a human cytomegalovirus (hCMV) promoter and bovine growth hormone (BGH) polyadenylation signal. HIV-1 *nef*/*tat*/*vif*, *env* expresses (i) an HIV-1 clade B Nef-Tat-Vif fusion under the control of an hCMV promoter and an SV40 polyadenylation signal; and (ii) an HIV-1 clade B primary isolate Env gp160 under the control of a simian cytomegalovirus (sCMV) promoter and BGH polyadenylation signal

- ***IL-12* plasmid (*IL-12* pDNA) adjuvant**: The HIV-MAG vaccine will be administered with a 3rd expression plasmid encoding the p35 and p40 subunits of human *IL-12*

- **Electroporation (EP) device**: The HIV-MAG vaccine, *IL-12* pDNA adjuvant, and placebo will be delivered by intramuscular (IM) injection followed by EP using the Ichor Medical Systems TriGrid™ Delivery System (TDS) EP device. Each dose will be delivered as 2 injections, 1 into each deltoid.

- **$rVSV_{IN}$N4CT1*gag*1 (VSV HIV *gag*) vaccine**: The VSV HIV *gag* vaccine candidate is an attenuated recombinant Indiana serotype vesicular stomatis virus ($rVSV_{IN}$) vector containing the HIV-1 Gag gene. The dose to be given will be the maximum tolerated dose (MTD) from a separate VSV phase 1 trial, HVTN 090, delivered as 2 x 1 mL injections by needle and syringe IM, 1 mL into each deltoid.

- **Placebo for HIV-MAG vaccine, *IL-12* pDNA adjuvant, and VSV HIV *gag* vaccine**: Sodium Chloride for Injection, USP 0.9%

Table 3-1 Schema

Study arm	N	Dose *IL-12* pDNA	Month 0	Month 1	Month 3	Month 6
Group 1	22	0 mcg	HIV-MAG*	HIV-MAG	HIV-MAG	VSV HIV *gag***
	3	—	placebo	placebo	placebo	placebo
Group 2	22	250 mcg	HIV-MAG + *IL-12* pDNA	HIV-MAG + *IL-12* pDNA	HIV-MAG + *IL-12* pDNA	VSV HIV *gag*
	3	—	placebo	placebo	placebo	placebo
Group 3	22	1000 mcg	HIV-MAG + *IL-12* pDNA	HIV-MAG + *IL-12* pDNA	HIV-MAG + *IL-12* pDNA	VSV HIV *gag*
	3	—	placebo	placebo	placebo	placebo
Group 4	22	1500 mcg	HIV-MAG + *IL-12* pDNA	HIV-MAG + *IL-12* pDNA	HIV-MAG + *IL-12* pDNA	VSV HIV *gag*
	3	—	placebo	placebo	placebo	placebo
**Total** 100 (88 vaccine / 12 placebo)						

*Dose of HIV-MAG is 3 mg throughout the trial. When given with adjuvant, HIV-MAG and IL-12 pDNA are admixed into the same syringe.

**The dose of VSV HIV *gag* throughout the trial is 3.4×10^7 plaque forming units (PFU) (nominal dose = 1×10^8 PFU) or lower MTD from HVTN 090. A nominal dose of 1×10^8 PFU based on the vaccine label provides an actual dose of 3.4×10^7 PFU per the release certificates of analysis.

Notes

All injections of HIV-MAG, *IL-12* pDNA, placebo for HIV-MAG, and placebo for *IL-12* are given with EP using the Ichor Medical Systems TDS EP device.

Groups 1, 2, and 3 will be opened to enrollment simultaneously. Groups 1 and 3 share the same visit schedule, with more frequent visits for evaluation of innate immune responses to the vaccines and will be randomized together. Group 2 will be randomized separately. Enrollment across all participating HVTN clinical research sites (CRSs) will be restricted to a maximum of 1 participant per day until 15 participants have been enrolled overall, in any group(s). Enrollment will then be held until the PSRT reviews available safety, tolerability, and acceptability data through day 14 for those participants. If these data are acceptable, enrollment may then proceed for Groups 1-3. See Section 11.3.1

To determine whether enrollment can proceed for Group 4, the PSRT will review all cumulative safety and tolerability data available from at least 24 participants enrolled in Groups 1 and 3 (12 in each group), and all 25 participants in Group 2, through day 14. If these data are acceptable, enrollment may then proceed for Group 4, with a maximum of 1 Group 4 participant per day across all participating HVTN CRSs until 5 participants have been enrolled. Enrollment for Group 4 will then be held until the PSRT reviews available safety, tolerability, and acceptability data through day 14 for those participants. If these data are acceptable, enrollment may then proceed for Group 4.

Participants

100 healthy, HIV-uninfected volunteers at low risk for HIV infection, aged 18 to 50 years; 88 vaccinees, 12 placebo recipients

Design

Multicenter, randomized, placebo-controlled, double-blind trial

Duration per participant

15 months per participant

In addition, participants will be contacted annually for purposes of extended safety surveillance for a total of 3 years following initial study injection.

Estimated total study duration

50 months (includes enrollment, planned safety holds, follow-up; and annual health contacts)

Investigational New Drug (IND) sponsor

DAIDS, NIAID, NIH, DHHS (Bethesda, Maryland, USA)

Study product providers

Vaccine provider

- HIV-MAG vaccine: Profectus Biosciences, Inc. (Tarrytown, New York, USA)
- *IL-12* pDNA adjuvant: Profectus Biosciences, Inc (Tarrytown, New York, USA)
- VSV HIV *gag* vaccine: Profectus Biosciences, Inc (Tarrytown, New York, USA)

Device provider

- TDS EP device: Ichor Medical Systems, Inc. (San Diego, California, USA)

Core operations

HVTN Vaccine Leadership Group/Core Operations Center, Fred Hutchinson Cancer Research Center (FHCRC) (Seattle, Washington, USA)

Statistical and data management center (SDMC)

Statistical Center for HIV/AIDS Research and Prevention (SCHARP), FHCRC (Seattle, Washington, USA)

HIV diagnostic laboratory

University of Washington Virology Specialty Laboratory (UW-VSL) (Seattle, Washington, USA)

Other laboratories

- Duke University Medical Center (Durham, North Carolina, USA)
- FHCRC/University of Washington (Seattle, Washington, USA)
- Profectus Biosciences, Inc (Tarrytown, New York, USA)

Study sites

HVTN Clinical Research Sites (HVTN CRSs) to be specified in the Site Announcement Memo

Safety monitoring

HVTN 087 PSRT; HVTN Safety Monitoring Board (SMB)

3.1 Protocol Team

Protocol leadership

Chair	Christine (Mhorag) Hay<br>University of Rochester<br>585-275-6962<br>christine_hay@urmc.rochester.edu	*Statistician*	Shuying (Sue) Li<br>SCHARP, FHCRC<br>206-667-2437<br>sli@fhcrc.org
Cochair	Greg Wilson<br>Vanderbilt University<br>615-343-5731<br>greg.wilson@vanderbilt.edu	*Medical officer*	Mary Allen<br>DAIDS, NIAID<br>301-402-2310<br>mallen@niaid.nih.gov
Protocol Team leader	Marnie Elizaga<br>HVTN Core, FHCRC<br>650-854-4984<br>melizaga@hvtn.org		

Other contributors to the original protocol

Core medical monitor	Marnie Elizaga<br>HVTN Core, FHCRC	*Clinical affairs*	Maija Anderson<br>SCHARP, FHCRC
Vaccine developer representative	John Eldridge<br>Profectus Biosciences, Inc.	*Clinical trials manager*	Carissa Karg<br>HVTN Core, FHCRC
Vaccine developer representative	Michael A. Egan<br>Profectus Biosciences, Inc.	*Project manager*	Diana Lynn<br>SCHARP, FHCRC
Vaccine developer representative	David Clarke<br>Profectus Biosciences, Inc.	*Senior project manager*	Drienna Holman<br>SCHARP, FHCRC
Device developer representative	Drew Hannaman<br>Ichor Medical Systems, Inc.	*Protocol development coordinator*	Julie Foster<br>HVTN Core, FHCRC
Laboratory Program representative	John Hural<br>HVTN Laboratory Program, FHCRC	*Community education unit representative*	Gail Broder<br>HVTN Core, FHCRC
Laboratory Program representative	Jin Bae<br>HVTN Laboratory Program, FHCRC	*Community educator/recruiter*	Casey Braddy<br>Vanderbilt University
Regulatory affairs	Liz Briesemeister<br>HVTN Core, FHCRC	*Community Advisory Board (CAB) member*	Richard Fowler<br>University of Rochester CAB
DAIDS Project Officer	Michael Pensiero<br>DAIDS, NIAID	*CAB member*	Frank Herrington<br>Vanderbilt University CAB
DAIDS protocol pharmacist	Scharla Estep<br>DAIDS, NIAID<br>301-435-3746	*Clinic coordinator*	Cathy Bunce<br>University of Rochester

4 Background

4.1 Rationale for trial concept

A strategy to elicit HIV-specific CD8+ cytotoxic T-lymphocyte (CTL) responses through vaccination remains an important goal in preventing HIV disease. Of the various approaches being developed as prophylactic HIV vaccines, those based on a heterologous plasmid DNA prime/live vector boost vaccination regimen appear especially promising in pre-clinical animal models [4,5]. The trial will evaluate the safety and immunogenicity of a cytokine enhanced HIV-1 multiantigen plasmid DNA (HIV-MAG) vaccine produced by Profectus BioSciences, Inc. when given with or without plasmid *IL-12* (*IL-12* pDNA) followed by a VSV HIV *gag* boost. The prime-boost vaccine regimen is intended to elicit robust and long-lived HIV-specific CD4+ and CD8+ T-cell responses believed to be critical in the control of virus replication [6-9].

This phase 1, randomized, placebo-controlled study is designed to evaluate the safety and immunogenicity of a novel HIV-MAG vaccine with increasing doses of *IL-12* pDNA delivered with EP in healthy HIV-uninfected adult volunteers. EP has been shown to be an efficient way to introduce DNA into cells [10,11]. EP is a technology in which a transient electric field is applied to enhance the cellular uptake of large molecules such as DNA and vaccines. EP works by temporarily increasing the permeability of cell membranes. This technology has been used for more than 3 decades by molecular biologists for cell transfection. More recently, clinical applications of EP have been tested, especially in cancer treatment and gene therapy [12-14]. Adding EP to the IM injection procedure has resulted in improved immunogenicity of DNA vaccines [15,16]. In a recent phase 1 study, HVTN 080, EP was used with the DNA vaccine, PENNVAX-B (PV-B) given IM with or without co-administration of *IL-12* pDNA. EP demonstrated a significant dose sparing effect, as doses were reduced compared to another trial with PV-B, HVTN 070 (see Section 4.10.2). Electroporation technology has the potential to change the prospects for DNA vaccines.

Although *IL-12* pDNA has been shown to enhance immune responses in preclinical vaccine studies [17-19], early studies with DNA vaccines given by IM injection in humans were disappointing. In HVTN 080 where *IL-12* was administered with EP however, a significant adjuvant effect of *IL-12* was shown. Two weeks after the 3rd vaccination, the overall response rate for T cells for recipients of PV-B with *IL-12* pDNA was 89%, compared to 66.7% for PV-B alone. Adjuvant effects may be dependent on the dose of *IL-12* pDNA administered. In preclinical studies, the adjuvant activity of *IL-12* pDNA is highly dose dependent with the highest doses of *IL-12* actually decreasing immunogenicity of the co-administered pDNA vaccine when compared to lower doses. In this trial several different dose levels of *IL-12* will be tested along with EP to attempt to further refine p*IL-12* dosing.

Prime-boost strategies are routinely used in vaccination regimens to increase the magnitude of the immune response. Heterologous prime-boost strategies are especially promising and are being studied as vaccine strategies for a number of infections including HIV, malaria, and tuberculosis. This trial will utilize an rVSV HIV *gag* vector as a boost for the HIV-MAG pDNA prime. A separate HVTN phase 1 dose escalation trial, HVTN 090, which is evaluating the safety and immunogenicity of the VSV HIV *gag* vaccine

delivered by standard IM injection, will start by Q4 2011. That study will provide safety data to support the dose of VSV HIV *gag* vaccine that will be given to participants as the boost of this study. There are many aspects of VSV biology that support the development of VSV vaccine vectors for the treatment and prevention of human disease. The virus genome is organized into discreet transcriptional units (TUs) for expression of viral proteins, and nucleotide signal sequences involved in the control of viral gene expression have been well defined. Accordingly, one or more additional TUs encoding foreign proteins can be added to the VSV genome and robustly expressed under control of the 3′ transcription promoter [20,21]. Furthermore, the abundance of foreign protein expression can be modulated depending on proximity of the extra TUs relative to the 3′ transcription promoter. The ability of VSV vaccine vectors to propagate vigorously in qualified continuous cell lines also facilitates industrial scale manufacture of vaccine preparations [22]. Importantly, because human infection is rare, VSV seroprevalence in the general population is very low; therefore, immunization with VSV vaccine is unlikely to activate and expand VSV-specific CD4+ T-cells that may lead to an increased risk of HIV infection. In addition, preexisting immunity will not likely be a factor affecting the efficacy of future VSV vaccine vectors. Indeed, the existence of many different natural (or wild-type) VSV serotypes may be exploited to design multiple VSV vaccine vectors for prevention and treatment of many different diseases. VSV vaccines do not cause any obvious signs of disease after parenteral administration to either livestock or nonhuman primates (NHP), and VSV vectors are anticipated to be innocuous in humans when administered parenterally [23-25].

This trial will answer a number of important questions regarding the safety and efficacy of *IL-12* enhancement of pDNA vaccines given by EP and the breadth of the immune response that can be achieved with HIV-MAG vaccine. The study is designed to determine the optimal dose of *IL-12* pDNA as a cytokine adjuvant to use with EP. In addition, the study will evaluate the effects of the HIV-MAG priming regimen and *IL-12* pDNA doses given with EP on immune responses to a prototype VSV HIV *gag* vaccine, to determine whether these differences in priming lead to detectable differences in immune responses following the boost.

4.2 HIV-MAG vaccine

The HIV-MAG vaccine consists of 2 plasmid DNA expression vectors, HIV-1 *gag*/*pol* and HIV-1 *nef*/*tat*/*vif*, *env*. HIV-1 *gag*/*pol* is a 7,487 base pair plasmid expressing an HIV-1 clade B Gag/Pol fusion under the control of an hCMV promoter and BGH polyadenylation signal. HIV-1 *nef*/*tat*/*vif*, *env* is an 8,750 base pair dual promoter plasmid expressing (i) an HIV-1 clade B Nef/Tat/Vif fusion under the control of an hCMV promoter and an SV40 (simian virus 40) polyadenylation signal; and (ii) an HIV-1 clade B primary isolate Env gp160 under the control of an sCMV promoter and BGH polyadenylation signal. The plasmids are formulated in 0.25% bupivacaine-HCl to enhance plasmid stability and entrance into cells. The HIV-1 *gag*/*pol* and HIV-1 *nef*/*tat*/*vif*, *env* were manufactured by Boehringer Ingelheim, Vienna, Austria and vialed by Cardinal Health, Albuquerque, NM. The HIV-MAG vaccine will be administered with a 3rd expression plasmid encoding the p35 and p40 subunits of human IL-12. The HIV-MAG vaccine will be delivered by two IM injections (one in each deltoid) followed by EP using the Ichor TDS. The HIV-MAG vaccine has been shown to be immunogenic in mice [7] and nonhuman primates [6] eliciting potent, balanced cell-mediated immune responses to multiple HIV-1 derived antigens.

4.3 *IL-12* pDNA adjuvant

The *IL-12* pDNA adjuvant, GENEVAX® *IL-12*, has previously been tested by the HVTN in 4 earlier trials (see Section 4.10.2): HVTN 060 (DAIDS-ES ID 10057, BB IND# 12367), HVTN 063 (DAIDS-ES ID 10058, BB IND# 12439), HVTN 070 (DAIDS-ES ID 10490, BB IND# 13449), and HVTN 080 (DAIDS-ES ID 10741, BB IND# 14116). The specific plasmid for use in this trial is GENEVAX® *IL-12*-4532. The *IL-12* pDNA is a dual promoter expression plasmid which expresses the genes encoding human IL-12 proteins p35 and p40 under separate regulatory control. The p35 subunit is under the control of the hCMV promoter/enhancer and the SV40 polyadenylation signal. The p40 subunit is under the control of the sCMV promoter and the BGH polyadenylation signal. The plasmid contains a chimeric kanamycin resistance gene and a pUC bacterial origin of replication. The plasmid adjuvant is formulated in 30 mM citrate buffer pH 6.5 containing 150 mM NaCl, 0.01% ethylenediamine tetraacetic acid (EDTA), and 0.25% bupivacaine-HCl. It was manufactured by DSM-Biologics, Groningen, The Netherlands.

Adjuvant effects may be dependent on the dose of *IL-12* pDNA administered. In preclinical studies, the adjuvant activity of *IL-12* pDNA is highly dose dependent with the highest doses of *IL-12* actually decreasing immunogenicity of the co-administered pDNA vaccine when compared to lower doses (Figure 4-1).

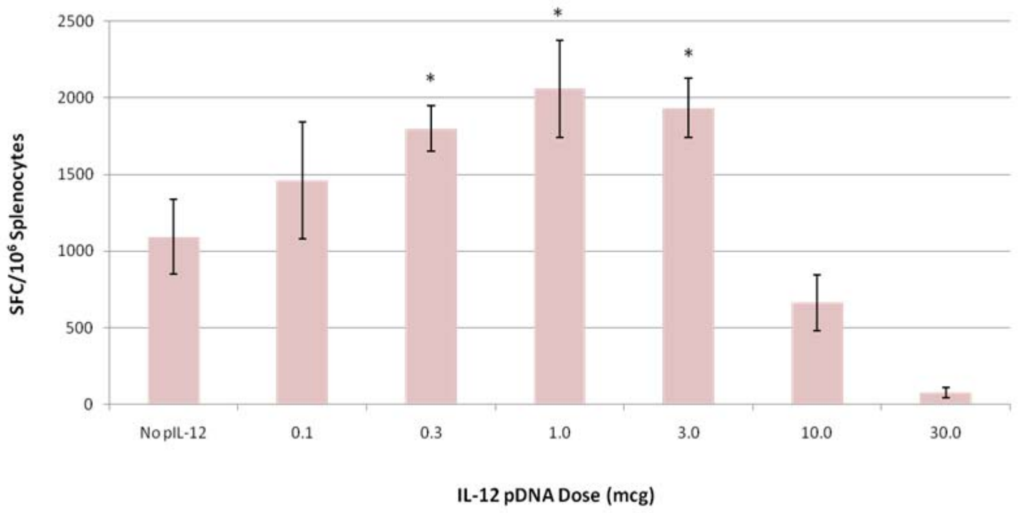

Figure 4-1 Profectus/Ichor (unpublished data): Balb/c mice were immunized IM/EP with 25mcg of a prototype pDNA vaccine encoding HIV Gag p55 in combination with increasing amounts (0, 0.1, 0.3, 1.0, 3.0, 10 and 30 mcg) of murine *IL-12* pDNA. Mice (n=5/group) were vaccinated at day 0 and spleens were harvested at 14 days after a single immunization and screened for the presence of HIV-1 Gag p55 peptide pool-specific interferon gamma (IFN-γ) enzyme-linked immunospot (ELISpot) responses. *: Represents statistically significant difference relative to no p*IL-12*. These results show that the use of higher doses of *IL-12* pDNA DECREASES the immunogenicity of a prototype pDNA vaccine

The dose dependence of adjuvant *IL-12* pDNA has been shown in a number of other preclinical model systems utilizing various different vaccine candidates [26,27]. A study

of an *IL-12* enhanced cytomegalovirus vaccine in humans showed a modest dose effect of rh*IL-12* [28]. As previously mentioned, this trend was also noted in HVTN 060, in which most participants with positive IFN-γ ELISpot responses to consensus clade B Gag peptide pools were from an intermediate dose group in the dose escalation phase of the trial. Gag-specific responses were detected in 4/9 (44.5%) participants who received 3 IM injections of Gag DNA vaccine with 500 mcg *IL-12* pDNA, compared to 2/33 (6%) participants who received no adjuvant; 0/10 participants who received 100 mcg *IL-12* pDNA, and 0/49 participants who received 1500 mcg *IL-12* pDNA.

4.4 VSV HIV *gag*

The gene order of wild-type VSV is 3'N-P-M-G-L 5', where N is the gene encoding the nucleocapsid protein (position 1), P is the gene encoding the phosphoprotein, M is the gene encoding the matrix protein, G is the gene encoding the surface glycoprotein, and L is the gene encoding the RNA-dependent RNA polymerase (position 5). The $rVSV_{IN}$N4CT1Gag1 (VSV HIV *gag*) vaccine candidate is a recombinant Indiana serotype VSV ($rVSV_{IN}$) vector containing the HIV-1 *gag* gene in the first position of the genome. The rVSV vector was attenuated by combination of two major genome modifications, which act synergistically to enhance virus attenuation. The attenuating modifications include the translocation of the N gene to the third position (N3) in the genome and truncation of the G protein cytoplasmic tail (CT) from 29 amino acids to only 1 amino acid (CT1). The HIV-1 *gag* gene was then inserted at position 1 of the genome, which shifted the N gene to position 4 (N4) in the genome, contributing to the further attenuation of this vector. The resulting gene order of this highly attenuated vector is 3'Gag-P-M-N-G/CT1-L 5' (rVSVN4CT1Gag1).

The rVSV vector was manufactured by Henogen, Charleroi, Belgium.

The dose to be given will be determined from the HVTN phase 1 trial of VSV HIV *gag* vaccine, HVTN 090. The dose will be delivered as two 1 mL injections by needle and syringe IM, 1 mL into each deltoid. The VSV HIV *gag* vaccine trial is anticipated to start by Q4 2011. Available safety data from HVTN 090 will be reviewed prior to initiation of boost injections in this study (Table 4-1).

Table 4-1 Schema for HVTN 090 phase 1 dose escalation of VSV HIV *gag*

				Vaccination schedule in months (days)	
**Dosage group**	**N**	**Nominal Dose**	**Actual Dose per CoA**	**0 (0)**	**2 (56)**
Group 1	10	1 x 10^4 PFU	4.6 x 10^3 PFU	VSV_{IN} HIV *gag* vaccine	VSV_{IN} HIV *gag* vaccine
	2	—	—	placebo	placebo
Group 2	10	1 x 10^5 PFU	4.6 x 10^4 PFU	VSV_{IN} HIV *gag* vaccine	VSV_{IN} HIV *gag* vaccine
	2	—	—	placebo	placebo
Group 3	10	1 x 10^6 PFU	4.8 x 10^5 PFU	VSV_{IN} HIV *gag* vaccine	VSV_{IN} HIV *gag* vaccine
	2	—	—	placebo	placebo
Group 4	10	1 x 10^7 PFU	4.2 x 10^6 PFU	VSV_{IN} HIV *gag* vaccine	VSV_{IN} HIV *gag* vaccine
	2	—	—	placebo	placebo
Group 5	10	1 x 10^8 PFU	3.4 x 10^7 PFU	VSV_{IN} HIV *gag* vaccine	VSV_{IN} HIV *gag* vaccine
	2	—	—	placebo	placebo
Total	60 (50 vaccine/10 placebo)				

VSV infection in humans is rare, but it can occur when animal handlers and veterinarians come in close contact with infected livestock and through inadvertent exposure of laboratory personnel. The most common portals of infection in humans are skin and mucous membranes of the nose, mouth, and eyes, although there is also serological evidence that suggests that some vesiculoviruses may be directly transmitted to humans through insect bites. Human infection with VSV can either be asymptomatic or may lead to disease symptoms, which include myalgia, headache, and fever that resolve in 3–5 days without complications. Because the vaccine candidate is a live, replication competent vector, there is a theoretical risk of human disease including neurologic manifestations. Refer to the Investigator's Brochure (IB) and Section 4.8.5 for more information on neurovirulence testing.

4.5 Electroporation

Electroporation has been shown to be an efficient means to introduce DNA into cells [10,11]. EP is a technology in which a transient electric field is applied to enhance the cellular uptake of large molecules such as DNA. EP works by temporarily increasing the permeability of cell membranes. Although EP has been used experimentally in humans [12,14], EP remains investigational. Preclinical studies have suggested that EP enhances the potency of pDNA vaccines [6,29]. Ichor Medical Systems has focused on the development of EP technology where the means for agent administration and electric field application are integrated into a fully automated administration device. The overarching goal of this approach, embodied in the Ichor TDS, is to enable the site of agent administration, placement of electrodes, and timing of EP delivery to be completed in a simple, user independent fashion. Each TDS device consists of three components, a single use Application Cartridge housing the EP electrodes and the agent of interest, a handheld, multi-use Integrated Applicator, and a Pulse Stimulator. Correlation of the electrode configuration with the agent distribution pattern intrinsic to the target tissue ensures that the EP effect is induced only in tissues where the agent of interest has been distributed improving tolerability and minimizing tissue disruption. To date, the TDS has been used for DNA delivery in more than 70 clinical trial subjects at pDNA doses of up

to 4.0 mg. No serious pDNA or device related adverse events have been reported and all of the subjects have complied with administration schedules, reaffirming the safety and tolerability of this delivery approach.

4.6 Trial design rationale

The primary objective of the trial is to evaluate the safety and tolerability of the HIV-1 multiantigen pDNA vaccine delivered with EP and given with and without increasing doses of plasmid human *IL-12*, in a prime-boost combination with a candidate HIV-1 rVSV vaccine. This phase 1 trial will enroll up to 100 participants of whom 88 will receive the study vaccines and 12 will receive placebo.

An earlier study, HVTN 080, has provided important initial safety and immunogenicity data on the use of EP with a HIV-1 pDNA vaccine and *IL-12* pDNA. This study, HVTN 087, will evaluate a different pDNA vaccine, HIV-MAG, with two additional dose levels of *IL-12* pDNA which were not tested in HVTN 080, and a different EP device from HVTN 080. This study will also evaluate the electroporated DNA vaccine regimen with a VSV HIV *gag* vaccine boost.

In addition to standard assays for cellular and humoral immunity (described in Sections 10.4 and 10.5), the study will include assays to characterize the innate immune response to the HIV MAG and VSV HIV vaccines, in Groups 1 and 3. The HVTN seeks to develop assays that will create a profile of the innate immune response to vaccines, and to use these assays to correlate innate responses with adaptive immune responses to determine which innate parameters are predictive of good quality memory T and B cell responses. A greater understanding of the innate immune responses to vaccines may yield critical information for the development of better vaccine vectors and adjuvants.

4.6.1 Dose rationale

HIV-MAG vaccine will be administered to all vaccinees at a dose of 3000 mcg which is an amount well below previously utilized doses of up to 8000 mcg of DNA in another HIV vaccine [30]. This dose is within the range of both nonhuman primate and human DNA vaccine studies including a study of an HIV DNA vaccine delivered with the TDS EP device, see Section 4.10.3. The *IL-12* pDNA component will be delivered at 0, 250, 1000, and 1500mcg. The 0 mcg *IL-12* pDNA dose, HIV-MAG alone with EP, will provide a comparison group to determine if *IL-12* pDNA contributes to increased immunogenicity over this group. 1000 mcg *IL-12* pDNA was the dose tested in HVTN 080 with PV-B given with EP, with evident adjuvant effect. To allow comparison to the HVTN 080 vaccine regimen, that dose will also be included in this trial. Doses of 250 and 1500 mcg are chosen to test both higher and lower doses than previously tested. See Section 4.10.2.

The trial evaluates 3 vaccinations of the HIV-MAG vaccine as a priming regimen followed by a single vaccination of VSV HIV *gag*. The priming effects of the HIV-MAG vaccine with *IL-12* pDNA at different doses may not be fully appreciated until after the VSV HIV *gag* boost vaccination. The VSV HIV *gag* boost will provide data for the dose selection of *IL-12* pDNA vaccine, as well as first-in-humans safety and immunogenicity information for the HIV-MAG-VSV HIV *gag* prime-boost regimen overall.

The total volume of the HIV-MAG vaccine and the *IL-12* plasmid at the highest dose will be slightly less than 2 mL. The vaccine or placebo will therefore be administered by two IM injections with EP into the deltoid (one in each arm). The VSV HIV *gag* vaccine or placebo will also be given as IM injections (no EP) into both deltoids as the dose for delivery is 2 mL.

4.6.2 Schedule

A total of 3 vaccinations of HIV-MAG vaccine with or without *IL-12* pDNA, or placebo will be given with EP at 0, 1, and 3 months, and then a single IM vaccination of VSV HIV *gag* vaccine or placebo will be given at 6 months without EP. Participants will be followed for a total study period of 15 months per subject. Annual health contacts continue for a total of 3 years following initial study injection.

4.6.3 Choice of control

Sodium Chloride for Injection USP, 0.9% will serve as the placebo for the HIV-MAG vaccine, *IL-12* pDNA, and rVSV vaccine. Sodium Chloride for Injection, USP 0.9% is nonreactogenic and well tolerated.

4.7 Plans for future product development and testing

Using the lessons learned from these two clinical studies, a third clinical study may be undertaken to evaluate the safety, tolerability and immunogenicity of an *IL-12* pDNA enhanced HIV-MAG, rVSV HIV heterologous prime-boost regimen in additional participants. That study may include a second rVSV vector boost with the existing vaccine, a heterologous serotype rVSV vaccine, or newer generation rVSV vectors encoding for additional HIV antigens. Comparison to other DNA delivery systems, including Biojector, may also be investigated.

4.8 Preclinical safety studies

Table 4-2 Summary of preclinical safety studies

Study number	Product	Type of Study	Animal	N	Dose group	Route	Schedule
Covance Study No. 6617-279	pDNA HIV-*gag*/*pol*<br>pDNA HIV-1 *nef*/*tat*/*vif*, *env*<br>pDNA hIL-12	Repeat dose toxicity	NZW rabbits	10 m<br>10 f	-saline control<br>-vehicle control<br>-HIV-MAG + *IL-12* pDNA	IM	5 cycles (1 dose / 3wks)
Ichor Study No. IMS2008-01	pDNA HIV-*gag*/*pol*<br>pDNA HIV-1 *nef*/*tat*/*vif*, *env*<br>pDNA hIL-12	Biodistribution / Persistence	Wistar outbred rats	11 m<br>11 f	-saline control<br>-HIV-MAG + *IL-12* pDNA (IM/EP)<br>-HIV-MAG + *IL-12* pDNA (IM)	IM,<br>IM/EP	Single dose, analysis at day 7, 60
Charles River Study 503130	pDNA HIV-*gag*/*pol*<br>pDNA HIV-1 *nef*/*tat*/*vif*, *env*<br>pDNA hIL-12	Repeat dose toxicity	NZW rabbits	10 m<br>10 f	-saline control<br>-HIV-MAG<br>-HIV-MAG + *IL-12* pDNA<br>-HIV-MAG + *IL-12* pDNA prime VSV HIV *gag* boost	IM/EP	6 cycle (1dose / 3 wks)
Charles River Study 503415	VSV HIV *gag*	10x dose toxicity	NZW rabbits	10 m<br>10 f	-Matrix<br>-VSV HIV *gag*	IM	Days 1, 29
Sierra Study ADO0003	rVSV$_{IN}$N4CT1*gag*1	Neurovirulence toxicity	Cynomolgus monkeys	6/group	-VSV IN wt (positive control)<br>-inactivated *gag*1N4CT1 (negative control)<br>-VSV HIV *gag*	IT	Single dose
Wyeth Study RPT-64154	rVSV$_{IN}$N4CT9*gag*1[a]	Virus biodistribution	Balb/c mice	35<br>50<br>50	-rVSV*gag*5<br>-VSV HIV*gag*	IV<br>IM<br>IN	Day 1

m = male
f = female
NZW = New Zealand White
VSV IN wt = a laboratory adapted wild type strain of VSV
wks = weeks
IT = intrathalmic
IV = intravenous
[a] rVSV$_{IN}$N4CT9*gag*1 is a closely related but more replication competent vector than VSV HIV *gag*.

4.8.1 Repeat dose toxicity of Profectus' HIV multiantigen pDNA vaccine (HIV-MAG) in combination with human *IL-12* pDNA by standard IM injection "Covance Study No. 6617-279"

This study was designed to assess the nonclinical toxicity of 5 IM dosages of 2 HIV multiantigen plasmid constructs (pDNAs) in combination with human *IL-12* pDNA, administered 3 weeks apart and to assess the reversibility, persistence, or delayed occurrence of any effects after a 30-day recovery in male and female New Zealand white rabbits (strain Hra:[NZW]SPF). The two HIV constructs consist of one expressing a Gag/Pol fusion protein (HIV-1 *gag*/*pol*), the other expressing a Nef/Tat/Vif fusion protein and Env protein (HIV-1 *nef*/*tat*/*vif*, *env*), combined with *ori*-altered h*IL-12* pDNA (GENEVAX® *IL-12*) as adjuvant.

HIV-MAG, vehicle-control, or saline-control were administered to 10 male and 10 female rabbits as an IM injection into the vastus lateralis muscle of the left and right leg (1.6 mL per leg or 3.2 mL total injection per dosing day) on days 1, 22, 43, 64, and 85 of the dosing phase followed by a 2- or 30-day observation period. Each HIV-MAG dose consisted of 3000 mcg of HIV-1 *gag*/*pol*, 3000 mcg of HIV-1 *nef*/*tat*/*vif*, *env* and 1500 mcg of *IL-12* pDNA administered as a total pDNA content of 2.38 mg/mL. The vehicle-control consisted of 0.25% bupivacaine HCl in 30 mM citrate buffer, 0.15M NaCl and 0.01% EDTA at pH 6.5. The saline control consisted of 0.9% Sodium Chloride for injection, USP. Evaluations for HIV-MAG-related effects were based on mortality, clinical observations, injection site irritation, body temperature, body weight, food consumption, ophthalmology, hematology, coagulation, clinical chemistry, organ weights, and macroscopic and microscopic examinations. Antibody evaluation of anti-HIV-MAG was also conducted.

There were no HIV-MAG related deaths during the study. All rabbits survived to the scheduled euthanasia, except 1 vehicle control male and 1 vehicle control female electively euthanized on days 67 and 22 of the dosing phase, respectively, due to a moribund condition related to spontaneous renal disease (male) or injection trauma (female).

In the rabbits that survived to scheduled termination, there were no HIV-MAG-related clinical signs or injection site irritation, or effects on body weight, food consumption, body temperature, ophthalmology, hematology, urinalysis, and macroscopic observations. There were no HIV-MAG-associated microscopic alterations. There were no differences between the saline control group and vehicle control group during the study.

For clinical chemistry, the only differences considered HIV-MAG-related occurred 2 days postdose in males and/or females compared to the vehicle controls (ie, days 3 and/or 87 of the dosing phase). These effects included mildly increased fibrinogen (24% to 34%), minimally increased globulin (15%), and minimally decreased albumin-to-globulin ratio (15%). These findings were consistent with a mild, transient inflammatory reaction to the test article injections. Therefore, they were not considered adverse based on the magnitude of change, and transitory occurrence.

For organ weights, the only differences considered HIV-MAG-related occurred in males and females at the end of the dosing phase compared to the vehicle controls. Absolute and relative (to body and brain) splenic weights were increased 54% to 76% in males and 23% to 36% in females. Due to the absence of hematologic, macroscopic and microscopic correlates and the lack of splenic weight change 30 days after the last dose, the splenic weight increase was not considered adverse.

Antibodies to HIV-MAG were not detected.

In conclusion, 5 IM dosages of the HIV-MAG pDNAs, expressing a Gag/Pol fusion protein, a Nef/Tat/Vif fusion protein and Env protein, and human *IL-12* pDNA, mixed at 3000 mcg, 3000 mcg, and 1500 mcg, respectively, administered 3 weeks apart in male and female Hra:(NZW) SPF New Zealand white rabbits at a dose volume of 3.2 mL per dose as a 2.38 mg/mL solution was well tolerated and did not cause any adverse effects.

4.8.2 Biodistribution, persistence and potential for integration of Profectus' HIV-1 multiantigen pDNA vaccine (HIV-MAG) in combination with human *IL-12* pDNA by

standard IM injection followed by in vivo EP "Ichor study No. IMS2008-01"

A good laboratory practice (GLP) study was initiated to characterize the systemic biodistribution and persistence/integration of Profectus Biosciences' HIV-1 multiantigen pDNA vaccine candidate (HIV-1 *gag*/*pol*; HIV-1 *nef*/*tat*/*vif*, *env,*) in combination with human *IL-12* pDNA when administered IM in rats either by conventional injection or EP-mediated delivery using Ichor's TDS. For this purpose 80 mcg of formulated vaccine pDNA (32 mcg HIV-1 *gag*/*pol*, 32 mcg HIV-1 *nef*/*tat*/*vif*, *env* , 16mcg GENEVAX® *IL-12*) was administered bilaterally to the tibialis anterior muscles of two groups of 22 rats each (11 rats/sex/group) per TDS or by conventional IM injection (total dose of 160 mcg per subject). A group of 12 rats (6 rats/sex) was injected with saline using the TDS device. Real-time quantitative polymerase chain reaction (qPCR) was used to estimate the levels of vaccine pDNA in tissue specimens. The presence of vaccine pDNA in total DNA (genomic + plasmid) isolated from blood, bone marrow, heart, lungs, liver, spleen, kidney, brain, testis/ovaries, draining lymph nodes from the injection site, muscles in which pDNA was administered (bilateral tibialis anterior), and overlying skin from the injected muscle was examined in this study (IMS2008-01) at Day 7 for cohorts of 10 rats from each group that received the vaccine pDNA and 6 rats from the group that received saline. Tissues from groups administered vaccine pDNA that tested positive for plasmid on Day 7 were evaluated for plasmid presence/integration 60 days after injection (12 rats per group).

Polymerase chain reaction (PCR) analyses of tissue specimens obtained at Day 7 showed that, for both TDS–mediated administration and conventional IM injection, distribution of vaccine pDNA was largely confined to the tissues at the site of administration (muscle and skin). Plasmid was detected in only 2 tissue types outside of the local injection site, all at very low levels (<150 copies/mcg genomic DNA). In the TDS delivery group, vaccine pDNA was detected in draining lymph nodes in 7 out of 10 subjects. In the conventional injection group, vaccine pDNA was detected in 4 lymph node specimens and 1 kidney specimen out of 10 subjects.

At the Day 60 timepoint, vaccine pDNA was detected in the tissues from the site of administration in both the TDS and conventional injection groups at levels of less than 7,000 copies/mcg. This is below the level recommended for further analysis in current guidance for plasmid vaccines for infectious disease indications [31]. Plasmid levels had decreased by approximately 1 – 2 orders of magnitude compared to the levels detected at the Day 7 timepoint. Plasmid was also detected in draining lymph nodes at a low level (<30 copies/mcg genomic DNA) in 1 out of 12 subjects in the TDS group, and no plasmid was detected outside of the local injection site in the conventional injection group.

These analyses indicate that IM delivery of vaccine pDNA resulted in an initial uptake of plasmid localized to the injection site that decreased substantially over time. No significant differences in biodistribution or persistence were observed when vaccine pDNA was administered by TDS–mediated administration or by conventional IM injection.

4.8.3 Repeat dose toxicity of Profectus' HIV-1 multiantigen pDNA vaccine (HIV-MAG) in combination with human *IL-12* pDNA delivered by standard IM injection and by electroporation with or without rVSV gag by injection. "Charles River Study No. 503130"

The objective of this study was to assess the toxicity of up to 6 cycles of IM injections followed with EP (1 cycle = 1 dose every 3 weeks) of HIV-MAG (HIV-1 *gag*/*pol* and HIV-1 *nef*/*tat*/*vif*, *env*) with or without the combination of *IL-12* pDNA (GENEVAX® *IL-12)* in male and female rabbits; where a subpopulation of HIV-MAG and *IL-12* pDNA-treated animals received booster vaccinations with $rVSV_{IN}$N4CT1*gag*1(VSV HIV *gag)* on Days 64 and 85. Reversibility, persistence, or delayed occurrence of any effects was evaluated following a 2 or 30 day recovery period after the last dose. The study design is detailed in Table 4-3.

Table 4-3 Study design for Charles River Study No. 503130

Group Number	Product	Route of Administration	Dose Volume/Site (mL)	Total Dose Volume[g] (mL)	Number of Males Assigned[a]	Number of Females Assigned[a]
1	0.9% Sodium Chloride for Injection USP[b]	IM	0.93	1.86	10	10
2	HIV-1 *gag*/*pol* and HIV-1 *nef*/*tat*/*vif*, *env*[c]	IM/EP	0.67	1.34	10	10
3	HIV-1 *gag*/*pol*, HIV-1 *nef*/*tat*/*vif*, *env*, and *IL-12* pDNA[d]	IM/EP	0.93	1.86	10	10
4	HIV-1 *gag*/*pol*, HIV-1 *nef*/*tat*/*vif*, *env*, and *IL-12* pDNA[e], followed by $rVSV_{IN}$N4CT1*gag*1 (Day 64) and $rVSV_{NJ}$N4CT1*gag*1 (Day 85)[f]	IM/EP<br>IM	0.93<br>0.10	1.86<br>0.20	10	10

IM/EP = intramuscular injection followed by EP

a Groups 1 to 3: Day 108 or 136 euthanasia; 5 animals/sex/group. Group 4: Day 87 or 115 euthanasia; 5 animals/sex/group.

b IM injection with an Autoject Ultem Plastic Support Structure (AUSS) depth adjuster, on Days 1, 22, 43, 64, 85 and 106.

c HIV multiantigen plasmids (4 mg total; 2 mg each individual pDNA) delivered by IM/EP, on Days 1, 22, 43, 64, 85 and 106.

d HIV multiantigen plasmids (5 mg total; 2 mg each individual pDNA plus 1 mg plasmid *IL-12*) delivered by IM/EP, on Days 1, 22, 43, 64, 85 and 106.

e HIV multiantigen plasmids (5 mg total; 2 mg each individual pDNA plus 1mg plasmid *IL-12*) delivered on Days 1, 22 and 43 by IM/EP.

f $rVSV_{IN}$N4CT1*gag*1 (VSV HIV *gag,* targeted $1x10^7$ PFU: actual dose $1.2x10^6$ PFU) delivered on Day 64 by IM injection and $rVSV_{NJ}$N4CT1Gag1 (targeted $1x10^7$ PFU: actual dose $2.4x10^6$ PFU) delivered on Day 85) by IM injection.

g Equally administered to the right and left vastus lateralis.

The following were evaluated: clinical signs (on each dosing occasions and once weekly on all other occasions), body weight (Day -8, at randomization, prior to and 24, 48 hours post each dosing occasions), food consumption (daily from Day -8), evaluation of injection sites (prior to 4, 24, 48 and 72 hours post each dose and prior to necropsy), body temperature (prior to (Days 1, 22, 43 and 64 only), 4 and 24 hours post each dose, on the day following any temperature above 40°C and prior to necropsy), ophthalmology (prior dosing initiation and during the week of necropsy), hematology and clinical biochemistry (prior to dosing initiation, on Days 4 and 66, following the last dose and at the end of the 30 day recovery period), c-reactive protein (prior to dosing initiation, prior to first dosing

with either HIV-MAG or rVSVs and 7 days post dose), IL-12 assessment (prior to dosing initiation, 48 hours, 3, 7, and 10 days post 1st and last dose), anti-test article antibody assessment (prior to dosing initiation, prior to dosing on Days 64 (group 4 only), 85 (all animals) and 106 (groups 1 - 3 only)), macroscopic observations and injection sites collection at necropsy, organ weights and histopathology.

Overall, there were no changes related to the administration of HIV-MAG or VSV HIV *gag* by IM or by the IM/EP process seen at the injection sites during in vivo irritability scoring, ophthalmic examinations and no effects on bone marrow proliferation and maturation.

Reductions in platelets, white blood cell mass (total white blood cells, neutrophils), higher mean platelet volume and fibrinogen levels, as well as decreases in red cell mass were seen on Day 4 (groups 3 and 4). However, these changes were no longer apparent during (or at the end) of the recovery period.

Clinical biochemistry changes consisted of increases in globulin and decreases in albumin/globulin ratio on Day 4 (groups 3 and 4) and creatine kinase enzyme levels that were significantly increased in all groups on Day 4 and remained high on Day 66 and post last dose in groups 2 and 3. The incidence of the increases in creatine kinase suggest a possible role of the EP procedures in these sustained elevations as animals of groups 2 and 3 had more occasions.

Microscopic changes were seen at the intramuscular injection sites (and adjacent muscles) and in the skin overlying the injection sites in Groups 2 and 3 at the end of the dosing and 30 day recovery periods, and in Group 4 at the end of the dosing period. The changes were most pronounced at the injections sites of Group 2 and 3 animals at the end of the dosing period where the lesions were characterized by necrotizing inflammation with presence of hemorrhage. At the end of the recovery period of Groups 2 and 3 animals, the lesions were mainly degenerative with evidence of fibrosis suggesting resolution of the lesions. At the end of the dosing period of Group 4 animals, lesions were less pronounced and characterized by an increased incidence of myofiber degeneration/necrosis and mononuclear cell infiltration. The electroporation procedures may have contributed to the development of the lesions at the intramuscular injection sites.

Peak human IL-12 levels were detected 48 hours post dose and on Days 4 and 8 for animals administered *IL-12* pDNA (ie, groups 3 and 4 animals). The overall human IL-12 levels ranged respectively from 1 to 53 pg/mL and 2 to 44 pg/mL, for group 3 and 4. In both aforementioned groups, there were no detectable IL-12 levels or very few samples with detectable levels after Day 8.

HIV-1 Env-specific IgG was detected in serum samples of 10 out of 20 animals of group 2 and in 8 of 20 animals in group 3, on sampling occasion Days 85 and 106. The persistence of the IgG levels post dose could not be determined as almost all animals assigned to the recovery phase did not have any detectable IgG levels throughout the study.

HIV-1 Gag-specific IgG was detected at constant levels in all serum samples of groups 2 and 3 on sampling occasion Days 85, 106, and prior to recovery necropsy (Day 135). There were no overall clear differences in HIV-1 Gag-specific IgG levels in those two

groups. Although HIV-1 Gag-specific IgG was detected in all group 4 animals, the levels were considerably lower than those seen in groups 2 and 3.

In conclusion, the administration of HIV-MAG with or without the combination of *IL-12* pDNA in rabbits on Days 1, 22, 43, 64, 85 and 106; where a subpopulation of HIV-MAG and *IL-12* pDNA-treated animals (Days 1, 22 and 43) received booster vaccinations with VSV HIV *gag* on Days 64 and 85 by IM injection/EP was associated with changes in body weights, food consumption, clinical and anatomical pathology, generally with complete or partial resolution of these changes by the end of the recovery period. Peak human IL-12 levels were detected 48 hours post dose and on Days 4 and 8 in animals administered *IL-12* PDNA (ie, groups 3 and 4 animals). HIV-1 Env-specific IgG was detected in animals of groups 2 to 3 and in one animal of group 4 while HIV-1 Gag-specific IgG was detected in animals of groups 2 to 3 throughout the dosing and recovery periods, and to a lesser degree in animals of group 4.

4.8.4 VSV HIV *gag* toxicity in rabbits at $3.0x10^7$ PFU "Charles River Study No. 503415"

For the toxicology study, groups of 20 rabbits (10 male, 10 female) were inoculated IM with rVSVN4CT1*gag*1 vectors Table 4-4. A total dose of 3×10^7 PFU was delivered at 2 injection sites; each site received 1 mL containing 1.5×10^7 PFU of virus. This is equivalent to the maximum intended dose in humans. In a modified repeat dose testing regimen, another group of rabbits first received 3.0×10^7 PFU $rVSV_{IN}$ N4CTI*gag*1, then was boosted with 4×10^7 PFU of the heterologous NJ serotype vector ($rVSV_{NJ}$N4CTI*gag*1). As nAb develops against $rVSV_{IN}$ N4CTI*gag*1 with the first dose, which could neutralize homologous virus in a second dose and mask potential toxicity, the heterologous serotype prime-boost regimen represents a more rigorous test than traditional repeat dose testing. Control animals received vaccine matrix (3.8 mM KH2PO4, 7.2 mM K2HPO4, 7.5% sucrose, 5mM L- glutamate, and 0.2% hydrolyzed gelatin type A, pH6.8 – 7.4). Rabbits were monitored for 28 days postinoculation for any clinical signs of disease and reaction to the vaccine(s). Blood and saliva samples were collected 1 day prior to inoculation and on days 2, 4, 8 and 28 postinoculation to assay for virus dissemination and shedding. Urine was collected from the bladder at necropsy to assay for the presence of virus. Serum was collected prior to dosing and then at intervals postinoculation to measure the Gag-specific humoral response and VSV-specific nAbs.

Table 4-4 VSV HIV *gag* toxicology study schema

Group Number Identification	Route of Administration	Dose volume/ site (mL)	Total Dose Volume[a] (mL)	Animal Number Males	Animal Number Females
1 / Vector Vehicle[b]	IM	1	2	1101, 1002, 1003, 1104, and 1005 to 1010	1601, 1502, 1603, and 1504 to 1510
2 / $rVSV_{IN}$N4CT1*gag*1[c]	IM	1	2	2001 to 2003, 2104, 2005, 2106, and 2007 to 2010	2501 to 2506, 2607, and 2508 to 2510
3 / $rVSV_{NJ}$N4CT1*gag*1[c]	IM	1	2	3001, 3002, 3103, and 3004 to 3010	3601, 3502 to 3504, 3605, and 3506 to 3510
4 / $rVSV_{IN}$N4CT1*gag*1 (Day1) and $rVSV_{NJ}$N4CT1*gag*1 (Day 29)[d]	IM	1	2	4001, 4102, and 4003 to 4010	4501 to 4506, 4607, and 4508 to 4510

[a]Administered to the right and left thigh muscles, for a targeted dose of 10^8 PFU per animal (actual dose 3.0×10^7 PFU for $rVSV_{IN}$ N4CT1*gag*1 and 4.0×10^7 PFU for $rVSV_{NJ}$ N4CT1*gag*1).

[b]Injection on Day 1 (All Group 1 animals); 3 animals/sex sacrificed on Day 4 and 2 animals/sex sacrificed on Day 29 followed by injection of surviving Group 1 animals on Day 29; 3 animals/sex sacrificed on Day 32 and the remaining 2 animals/sex sacrificed Day 57.

[c]Injection of all animals on Day 1; 5 animals/sex/group sacrificed on Day 4 and the remaining 5 animals/sex/group sacrificed on Day 29.

[d]5 animals/sex/group sacrificed on Day 33 and the remaining 5 animals/sex/group sacrificed on Day 57.

There were no rVSV-related changes in clinical observations including body weight, food consumption, injection site findings, ophthalmic examinations and no changes were seen in bone marrow proliferation and maturation. On Day 1 and/or Day 29 (dosing occasions), transient increases in mean body temperatures of Groups 2 to 4 were seen at the 6-hour post dose timepoint and returning to predose values as early as 24 hours post dose, when compared to pretreatment values and to the Vector Vehicle group.

Transient changes in hematology parameters observed were increases in neutrophils and fibrinogen, decreases in hemoglobin, hematocrit, and platelet count and an increase in activated partial thromboplastin. Lymphoid hyperplasia was observed in the iliac lymph nodes. Minimal to slight intramuscular inflammation often with myofiber degeneration was seen at the injection site; minimal to slight epineural inflammation observed in the sciatic nerve of Group 4 animals only, was interpreted as a possible extension of injection site intramuscular inflammation. Transient changes in creatine kinase and C-reactive protein were also observed. All of the observed changes showed evidence of either complete or partial recovery during the observation period. Based on the above findings, both $rVSV_{IN}$N4CT1*gag*1and/or $rVSV_{NJ}$N4CT1*gag*1administered individually or sequentially were well tolerated overall. Finally, no virus was detected in blood, saliva, and urine at any timepoint assayed postinoculation.

4.8.5 Neurovirulence potential of candidate VSV vaccine vectors “Sierra Study ADO0003”

The attenuated VSV HIV *gag* vector has undergone extensive pre-clinical safety testing in stringent mouse and NHP neurovirulence (NV) models. In mice the VSV HIV *gag* vector did not produce any significant clinical signs of disease following either IM or intracranial (IC) inoculation of mice with 10^7 PFU of virus. Mice are exquisitely sensitive to VSV by the IC route of inoculation (50% lethal dose – LD_{50}, is 1-5 PFU for wild type isolates of VSV), and results from the murine NV studies confirm the highly attenuated phenotype of the clinical candidate (Figure 4-2).

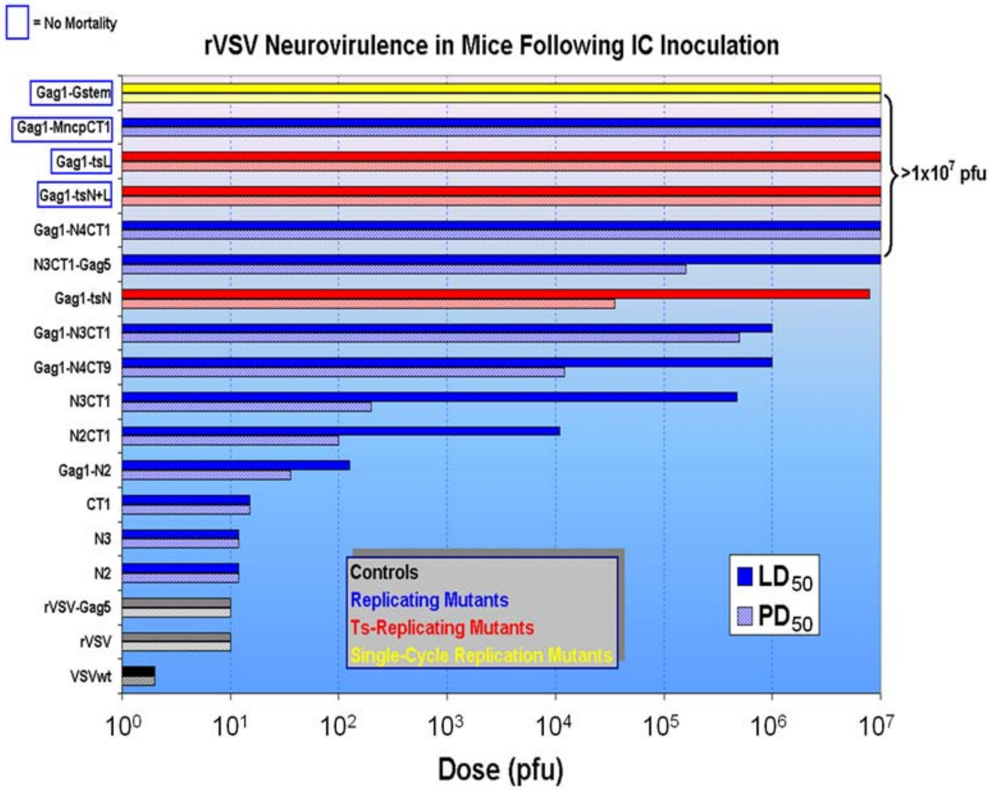

Figure 4-2 LD_{50} titers for rVSV vectors. Groups of 10 mice were inoculated intra-cranially (IC) with serial 10-fold dilutions of each rVSV vector. Cumulative lethality across dilutions tested was used to calculate an LD_{50} for each vector. The $rVSV_{IN}$N4CT1gag1 clinical candidate (Gag1-N4CT1) did not cause any lethality at doses up to 10^7 PFU, and an LD_{50} could not be determined. For comparison VSVwt (wild type VSV) had an LD_{50} titer of 2-3 PFU.

In concordance with results from the murine NV model, the $rVSV_{IN}$N4CT1gag1 vector caused no clinical signs of disease following either IM inoculation or direct intra-thalamic (IT) injection of NHPs with the anticipated clinical dose (10^7 PFU), and produced predominantly mild inflammatory responses in the CNS located mainly close to the IT site of inoculation (Unpublished data).

4.8.6 In vivo biodistribution of rVSV vaccine vectors in mice “Wyeth Study RPT-64154”

Virus bio-distribution studies have been performed in mice to investigate the degree of virus replication and dissemination in a permissive host, following IM inoculation. Mice were inoculated with 10^8 PFU of $rVSV_{IN}$N4CT9*gag*1 (10X the anticipated clinical dose),

a closely related but more replication competent vector than $rVSV_{IN}N4CT1$*gag*1 (VSV HIV *gag*). Vector replication and spread was monitored by assay of infectious virus and by virus specific qPCR performed on tissue, blood and organ samples collected at intervals post inoculation. The major finding from these studies was the localization of virtually all measurable virus replication at the IM site of inoculation and in the major draining, popliteal lymph node. Viral replication peaked by day 1- 2 post inoculation and there was no evidence of viral gene expression by day 10 post inoculation. [32]

4.8.7 Summary of pre-clinical safety data

Results of the biodistribution, the single and repeat dose toxicology studies conducted with HIV-MAG alone or in combination with *IL-12* pDNA, delivered IM in combination with EP support the clinical evaluation of the HIV MAG pDNA with *IL-12* pDNA. Testing in stringent mouse and NHP NV models, as well as biodistribution and shedding studies in mice conducted with the attenuated VSV HIV *gag* vector, support moving these candidate vaccines into clinical evaluation.

4.9 Preclinical immunogenicity studies

Table 4-5 Summary of preclinical immunogenicity studies

Study number	Product	Animal	N	Dose groups	Route	Schedul e	Assay
Rh-032	Multiantigen pDNA plus *IL-12* pDNA	Rhesus	6	10mg HIV pDNA	IM	0, 4, 8 weeks	ELISpot
			6	2mg HIV pDNA	EP		

4.9.1 Preclinical immunogenicity of Profectus Biosciences' HIV-1. multiantigen pDNA vaccine with *IL-12* with and without in vivo electroporation in rhesus macaques "Study No. Rh-032"

In a pre-clinical study, groups of rhesus macaques were immunized at week 0, 4 and 8 with either 10 mg of an HIV-1 multiantigen DNA vaccine plus rhesus *IL-12* pDNA by standard IM injection or 2.0 mg of the DNA vaccine plus *IL-12* pDNA by IM injection followed by EP. The results indicate (Figure 4-3) that EP led to stronger cellular responses versus IM injection as measured by IFN-γ ELISpot assay. At 8 and 22 weeks after the final pDNA immunization there was a 10- and 45-fold increase in HIV-specific ELISpot responses compared to the non-EP group translating to an apparent 50- or 225-fold increase in pDNA vaccine potency, respectively. Importantly, EP enhanced the immune response against the less immunogenic antigens (*nef*-*tat*-*vif*) resulting in a more balanced immune response. In addition, antibody responses against Env showed that EP led to ~2.5-log increase in antibody titer as compared to the non-EP group [6].

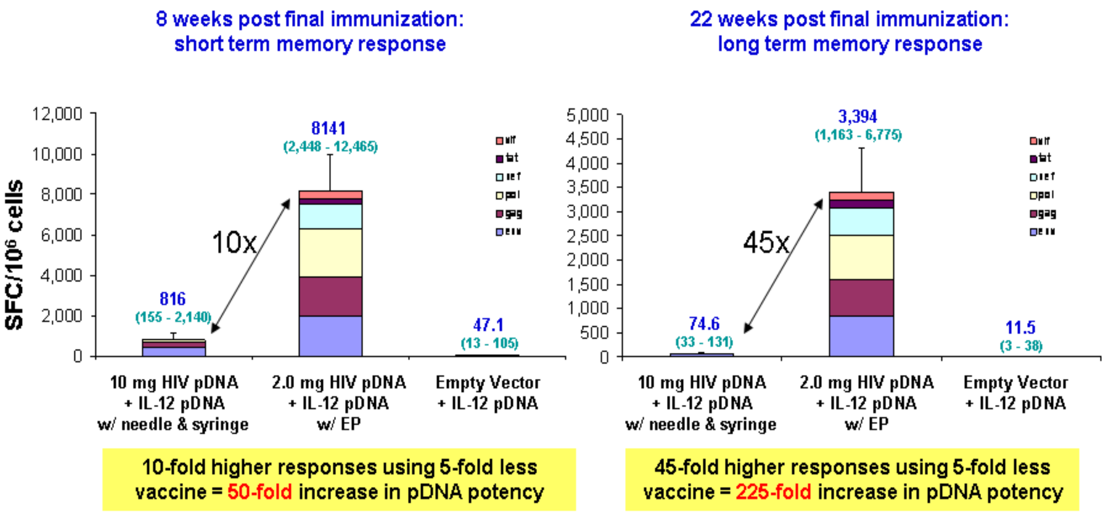

Figure 4-3 Total vaccine-specific cellular immune responses in rhesus macaques are dramatically improved through the use of in vivo EP

4.9.2 Preclinical Immunogenicity of rVSV *gag* p55

The first NHP experiments to evaluate rVSV as a vector system HIV were conducted by Dr. John Rose and colleagues at Yale. In these pioneering studies, rhesus macaques were immunized with a combination of two prototypic rVSV vectors expressing an HIV-1–89.6 *env* gp160/VSV-G fusion polypeptide and simian immunodeficiency virus (SIV) *gag* p55 protein. In this study, all 7 macaques receiving rVSV expressing HIV *env* and SIV *gag* remained disease-free after simian/human immunodeficiency virus (SHIV)-89.6P challenge for > 4 years. By comparison, all 8 control macaques progressed to AIDS with an average time of 8 months. The protection from AIDS in this study correlated with large differences in peak viral loads, low or undetectable viral loads at setpoint, and with the preservation of CD4+ T cells in the vaccinees relative to controls. This encouraging level of post-challenge vaccine efficacy suggested that rVSV vectors expressing HIV genes might be an effective AIDS vaccine in humans.

In a more recent pre-clinical study, groups of rhesus macaques were immunized at weeks 0 and 8 with $1x10^7$ PFU of a highly attenuated replication competent rVSV vaccine vector encoding HIV-1 *gag* p55 (VSV HIV *gag*) by standard IM injection. For comparison, another group of rhesus macaques were immunized with $1x10^7$ PFU of the much more virulent prototypic rVSV vector (rVSV-HIV *gag*5) shown previously by Jack Rose and colleagues to protect macaques against simian-AIDS in a pathogenic SHIV challenge model. The data demonstrate that the highly attenuated rVSV vector was as good as the more virulent vector in eliciting HIV *gag*-specific IFN-γ ELISpot responses following vaccination (Figure 4-4).

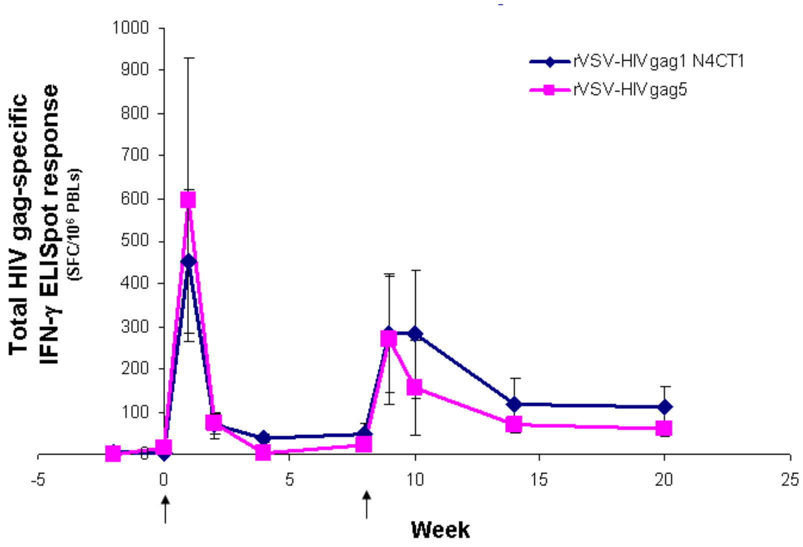

Figure 4-4 Induction of HIV *gag*-specific IFN-γ ELISpot responses in rhesus macaques following immunization with a highly attenuated replication competent rVSV vaccine vector

Recently, it has been demonstrated that priming with a series of IM injections of plasmid DNA *IL-12* and SIV *gag* effectively enhanced the immunogenicity and post-challenge efficacy of two doses of rVSV expressing HIV*env* and SIV*gag* in rhesus macaques. As shown in Figure 4-5, rhesus macaques receiving the pDNA prime, rVSV boost vaccination regimen demonstrated significantly increased SIV*gag*-specific cell-mediated immune responses relative to macaques receiving only the pDNA or the rVSV vectored immunizations. In addition, macaques receiving the combination plasmid DNA prime, rVSV boost vaccination regimen demonstrated significantly lower viral loads post-intravenous SHIV-89.6P challenge relative to macaques receiving only the rVSV vectored immunizations (data not shown).

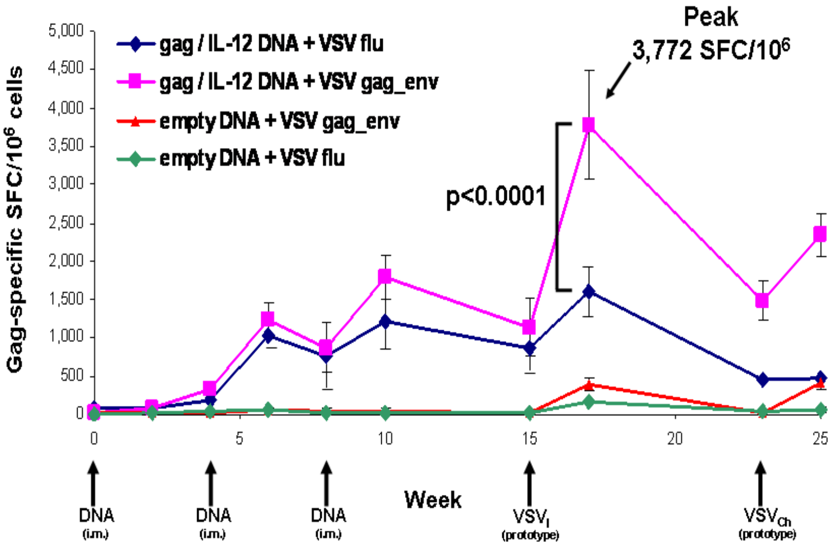

Figure 4-5 Induction of HIV Gag-specific IFN-γ ELISpot responses in rhesus macaques following immunization with a heterologus cytokine-enhanced pDNA prime, rVSV boost vaccination regimen

4.10 Clinical studies

Clinical experience with the HIV-MAG vaccine, the *IL-12* pDNA adjuvant and VSV HIV *gag* is summarized in Table 4-6. More detail on the clinical trials summarized in Table 4-6 is presented in the following sections.

Table 4-6 Summary of clinical studies

Vaccine component	Previous clinical experience	Notes
IL-12 pDNA	Healthy Adults:<br>HVTN 060, HVTN 063, HVTN 070, HVTN 080	To date, 146 healthy adult volunteers have received *IL-12* pDNA without EP, and 30 have received *IL-12* pDNA with EP.
	HIV+ individuals:<br>Wyeth study # 6120K1-100	To date, 10 HIV+ patients have received *IL-12* pDNA by IM injection
In vivo EP via the *TriGrid™ delivery device*	Healthy adults:<br><br>Rockefeller University Hospital, ClinicalTrials.gov number NCT00545987	24 subjects have received a DNA vaccine encoding HIV-1 subtype B'/C antigens env, gag, pol, nef, and tat using the intramuscular TriGrid Delivery Device. Subjects have received up to three administrations at total DNA doses of up to 4.0mg. An additional 8 subjects received placebo via the TriGrid device.
	Emory Vaccine Trials Evaluation Unit, ClinicalTrials.gov number NCT01169077 (ongoing)	10 subjects have received a DNA vaccine encoding multiple CD4+ and CD8+ epitopes derived from proteins expressed by *Plasmodium falciparum* using the intramuscular TriGrid Delivery Device. Subjects have received three administrations at a DNA dose of 0.25 mg. An additional 3 subjects received placebo via the TriGrid device.
	Stage IIB-IV Melanoma Patients:<br><br>Memorial Sloan Kettering Cancer Center 07-003	23 patients have been administered a DNA vaccine encoding a xenogeneic form of the tyrosinase antigen using the intramuscular TriGrid Delivery Device. Patients have received up to 5 administrations at DNA doses of up to 1.5 mg.

4.10.1 Clinical studies using Profectus Biosciences' HIV-1 multiantigen pDNA vaccine (HIV-MAG), the proposed product combination, and prime-boost regimen with VSV_{IN} *gag*

No human data are available for the HIV-MAG vaccine, the proposed product combination with IL-12 pDNA**,** or the prime-boost regimen with VSV_{IN} *gag*.

The NIAID-funded ACTG is conducting protocol A5281, a multicenter, phase 1, placebo-controlled, dose-escalation study that will enroll 60 HIV-infected subjects on stable anti-retroviral therapy. It will assess the safety and immunogenicity of a fixed 3 mg dose of the Profectus HIV-MAG vaccine administered with escalating doses to 1 mg of GENEVAX™ IL-12 pDNA adjuvant and delivered with EP using Ichor TDS. This study started enrolling April 2011.

HVTN 090, A phase 1 clinical trial to evaluate the safety and immunogenicity of VSV_{IN} HIV *gag* vaccine given intramuscularly in healthy, HIV-1–uninfected adult participants, is expected to start Q4, 2011.

4.10.2 Clinical studies using *IL-12* pDNA

The human *IL-12* pDNA to be used in the current study has been evaluated for safety and adjuvant activity in a number of clinical studies conducted in healthy adult volunteers (HVTN 060, 063, 070 and 080) and HIV+ individuals (6120K1-100).

4.10.2.1 Phase 1 study of prototype HIV-1 gag p37-expressing pDNA construct in combination with human IL-12 pDNA delivered by standard IM injection in HIV-negative adults (HVTN 060)

The HVTN has conducted a phase 1 trial, HVTN 060 (BB#12367, DAIDS-ES ID 10057), which evaluated the safety and immunogenicity of a prototype HIV-1 *gag* p37 expressing pDNA vaccine (GENEVAX® *gag*-2962) alone or in combination with human *IL-12* pDNA (GENEVAX® *IL-12*-6285 or -4532). Participants included 132 volunteers in the US and 12 Part B volunteers in Thailand. See Table 4-7

Table 4-7 Trial schema for HVTN 060

		Vaccination schedule in months (days)							
		Dose (mcg)			Priming			Boosting	
Groups	N	gag	IL-12	CTL MEP	0 (0)	1 (28)	3 (84)	6 (168)	9 (273)
Part A									
1	10	1500	—	—	*gag* DNA	*gag* DNA	*gag* DNA	—	—
	2	—	—	—	placebo	placebo	placebo	—	—
2	10	1500	100	—	*gag* DNA + *IL-12* DNA	*gag* DNA + *IL-12* DNA	*gag* DNA + *IL-12* DNA	—	—
	2	—	—	—	placebo	placebo	placebo	—	—
3	10	1500	500	—	*gag* DNA + *IL-12* DNA	*gag* DNA + *IL-12* DNA	*gag* DNA + *IL-12* DNA	—	—
	2	—	—	—	placebo	placebo	placebo	—	—
4	10	1500	1500	—	*gag* DNA + *IL-12* DNA	*gag* DNA + *IL-12* DNA	*gag* DNA + *IL-12* DNA	—	—
	2	—	—	—	placebo	placebo	placebo	—	—
Part B									
5	30	1500	—	—	*gag* DNA	*gag* DNA	*gag* DNA	*gag* DNA	*gag* DNA
	6	—	—	—	placebo	placebo	placebo	placebo	placebo
6	30	1500	1500	—	*gag* DNA + *IL-12* DNA	*gag* DNA + *IL-12* DNA	*gag* DNA + *IL-12* DNA	*gag* DNA + *IL-12* DNA	*gag* DNA + *IL-12* DNA
	6	—	—	—	placebo	placebo	placebo	placebo	placebo
7	30	1500	1500	1000	*gag* DNA + *IL-12* DNA	*gag* DNA + *IL-12* DNA	*gag* DNA + *IL-12* DNA	CTL MEP	CTL MEP
	6	—	—		placebo	placebo	placebo	placebo	placebo
Total	48 (Part A) + 108 (Part B) = 156								

There were no adverse events (AEs) among vaccinated participants that were considered probably or definitely related to vaccination. The vaccine and cytokine adjuvant did not have any apparent effects on hematologic parameters (complete blood count), CD4 counts, or serum chemistries compared to placebo. There were no fatalities reported during the trial, no life-threatening AEs, and no serious adverse events (SAEs).

An assay for *IL-12* antibody was performed, to determine whether administration of *IL-12* DNA might have elicited antibody that might affect native IL-12. Evaluated samples were blinded to group assignment and to assignment to the vaccine or placebo arm. One person had a positive result (32.3 NU; a positive response was >30 NU) for antibody to IL-12 at day 14 that was not present at baseline, day 42, or day 98.

Immunogenicity was assessed by IFN-γ ELISpot after 3rd and 4th vaccinations, using peptide pools representing clade B consensus Gag. Unblinded results from group 1 and group 5 (*gag* DNA alone) pooled show a 2/33 (6.1%) IFN-γ ELISpot response rate 2 weeks after the third injection; both participants were in group 5. Two weeks after the fourth injection 2/23 participants (8.7%) in group 5 also had positive ELISpot responses; one of those had responded earlier. There were no responses in group 2 (*gag* and *IL-12* DNA 100 mcg). In group 3, 4/9 (44.4%) participants responded after 3 vaccinations with *gag* DNA and *IL-12* DNA 500 mcg (95% confidence interval 18.9%-73.3%). In groups 4, 6, and 7, no responses were seen after 3 vaccinations of *gag* with *IL-12* DNA 1500 mcg among 49 samples assayed. In addition, 24 samples from group 6 were tested after the fourth injection, and 11 samples were tested after the fifth injection, and no responses were detected at those timepoints. The range of background adjusted spot counts among the positive samples from groups 3 and 5 was 55-141 spot-forming cells (SFC)/10^6 peripheral blood mononuclear cells (PBMC).

Serological tests for binding antibodies to p55 *gag* were assessed by a validated enzyme-linked immunosorbent assay (ELISA) using single serum dilutions (1/100), at baseline, two weeks after third vaccination, and after fifth vaccination for Part B participants. No samples were positive for binding antibodies to p55 *gag*.

In summary, local and systemic reactions to vaccination with the HIV-1 *gag* p37 pDNA vaccine GENEVAX® *gag*-2962) and *IL-12* pDNA (GENEVAX® *IL-12*-6285 and GENEVAX® *IL-12*-4532) were mild to moderate in the HVTN 060 phase 1 trial. No pattern of systemic AEs emerged during the study, and no SAEs related to the study products were observed. The pDNA vaccine and the *IL-12* pDNA cytokine adjuvant have been well-tolerated, with an acceptable safety profile in this trial. The pDNA vaccine regimen delivered by standard IM injection was very minimally immunogenic.

4.10.2.2 Phase 1 study of prototype HIV-1 gag p37-expressing pDNA construct in combination with human IL-15 pDNA or IL-12 pDNA delivered by standard IM injection in HIV-negative adults (HVTN 063)

HVTN 063 (BB IND# 12439, DAIDS-ES ID 10058) tested the safety and immunogenicity of a prototype HIV-1 *gag* (p37) pDNA vaccine (GENEVAX® *gag*-2962) alone or with plasmids encoding *IL-15* (GENEVAX® *IL-15*-1696), and *IL-12* (GENEVAX® *IL-12*-4532) in healthy, HIV-1–uninfected adults. This was the first-time-in-humans evaluation for GENEVAX® *IL-15*-1696 as a molecular adjuvant.

In this study, participants received 1500 mcg HIV *gag* p37 pDNA vaccine with *IL-15* DNA at 0, 100, 500, or 1500 mcg, or placebo. The study had two parts: Part A, a dose escalation study of *IL-15* DNA, and Part B, a regimen selection study, of *gag* DNA with *IL-15* DNA, given up to 5 times, or as 3 injections subsequently boosted by two injections of *gag* DNA with *IL-12* DNA. See Table 4-8.

Table 4-8 Trial schema for HVTN 063

					Vaccination schedule in months (days)			Booster schedule in months (days)	
Study arm	N	*gag* DNA dose (mcg)	*IL-15* DNA dose (mcg)	*IL-12* DNA dose (mcg)	0 (0)	1 (28)	3 (84)	6 (168)	9 (273)
**Part A**									
1	10	1500	—	—	*gag* DNA	*gag* DNA	*gag* DNA	—	—
	2	—	—	—	placebo	Placebo	placebo	—	—
2	10	1500	100	—	*gag* DNA + *IL-15* DNA	*gag* DNA + *IL-15* DNA	*gag* DNA + *IL-15* DNA	—	—
	2	—	—	—	placebo	Placebo	placebo	—	—
3	10	1500	500	—	*gag* DNA + *IL-15* DNA	*gag* DNA + *IL-15* DNA	*gag* DNA + *IL-15* DNA	—	—
	2	—	—	—	placebo	Placebo	placebo	—	—
4	10	1500	1500	—	*gag* DNA + *IL-15* DNA	*gag* DNA + *IL-15* DNA	*gag* DNA + *IL-15* DNA	—	—
	2	—	—	—	placebo	Placebo	placebo	—	—
Pause for safety evaluation									
**Part B**									
5	30	1500	1500	—	*gag* DNA + *IL-15* DNA	*gag* DNA + *IL-15* DNA	*gag* DNA + *IL-15* DNA	*gag* DNA + *IL-15* DNA	*gag* DNA + *IL-15* DNA
	6	—	—	—	placebo	placebo	placebo	placebo	placebo
6	0	—	—	—	—	—	—	—	—
	0	—	—	—	—	—	—	—	—
7	30	1500	1500	1500	*gag* DNA + *IL-15* DNA	*gag* DNA + *IL-15* DNA	*gag* DNA + *IL-15* DNA	*gag* DNA + *IL-12* DNA	*gag* DNA + *IL-12* DNA
	6	—	—	—	placebo	placebo	placebo	placebo	placebo
Total: A (48) + B (72) = 120									

In all, 100 people received the *gag* DNA vaccine, 67 men and 53 women: 10 received *gag* DNA vaccine alone with no adjuvant, 64 received vaccine with *IL-15* DNA adjuvant, and 26 received vaccine with each of the two adjuvants (*IL-15* DNA and *IL-12* DNA). Twenty people received the placebo (Sodium Chloride for Injection USP, 0.9%).

Participants were asked to record and report reactogenicity symptoms for 3 days following each injection, or until resolution of symptoms, including: injection site pain, tenderness, erythema, and/or induration/swelling/edema; systemic symptoms including malaise and/or fatigue, myalgia, headache, nausea, vomiting, chills, arthralgia; and temperature. The vaccinations were well tolerated. Injection site pain and/or tenderness, and systemic symptoms such as malaise/fatigue, chills, nausea, myalgia, arthralgia or headache, have been mild or absent in most participants. There were no severe reactions related to vaccine. There were no fatalities reported during the trial.

AEs that were reported as “definitely related” or “probably related” to vaccination were mild in severity: 1 event each of injection site pruritis (T1), injection site swelling (T1), injection site pain (T7), injection site papule (T7), pyrexia (T7), and injection site hematoma (T5).

An assay for *IL-15* neutralizing antibody (nAb) was performed, to determine whether administration of *IL-15* DNA might have elicited antibody that might affect native IL-15. For the 65 participants tested from Part B groups 5 and 7, an assay for *IL-12* nAb was

also done. Evaluated samples were blinded to group assignment and to assignment to the vaccine or placebo arm. There were no positive results in 38 Part A vaccinees and 8 controls tested at baseline and after the third vaccination. From Part B, 2/26 group 7 vaccinees, 0/28 group 5 vaccinees, and 0/11 controls had positive results for *IL-15* nAb at day 273, after 4 vaccinations. A positive response was defined as a result > 11 NU/mL. The group 7 participants had responses of 11.5 and 17.7 NU/mL, respectively. No Part B participant had a positive result for *IL-12* nAb, defined as a result > 30 NU/mL.

Immunogenicity was assessed by validated IFN-γ ELISpot 2 weeks after 3rd and 4th vaccinations, using cryopreserved PBMC stimulated overnight with synthetic peptide pools representing clade B consensus *gag*. The sums of background-corrected responses to *gag* ConB from the 3 responders from treatment group 5 were: 195, 185 and 105 SFC/10^6 PBMC. The sum of background-corrected responses to *gag* ConB from the single responder in treatment group 7 was 113 SFC /10^6 PBMC. One participant in a placebo group from Part A had a positive response at baseline and after 3 injections of placebo. Due to the low frequency of responses after the third and fourth vaccinations, samples after the fifth vaccination were not assessed.

Serological tests for binding antibodies to p55 *gag* were assessed by a validated ELISA using single serum dilutions (1/100), at baseline, two weeks after third vaccination for Part A participants (groups 1-4: 37 vaccinees and 8 controls tested), and after fifth vaccination for Part B participants (groups 5 and 7: 56 vaccinees and 12 controls tested). No samples were positive for vaccine-induced binding antibodies to p55 *gag*. One participant in group 7 had a positive result at baseline and after fifth vaccination.

In summary, local and systemic reactions to vaccination with the Gag DNA vaccine GENEVAX® Gag-2962) and *IL-15* DNA (GENEVAX® *IL-15*-1696), and *IL-12* DNA (GENEVAX® *IL-12*-4532) have been mild to moderate in the HVTN 063 phase 1 trial. No pattern of systemic AEs related to vaccination emerged during the study, and no SAEs related to the study vaccines were observed. The vaccines have been well-tolerated, with an acceptable safety profile in this trial.

4.10.2.3 Phase 1 study of PENNVAX-B™ pDNA construct expressing HIV gag, pol and env in combination with human IL-15 pDNA or IL-12 pDNA delivered by standard IM injection in HIV-negative adults (HVTN 070)

The HVTN 070 clinical trial (BB IND# 13449, DAIDS-ES ID 10490) was designed to evaluate the safety and immunogenicity of PV-B (*gag*, *pol*, *env*) alone, with *IL-12* DNA (GENEVAX®-*IL-12*-4532), or with a dose escalation of *IL-15* DNA (pIL15EAM). PV-B and *IL-15* DNA were provided by David Weiner, University of Pennsylvania School of Medicine (Philadelphia, PA, USA). The *IL-12* DNA was provided by Profectus Biosciences, (Tarrytown, NY, USA). HVTN 070 was initiated in October, 2007. Enrollment was completed in January, 2009, and all vaccinations were completed by August, 2009.

Table 4-9 Trial schema for HVTN 070

		Dose			Vaccination schedule in months (days)			
**Study arm**	**Number**	**PV-B**	***IL-15* DNA**	***IL-12* DNA**	**0 (0)**	**1 (28)**	**3 (84)**	**6 (168)**
Group 1	10	6 mg	0.8 mg	—	PV-B + *IL-15*	PV-B + *IL-15*	PV-B + *IL-15*	PV-B + *IL-15*
	2	—	—	—	Control	Control	Control	Control
Group 2	30	6 mg	—	—	PV-B	PV-B	PV-B	PV-B
	6	—	—	—	Control	Control	Control	Control
Group 3	30	6 mg	—	1.5 mg	PV-B + *IL-12*	PV-B + *IL-12*	PV-B + *IL-12*	PV-B + *IL-12*
	6	—	—	—	Control	Control	Control	Control
Group 4	30	6 mg	2 mg	—	PV-B + *IL-15*	PV-B + *IL-15*	PV-B + *IL-15*	PV-B + *IL-15*
	6	—	—	—	Control	Control	Control	Control
Total	120 (100 vaccine + 20 control)							

PV-B = PENNVAX™-B

Thirteen participants discontinued the vaccination series early.

Six participants were discontinued from vaccination by the HVTN 070 PSRT for clinical reasons or AEs. All were vaccine recipients:

- One person in group 1 was discontinued from vaccinations after the second vaccination, due to Grade 2 neutropenia, probably not related to vaccination.

- One person in group 2 was discontinued from vaccinations after the third vaccination, due to the subject's relapse into drug abuse involving intranasal heroin, and an associated Grade 3 weight loss, which were not related to vaccination.

- One person in group 4 was discontinued from vaccinations after the second vaccination, for symptoms occurring 10 minutes after vaccination, including globus sensation, bilateral hand paresthesias, nausea, and throat tingling. Although symptoms were mild and self-limited and the site clinician suspected the symptoms were due to pre-existing gastroesophageal reflux disease and anxiety, the site clinician and the PSRT were concerned that the symptoms could possibly represent an allergic reaction and opted not to revaccinate the participant.

- One participant in group 4 was discontinued for active cocaine use.

- One participant in group 2 was discontinued for moderate atypical lateral epicondylitis, considered not related to vaccination.

- One participant in group 2 was discontinued for cervical radiculopathy with an exacerbation of peripheral sensorimotor neuropathy (a pre-existing condition that was undisclosed at study entry) after 1 vaccination. This was considered to be

possibly related to vaccination by the site investigator, and was also reported as an SAE.

Thirteen participants discontinued the study early: 1 was unable to adhere to the visit schedule, 5 relocated, 6 were unable to be contacted, and 1 was no longer available.

Participants were asked to record and report reactogenicity symptoms for 3 days following each injection, or until resolution of symptoms, including: injection site pain, tenderness, erythema, and induration/swelling/edema; systemic symptoms, such as malaise and/or fatigue, myalgia, headache, nausea, vomiting, chills, arthralgia, and oral temperature. No participant reported severe reactogenicity symptoms. The maximum severity of reactogenicity symptoms can be compared in Figure 4-7 to the same assessments in HVTN 080, which gave the same products with EP.

There were no statistically significant differences observed for any of the reactogenicity signs or symptoms between the four vaccine arms. Combining vaccine arms and comparing to placebos, the vaccine arms had more pain ($p=0.02$), tenderness ($p < 0.0001$), and pain and/or tenderness ($p=.0008$). There were no differences for erythema, induration or systemic symptoms.

Three SAEs have been reported for this study: the exacerbation of peripheral sensorimotor neuropathy mentioned above, which led to the participant's discontinuation of vaccination in group 2; a death in group 2 which occurred 6 months after vaccination, which was considered probably not related to vaccination; and a hospitalization of a participant in group 3 with fever, flank pain, and abdominal lymphadenopathy which was considered probably not related to vaccination.

AEs that were assessed by site investigators as possibly, probably, or definitely related to vaccination with PV-B alone were 1 event each of alanine aminotransferase (ALT) increased, aspartate aminotransferase (AST) increased, hemoglobin decreased, injection site pruritis, blood creatinine increased, CD4+ lymphocytes decreased, microcystic anemia, peripheral sensorimotor neuropathy, and pharyngitis. AEs that were considered possibly, probably, or definitely related to vaccination with PV-B with IL-12 pDNA were 1 event of injection site hematoma and 1event of macular rash.

Flow cytometry was used to examine HIV-1-specific CD4+ and CD8+ T-cell responses using a validated intracellular cytokine staining (ICS) assay for IL-2 and/or IFN-γ. PBMC are stimulated with synthetic HIV-1 peptides that span the proteins encoded by the vaccine construct. The method used is based on Potential T-Cell epitopes (PTE). The primary cellular immunogenicity endpoints for HVTN 070 are responses at days 98 and 182 (i.e. visits 7 and 9), corresponding to 2 weeks following the 3rd and 4th vaccinations.

Responses were primarily for CD4+ T-cells, ranging from 19.2% - 28.0% among vaccinees at day 98 and from 28.6% - 40.7% at day 182. Responses to Pol (26/33) and Gag (20/33) were more frequent than responses to Env (10/33). CD8+ T-cell response rates were < 8% for all treatment arms at both days 98 and 182. These are summarized in Figure 4-8 for comparison with results from HVTN 080 which gave the same products with EP.

Neutralizing antibody titers against HIV-1 strains MN and SF162.LS were observed in low titers in a few vaccine and placebo recipients. The false positive rate from controls

was 5/36 = 14% for MN and 1/36 = 3% for SF162.LS. A small number of titers in vaccine groups were slightly higher than the false positives but overall the nAb responses were weak. For the MN isolate at day 98, vaccinees who received PV-B with IL-15 2 mg had the highest response rate, 25.9% (7/27), followed by participants receiving PV-B with IL-12 DNA with 20.0% (6/30), and PV-B alone, 3.7% (1/27). MN response rates were lower at day 182. No one in the group that received PV-B with IL-15 DNA 0.8 mg responded at either timepoint. There were no responses in the vaccine arms to SF162.LS, at either timepoint.

Binding antibodies to consensus B Env and Gag were assessed by a validated ELISA using single serum dilutions (1/20). There was one low level responder (out of 94 vaccinees assessed) who received PV-B + IL-12 DNA.

In summary, local and systemic reactions have been mild to moderate in the HVTN 070 phase 1 trial. Thirty participants have received PV-B 6 mg with *IL-12* DNA (GENEVAX®-*IL-12*-4532) 1.5 mg, of whom 27have received 4 injections. Also, an additional 70participants have received PV-B 6 mg with or without *IL-15* DNA (pIL15EAM), of whom 56 have received 4 injections. No severe reactions or other safety concerns related to the study products have been observed. Overall, the study products have been well-tolerated, with an acceptable safety profile to date. The T cell responses to PV-B were more frequent than previously seen in HVTN 060 and HVTN 063 with the Wyeth p37 *gag* DNA vaccine, and were CD4+. Responses were more frequent to Gag and Pol antigens relative to Env.

4.10.2.4 A phase 1 clinical trial to evaluate the safety and immunogenicity of PENNVAX™-B (gag, pol, env) vaccine, with or without IL-12 DNA plasmid, delivered via electroporation in healthy, HIV-1 – uninfected adult participants (HVTN 080)

IL-12 DNA is also being evaluated with PV-B (*gag*, *pol*, *env*) vaccine in HIV-uninfected healthy adults in HVTN 080. Both study products are delivered via EP. The trial uses the VGX CELLECTRA® EP system.

The trial schema is given below.

Table 4-10 Trial schema for HVTN 080

Study arm	N	Dose via EP	Vaccination schedule in months (days) 0 (0)	1 (28)	3 (84)
Group 1	10	3 mg PV-B	PV-B	PV-B	PV-B
	2		Placebo	Placebo	Placebo
Group 2	10	3 mg PV-B + 1 mg *IL-12* DNA	PV-B+ *IL-12* DNA	PV-B+ *IL-12* DNA	PV-B+ *IL-12* DNA
	2		Placebo	Placebo	Placebo
Group 3	20	3 mg PV-B + 1 mg *IL-12* DNA	PV-B+ *IL-12* DNA	PV-B+ *IL-12* DNA	PV-B+ *IL-12* DNA
	4		Placebo	Placebo	Placebo
Total		40 + 8 = 48			

PV-B = PENNVAX-B™

The first participant was enrolled November 9, 2009. Injections for the study were completed August 26, 2010.

As one measure of tolerability, participants are asked to mark along a 10 cm line (0 cm = no pain to 10 cm = worst pain) to indicate their perceived severity of discomfort related to EP at 3 timepoints: immediately after injection/EP, at 5 minutes, and at 30 minutes. Their visual analog scale (VAS) scores are indicated in Figure 4-6. The safety data are still blinded, so placebo recipients are included in the groups.

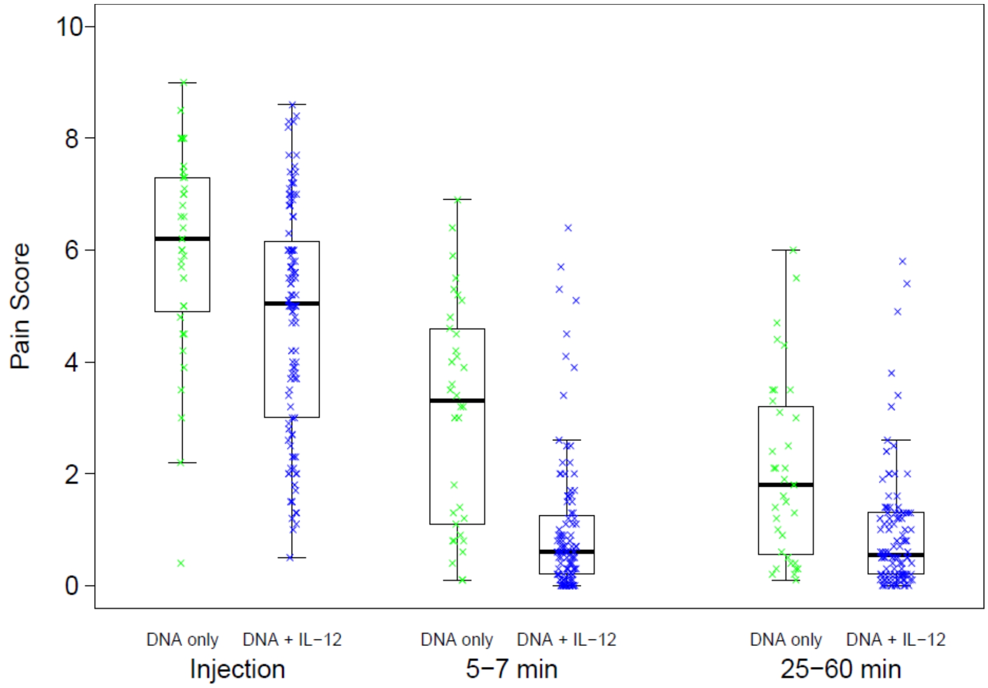

Figure 4-6 Visual Analog Scale scores, all vaccinations combined. Data includes placebos.

Of note, participants who received PV-B or placebo alone (DNA only group) reported significantly more pain on their VAS than participants who also received *IL-12* pDNA (DNA+IL12). Taking all visits together (and ignoring that there are multiple scores from the same people) p-values from Wilcoxon rank test are .003 at time 0, < .001 at 5 min, and < .001 at 25 minutes. One possibility is that this difference is due to the increased total dose of bupivacaine given with the addition of *IL-12* pDNA. Both the vaccine and adjuvant preparations are formulated in 0.25% bupivacaine.

No SAEs or safety concerns related to vaccination have been identified, as of February 2011. The following AEs were reported by the group that received PV-B vaccine or placebo given with EP and were considered related to the study product: ALT increase, decreased appetite, injection site pain and induration, each reported by 1 participant. AEs that were considered related to PV-B with IL-12 pDNA or placebo, given by EP, were: injection site reaction, presyncope, device malfunction, injection site pain, tenderness, induration, and erythema, each reported by 1 participant.

Three participants discontinued vaccinations early. One participant was discontinued from vaccination due to the discovery of an ongoing pre-existing condition, not previously known to the site, of bony disease of the spine. The participant presented with pain and numbness in the right leg which eventually led to an AE report of moderate exacerbation of pre-existing spinal stenosis L1-2, which was ongoing at the end of study participation. Two participants refused to receive additional vaccinations, as they considered the process or the reactogenicity symptoms too painful.

Reactogenicity refers to injection site or systemic symptoms within the first 3 days after vaccination. The maximum severity of reactogenicity symptoms reported by participants, occurring within 3 days of vaccination, are summarized in Figure 4-7 on the right in

comparison to results from HVTN 070, left. There were no severe reactogenicity symptoms.

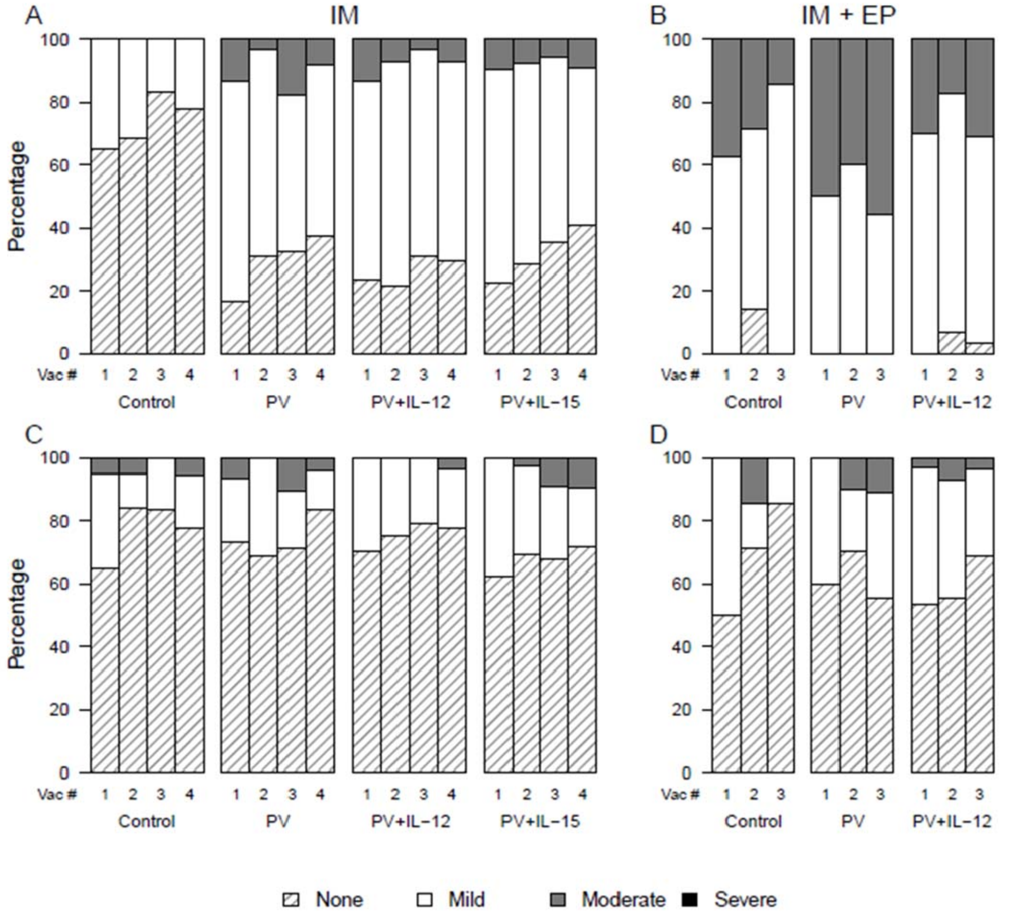

Figure 4-7 Maximum severity of rectogeniciy symptoms after vaccination with control or PV-B with IL-12pDNA or IL-15pDNA, and with or without EP. (A) Severity of injection site symptoms (pain/tenderness) in HVTN 070 (B) Severity of injection site symptoms in HVTN 080 (C) Severity of systemic symptoms (malaise and/or fatigue, myalgia, headache, nausea, vomiting, chills, arthralgia) in HVTN 070 (D) Severity of systemic symptoms in HVTN 080

For HVTN 080 there were no statistically significant differences for any reactogenicity signs/symptoms between PV-B alone compared to PV-B + IL-12 pDNA or for the vaccine arms compared to placebos.

Electroporation of PV-B and IL-12 pDNA improved immune responses significantly compared to the responses seen in HVTN 070 with standard IM injection, as seen in Figure 4-8 and Figure 4-9. Two weeks after the third vaccination, 81% of subjects receiving PV-B with IL-12 with EP had a CD4+ T cell response (median magnitude 0.2% by ICS), and 52% had a CD8+ T cell response (median magnitude 0.3% by ICS). The overall response rate for T cells was 89%. Responses were observed to all antigens, though few Env CD4+ T cell responses and few Gag CD8+ T cell responses were seen. In terms of adjuvant activity of the IL-12 pDNA, it was seen that after two vaccinations, 71.4% (20 out of 28) of subjects receiving PV-B plus IL-12 pDNA mounted an antigen-

specific T-cell response compared to only 30.0% (3 out of 10) of subjects receiving PV-B alone. Likewise, after three vaccinations: 88.9% (24 out of 27) receiving PV-B plus GENEVAX™ IL-12 pDNA mounted an antigen-specific T-cell response compared to only 66.7% (6 out of 9) of subjects receiving PV-B alone.

IL-12 pDNA and EP in this study showed a significant dose-sparing effect. In HVTN 080, people were vaccinated with cumulative doses of 9 mg PV-B and 3 mg IL-12 pDNA, compared to HVTN 070 in which people received up to 24 mg PV-B in the course of the study and yet had lower response rates.

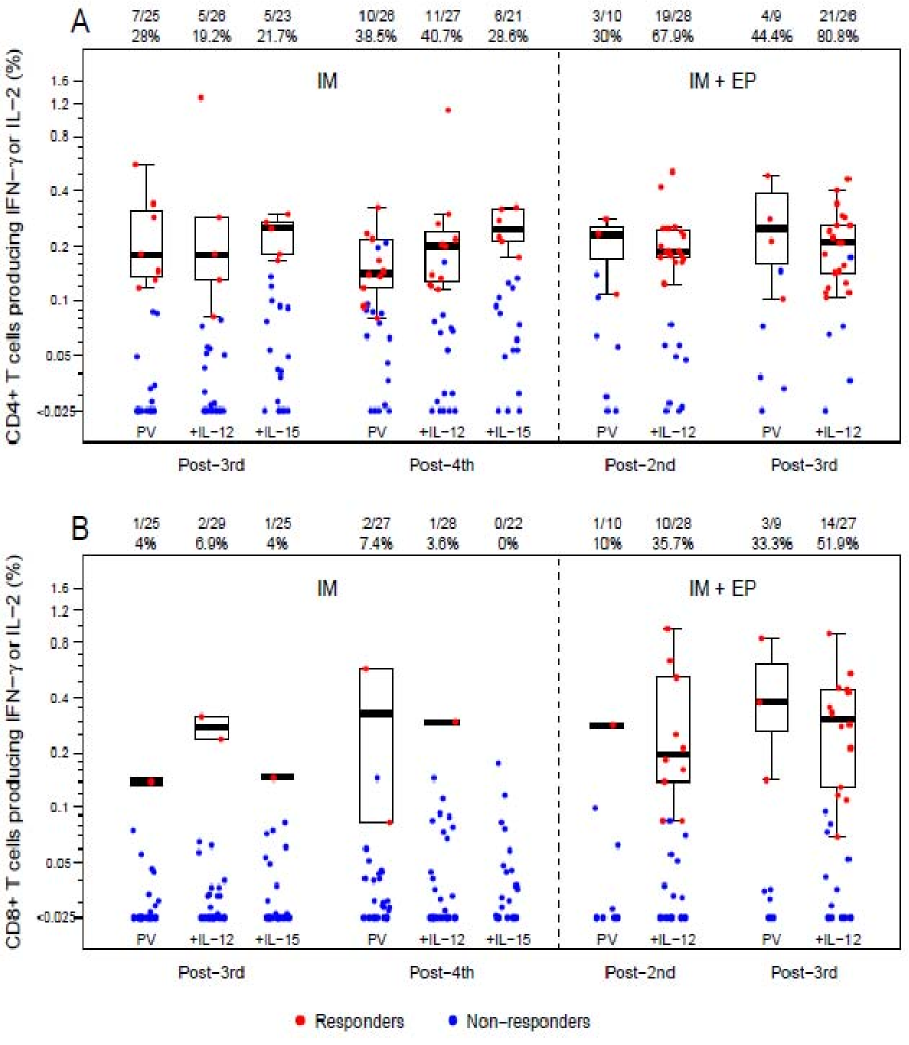

Figure 4-8 HVTN 070 and HVTN 080 ICS responses against ANY global PTE peptides, comparing standard IM injection to IM injection with EP and lower doses of product. (A) CD4+ T cells (B) CD8+ T cells

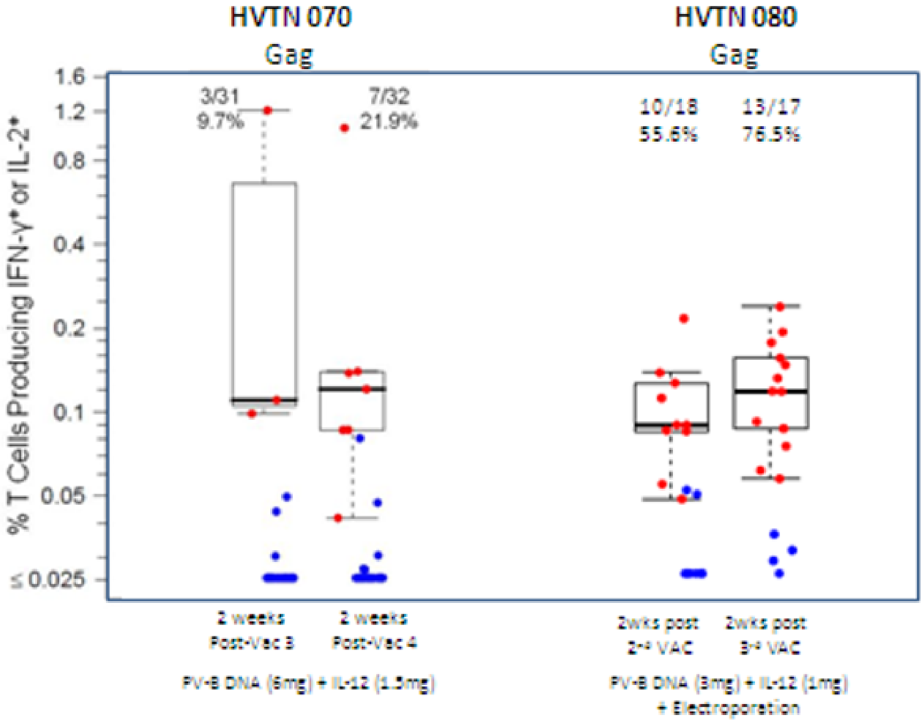

Figure 4-9 HVTN 070 and HVTN 080 ICS responses against Gag global PTE peptides

Neutralizing antibodies against HIV-1 strains Bal.26, MN.3, MW965.26, NPO3.13 and SF162.LS were measured. Low level (titer < 20) positive responses to the MW965.26 isolate were observed in 10 participants. For group 1 receiving PV-B alone the response rate was 60.0% at day 42 and 20.0% at day 98. For the combined groups 2 and 3 receiving PV-B and IL-12 pDNA, the response rate was 20.0% at day 42 and 30.0% at day 98. One placebo recipient had a positive response for MW965.26 at day 98. Four participants (2 from Group 1 and 2 from Group 2) had a positive response at both timepoints. No responses were observed against Bal.26, MN.3, NPO3.13, and SF162.LS viruses.

In summary, PV-B and IL-12 pDNA can be safely administered with IM injection and EP using the VGX CELLECTRA® EP system. IM vaccination with EP is adequately tolerated by participants although it is associated with a moderate amount of transient discomfort. Electroporation significantly enhances the cellular immunogenicity of DNA, and in this study allowed detection of a significant adjuvant effect of IL-12 pDNA affecting the frequency of CD4+ and CD8+ T cell responses to be measured for the first time. Electroporation technology has the potential to change the prospects for DNA vaccines.

4.10.3 Clinical studies of the proposed EP device

A study of the ADVAX clade B'/C HIV-1 plasmid DNA vaccine (*gag, pol, env, nef-tat*) delivered by EP using the TDS device or by conventional IM administration was conducted at the Rockefeller University Hospital.[33] The DNA vaccine candidate was developed by the Aaron Diamond AIDS Research Center (ADARC) with the

International AIDS Vaccine Initiative (IAVI), and the trial is funded by a Collaboration for AIDS Vaccine Discovery (CAVD) grant to ADARC.[34] The study was a randomized, study in 40 HIV uninfected subjects including the following groups:

- ADVAX + TDS-EP (3 dose groups – 0.2, 1.0, 4.0 mg)
- ADVAX alone (single dose group – 4.0 mg)
- Placebo + TDS-EP

The study was designed to compare the safety, tolerability, and immunogenicity of ADVAX following administration via either the TDS EP delivery device or conventional IM injection. A total of 24 volunteers received ADVAX by EP at one of the three dosage levels (0.2 mg, 1.0 mg or 4.0 mg) another 8 by conventional IM injection at the 4.0 mg dose level, and another 8 were given a placebo via the TDS device. The subjects received two administrations at study weeks 0 and 8. Following a review of the interim data, the protocol was amended to allow for a third administration in the 4.0 mg EP dose group and corresponding placebo subjects at study week 36 (n=8 for 4.0 mg ADVAX + TDS EP, n=3 for placebo).

Adverse Events

ADVAX delivered IM or EP was safe and reasonably well-tolerated, although most volunteers in all dose groups reported local pain and/or tenderness ranging from mild to moderate severity. The proportion of volunteers with mild to moderate local pain and/or tenderness was lower in the 4mg IM group (2/8) than in the EP groups (6/8 EP-placebo and 8/8 each ADVAX EP group). However, at four days post administration, there was no significant difference in local reactogenicity between any of the groups. A majority of local reactions resolved within one day; all resolved within 7 days. The maximum severity of systemic reactogenicity events within 4 days after any ADVAX or placebo administration was moderate. All systemic reactions resolved within 2 days. There were no observed differences in the frequency or systemic reactogenicity between the study groups.

Overall, the vast majority of adverse events observed during the study were mild. No volunteers discontinued the study due to adverse events. None of the moderate adverse events were related to the vaccine candidate or administration procedure, and the only serious adverse event occurring during the study (hospitalization for coronary artery disease), was also unrelated to either the vaccine candidate or delivery procedure. No differences in clinical laboratory parameters were observed between study groups.

Tolerability

Tolerability assessments were performed to assess the tolerability of the EP procedure. This included questionnaire based assessments of pain immediately after injection, immediately after EP administration, and at 30 minutes after EP administration. The intensity of pain was greatest immediately after electrical stimulation of the muscle, decreasing significantly by 30 minutes post administration (see Figure 4-10). There were no differences in the level of discomfort reported among any of the four groups administered EP. The level of tolerability was independent of age, gender, body weight, skin fold thickness, or administration in dominant versus non-dominant arm. The

majority of subjects expressed a willingness to undergo the procedure to protect against a serious disease where no vaccine is currently available, such as HIV (96%), or to improve protection against a disease where a vaccine is already available, such as influenza (85%).

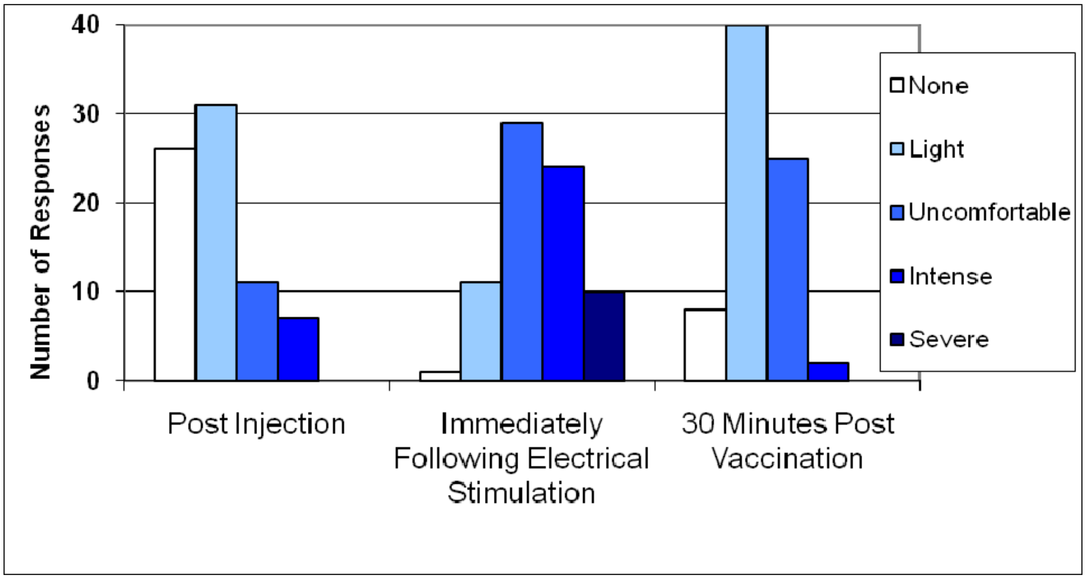

Figure 4-10 Tolerability of ADVAX-IM vs ADVAX-EP

Immunogenicity

IFN-γ ELISpot response rates were IM: 0/8 (0%), 0.2 mg EP: 1/8 (13%), 1.5 mg EP: 5/8 (63%), and 4.0 mg EP: 7/8 (83%). There were no responses to placebo, by definition. The magnitude of response increased in the EP groups in a dose-dependent manner. The number of antigens to which the response was detected improved with EP and increasing dosage. Responses in the mid and high dose EP groups persisted until the end of the study.

4.11 Potential risks of study products and administration

4.11.1 Multiantigen pDNA HIV (HIV-MAG) vaccine risk

As the vaccine plasmids HIV-1 *gag*/*pol* and HIV-1 *nef*/*tat*/*vif*, *env* are formulated in 0.25% bupivacaine there is a risk of allergic reaction, including rash, urticaria, angioedema, bronchospasm, or anaphylaxis.

Significant adverse experiences including cardiac arrest and death have occurred following intravenous (IV) delivery of bupivacaine. In most cases, this has followed use of bupivacaine at a dose of 1.6 mg/kg. Bupivacaine in this study is being administered IM at a dose of 2.5 mg/ml (maximum 2 ml, or 5 mg). A 50 kg person would receive a dose of 0.1 mg/kg per dose administered.

Participants with a history of allergic reaction to amide-type local anesthetics (bupivacaine [Marcaine], lidocaine [Xylocaine], mepivacaine [Polocaine/Carbocaine], etidocaine [Duranest], prilocaine [Citanest, EMLA® cream]) will be excluded.

4.11.2 *IL-12* pDNA risk

As the *IL-12* plasmid is formulated in 0.25% bupivacaine, there is a risk of allergic reaction, including rash, urticaria, angioedema, bronchospasm, or anaphylaxis.

Significant adverse experiences including cardiac arrest and death have occurred following IV delivery of bupivacaine. In most cases, this has followed use of bupivacaine at a dose of 1.6 mg/kg. Bupivacaine in this study is being administered IM at a dose of 2.5 mg/ml (maximum 2 ml, or 5 mg). A 50 kg person would receive a dose of 0.1 mg/kg per dose administered.

Participants with a history of allergic reaction to amide-type local anesthetics (bupivacaine [Marcaine], lidocaine [Xylocaine]. Mepivacaine [Polocaine/Carbocaine], etidocaine [Duranest], prilocaine [Citanest, EMLA® cream]) will be excluded.

It is possible that administration of *IL-12* DNA might elicit antibody that might affect native IL-12. The study incorporates testing for IL-12 antibody at baseline and postvaccination.

4.11.3 Electroporation risks

Other than transient injection site discomfort, risks of EP have been minimal in prior studies but there is the possibility for electrical injury, disruption of function of implanted electronic medical devices (eg, pacemaker, implantable cardioverter defibrillator), vasovagal reaction, elevated creatine phosphokinase (CPK), or the exacerbation of cardiac arrhythmias. Participants with a history of cardiac arrhythmia (excluding sinus arrhythmia) or certain metal implants will be excluded (see Section 7.2).

4.11.4 VSV HIV *gag* risks

VSV HIV *gag* risks include the possibility of fever, chills, mild flu-like syndrome, arthralgia, vesicular rash, nausea, or dizziness in the first few days following injection. There is a theoretical risk of viral encephalitis or neurological impairment following vaccination with rVSV. This vaccine vector has been specifically attenuated to eliminate neurovirulence. This has been demonstrated in sensitive animal models. However, as a precaution, volunteers may not participate if they have a neurological/neuropsychiatric disorder that could be confused with reactions to the VSV HIV *gag* study product and interfere with the assessment of safety.

There is a hypothetical risk of eliciting autoimmune responses in vaccine recipients. This risk is based on the identification of a peptide octamer and other shorter peptides in the VSV Indiana serotype (VSV_{IN}) N protein that are very similar in sequence to peptides found in Ro60kD, which bind the anti-Ro/SSA antibodies found in sera of patients with systemic lupus erythematosis (SLE) [35]. Antibodies that bind Ro protein can also be elicited in rabbits that are hyper-immunized with purified VSV_{IN} N protein [36]. In addition, antibodies have been detected in SLE patients that cross react with VSV_{IN} M and to a lesser extent N core proteins but at a much lower frequency to the VSV_{IN} G protein [37]. However, it is not clear what these observations mean since antibodies that

react to the peptide octamer in Ro60kD account for only a small minority of the anti-Ro60kD auto antibodies in SLE patients [38]. Also, the detection of antibodies in the sera of SLE patients that react with VSV_{IN} M and N proteins but not with G protein is puzzling, because the VSV G protein has strong antigenic properties and typically elicits a very robust and durable humoral immune response [39]. Furthermore, the epidemiology of SLE does not support a specific role for VSV_{IN} as a causative agent, since VSV_{IN} is found only in the Americas and the frequency of SLE in the United kingdom (UK) and the USA is very similar [40]. Based on the available published literature, there is currently no convincing evidence that infection with VSV_{IN} leads to the development of SLE.

Table 4-11 Summary of potential risks of study products and administration

Common	Mild to moderate injection site pain, tenderness, erythema, or swelling/induration/edema<br>Malaise/fatigue, myalgia, or headache in the first few days following injection<br>A vaccine-induced positive HIV antibody test result
Less common	Severe injection site pain or tenderness<br>Fever, chills, flu-like syndrome, arthralgia, rash, nausea, or dizziness in the first few days following injection<br>Vasovagal reaction/lightheadedness/dizziness related to the injection procedure<br>Transient changes in clinical laboratory values<br>Injection site hematoma, bruising/ecchymosis, laceration, or bleeding related to the injection procedure
Uncommon or rare	Severe localized injection site reaction, such as sterile abscess or secondary bacterial infection<br>Allergic reaction, including rash, urticaria, angioedema, bronchospasm, or anaphylaxis
Unknown frequency or theoretical risks	Muscle damage at the injection site<br>Autoimmune disease or cancer<br>VSV: Viral encephalitis; neurological impairment<br>Electrical injury with EP<br>Disruption of function of implanted electronic medical devices with EP<br>Exacerbation of cardiac arrhythmias with EP<br>Vesicular rash<br>Effects on a participant’s response to an approved HIV vaccine administered in the future<br>Effects on susceptibility to HIV, if the participant is exposed to HIV<br>Effects on the course of HIV infection/disease, if the participant is infected with HIV<br>Effects on the fetus and on pregnancy

5 Objectives and endpoints

5.1 Primary objectives and endpoints

Primary objective:

To evaluate the safety and tolerability of a prime-boost regimen of HIV-MAG vaccine given with and without plasmid human *IL-12*, delivered with EP, followed by a VSV HIV *gag* boost in healthy HIV-uninfected adult volunteers.

Primary endpoints:

- Frequency and severity of local injection/EP site reactogenicity signs and symptoms: pain, tenderness, erythema, induration, and maximum severity of pain and/or tenderness.
- Frequency and severity of systemic reactogenicity signs and symptoms: fever, malaise/fatigue, myalgia, headache, nausea, vomiting, chills, arthralgia, and maximum severity of systemic symptoms.
- Magnitude of local injection/EP site pain as measured by a visual analog scale.
- Frequency of AEs categorized by MedDRA body system, MedDRA preferred term, severity and assessed relationship to study products. Detailed description of all AEs meeting DAIDS criteria for expedited reporting.
- The distribution of values of safety laboratory measures: white blood cells, neutrophils, lymphocytes, hemoglobin, alkaline phosphatase, platelets, ALT, AST, creatinine, and CPK at baseline and at follow-up visits postvaccination.
- Number of participants with early discontinuation of vaccinations and reason for discontinuation.
- Distribution of responses to questions regarding acceptability of study injections procedures.

5.2 Secondary objectives and endpoints

Secondary objectives 1:

- To determine the optimal tested dose of *IL-12* pDNA to improve the immunogenicity of an HIV-MAG vaccine prime delivered with EP, followed by a VSV HIV *gag* boost in healthy HIV-uninfected adult volunteers
- To evaluate and characterize HIV-1-specific CD4+ and CD8+ lymphocyte responses to an HIV-MAG vaccine prime given with and without *IL-12* pDNA, delivered with EP, followed by a VSV HIV *gag* boost

Secondary endpoints 1*:*

- Response rate of CD4+ T cell responses measured by ICS for IFN-γ and/or IL-2, to HIV PTE peptide pools representing Gag, Pol, Env, Nef, Tat, and Vif
- Response rate of CD8+ T cell responses measured by ICS for IFN-γ and/or IL-2, to HIV PTE peptide pools representing Gag, Pol, Env, Nef, Tat and Vif
- (At the option of the HVTN Laboratory Program) Response rate of T-cell responses as measured by IFN-γ ELISpot

Secondary objective 2:

To evaluate HIV specific humoral immune responses to the vaccine regimens

Secondary endpoint 2*:*

Induction of binding antibodies to HIV-1 *gag* p55 and HIV-1 6101 *env* gp160

5.3 Exploratory objectives

Exploratory objective 1:

To monitor for *IL-12* pDNA adjuvant-induced antibodies to IL-12

Exploratory endpoint 1:

Frequency of *IL-12* pDNA adjuvant-induced antibodies to IL-12

Exploratory objective 2:

To evaluate the innate immune response to the vaccine regimens by identifying vaccine-associated changes in immune cell populations, gene expression and soluble factors in the serum

Exploratory endpoints 2:

- Blood concentrations of dendritic cells, monocytes, granulocytes and lymphocyte populations (T, B, and NK cells)
- Changes in PBMC gene expression relative to pre-vaccine levels of key genes expected to change, such as IP-10 and MCP-1
- Concentrations of cytokines and chemokines (e.g., IFN-γ, IL-6, TNF-α, IL-10, and MCP-1) in serum samples
- NK-cell phenotyping and function by flow cytometry
- B-cell phenotyping by flow cytometry

6 Statistical considerations

This trial is designed to test the safety and immunogenicity of multiantigen HIV-1 DNA (HIV-MAG) vaccine regimens either without plasmid human *IL-12* or with various different doses of plasmid human *IL-12* and delivered with EP, followed by an rVSV vector boost.

IL-12 pDNA has been shown to be safe and well-tolerated up to doses of 1.5 mg IM in other HVTN studies. DNA vaccines have been shown to be safe and well-tolerated in doses up to 8 mg DNA in a single injection [30]. The doses of DNA and *IL-12* pDNA being utilized in this study are below these levels. The total volume of the HIV-MAG vaccine and the *IL-12* pDNA at the highest dose will be slightly less than 2 cc. The vaccine will therefore be administered by two IM injections into the deltoid (one on each arm), and when EP is given, both arms will be subject to the procedure as well.

Results of preclinical immunogenicity studies in macaques indicate that EP delivery of HIV-1 DNA vaccine plus *IL-12* pDNA gave immune responses of superior magnitude at lower doses than the vaccine administered without EP. A major purpose of this trial is to determine if the candidate multiantigen DNA vaccine administered with *IL-12* pDNA and given via EP is safe and immunogenic. This trial also aims to select the *IL-12* pDNA dose that best enhances responses to the HIV-MAG vaccine. Priming effects of DNA vaccination may be best assessed following heterologous boost vaccination. This trial includes a rVSV HIV *gag* vaccine boost to provide additional data for the dose selection of *IL-12* pDNA, as well as first-in-humans safety and immunogenicity information for the pDNA-rVSV prime-boost regimen overall. Initial safety data for the VSV_{IN} HIV *gag* vaccine will first be obtained from the HVTN rVSV phase 1 trial, HVTN 090.

6.1 Accrual and sample size calculations

Recruitment will target 100 healthy, HIV-uninfected adult participants 18-50 years of age. Enrollment will occur in 2 stages as indicated in the Table 3-1 schema footnotes and in Section 11.1.3. See the schema table for the detailed vaccine assignments.

Since enrollment is concurrent with receiving the first study vaccination, all participants will provide some safety data. Hence, sample size calculations for safety in Section 6.1.1 are based on the target sample sizes. It is possible, however, for immunogenicity data to be missing; previous HVTN and AIDS Vaccine Evaluation group (AVEG) studies suggest 10% is a reasonable estimate for the rate of missing data. For this reason, the sample size calculations in Section 6.1.2 account for 10% of enrolled participants having missing data for the major immunogenicity endpoints.

6.1.1 Sample size calculations for safety

The goal of the safety evaluation for this study is to identify safety concerns associated with product administration. Sample size calculations for safety are expressed in terms of the ability to detect SAEs requiring expedited reporting to DAIDS (see Section 11.2.3).

The ability of the study to detect SAEs can be expressed by the true event rate above which at least 1 event would likely be observed and the true event rate below which no events would likely be observed. Specifically, for each vaccine arm of the study in groups

1-4 (n =22), there is a 90% chance of observing at least 1 event if the true rate of such an event is 10.0% or more; and there is a 90% chance of observing no events if the true rate is 0.4% or less. For the vaccine arm in the vaccine/adjuvant regimens (Groups 2-4) combined (n=66), there is a 90% chance of observing at least 1 event if the true rate of such an event is 3.5% or more; and there is a 90% chance of observing no events if the true rate is 0.1% or less. In HVTN vaccine trials from December 2000 through September 2010 about 4% of participants who received placebos experienced an SAE.

Probabilities of observing 0, 1 or more, and 2 or more events among arms of size 22 (Groups 1-4) and 66 (Groups 2-4 combined) are presented in Table 6-1 for a range of possible true adverse event rates. These calculations provide a more complete picture of the sensitivity of this study design to identify potential safety problems with the vaccine.

Table 6-1 Probability of observing 0 events, 1 or more events, and 2 or more events, among arms of size 22 and size 66 for different true event rates

True event rate (%)	Pr(0/22)	Pr(1+/22)	Pr(2+/22)	Pr(0/66)	Pr(1+/66)	Pr(2+/66)
1	80.2	19.8	2.0	51.5	48.5	14.1
3.5	40.7	59.3	21.9	6.8	93.2	74.7
5	32.4	67.6	30.2	3.4	96.6	84.8
10	9.8	90.2	66.1	0.1	99.9	99.2
20	0.7	99.3	95.2	<0.1	>99.9	>99.9
30	<0.1	>99.9	99.6	<0.1	>99.9	>99.9
40	<0.1	>99.9	>99.9	<0.1	>99.9	>99.9

An alternative way of describing the statistical properties of the study design is in terms of the 95% confidence interval for the true rate of an adverse event based on the observed data. Table 6-2 shows the 2-sided 95% confidence intervals for the probability of an event based on a particular observed rate. Calculations are done using the score test method of Agresti and Coull [41]. If none of the 66 participants receiving a vaccine/adjuvant regimen (group 2-4 combined) experience a safety event, the 95% 2-sided upper confidence bound for the true rate of such events in the total vaccinated population is 5.5%. For each individual vaccine arm (Groups 1-4, n = 22), the 2-sided upper confidence bound for this rate is 14.9%.

Table 6-2 Two-sided 95% confidence intervals based on observing a particular rate of safety endpoints for arms of size 22 and size 66 in the study

Observed event rate	Confidence interval (%)
0/22	[0, 14.9]
1/22	[0.8, 21.8]
2/22	[2.5, 27.8]
0/66	[0, 5.5]
1/66	[0.3, 8.1]
2/66	[0.8, 10.4]

6.1.2 Sample size calculations for immunogenicity

The main goals of this trial regarding immunogenicity outcomes involve a preliminary estimation of response rates. The precision with which the true response rate can be estimated from the observed data depends on the true underlying response rate and the sample size. Two-sided 95% confidence intervals for the response rate based on observing a particular rate of responses in the vaccinees is shown in Table 6-3. Calculations are done using the score test method of Agresti and Coull [41]. The n = 20 assumes a 10% loss of data.

Table 6-3 Two-sided 95% confidence intervals for the true response rate based on observing a particular rate of responses in the vaccinees (n = 20)

No. of responses	Observed response rate (%)	Confidence interval
1/ 20	5	[0.9, 23.6]
3/ 20	15	[5.2, 36.0]
5/ 20	25	[11.2, 46.9]
7/ 20	35	[18.1, 56.7]
9/ 20	45	[25.8, 65.8]
11/ 20	55	[34.2, 74.2]
13/ 20	65	[43.3, 81.9]
15/ 20	75	[53.1, 88.8]
17/ 20	85	[64, 94.8]
19/20	95	[76.4, 99.1]

As shown in Table 6-4, there is limited power for a formal comparison of immunogenicity response rates of vaccinees between vaccine groups of size n = 20 and, hence, formal comparisons are not listed in the study objectives. For either 80% or 90% power, the sizes of differences that the trial is powered to detect are fairly large. These calculations use a Fisher's exact 2-sided test with a Type I error rate of 0.05.

Table 6-4 Power for comparison of response rates between 2 arms (n1 = 20, n2 = 20)

True response rate in Arm 1 (%)	Minimum true response rate in Arm 2 (%) in order to detect the difference with 80% power	90% power
10	55	62
20	68	74
30	78	84
40	87	91
50	93	97

An alternative to formal comparisons of arms is to rank the arms by their response rates. For arms of size 20, we can assess the reliability of this study to select the best arm with respect to the magnitude of response rates. Table 6-5 shows various true response rates for which this study with high probability will correctly select the arm with the highest response rate. Each line in the table shows the results based on 40,000 simulated datasets generated using 2 (best/next best) different binomial probabilities for 4 arms of size 20 (Groups 1-4), with the best (highest) response probability used to generate data for one arm and the next best (low) response probability used to generate data for the remaining 3 arms [41]. If the difference in response between the best and next best arms is smaller than the assumed difference, the chance of correctly selecting the arm with the true highest response will be less than 80% (90%).

Table 6-5 True immunogenicity response rates for which the regimen with the highest response probability will be correctly selected with 0.8 (0.9) probability

Second best response probability	Best response probability	Difference
10%	27%(31%)	17%(21%)
20%	40%(46%)	20%(26%)
30%	51%(57%)	21%(27%)
40%	61%(68%)	21%(28%)
50%	71%(77%)	21%(27%)
60%	80%(85%)	20%(25%)
70%	88%(92%)	18%(22%)
80%	95%(98%)	15%(18%)

When the response rates of two arms are similar, their response magnitudes will be compared. The response magnitude is measured by the percent of CD4+ cells responding to IL2/IFN-γ to any antigen measured by ICS. (Note that CD8+ response rate is generally low in DNA vaccine products tested so far. A reliable estimate of mean and standard deviation of response magnitude cannot be obtained from previous trials. Our power evaluations are done solely based on CD4+ response magnitudes.) We used CD4+ response magnitudes measured from 9 responders out of 21 participants who were assigned in HIV-1 DNA plasmid vaccine VRC-HIVDNA009-00-VP group of HVTN 068 at visit 8 (two weeks after the second vaccination) to estimate the mean and standard deviation among pDNA vaccine recipients in Group 5. The log-transformed response magnitudes are assumed to be normally distributed. The mean and standard deviation are -0.75 and 0.23, respectively. The same standard deviation is assumed for other vaccine

groups. Table 6-6 presents the minimum detectable difference in mean $\log_{10}$ response magnitudes between the two arms for 80% and 90% power based on normal two-sided test with a Type I error rate of 0.05, given each common true response rate.

Table 6-6 Minimum detectable difference in mean $\log_{10}$ response magnitude between two arms when their response rates are similar (n1=20, n2=20)

Common true response rate (%)	Minimum detectable difference in mean $\log_{10}$ response magnitude		Minimum detectable difference in mean $\log_{10}$ response magnitude relative to standard deviation	
	Power=80%	Power=90%	Power=80%	Power=90%
50	0.368	0.437	1.6	1.9
60	0.322	0.391	1.4	1.7
70	0.299	0.345	1.3	1.5
80	0.276	0.322	1.2	1.4
90	0.253	0.299	1.1	1.3

If the response rates between the best and the second best are different but the difference is less the difference presented in Table 6-5 to distinguish them by their response rate alone, the response rate and magnitude are compared jointly using Lachenbruch's test statistics [42]. Table 6-7 presents the minimum detectable difference in mean $\log_{10}$ response magnitude between the arms with the best and the second best response rate assuming that the difference of two response rates is 10%.

Table 6-7 Minimum detectable difference in mean $\log_{10}$ responses magnitude (n1=20, n2=20)

True response rate (%) in Arm 1	True response rate (%) in Arm 2	Minimum detectable difference in mean log10 response magnitude		Minimum detectable difference in mean $\log_{10}$ response magnitude relative to standard deviation	
		Power=80%	Power=90%	Power=80%	Power=90%
50	60	0.345	0.414	1.5	1.8
60	70	0.322	0.368	1.4	1.6
70	80	0.276	0.322	1.2	1.4
80	90	0.253	0.299	1.1	1.3

6.2 Randomization

Groups 1, 2, and 3 will be opened to enrollment simultaneously. Groups 1 and 3 share the same visit schedule, with more frequent visits for evaluation of innate immune responses to the vaccines, and will be randomized together. Group 2 will be randomized separately, so that participants who are not available for the frequent visits required for Groups 1 and 3 may also participate. Group 4, which tests the highest dose of IL-12 pDNA, will be enrolled and randomized after safety data through day 14 has been reviewed from at least 24 participants enrolled in Groups 1 and 3 (12 in each group), and all 25 participants in Group 2. In this way, participants will usually have a choice of frequent visits in Groups 1 and 3, or less frequent study visits in Group 2 or 4.

6.3 Blinding

Participants and site staff (except for site pharmacists) will be blinded as to participant treatment arm assignments (eg, active vaccine or control) but not to group assignment. Study product assignments are accessible to those HVTN CRS pharmacists, DAIDS protocol pharmacists and contract monitors, and SDMC staff who are required to know this information in order to ensure proper trial conduct. Any discussion of study product assignment between pharmacy staff and any other HVTN CRS staff is prohibited. The HVTN SMB members also are unblinded to treatment assignment in order to conduct review of trial safety.

When a participant leaves the trial prior to study completion, the participant will be told he or she must wait until the all participants are unblinded to learn his or her treatment assignment.

Emergency unblinding decisions will be made by the site investigator. If time permits, the HVTN PSRT should be consulted before emergency unblinding occurs.

6.4 Statistical analysis

All data from enrolled participants will be analyzed according to the initial randomization assignment regardless of how many vaccinations they received. Since enrollment is concurrent with receiving the first vaccination, all participants will have received at least 1 vaccination and therefore will provide some safety data. The analysis is a modified intent-to-treat analysis in that individuals who are randomized but not enrolled do not contribute data and hence are excluded. Because of blinding and the brief length of time between randomization and enrollment—typically no more than 4 working days—very few such individuals are expected.

Analyses for primary endpoints will be performed using SAS and R. All other descriptive and inferential statistical analyses will be performed using SAS, StatXact, or R statistical software.

No formal multiple comparison adjustments will be employed for multiple safety endpoints, multiple primary immunogenicity endpoints, or secondary endpoints.

6.4.1 Analysis variables

The analysis variables consist of baseline participant characteristic, safety, and immunogenicity for primary- and secondary-objective analyses.

6.4.2 Baseline comparability

Treatment arms will be compared for baseline participant characteristics using descriptive statistics.

6.4.3 Safety/tolerability analysis

Since enrollment is concurrent with receiving the first vaccination, all participants will have received at least 1 vaccination and therefore will provide some safety data.

Reactogenicity

The number and percentage of participants experiencing each type of local and systemic reactogenicity sign or symptom will be tabulated by severity and treatment arm and the percentages displayed graphically by arm. For a given sign or symptom, each participant's reactogenicity will be counted once under the maximum severity for all injection visits. In addition, to the individual types of events, the maximum severity of local pain or tenderness, induration or erythema, and of systemic symptoms will be calculated. Kruskal-Wallis tests will be used to test for differences in severity between arms.

Pain assessment

The magnitude of local injection/EP site pain as measured by a visual analog scale will be plotted by time (immediately, 5 minutes, and 25 minutes post vaccination) at each visit and by treatment arm. The mean value for treatment arm and visit will be superimposed on the graph.

AEs

AEs will be summarized using MedDRA body system and preferred terms. Tables will show by treatment arm the number and percentage of participants experiencing an AE within a body system or within preferred term category by severity or by relationship to study product. For the calculations in these tables, a participant with multiple AEs within a category will be counted once under the maximum severity or the strongest recorded causal relationship to study product. Formal statistical testing comparing arms is not planned since interpretation of differences must rely heavily upon clinical judgment.

A listing of SAEs reported to the DAIDS Regulatory Support Center (RSC) Safety Office will provide details of the events including severity, relationship to study product, time between onset and last vaccination, and number of vaccinations received. A separate listing will do the same for AEs of special interest (AESI). AESI for this protocol include but are not limited to autoimmune disorders; a sample list of AESI is provided in Appendix J.

Local laboratory values

Boxplots of local laboratory values will be generated for baseline values and for values measured during the course of the study by treatment arm and visit. Each boxplot will show the first quartile, the median, and the third quartile. Outliers (values outside the boxplot) will also be plotted. If appropriate, horizontal lines representing boundaries for abnormal values will be plotted.

For each local laboratory measure, summary statistics will also be presented by treatment arm and timepoint, as well as changes from baseline for post-enrollment values. In addition, the number (percentage) of participants with local laboratory values recorded as meeting Grade 1 AE criteria or above as specified in the DAIDS AE Grading Table (see Section 9.10) will be tabulated by treatment arm for each post-vaccination timepoint. Clinical laboratory abnormalities without an associated clinical diagnosis will also be reported as AEs and will be included in the tabulation of AEs described above.

Reasons for vaccination discontinuation and early study termination

The number and percentage of participants who discontinue vaccination and who terminate the study early will be tabulated by reason and treatment arm.

Acceptability

The number and percentage of responses to the acceptability questions will be tabulated by vaccination time, treatment arm, and overall.

6.4.4 Immunogenicity analysis

For the statistical analysis of immunogenicity endpoints, data from enrolled participants will be used according to the initial randomization assignment regardless of how many injections they received. Additional analyses may be performed limited to participants who received all scheduled injections per protocol. Assay results that are unreliable, from specimens collected outside of the visit window, or from HIV-infected participants postinfection are excluded. Since the exact date of HIV infection is unknown, any assay data from blood draws 4 weeks prior to an infected participant's last seronegative sample and thereafter may be excluded. If an HIV-infected participant does not have a seronegative sample postenrollment, then all data from that participant may be excluded from the analysis.

Qualitative assay data (eg, IFN-γ and/or IL-2 response as measured by ICS or ELISpot response rates) will be analyzed by tabulating the frequency of positive response for each assay by antigen and treatment arm at each timepoint at which an assessment is performed. Crude response rates will be presented with their corresponding 95% confidence interval estimates calculated using the score test method of Agresti and Coull [41]. Because of the small numbers of control participants in each group, no adjustment will be made to the vaccine arm estimates for the false positive rates in the control arms. Fisher's exact tests will be used to compare the response rates of any 2 vaccine arms, with a significant difference declared if the 2-sided p-value is ≤ 0.05.

In addition to response rate estimates for each timepoint, the probability of observing at least 1 positive response by a given timepoint and the probability of observing more than 1 positive response by a given timepoint will be estimated, with corresponding confidence intervals, for each vaccine arm using maximum likelihood-based methods [43].

For continuous assay data (eg, number of spot forming cells from ELISpot assay or percentage of positive cells from ICS assay) graphical and tabular summaries of the distributions by antigen, treatment arm, and timepoint will be made. The difference between two arms at a specific timepoint will be tested with a nonparametric Wilcoxon rank sum test if the data are not normally distributed and with a 2-sample t-test if the data appear to be normally distributed. An appropriate data transformation (eg, $\log_{10}$ transformation) may be applied to better satisfy assumptions of symmetry and homoscedasticity (constant variance).

More sophisticated analyses employing repeated measures methodology (for example, repeated measures analysis of variance [ANOVA] or generalized estimating equations) may be utilized to incorporate immune responses over several timepoints. However,

inference from such analyses would be limited by the small sample size of this study. All statistical tests will be 2-sided and will be considered statistically significant if $p \leq 0.05$.

Some immunologic assays that have underlying continuous or count-type readout are dichotomized into responder/nonresponder categories (eg, ELISpot assay and ICS assay). If treatment arm differences for these assays are best summarized by a mixture model, then Lachenbruch's test statistic [44] will be used to evaluate the composite null hypothesis of equal response rates in the 2 arms and equal response distributions among responders in the 2 such arms. This test statistic equals the square of a binomial Z-statistic for comparing the response rates plus the square of a Wilcoxon statistic for comparing the response distributions in the subgroup of responders. A permutation procedure is used to obtain a 2-sided p-value.

6.4.4.1 Missing data

Based upon previous AVEG and HVTN trials, missing 10% of immunogenicity results for a specific assay is common due to study participants terminating from the study early, problems in shipping specimens, or low cell viability of processed PBMC. To achieve unbiased statistical estimation and inferences with nonparametric tests and generalized linear models fit by generalized estimating equation (GEE) methods, missing data are assumed to be missing completely at random (MCAR). MCAR assumes that the probability of an observation being missing does not depend upon the observed responses or upon any unobserved covariates but may depend upon observed covariates (eg, missing more among whites than nonwhites). When missing data are minimal (specifically if no more than 20% of participants are missing any values), then nonparametric tests and GEE methods will be used, because violations of the MCAR assumption will have little impact on the estimates and hypothesis tests. These models will include as covariates all available baseline predictors of the missing outcomes.

If a substantial amount of immunogenicity data are missing (at least 1 value missing from more than 20% of participants), then using the methods that require the MCAR assumption may give misleading results. In this situation, analyses of the immunogenicity endpoints at a specific timepoint will be performed using parametric generalized linear models fit by maximum likelihood. These methods provide unbiased estimation and inferences under the parametric modeling assumptions and the assumption that the missing data are missing at random (MAR). MAR assumes that the probability of an observation being missing depends upon the observed responses and upon observed covariates, but not upon any unobserved factors. Generalized linear models for response rates will use a binomial error distribution and for quantitative endpoints, a normal error distribution. For assessing repeated immunogenicity measurement, linear mixed effects models will be used. If the immunological outcomes are left- and/or right- censored, then the linear mixed effects models of Hughes [45] will be used, because they accommodate the censoring. In addition, secondary analyses of repeated immunogenicity measurements will be done using weighted GEE [46] methods, which are valid under MAR. All of the models described above will include as covariates all available baseline predictors of the missing outcomes.

6.4.5 Analyses prior to end of study

Safety

During the course of the trial, unblinded analyses of safety data will be prepared approximately every 4 months for review by the SMB. Unblinded ad hoc safety reports may also be prepared for SMB review at the request of the PSRT. The HVTN leadership must approve any other requests for unblinded safety data prior to the end of the study.

Immunogenicity

An unblinded statistical analysis by treatment assignment of an immunogenicity endpoint may be performed when the Laboratory Program has completed testing at least 80% of samples from the primary immunogenicity visit and all participants have completed the visit. The Laboratory Program will review the analysis report prior to distribution to the protocol chairs, DAIDS, vaccine developer, and other key HVTN members and investigators. Distribution will be limited to those with a need to know for the purpose of informing future trial-related decisions. The HVTN leadership must approve any other requests for HVTN immunogenicity analyses prior to the end of the study. Any analyses conducted prior to the end of the study should not compromise the integrity of the trial in terms of participant retention or safety or immunogenicity endpoint assessments.

7 Selection and withdrawal of participants

Participants will be healthy, HIV-1–uninfected (seronegative) adults who comprehend the purpose of the study and have provided written informed consent. Volunteers will be recruited and screened; those determined to be eligible, based on the inclusion and exclusion criteria, will be enrolled in the study. Final eligibility determination will depend on results of laboratory tests, medical history, physical examinations, and answers to self-administered and/or interview questions.

Investigators should always use good clinical judgment in considering a volunteer's overall fitness for trial participation. Some volunteers may not be appropriate for enrollment even if they meet all inclusion/exclusion criteria. Medical, psychiatric, occupational, or other conditions may make evaluation of safety and/or immunogenicity difficult, and some volunteers may be poor candidates for retention.

An rVSV vector will be given to participants in this trial. This vaccine vector has been specifically attenuated to eliminate neurovirulence. This has been demonstrated in sensitive animal models. However, as a precaution, volunteers may not participate if they have a neurological/neuropsychiatric disorder that could be confused with reactions to the VSV HIV *gag* study product and interfere with the assessment of safety.

As the DNA vaccine and adjuvant used in this study will be given by EP, volunteers may not participate if they have certain metal implants, a surgical or traumatic metal implant in the upper limb and or torso, or a history of cardiac arrhythmias.

Determination of eligibility, taking into account all inclusion and exclusion criteria, must be made within 56 days prior to enrollment unless otherwise noted in Sections 7.1 and 7.2.

7.1 Inclusion criteria

1. **Age** of 18 to 50 years

2. **Access to a participating HVTN CRS** and willingness to be followed for the planned duration of the study

3. Ability and willingness to provide **informed consent**

4. **Assessment of understanding:** volunteer demonstrates understanding of this study and completes a questionnaire prior to first vaccination with verbal demonstration of understanding of all questionnaire items answered incorrectly

5. Willingness to receive **HIV test results**

6. Willingness to discuss **HIV infection risks**, amenable to **HIV risk reduction counseling,** and committed to maintaining behavior consistent with low risk of HIV exposure through the last required protocol clinic visit

7. **Willing to be contacted annually** after completion of scheduled clinic visits for a total of 3 years following initial study injection.

8. **Agrees not to enroll in another study** of an investigational research agent prior to completion of last required protocol clinic visit (excludes annual contacts for safety surveillance)

9. **Good general health** as shown by medical history, physical exam, and screening laboratory tests

10. Assessed by the clinic staff as being at **"low risk" for HIV infection**.

11. **Hemoglobin** ≥ 11.0 g/dL for volunteers who were born female, ≥ 13.0 g/dL for volunteers who were born male

12. **White blood cell (WBC) count** = 3300 to 12,000 cells/mm^3

13. **Total lymphocyte count** ≥ 800 cells/mm^3

14. **Remaining differential** either within institutional normal range or with site physician approval

15. **Platelets** = 125,000 to 550,000/mm^3

16. **Chemistry panel**: ALT, AST, alkaline phosphatase, and creatinine values ≤ institutional upper limits of normal; CPK ≤ 2.0 times the institutional upper limit of normal.

17. **Negative HIV-1 and -2 blood test:** US participants must have a negative FDA-approved immunoassay

18. Negative Hepatitis B surface antigen (HBsAg)

19. **Negative anti-Hepatitis C virus antibodies** (anti-HCV), or negative HCV PCR if the anti-HCV is positive

20. Normal urine:

    - Negative urine glucose, and
    - Negative or trace urine protein, and
    - Negative or trace urine hemoglobin (if trace hemoglobin is present on dipstick, a microscopic urinalysis within institutional normal range).

21. **Volunteers who were born female**: negative serum or urine beta human chorionic gonadotropin (β-HCG) pregnancy test performed prior to vaccination on the day of initial vaccination

22. **Reproductive status**: A volunteer who was born female must:

    - Agree to consistently use effective contraception (see Appendix B) for sexual activity that could lead to pregnancy from at least 21 days prior to enrollment

through the last required protocol clinic visit. Effective contraception is defined as using any of the following methods:

- Condoms (male or female) with or without a spermicide,
- Diaphragm or cervical cap with spermicide,
- Intrauterine device (IUD),
- Hormonal contraception, or
- Successful vasectomy in the male partner (considered successful if a volunteer reports that a male partner has [1] documentation of azoospermia by microscopy, or [2] a vasectomy more than 2 years ago with no resultant pregnancy despite sexual activity postvasectomy);

- Or not be of reproductive potential, such as having reached menopause (no menses for 1 year) or having undergone hysterectomy, bilateral oophorectomy, or tubal ligation;

- Or be sexually abstinent.

23. **Volunteers who were born female must also agree not to seek pregnancy through alternative methods,** such as artificial insemination or in vitro fertilization until after the last required protocol clinic visit

7.2 Exclusion criteria

1. **Allergy to amide-type local anesthetics** (bupivacaine [Marcaine], lidocaine [Xylocaine], mepivacaine [Polocaine/Carbocaine], etidocaine [Duranest], prilocaine [Citanest, EMLA® cream])

2. **Presence of implanted electronic medical device** (eg, pacemaker, implantable cardioverter defibrillator)

3. **Presence of surgical or traumatic metal implant** in the upper limb and/or upper torso

4. **Sinus bradycardia (defined as < 50 bpm on exam) or a history of cardiac arrhythmia**: eg, supraventricular tachycardia, atrial fibrillation, or frequent ectopy

5. **Neurological or neuropsychiatric disorder** that may interfere with the assessment of safety: eg, frequent recurring headaches (ie a pattern of > 1 headache per month affecting activities of daily living (ADLs)/work, frequent or severe/complicated migraines, cluster headaches), a chronic pain syndrome, dizziness, history of meningitis or encephalitis, cranial/spinal/peripheral neuropathy, limb weakness or paralysis, movement disorder, narcolepsy, stroke with sequelae, moderate/severe major depressive disorder, moderate /severe bipolar disorder

6. **Seizure disorder**

7. **Untreated or incompletely treated syphilis infection**

8. **HIV vaccine(s)** received in a prior HIV vaccine trial. For potential participants who have received control/placebo in an HIV vaccine trial, the HVTN 087 PSRT will determine eligibility on a case-by-case basis.

9. **Non-HIV experimental vaccine(s)** received within the last 5 years in a prior vaccine trial. Exceptions may be made for vaccines that have subsequently undergone licensure by the FDA. For potential participants who have received control/placebo in an experimental vaccine trial, the HVTN 087 PSRT will determine eligibility on a case-by-case basis. For potential participants who have received an experimental vaccine(s) greater than 5 years ago, eligibility for enrollment will be determined by the PSRT on a case-by-case basis.

10. **Immunosuppressive medications** received within 168 days before first vaccination. (Not excluded: [1] corticosteroid nasal spray for allergic rhinitis; [2] topical corticosteroids for mild, uncomplicated dermatitis; or [3] oral/parenteral corticosteroids given for non-chronic conditions not expected to recur [length of therapy 10 days or less with completion at least 30 days prior to enrollment].)

11. **Blood products** received within 120 days before first vaccination

12. **Immunoglobulin** received within 60 days before first vaccination

13. **Live attenuated vaccines** other than influenza vaccine received within 30 days before first vaccination or scheduled within 14 days after injection (eg, measles, mumps, and rubella [MMR]; oral polio vaccine [OPV]; varicella; yellow fever)

14. **Investigational research agents** received within 30 days before first vaccination

15. **Intent to participate in another study** of an investigational research agent during the planned duration of the HVTN 087 study

16. **Influenza vaccine or any vaccines that are not live attenuated vaccines** and were received within 14 days prior to first vaccination (eg, tetanus, pneumococcal, Hepatitis A or B)

17. **Allergy treatment with antigen injections** within 30 days before first vaccination or that are scheduled within 14 days after first vaccination

18. **Current anti-tuberculosis (TB) prophylaxis or therapy**

19. **Clinically significant medical condition,** physical examination findings, clinically significant abnormal laboratory results, or past medical history with clinically significant implications for current health. A clinically significant condition or process includes but is not limited to:

    - A process that would affect the immune response,

    - A process that would require medication that affects the immune response,

    - Any contraindication to repeated injections or blood draws,

- A condition that requires active medical intervention or monitoring to avert grave danger to the participant's health or well-being during the study period,
- A condition or process for which signs or symptoms could be confused with reactions to vaccine, or
- Any condition specifically listed among the exclusion criteria below.

20. **Any medical, psychiatric, occupational, or other condition** that, in the judgment of the investigator, would interfere with, or serve as a contraindication to, protocol adherence, assessment of safety or reactogenicity, or a participant's ability to give informed consent

21. **Serious adverse reactions to vaccines** including anaphylaxis and related symptoms such as hives, respiratory difficulty, angioedema, and/or abdominal pain. (Not excluded: a participant who had a nonanaphylactic adverse reaction to pertussis vaccine as a child.)

22. **Autoimmune disease**

23. **Immunodeficiency**

24. **Asthma** other than mild, well-controlled asthma. Exclude a participant who:
    - Generally uses a bronchodilator (beta$_2$ agonist) daily, or
    - In the past year, has (any of the following):
        - Had > 1 exacerbation of symptoms treated with oral steroids (Note: oral/parenteral steroid use for asthma is exclusionary within 168 days before first vaccination.);
        - Routinely used moderate to high dose inhaled corticosteroids (eg, more than the equivalent of 250 mcg fluticasone; 400 mcg budesonide; 500 mcg beclomethasone; or 1000 mcg triamcinolone/flunisolide, as a daily dose) or theophylline for asthma; or
        - Needed emergency care, urgent care, hospitalization, or intubation for asthma.

25. **Diabetes mellitus** type 1 or type 2, including cases controlled with diet alone. (Not excluded: history of isolated gestational diabetes.)

26. **Thyroidectomy, or thyroid disease** requiring medication during the last 12 months

27. History of hereditary **angioedema**, acquired angioedema, or idiopathic angioedema

28. **Hypertension:**
    - If a person has been found to have elevated blood pressure or hypertension during screening or previously, exclude for blood pressure that is not well controlled. Well-controlled blood pressure is defined as consistently ≤ 140 mm Hg systolic and ≤ 90 mm Hg diastolic, with or without medication, with only

isolated, brief instances of higher readings, which must be ≤ 150 mm Hg systolic and ≤ 100 mm Hg diastolic. For these participants, blood pressure must be ≤ 140 mm Hg systolic and ≤ 90 mm Hg diastolic at enrollment.

- If a person has NOT been found to have elevated blood pressure or hypertension during screening or previously, exclude for systolic blood pressure ≥ 150 mm Hg at enrollment or diastolic blood pressure ≥ 100 mm Hg at enrollment.

29. **Body mass index (BMI)** ≥ 40; or BMI ≥ 35 with 2 or more of the following: age > 45, systolic blood pressure > 140 mm Hg, diastolic blood pressure > 90 mm Hg, current smoker, known hyperlipidemia

30. **Deltoid skin fold measurement by caliper > 40 mm**

31. **Bleeding disorder** diagnosed by a doctor (eg, factor deficiency, coagulopathy, or platelet disorder requiring special precautions)

32. **Malignancy** (Not excluded: a participant with a surgical excision and subsequent observation period that in the investigator's estimation has a reasonable assurance of sustained cure or is unlikely to recur during the period of the study.)

33. **Asplenia**: any condition resulting in the absence of a functional spleen

34. **Psychiatric condition** that precludes compliance with the protocol. Specifically excluded are persons with psychoses within the past 3 years, ongoing risk for suicide, or history of suicide attempt or gesture within the past 3 years.

35. **Pregnant or breastfeeding**

7.3 Participant departure from vaccination schedule or withdrawal

This section concerns an individual participant's departure from the vaccination schedule. Pause rules for the trial as a whole are described in Section 11.4.

7.3.1 EP device applied without vaccination

At the month 0 visit, if the Ichor TDS EP device is applied to a participant's arm, and the needle/electrodes contact the participant, but for any reason the participant does not receive any injection of HIV-MAG +/- p*IL-12* or placebo at that visit, then this device-only participant is not considered to be enrolled in the study. Clinic procedures and safety reporting requirements for device-only participants are specified in Section 9.3.

At other visits, if the Ichor TDS EP device is applied to a participant's arm but for any reason the participant does not receive any injection of HIV-MAG +/- p*IL-12* or placebo within the specified visit window period, then the participant will have missed the vaccination (see Section 9.3.1). *Note: A person who receives 1 out of 2 injections at a vaccination visit has not missed the vaccination.*

7.3.2 Delaying vaccinations for a participant

Under certain circumstances, a participant's scheduled vaccination will be delayed. The factors to be considered in such a decision include but are not limited to the following:

- Live attenuated vaccines other than influenza vaccine received within 30 days prior to any vaccination,
- Influenza vaccine or any vaccines that are not live attenuated vaccines (eg, pneumococcal) and were received within 14 days prior to any vaccination,
- Allergy treatment with antigen injections within 30 days prior to any study injection,
- Blood products or immunoglobulin received within 45 days prior to any vaccination, or
- Prevaccination abnormal vital signs or clinical symptoms that may mask assessment of vaccine reaction.

Vaccinations should not be administered outside the visit window period specified in the *HVTN 087 Study Specific Procedures*.

In order to avoid vaccination delays and missed vaccinations, participants who plan to receive licensed vaccines, allergy treatments, or other investigational research agents should be counseled to schedule receipt of these substances outside the intervals indicated above, when possible. Because their effects on safety and immunogenicity assessments and their interactions with study vaccines are unknown, if circumstances allow, these substances should also be avoided in the 2-week interval between a study vaccination and completion of the next scheduled postvaccination follow-up visit.

7.3.3 Participant departure from vaccination schedule

Every effort should be made to follow the vaccination schedule per the protocol. If a participant misses a vaccination and the visit window period for the vaccination has passed, that vaccination cannot be given. The participant should be asked to continue study visits. The participant should resume the vaccination schedule with the next vaccination unless there are circumstances that require further delay or permanent discontinuation of vaccination (see Sections 7.3.2 and 7.3.4).

If a participant receives only one of the two injections(i.e. a half-dose of vaccine/placebo), the Principal Investigator or designated physician will notify the PSRT, and provide a recommendation regarding whether the vaccination schedule can be resumed with the next scheduled injection.

7.3.4 Discontinuing vaccination for a participant

Under certain circumstances, an individual participant's vaccinations will be permanently discontinued. Specific events that will result in stopping a participant's vaccination schedule include:

- Co-enrollment in a study with an investigational research agent (rare exceptions allowing for the continuation of vaccinations may be granted with the unanimous consent of the PSRT)

- Clinically significant condition (ie, a condition that affects the immune system or for which continued vaccinations and/or blood draws may pose additional risk), including but not limited to the following:
  - Pregnancy (regardless of outcome);
  - Any grade 4 local or systemic reactogenicity symptom, lab abnormality, or AE that is subsequently considered to be related to vaccination;
  - Any grade 3 lab abnormality or other clinical AE (exception: fever or vomiting and subjective local and systemic symptoms) that is subsequently considered to be related to vaccination; or
  - Clinically significant type 1 hypersensitivity reaction associated with study vaccination. Consultation with the HVTN 087 PSRT is required prior to subsequent vaccinations following any type 1 hypersensitivity reaction associated with study vaccination; or

- Investigator determination in consultation with Protocol Team leadership (eg, for repeated nonadherence to study staff instructions).

- Participant misses more than 1 vaccination (see Section 7.3.3).

Such participants should be counseled on the importance of continuing with the study and strongly encouraged to participate in follow-up visits and protocol-related procedures (unless medically contraindicated) per the protocol for the remainder of the trial.

In addition, vaccinations will be stopped for participants diagnosed with HIV infection. HIV-infected participants will not continue in the trial (see Sections 7.3.5 and 9.7.1).

7.3.5 Participant termination from the study

Under certain circumstances, an individual participant may be terminated from participation in this study. Specific events that will result in early termination include:

- Participant refuses further participation,

- Participant relocates and remote follow-up or transfer to another HVTN CRS is not possible,

- HVTN CRS determines that the participant is lost to follow-up,

- Participant becomes HIV infected, or

- Investigator decides, in consultation with Protocol Team leadership, to terminate participation (eg, if participant exhibits inappropriate behavior toward clinic staff).

8 Study product preparation and administration

CRS pharmacists should consult the *Pharmacy Guidelines and Instructions for DAIDS Clinical Trials Networks* for standard pharmacy operations. The protocol schema is shown in Table 3-1. See the Investigator's Brochures for further information about study products. The actual dose of VSV IN HIV gag to be administered is listed in italics below each treatment group, in the CoA and in Table 3-1. The syringes will be labeled with the actual dose (see Section 8.3)

8.1 Vaccine regimen

The schedule of vaccination is shown in Section 3 and additional information is given below.

Group 1

Treatment 1 (T1): ProfectusVax DNA Plasmid 1.5 mg (HIV-1 gag/pol 0.75 mg admixed with HIV-1 nef/tat/vif, env 0.75 mg) administered as 0.5 mL IM in left deltoid **and** ProfectusVax DNA Plasmid 1.5 mg (HIV-1 gag/pol 0.75 mg admixed with HIV-1 nef/tat/vif, env 0.75 mg) administered as 0.5 mL IM in right deltoid at Months 0, 1, and 3 using the Ichor Medical Systems TriGrid™ Delivery System (TDS) electroporation (EP) device.

THEN

VSV IN HIV gag $1x10^8$ PFU† administered as $5x10^7$ PFU in 1 mL IM in left deltoid **and** $5x10^7$ PFU in 1 mL IM in right deltoid at Month 6.*

**Nominal dose (per label) – actual dose is 3.4 x 10^7 PFU given as 1.7 $x10^7$PFU in each deltoid*

†Safety data from HVTN 090 will be evaluated by the HVTN 087 PSRT prior to administration of VSV IN HIV gag boosting in HVTN 087. Any change in dose of VSV IN HIV gag would be specified by a protocol modification.

Control 1 (C1): Placebo for ProfectusVax DNA Plasmid (Sodium Chloride for Injection, USP 0.9%) administered as 0.5 mL IM in left deltoid **and** Placebo for ProfectusVax DNA Plasmid (Sodium Chloride for Injection, USP 0.9%) administered as 0.5 mL IM in right deltoid at Months 0, 1, and 3 using the Ichor Medical Systems TDS EP device.

THEN

Placebo for VSV IN HIV gag (Sodium Chloride for Injection, USP 0.9%) administered as 1 mL IM in left deltoid **and** 1 mL IM in right deltoid at Month 6.

Group 2

Treatment 2 (T2): GENEVAX® IL-12 DNA Plasmid 125 mcg admixed with ProfectusVax DNA Plasmid 1.5 mg (HIV-1 gag/pol 0.75 mg admixed with HIV-1 nef/tat/vif, env 0.75 mg) administered as 0.55 mL IM in left deltoid **and** GENEVAX® IL-12 DNA Plasmid 125 mcg admixed with ProfectusVax DNA Plasmid 1.5 mg (HIV-1 gag/pol 0.75 mg admixed with HIV-1 nef/tat/vif, env 0.75 mg) administered as 0.55 mL IM in right deltoid at Months 0, 1, and 3 using the Ichor Medical Systems TDS EP device.

THEN

VSV IN HIV gag $1x10^8$ PFU† administered as $5x10^7$ PFU in 1 mL IM in left deltoid **and** $5x10^7$ PFU in 1 mL IM in right deltoid at Month 6.*

**Nominal dose (per label) – actual dose is 3.4 x 10^7 PFU given as 1.7 $x10^7$PFU in each deltoid*

†Safety data from HVTN 090 will be evaluated by the HVTN 087 PSRT prior to administration of VSV IN HIV gag boosting in HVTN 087. Any change in dose of VSV IN HIV gag would be specified by a protocol modification.

Control 2 (C2): Placebos for GENEVAX® IL-12 DNA Plasmid /ProfectusVax DNA Plasmid (Sodium Chloride for Injection, USP 0.9%) administered as 0.55 mL IM in left deltoid **and** Placebos for GENEVAX® IL-12 DNA Plasmid /ProfectusVax DNA Plasmid (Sodium Chloride for Injection, USP 0.9%) administered as 0.55 mL IM in right deltoid at Months 0, 1, and 3 using the Ichor Medical Systems TDS EP device.

THEN

Placebo for VSV IN HIV gag (Sodium Chloride for Injection, USP 0.9%) administered as 1 mL IM in left deltoid **and** 1 mL IM in right deltoid at Month 6.

Group 3

Treatment 3 (T3): GENEVAX® IL-12 DNA Plasmid 500 mcg admixed with ProfectusVax DNA Plasmid 1.5 mg (HIV-1 gag/pol 0.75 mg admixed with HIV-1 nef/tat/vif, env 0.75 mg) administered as 0.75 mL IM in left deltoid **and** GENEVAX® IL-12 DNA Plasmid 500 mcg admixed with ProfectusVax DNA Plasmid 1.5 mg (HIV-1 gag/pol 0.75 mg admixed with HIV-1 nef/tat/vif, env 0.75 mg) administered as 0.75 mL IM in right deltoid at Months 0, 1, and 3 using the Ichor Medical Systems TDS EP device.

THEN

VSV IN HIV gag 1x10^8 PFU† administered as 5x10^7 PFU in 1 mL IM in left deltoid **and** 5x10^7 PFU in 1 mL IM in right deltoid at Month 6.*

**Nominal dose (per label) – actual dose is 3.4 x 10^7 PFU given as 1.7 x10^{7}PFU in each deltoid*

†Safety data from HVTN 090 will be evaluated by the HVTN 087 PSRT prior to administration of VSV IN HIV gag boosting in HVTN 087. Any change in dose of VSV IN HIV gag would be specified by a protocol modification.

Control 3 (C3): Placebos for GENEVAX® IL-12 DNA Plasmid /ProfectusVax DNA Plasmid (Sodium Chloride for Injection, USP 0.9%) administered as 0.75 mL IM in left deltoid **and** Placebos for GENEVAX® IL-12 DNA Plasmid /ProfectusVax DNA Plasmid (Sodium Chloride for Injection, USP 0.9%) administered as 0.75 mL IM in right deltoid at Months 0, 1, and 3 using the Ichor Medical Systems TDS EP device.

THEN

Placebo for VSV IN HIV gag (Sodium Chloride for Injection, USP 0.9%) administered as 1 mL IM in left deltoid **and** 1 mL IM in right deltoid at Month 6.

Group 4

Treatment 4 (T4): GENEVAX® IL-12 DNA Plasmid 750 mcg admixed with ProfectusVax DNA Plasmid 1.5 mg (HIV-1 gag/pol 0.75 mg admixed with HIV-1 nef/tat/vif, env 0.75 mg) administered as 0.9 mL IM in left deltoid **and** GENEVAX® IL-12 DNA Plasmid 750 mcg admixed with ProfectusVax DNA Plasmid 1.5 mg (HIV-1 gag/pol 0.75 mg admixed with HIV-1 nef/tat/vif, env 0.75 mg) administered as 0.9 mL IM in right deltoid at Months 0, 1, and 3 using the Ichor Medical Systems TDS EP device.

THEN

VSV IN HIV gag 1x10^8 PFU† administered as 5x10^7 PFU in 1 mL IM in left deltoid **and** 5x10^7 PFU in 1 mL IM in right deltoid at Month 6.*

**Nominal dose (per label) – actual dose is 3.4 x 10^7 PFU given as 1.7 x10^{7}PFU in each deltoid*

†Safety data from HVTN 090 will be evaluated by the HVTN 087 PSRT prior to administration of VSV IN HIV gag boosting in HVTN 087. Any change in dose of VSV IN HIV gag would be specified by a protocol modification.

Control 4 (C4): Placebos for GENEVAX® IL-12 DNA Plasmid /ProfectusVax DNA Plasmid (Sodium Chloride for Injection, USP 0.9%) administered as

0.9 mL IM in left deltoid **and** Placebos for GENEVAX® IL-12 DNA Plasmid /ProfectusVax DNA Plasmid (Sodium Chloride for Injection, USP 0.9%) administered as 0.9 mL IM in right deltoid at Months 0, 1, and 3 using the Ichor Medical Systems TDS EP device.

THEN

Placebo for VSV IN HIV gag (Sodium Chloride for Injection, USP 0.9%) administered as 1 mL IM in left deltoid **and** 1 mL IM in right deltoid at Month 6.

8.2 Study product formulation

ProfectusVax DNA Plasmid HIV-1 *gag/pol* (component of HIV MAG pDNA)

ProfectusVax DNA Plasmid HIV-1 *gag*/*pol* is a clear, colorless solution in a 2 mL single use vial. Each 2 mL vial contains 0.8 ± 0.04 mL of ProfectusVax HIV-1 gag/*pol* pDNA at a concentration of 3 mg/mL in 30mM citrate buffer pH 6.5 containing 150 mM NaCl, 0.015 EDTA, and 0.25% bupivacaine. The product should be stored at 2°C to 8°C.

The product is contraindicated in participants with known hypersensitivity to bupivacaine.

ProfectusVax DNA Plasmid HIV-1 *nef/tat/vif, env* (component of HIV MAG pDNA)

ProfectusVax DNA Plasmid HIV-1 *nef*/*tat*/*vif*, *env* is a clear, colorless solution in a 2 mL single use vial. Each 2 mL vial contains 0.8 ± 0.04 mL of ProfectusVax HIV-1 *nef*/*tat*/*vif*, *env* pDNA at a concentration of 3 mg/mL in 30mM citrate buffer pH 6.5 containing 150 mM NaCl, 0.015 EDTA, and 0.25% bupivacaine-HCl. The product should be stored at 2°C to 8°C.

The product is contraindicated in participants with known hypersensitivity to bupivacaine.

GENEVAX® *IL-12* DNA Plasmid (GENEVAX® *IL-12*-4532, WLV-103M, *IL-12* pDNA, *IL-12* plasmid**)**

GENEVAX® *IL-12* DNA Plasmid is a clear colorless solution in a 2 mL single use vial. Each 2 mL vial contains 0.9 ± 0.04 mL of *IL-12* pDNA at a concentration of 2 mg/mL in 30 mM citrate buffer pH 6.5 containing 150 mM NaCl, 0.01% EDTA and 0.25% bupivacaine-HCl. The product should be stored at 2° to 8°C.

The product is contraindicated in participants with known hypersensitivity to bupivacaine.

VSV IN HIV gag ($rVSV_{IN}N4CT1gag1$, VSV HIV gag)

ProfectusVax VSV_{IN} HIV gag is a clear colorless solution. It is formulated in an aqueous liquid phosphate buffer containing gelatin as a virus stabilizer. Each 3 mL single use vial contains 1.3 mL of vaccine. Each vial will be labeled as containing VSV_{IN} HIV gag at $5x10^7$ PFU/mL. (Please see the comparison of label claim to release potency titer in the table below).

Comparison of Label Claim to Release Potency Titer

Label Claim	Release Potency Titer (T_o)
5x10[7] PFU/mL	1.7x10[7] PFU/mL

The product should be stored at -70°C to -85°C. Once thawed the product may not be refrozen. If for any reason the product will not be administered immediately after thawing, it can be stored in the vial at 2°C to 8°C for up to 4 hours.

Placebo (Sodium Chloride for Injection, USP 0.9%)

Sodium Chloride for Injection, USP 0.9% will be used as the placebo for all groups. The volume to be administered will vary from group to group to maintain the blind between active and placebo treatment (T and C) in each group. The vials must be stored as directed by the manufacturer of the product unless otherwise instructed.

The study products are described in further detail in the IB.

8.3 Preparation of study products

8.3.1 ProfectusVax DNA Plasmid 1.5 mg (HIV-1 *gag/pol* 0.75 mg admixed with HIV-1 *nef/tat/vif*, *env* 0.75 mg) or Placebo (Group 1)

<u>ProfectusVax DNA Plasmid 1.5 mg (HIV-1 *gag/pol* 0.75 mg admixed with HIV-1 *nef/tat/vif*, *env* 0.75 mg)</u>

One vial containing HIV-1 *gag/pol* at 3 mg/mL and one vial containing HIV-1 *nef/tat/vif*, *env* at 3 mg/mL will be used to prepare the two syringes. The vials should be visually inspected prior to use. If the vial contains material different from its description above, do not use the vial and contact the protocol pharmacist. The pharmacist will also inspect the sterile pouch containing the TriGrid™ Application Cartridge for integrity and verify the Cartridge has not passed expiration. If any tears or gaps in the sterile pouch are noted or if the expiration date has been reached, the Application Cartridge should be placed in quarantine and the protocol pharmacist should be informed. *Note: In the event that the Cartridge Cap has become detached from the Cartridge in the sterile pouch, the Cartridge may still be used if the Cartridge Cap can be replaced without contaminating the patient contact portion of the Cartridge.*

- ☐ Using aseptic technique, the pharmacist will use a 0.3 mL syringe to withdraw 0.1 mL of HIV-1 *gag/pol* from the vial. Discard the syringe containing 0.1 mL HIV-1 *gag/pol* into an appropriate sharps container. This vial will now contain 0.7 mL of HIV-1 *gag/pol*.
- ☐ Using aseptic technique, the pharmacist will use a 3 mL syringe, or smaller, to withdraw 0.7 mL of HIV-1 *nef/tat/vif*, *env* and inject directly into the vial containing 0.7 mL HIV-1 *gag/pol*.
- ☐ The pharmacist will mix the vial containing the mixture of HIV-1 *gag/pol* and HIV-1 *nef/tat/vif*, *env* plasmids 10 times by gentle inversion.
- ☐ Using two new 3 mL syringes (BD# 309585) each fitted with a 22 Ga x 1.5'' needle (BD# 305156), the pharmacist will withdraw 0.5 mL of this final product into each syringe and insert the syringe with the needle still attached into each Application Cartridge as directed in the *HVTN 087 Study Specific Procedures*.

<u>Note</u>: Use the syringe tabs to insert the syringe into the Application Cartridge. Do not depress the syringe plunger during insertion of the syringe into the Application Cartridge as this will cause the agent to be injected into the Cartridge.

<u>Caution</u>: During the syringe loading procedure, do not physically touch the syringe needle or cause it to come in contact with a non-sterile surface. Note that, although the inside of the Application Cartridge is sterile, contact between the syringe needle and the inner housing of the Application Cartridge should be avoided to the extent possible. Contact between the syringe needle and Application Cartridge could result in deformation of the syringe needle and increased discomfort for the subject upon insertion. If contamination and/or deformation of the injection needle is suspected, replace the injection needle with a new BD 22 Ga x 1.5" injection needle (BD model 305156). To prevent loss of dosage make sure to withdraw agent from needle before replacing.

- ☐ Each Application Cartridge will have the Participant's ID number written on it using a Sharpie or other similar pen. The cartridge will then be placed back in the pouch that it was removed from and the pouch will be labeled as "HIV MAG 1500 mcg/0.5 mL or placebo per syringe (syringe __ of 2)" in such a fashion that the label can be removed and affixed to the case report form (CRF).
- ☐ The pouch containing the syringe must be labeled with a four-hour expiration date from the time the vials are removed from the refrigerator and the contents admixed. The label must also contain the words "Administer as soon as possible."

Any unused portion of entered vials and expired pre-filled syringes should be disposed of in accordance with institutional or pharmacy policy.

<u>Placebo for ProfectusVax DNA Plasmid 1.5 mg</u>

- ☐ One vial containing Sodium Chloride for Injection, USP 0.9% will be needed to prepare the two syringes. The vial should be visually inspected prior to use. The pharmacist will also inspect the sterile pouch containing the TriGrid™ Application Cartridge for integrity and verify the Cartridge has not passed expiration. If any tears or gaps in the sterile pouch are noted or if the expiration date has been reached, the Application Cartridge should be placed in quarantine and the protocol pharmacist should be informed. *Note: In the event that the Cartridge Cap has become detached from the Cartridge in the sterile pouch, the Cartridge may still be used if the Cartridge Cap can be replaced without contaminating the patient contact portion of the Cartridge.*
- ☐ Using aseptic technique, the pharmacist, using two new 3 mL syringes (BD# 309585) each fitted with a 22 Ga x 1.5'' needle (BD# 305156), will withdraw 0.5 mL of the Sodium Chloride for Injection, USP 0.9% into each syringe and insert the syringe with the needle still attached into each Application Cartridge as directed in the *HVTN 087 Study Specific Procedures*.

<u>Note</u>: Use the syringe tabs to insert the syringe into the Application Cartridge. Do not depress the syringe plunger during insertion of the syringe into the Application Cartridge as this will cause the agent to be injected into the Cartridge.

<u>Caution</u>: During the syringe loading procedure, do not physically touch the syringe needle or cause it to come in contact with a non-sterile surface. Note that, although the inside of the Application Cartridge is sterile, contact between the syringe needle and the inner housing of the Application Cartridge should be avoided to the extent possible. Contact between the syringe needle and Application Cartridge could result in deformation of the syringe needle and increased discomfort for the subject upon insertion. If contamination and/or deformation of the injection needle is suspected, replace the injection needle with a new BD 22 Ga x 1.5" injection needle (BD model 305156). To prevent loss of dosage make sure to withdraw agent from needle before replacing.

- ☐ Each Application Cartridge will have the Participant's ID number written on it using a Sharpie or other similar pen. The cartridge will then be placed back in the pouch that it was removed from and the pouch will be labeled as "HIV MAG 1500 mcg/0.5 mL or placebo per syringe (syringe __ of 2)" in such a fashion that the label can be removed and affixed to the CRF.
- ☐ The pouch containing the syringe must be labeled with a four-hour expiration date from the time the vial is removed from storage. The label must also contain the words "Administer as soon as possible."

8.3.2 GENEVAX® IL-12 DNA Plasmid 125 mcg admixed with ProfectusVax DNA Plasmid 1.5 mg (HIV-1 *gag/pol* 0.75 mg admixed with HIV-1 *nef/tat/vif*, *env* 0.75 mg) or Placebo (Group 2)

GENEVAX® IL-12 DNA Plasmid 125 mcg admixed with ProfectusVax DNA Plasmid 1.5 mg (HIV-1 *gag*/*pol* 0.75 mg admixed with HIV-1 *nef*/*tat*/*vif*, *env* 0.75 mg)

One vial containing HIV-1 *gag*/*pol* 3 mg/mL, one vial containing HIV-1 *nef*/*tat*/*vif*, *env* at 3 mg/mL, and one vial containing IL-12 DNA Plasmid at 2 mg/mL will be needed to prepare the two syringes. The vials should be visually inspected prior to use. If the vial contains material different from its description above, do not use the vial and contact the protocol pharmacist. The pharmacist will also inspect the sterile pouch containing the TriGrid™ Application Cartridge for integrity and verify the Cartridge has not passed expiration. If any tears or gaps in the sterile pouch are noted or if the expiration date has been reached, the Application Cartridge should be placed in quarantine and the protocol pharmacist should be informed. *Note: In the event that the Cartridge Cap has become detached from the Cartridge in the sterile pouch, the Cartridge may still be used if the Cartridge Cap can be replaced without contaminating the patient contact portion of the Cartridge.*

- ☐ Using aseptic technique, the pharmacist will use a 0.3 mL syringe to withdraw 0.1 mL of HIV-1 *gag*/*pol* from the vial. Discard the syringe containing 0.1 mL HIV-1 *gag*/*pol* into an appropriate sharps container. This vial will now contain 0.7 mL of HIV-1 *gag*/*pol*.
- ☐ Using aseptic technique, the pharmacist will use a 1 mL syringe to withdraw 0.7 mL of HIV-1 *nef*/*tat*/*vif*, *env* and inject directly into the vial containing 0.7 mL HIV-1 *gag*/*pol*.
- ☐ Using aseptic technique, the pharmacist will use a 0.3 mL syringe to withdraw 0.175 mL of GENEVAX® IL-12 DNA Plasmid and inject directly into the vial containing the HIV-1 *gag*/*pol* and HIV-1 *nef*/*tat*/*vif*, *env* mixture. This vial will now contain

0.175 + 0.7 + 0.7 mL = 1.575 mL of GENEVAX® IL-12 DNA Plasmid admixed with ProfectusVax DNA Plasmid (HIV-1 *gag*/*pol* admixed with HIV-1 *nef*/*tat*/*vif*, *env*).

- ☐ The pharmacist will mix the vial containing the mixture of GENEVAX® IL-12 DNA Plasmid admixed with ProfectusVax DNA Plasmid (HIV-1 *gag*/*pol* admixed with HIV-1 *nef*/*tat*/*vif*, *env*).plasmids 10 times by gentle inversion.
- ☐ Using two new 3 mL syringes (BD# 309585) each fitted with a 22 Ga x 1.5'' needle (BD# 305156), the pharmacist will withdraw 0.55 mL of this final product into each syringe and insert the syringe with the needle still attached into each Application Cartridge as directed in the *HVTN 087 Study Specific Procedures*.

<u>Note</u>: Use the syringe tabs to insert the syringe into the Application Cartridge. Do not depress the syringe plunger during insertion of the syringe into the Application Cartridge as this will cause the agent to be injected into the Cartridge.

<u>Caution</u>: During the syringe loading procedure, do not physically touch the syringe needle or cause it to come in contact with a non-sterile surface. Note that, although the inside of the Application Cartridge is sterile, contact between the syringe needle and the inner housing of the Application Cartridge should be avoided to the extent possible. Contact between the syringe needle and Application Cartridge could result in deformation of the syringe needle and increased discomfort for the subject upon insertion. If contamination and/or deformation of the injection needle is suspected, replace the injection needle with a new BD 22 Ga x 1.5" injection needle (BD model 305156). To prevent loss of dosage make sure to withdraw agent from needle before replacing.

- ☐ Each Application Cartridge will have the Participant's ID number written on it using a Sharpie or other similar pen. The cartridge will then be placed back in the pouch that it was removed from and the pouch will be labeled as "HIV MAG 1500 mcg / IL-12 125 mcg/0.55 mL or placebo per syringe (syringe __ of 2)" in such a fashion that the label can be removed and affixed to the CRF.
- ☐ The pouch containing the syringe must be labeled with a four-hour expiration date from the time the vials are removed from the refrigerator and the contents admixed. The label must also contain the words "Administer as soon as possible."

Any unused portion of entered vials and expired pre-filled syringes should be disposed of in accordance with institutional or pharmacy policy.

<u>Placebo for GENEVAX® IL-12 DNA Plasmid / ProfectusVax DNA Plasmid</u>

- ☐ One vial containing Sodium Chloride for Injection, USP 0.9% will be needed to prepare the two syringes. The vial should be visually inspected prior to use. The pharmacist will also inspect the sterile pouch containing the TriGrid™ Application Cartridge for integrity and verify the Cartridge has not passed expiration. If any tears or gaps in the sterile pouch are noted or if the expiration date has been reached, the Application Cartridge should be placed in quarantine and the protocol pharmacist should be informed. *Note: In the event that the Cartridge Cap has become detached from the Cartridge in the sterile pouch, the Cartridge may still be used if the Cartridge Cap can be replaced without contaminating the patient contact portion of the Cartridge.*

- ☐ Using aseptic technique, the pharmacist, using two new 3 mL syringes (BD# 309585) each fitted with a 22 Ga x 1.5'' needle (BD# 305156), will withdraw 0.55 mL of the Sodium Chloride for Injection, USP 0.9% into each syringe and insert the syringe with the needle still attached into each Application Cartridge as directed in the *HVTN 087 Study Specific Procedures*.

Note: Use the syringe tabs to insert the syringe into the Application Cartridge. Do not depress the syringe plunger during insertion of the syringe into the Application Cartridge as this will cause the agent to be injected into the Cartridge.

Caution: During the syringe loading procedure, do not physically touch the syringe needle or cause it to come in contact with a non-sterile surface. Note that, although the inside of the Application Cartridge is sterile, contact between the syringe needle and the inner housing of the Application Cartridge should be avoided to the extent possible. Contact between the syringe needle and Application Cartridge could result in deformation of the syringe needle and increased discomfort for the subject upon insertion. If contamination and/or deformation of the injection needle is suspected, replace the injection needle with a new BD 22 Ga x 1.5" injection needle (BD model 305156). To prevent loss of dosage make sure to withdraw agent from needle before replacing.

- ☐ Each Application Cartridge will have the Participant's ID number written on it using a Sharpie or other similar pen. The cartridge will then be placed back in the pouch that it was removed from and the pouch will be labeled as "HIV MAG 1500 mcg / IL-12 125 mcg/0.55 mL or placebo per syringe (syringe __ of 2)" in such a fashion that the label can be removed and affixed to the CRF.
- ☐ The pouch containing the syringe must be labeled with a four-hour expiration date from the time the vial is removed from storage. The label must also contain the words "Administer as soon as possible."

8.3.3 GENEVAX® IL-12 DNA Plasmid 500 mcg admixed with ProfectusVax DNA Plasmid 1.5 mg (HIV-1 *gag/pol* 0.75 mg admixed with HIV-1 *nef/tat/vif*, *env* 0.75 mg) or Placebo (Group 3)

GENEVAX® IL-12 DNA Plasmid 500 mcg admixed with ProfectusVax DNA Plasmid 1.5 mg (HIV-1 *gag/pol* 0.75 mg admixed with HIV-1 *nef/tat/vif*, *env* 0.75 mg)

One vial containing HIV-1 *gag/pol* at 3 mg/mL, one vial containing HIV-1 *nef/tat/vif*, *env* at 3 mg/mL, and one vial containing IL-12 DNA Plasmid at 2mg/mL will be needed to prepare the two syringes. The vials should be visually inspected prior to use. If the vial contains material different from its description above, do not use the vial and contact the protocol pharmacist. The pharmacist will also inspect the sterile pouch containing the TriGrid™ Application Cartridge for integrity and verify the Cartridge has not passed expiration. If any tears or gaps in the sterile pouch are noted or if the expiration date has been reached, the Application Cartridge should be placed in quarantine and the protocol pharmacist should be informed. *Note: In the event that the Cartridge Cap has become detached from the Cartridge in the sterile pouch, the Cartridge may still be used if the Cartridge Cap can be replaced without contaminating the patient contact portion of the Cartridge.*

- ☐ Using aseptic technique, the pharmacist will use a 0.3 mL syringe to withdraw 0.1 mL of HIV-1 *gag/pol* from the vial. Discard the syringe containing 0.1 mL HIV-1

gag/*pol* into an appropriate sharps container. This vial will now contain 0.7 mL of HIV-1 *gag*/*pol*.

- ☐ Using aseptic technique, the pharmacist will use a 1 mL syringe to withdraw 0.7 mL of HIV-1 *nef*/*tat*/*vif*, *env* and inject directly into the vial containing 0.7 mL HIV-1 *gag*/*pol*.
- ☐ Using aseptic technique, the pharmacist will use a 1 mL syringe to withdraw 0.7 mL of GENEVAX® IL-12 DNA Plasmid and inject directly into the vial containing the HIV-1 *gag*/*pol* and HIV-1 *nef*/*tat*/*vif*, *env* mixture. This vial will now contain 0.7 + 0.7 + 0.7 ml = 2.1 mL of GENEVAX® IL-12 DNA Plasmid admixed with ProfectusVax DNA Plasmid (HIV-1 *gag*/*pol* admixed with HIV-1 *nef*/*tat*/*vif*, *env*).
- ☐ The pharmacist will mix the vial containing the mixture of GENEVAX® IL-12 DNA Plasmid admixed with ProfectusVax DNA Plasmid (HIV-1 *gag*/*pol* admixed with HIV-1 *nef*/*tat*/*vif*, *env*) plasmids 10 times by gentle inversion.
- ☐ Using two new 3 mL syringes (BD# 309585) each fitted with a 22 Ga x 1.5'' needle (BD# 305156), the pharmacist will withdraw 0.75 mL of this final product into each syringe and insert the syringe with the needle still attached into each Application Cartridge as directed in the *HVTN 087 Study Specific Procedures*.

Note: Use the syringe tabs to insert the syringe into the Application Cartridge. Do not depress the syringe plunger during insertion of the syringe into the Application Cartridge as this will cause the agent to be injected into the Cartridge.

Caution: During the syringe loading procedure, do not physically touch the syringe needle or cause it to come in contact with a non-sterile surface. Note that, although the inside of the Application Cartridge is sterile, contact between the syringe needle and the inner housing of the Application Cartridge should be avoided to the extent possible. Contact between the syringe needle and Application Cartridge could result in deformation of the syringe needle and increased discomfort for the subject upon insertion. If contamination and/or deformation of the injection needle is suspected, replace the injection needle with a new BD 22 Ga x 1.5" injection needle (BD model 305156). To prevent loss of dosage make sure to withdraw agent from needle before replacing.

- ☐ Each Application Cartridge will have the Participant's ID number written on it using a Sharpie or other similar pen. The cartridge will then be placed back in the pouch that it was removed from and the pouch will be labeled as "HIV MAG 1500 mcg / IL-12 500 mcg/0.75 mL or placebo per syringe (syringe __ of 2)" in such a fashion that the label can be removed and affixed to the CRF.
- ☐ The pouch containing the syringe must be labeled with a four-hour expiration date from the time the vials are removed from the refrigerator and the contents admixed. The label must also contain the words "Administer as soon as possible."

Any unused portion of entered vials and expired pre-filled syringes should be disposed of in accordance with institutional or pharmacy policy.

Placebo for GENEVAX® IL-12 DNA Plasmid / ProfectusVax DNA Plasmid

One vial containing Sodium Chloride for Injection, USP 0.9% will be needed to prepare the two syringes. The vial should be visually inspected prior to use.

Using aseptic technique, the pharmacist using two new 3 mL syringes (BD# 309585) each fitted with a 22 Ga x 1.5'' needle (BD# 305156) , will withdraw 0.75 mL of the Sodium Chloride for Injection, USP 0.9% into each syringe and insert the syringe with the needle still attached into each Application Cartridge as directed in the *HVTN 087 Study Specific Procedures*.

Note: Use the syringe tabs to insert the syringe into the Application Cartridge. Do not depress the syringe plunger during insertion of the syringe into the Application Cartridge as this will cause the agent to be injected into the Cartridge.

Caution: During the syringe loading procedure, do not physically touch the syringe needle or cause it to come in contact with a non-sterile surface. Note that, although the inside of the Application Cartridge is sterile, contact between the syringe needle and the inner housing of the Application Cartridge should be avoided to the extent possible. Contact between the syringe needle and Application Cartridge could result in deformation of the syringe needle and increased discomfort for the subject upon insertion. If contamination and/or deformation of the injection needle is suspected, replace the injection needle with a new BD 22 Ga x 1.5" injection needle (BD model 305156). To prevent loss of dosage make sure to withdraw agent from needle before replacing.

- ☐ Each Application Cartridge will have the Participant's ID number written on it using a Sharpie or other similar pen. The cartridge will then be placed back in the pouch that it was removed from and the pouch will be labeled as "HIV MAG 1500 mcg / IL-12 500 mcg/0.75 mL or placebo per syringe (syringe __ of 2)" in such a fashion that the label can be removed and affixed to the CRF.
- ☐ The pouch containing the syringe must be labeled with a four-hour expiration date from the time the vial is removed from storage. The label must also contain the words "Administer as soon as possible."

8.3.4 GENEVAX® IL-12 DNA Plasmid 750 mcg admixed with ProfectusVax DNA Plasmid 1.5 mg (HIV-1 *gag/pol* 0.75 mg admixed with HIV-1 *nef/tat/vif*, *env* 0.75 mg) or Placebo (Group 4)

GENEVAX® IL-12 DNA Plasmid 750 mcg admixed with ProfectusVax DNA Plasmid 1.5 mg (HIV-1 *gag/pol* 0.75 mg admixed with HIV-1 *nef/tat/vif*, *env* 0.75 mg)

One vial containing HIV-1 *gag/pol* at 3 mg/mL, one vial containing HIV-1 *nef/tat/vif*, *env* at 3 mg/mL, and one vial containing IL-12 DNA Plasmid at 2mg/mL will be needed to prepare the two syringes. The vials should be visually inspected prior to use. If the vial contains material different from its description above, do not use the vial and contact the protocol pharmacist. The pharmacist will also inspect the sterile pouch containing the TriGrid™ Application Cartridge for integrity and verify the Cartridge has not passed expiration. If any tears or gaps in the sterile pouch are noted or if the expiration date has been reached, the Application Cartridge should be placed in quarantine and the protocol pharmacist should be informed. *Note: In the event that the Cartridge Cap has become detached from the Cartridge in the sterile pouch, the Cartridge may still be used if the Cartridge Cap can be replaced without contaminating the patient contact portion of the Cartridge.*

☐ Using aseptic technique, the pharmacist will use a 0.3 mL syringe to withdraw 0.1 mL of HIV-1 *gag*/*pol* from the vial. Discard the syringe containing 0.1 mL HIV-1 *gag*/*pol* into an appropriate sharps container. This vial will now contain 0.7 mL of HIV-1 *gag*/*pol*.

☐ Using aseptic technique, the pharmacist will use a 1 mL insulin syringe to withdraw 0.7 mL of HIV-1 *nef*/*tat*/*vif*, *env* and inject directly into the vial containing 0.7 mL HIV-1 *gag*/*pol*.

☐ Using aseptic technique, the pharmacist will use a 3 mL syringe to withdraw 1 mL of GENEVAX® IL-12 DNA Plasmid and inject directly into the vial containing the HIV-1 *gag*/*pol* and HIV-1 *nef*/*tat*/*vif*, *env* mixture. This vial will now contain 1 + 0.7 + 0.7 mL = 2.4 mL of GENEVAX® IL-12 DNA Plasmid admixed with ProfectusVax DNA Plasmid (HIV-1 *gag*/*pol* admixed with HIV-1 *nef*/*tat*/*vif*, *env*).

☐ The pharmacist will mix the vial containing the mixture of GENEVAX® IL-12 DNA Plasmid admixed with ProfectusVax DNA Plasmid (HIV-1 *gag*/*pol* admixed with HIV-1 *nef*/*tat*/*vif*, *env*) plasmids 10 times by gentle inversion.

☐ Using two new 3 mL syringes (BD# 309585) each fitted with a 22 Ga x 1.5'' needle (BD# 305156), the pharmacist will withdraw 0.9 mL of this final product into each syringe and insert the syringe with the needle still attached into each Application Cartridge as directed in the *HVTN 087 Study Specific Procedures*.

<u>Note</u>: *Use the syringe tabs to insert the syringe into the Application Cartridge. Do not depress the syringe plunger during insertion of the syringe into the Application Cartridge as this will cause the agent to be injected into the Cartridge.*

<u>Caution</u>: *During the syringe loading procedure, do not physically touch the syringe needle or cause it to come in contact with a non-sterile surface. Note that, although the inside of the Application Cartridge is sterile, contact between the syringe needle and the inner housing of the Application Cartridge should be avoided to the extent possible. Contact between the syringe needle and Application Cartridge could result in deformation of the syringe needle and increased discomfort for the subject upon insertion. If contamination and/or deformation of the injection needle is suspected, replace the injection needle with a new BD 22 Ga x 1.5" injection needle (BD model 305156). To prevent loss of dosage make sure to withdraw agent from needle before replacing.*

☐ Each Application Cartridge will have the Participant's ID number written on it using a Sharpie or other similar pen. The cartridge will then be placed back in the pouch that it was removed from and the pouch will be labeled as "HIV MAG 1500 mcg / IL-12 750 mcg/0.9 mL or placebo per syringe (syringe __ of 2)" in such a fashion that the label can be removed and affixed to the CRF.

☐ The pouch containing the syringe must be labeled with a four-hour expiration date from the time the vials are removed from the refrigerator and the contents admixed. The label must also contain the words "Administer as soon as possible."

Any unused portion of entered vials and expired pre-filled syringes should be disposed of in accordance with institutional or pharmacy policy.

Placebo for GENEVAX® IL-12 DNA Plasmid / ProfectusVax DNA Plasmid

One vial containing Sodium Chloride for Injection, USP 0.9% will be needed to prepare the two syringes. The vial should be visually inspected prior to use.

Using aseptic technique, the pharmacist, using two new 3 mL syringes (BD# 309585) each fitted with a 22 Ga x 1.5'' needle (BD# 305156), will withdraw 0.9 mL of the Sodium Chloride for Injection, USP 0.9% into each syringe and insert the syringe with the needle still attached into each Application Cartridge as directed in the *HVTN 087 Study Specific Procedures*.

Note: *Use the syringe tabs to insert the syringe into the Application Cartridge. Do not depress the syringe plunger during insertion of the syringe into the Application Cartridge as this will cause the agent to be injected into the Cartridge.*

Caution: *During the syringe loading procedure, do not physically touch the syringe needle or cause it to come in contact with a non-sterile surface. Note that, although the inside of the Application Cartridge is sterile, contact between the syringe needle and the inner housing of the Application Cartridge should be avoided to the extent possible. Contact between the syringe needle and Application Cartridge could result in deformation of the syringe needle and increased discomfort for the subject upon insertion. If contamination and/or deformation of the injection needle is suspected, replace the injection needle with a new BD 22 Ga x 1.5" injection needle (BD model 305156). To prevent loss of dosage make sure to withdraw agent from needle before replacing.*

- ☐ Each Application Cartridge will have the Participant's ID number written on it using a Sharpie or other similar pen. The cartridge will then be placed back in the pouch that it was removed from and the pouch will be labeled as "HIV MAG 1500 mcg / IL-12 750 mcg/0.9 mL or placebo per syringe (syringe __ of 2)" in such a fashion that the label can be removed and affixed to the CRF.
- ☐ The pouch containing the syringe must be labeled with a four-hour expiration date from the time the vial is removed from storage. The label must also contain the words "Administer as soon as possible."

8.3.5 VSV_{IN} HIV *gag* vaccine $1x10^8$ PFU dose or Placebo (All Groups)

NOTE: Protective equipment (gloves, eye protection and mask) should be worn during study product preparation for the VSV_{IN} HIV *gag* vaccine (not placebo). Care should be taken to avoid aerosol production.

VSV_{IN} HIV *gag* vaccine$1x10^8$ PFU dose (actual dose $3.4x10^7$ PFU/2 mL)

To prepare, the pharmacist will remove two vials of VSV IN HIV gag vaccine labeled as $5x10^7$ PFU/mL from the freezer and allow to thaw at room temperature. Once thawed, invert each vial containing VSV IN HIV gag 10 times rapidly and successively.

Using aseptic technique, the pharmacist will withdraw 1 mL of the VSV IN HIV gag vaccine from each vial into a separate 3 mL or 5 mL syringe.

Each syringe should be labeled as VSV_{IN} HIV *gag* $1.7x10^7$ PFU/mL or Placebo (___ of 2 syringes). The syringes must be labeled with a four-hour expiration date/time from the time the vial is removed from the freezer. The label must also contain the words "Administer as soon as possible."

Any unused portion of entered vials and expired pre-filled syringes should be disposed of in a bio-hazard container.

Placebo for VSV_{IN} HIV *gag* vaccine

To prepare, the pharmacist will remove a sufficient quantity of vials of Sodium Chloride for Injection USP, 0.9% from storage.

Using aseptic technique, the pharmacist will withdraw 1 mL of the Sodium Chloride for Injection USP, 0.9% into two separate 3 mL or 5 mL syringes so that each syringe contains 1 mL.

Each syringe should be labeled as VSV_{IN} HIV *gag* $1.7x10^7$ PFU/mL or Placebo (___ of 2 syringes). The syringes must be labeled with a four-hour expiration date/time from the time the vial is removed from storage. The label must also contain the words "Administer as soon as possible."

8.4 Administration

At sites where registered pharmacists are legally authorized to administer drug, the HVTN CRS may choose to have the HVTN CRS pharmacist administer the vaccinations.

Months 0, 1, and 3 only

Administration of vaccine or placebo for all groups consists of 2 injections, 1 each in left and right medial deltoids using the Ichor Medical Systems Intramuscular TriGrid Delivery System (TDS-IM) EP device at Months 0, 1, and 3.

The TDS-IM device will be used as directed by Ichor Medical Systems, Inc. (Please refer to the TDS-IM User Manual for further instruction). The TDS-IM has three components: The TDS-IM Pulse Stimulator, the Integrated Applicator (reusable) and the Application Cartridge (single-use). The Application Cartridge is the only patient contact component of the system. Only use Becton Dickinson 3.0 mL sterile syringe and Becton Dickinson 22 Ga x 1.5" injection needle with the Application Cartridge.

The used TDS-IM Application Cartridge should be disposed of in accordance with institutional policy in the clinic. It should NOT be returned to pharmacy.

Prior to applying the EP procedure, the participant's skin to muscle thickness must be assessed for each arm separately. This may be assessed once, at any time prior to the first injection with EP for a participant, and is not required prior to each procedure. However the skin fold thickness should be reassessed for a participant whose body weight has changed by 10% since the previous skin fold thickness assessment. The skin fold thickness for each arm is recorded in the participant study chart. The Application

Cartridge penetration depth will be set prior to each procedure according to these measurements.

To assess the thickness of the skin and subcutaneous tissue use your thumb and forefinger to "pinch" the skin and subcutaneous tissue overlying the muscle at the administration site together and measure the fold thickness using skin calipers or other suitable measurement device as directed in the *HVTN 087 Study Specific Procedures*. Based on this measurement, use the chart below to select the appropriate depth setting on the Application Cartridge for each arm. Record the skin fold thickness for each arm in the participant study chart. The selected depth setting on the Cartridge is indicated by the number of indicator lines visible on Cartridge (see Table 8-1)

Table 8-1 Selection of device depth setting

Range of Measured **skin fold** Thickness [mm]	Device Depth Selection	Number of indicator lines visible
< 14 mm	A	3
14-24 mm	B	2
24-40 mm	C	1
>40 mm	Exclude from protocol	Exclude from protocol

Month 6 only

Two 1 mL injections of the VSV_{IN} HIV *gag* or placebo will be administered for each dose with 1 mL being given in each deltoid. All injections must be administered as soon as possible after preparation by the pharmacy.

When preparing the VSV_{IN} HIV *gag* or placebo dose in 2 syringes and administering the dose, care should be taken to avoid aerosol production. Consideration should be given to the volume of solution in each needle before and after the dose is administered. Particularly, if the needles used to withdraw the product are replaced prior to vaccine administration, consideration should be given to conserving the full dose of product. The pharmacy and clinic staff members are encouraged to work together to administer the dose specified in the protocol.

8.5 Acquisition of study products

All active study products will be provided by Profectus BioSciences, Inc. The placebo (Sodium Chloride for Injection, USP 0.9%) will not be provided through the protocol but must be purchased by the site.

The TriGrid™ Delivery System and TriGrid™ Application Cartridge (electroporation array), as well as the 3 mL syringes (BD #309585) and B-D 22 Ga.x1½" needle (BD

#305156) for Months 0, 1 and 3 only, will be provided by Ichor Medical Systems. All other syringes used in preparation of the study products will not be provided through the protocol and must be purchased by the site.

Once an HVTN CRS is protocol registered, the pharmacist can obtain study products, including the TriGrid™ Application Cartridge from the NIAID Clinical Research Products Management Center (CRPMC) by following the ordering procedures given in *Pharmacy Guidelines and Instructions for DAIDS Clinical Trials Networks.* The TriGrid™ Delivery System will be shipped directly to the sites by Ichor Medical Systems, Inc.

8.6 Pharmacy records

The HVTN CRS pharmacist is required to maintain complete records of all study products. The pharmacist of record is responsible for maintaining randomization codes and randomization confirmation notices for each participant in a secure manner.

8.7 Final disposition of study products

All unused study products must be returned to the CRPMC after the study is completed or terminated unless otherwise instructed. The procedures and relevant form are included in the *Pharmacy Guidelines and Instructions for DAIDS Clinical Trials Networks*.

9 Clinical procedures

The schedule of clinical procedures is shown in Appendix G and Appendix H.

9.1 Informed consent

Informed consent is the process of ensuring that participants fully understand what will and may happen to them while participating in a research study. The HVTN informed consent form documents that a participant (1) has been informed about the potential risks, benefits, and alternatives to participation, and (2) is willing to participate in an HVTN study. Informed consent encompasses all written or verbal study information HVTN CRS staff provide to the participant, before and during the trial. HVTN CRS staff will obtain informed consent of participants according to HVTN policies and procedures.

The informed consent process continues throughout the study. Key study concepts should be reviewed periodically with the participant and the review should be documented. At each study visit, HVTN CRS staff should consider reviewing the procedures and requirements for that visit and for the remaining visits. Additionally, if any new information is learned that might affect the participants' decisions to stay in the trial, this information will be shared with trial participants. If necessary, participants will be asked to sign revised informed consent forms.

An HVTN CRS may employ recruitment efforts prior to the participant consenting. For example, some HVTN CRSs use a telephone script to prescreen people before they come to the clinic for a full screening visit. Participants must sign a screening or protocol-specific consent before any procedures are performed to determine eligibility. HVTN CRSs must submit recruitment and prescreening materials to IRBs/ECs for human subjects protection review and approval.

9.1.1 Screening consent form

Some HVTN CRSs have approval from their local IRB or EC to use a general screening consent form that allows screening for an unspecified HIV vaccine trial. In this way, HVTN CRS staff can continually screen potential participants and, when needed, proceed quickly to obtain protocol-specific enrollment consent. Sites conducting IRB/EC-approved general screening or prescreening may use the results from this screening to determine eligibility for this protocol, provided the tests are conducted within the time periods specified in the eligibility criteria. Without a general screening consent, screening for a specific study cannot take place until the site is activated by HVTN Regulatory Affairs.

9.1.2 Protocol-specific consent form

The protocol-specific consent form describes the study products to be used and all aspects of protocol participation, including screening and enrollment procedures. A sample protocol-specific consent form is located in Appendix A.

Each HVTN CRS is responsible for developing a protocol-specific consent form for local use, based on the sample protocol-specific consent form in Appendix A. The consent form must be developed in accordance with local IRB/EC requirements and the principles

of informed consent as described in Title 45, Code of Federal Regulations (CFR) Part 46 and Title 21 CFR, Part 50, and in the International Conference on Harmonisation (ICH) E6, Good Clinical Practice: Consolidated Guidance 4.8.

Study sites are strongly encouraged to have their local CABs review their site-specific consent form. This review should include, but should not be limited to, issues of cultural competence, local language considerations, and the level of understandability.

The sample informed consent form includes instructions throughout the document for developing specific content.

Prior to implementing version 1 of this protocol, each site must have the protocol and site-specific protocol consent form(s) approved by its IRB/EC and any other applicable Regulatory Entity (RE). Prior to site activation, site-specific informed consent forms will be reviewed and approved by the DAIDS Protocol Registration Office (PRO) and/or HVTN Regulatory Affairs.

Regarding protocol registration, sites should follow procedures outlined in the current version of the DAIDS Protocol Registration Manual.

9.1.3 VISP registry consent form

Experimental HIV vaccines may induce antibody production to HIV antigens, producing reactive results on commercially available HIV test kits. This is called “vaccine-induced seropositivity” (VISP) (see Section 9.7.1). In order to provide post-study HIV testing to distinguish between VISP and HIV infection, and to mitigate potential social harms resulting from VISP in HIV vaccine recipients who are not infected with HIV, the HVTN has created a VISP registry. Following study unblinding, the registry will allow HVTN counselors to verify that an individual has received an HIV vaccine, and therefore has the potential for VISP. Information in the VISP registry will not be used for research. Rather, the registry exists to support provision of post-study testing and counseling services to HIV vaccine recipients.

The VISP registry consent form describes the purpose of the VISP registry, the participant information to be included in the registry, confidentiality protections, and risks and benefits associated with inclusion in the registry. The VISP registry consent form is contained in Appendix C.

The VISP Registry consent form will be presented to all participants. It is recommended to be presented no later than the last scheduled vaccination visit.

9.1.4 Assessment of Understanding

Study staff are responsible for ensuring that participants fully understand the study before enrolling them. This process involves reviewing the informed consent form with the participant, allowing time for the participant to reflect on the procedures and issues presented, and answering all questions completely.

An Assessment of Understanding is used to document the participant’s understanding of key concepts in this HIV vaccine trial. The participant must complete the Assessment of Understanding before enrollment. Staff may provide assistance in reading and

understanding the questions and responses, if necessary. Participants must verbalize understanding of all questions answered incorrectly. This process and the participant's understanding of the key concepts should be recorded in source documentation at the site.

IRBs/ECs may require that a participant has signed either a screening or protocol-specific consent document prior to administering the Assessment of Understanding. The consent process (including the use of the Assessment of Understanding) should be explained thoroughly to the IRB/EC, whose recommendations should be followed.

9.2 Pre-enrollment procedures

Screening may occur over the course of several contacts/visits, up to and including before vaccination on day 0. All inclusion and exclusion criteria must be assessed within 56 days before enrollment, unless otherwise specified in the eligibility criteria (or below in this section).

After the appropriate informed consent has been obtained and before enrollment, the following procedures are performed:

- Medical history, documented in the case history record;
- Deltoid skin fold measurement (see Section 8.4and *HVTN 087 Study Specific Procedures*)
- Assessment of whether the volunteer is at low risk for HIV infection;
- Complete physical examination, including height, weight, vital signs, and clinical assessments of head, ears, eyes, nose, and throat; neck; lymph nodes; heart; chest; abdomen; extremities; neurological function; and skin;
- Mini-Mental State Examination
- Assessment of concomitant medications the volunteer is taking, including prescription and nonprescription drugs, vitamins, topical products, alternative/complementary medicines (eg, herbal and health food supplements), recreational drugs, vaccinations, and allergy shots (record the complete generic name for all medications);
- Laboratory tests as defined in the inclusion and exclusion criteria, including:
  - Screening HIV test,
  - HBsAg,
  - Anti-HCV,
  - Syphilis test,
  - Complete blood count (CBC) with differential and platelets,
  - Chemistry panel (ALT, AST, alkaline phosphatase, creatinine, and CPK),

- Urine dipstick (as described in Section 9.8),
- Urine or serum pregnancy test (participants who were born female),

- Administration of behavioral risk assessment questionnaire;

- Obtaining of volunteer demographics in compliance with the *NIH Policy on Reporting Race and Ethnicity Data: Subjects in Clinical Research,* Aug. 8, 2001 (available at http://grants.nih.gov/grants/guide/notice-files/NOT-OD-01-053.html);

- Counseling on HIV testing and risk reduction, performed in compliance with the US Centers for Disease Control and Prevention (CDC)'s current guidelines or other local guidelines for HIV counseling, testing, and referral as described in Section 9.7; and

- Discussion of pregnancy prevention. A pregnant or breastfeeding person may not be enrolled in this trial. Specific criteria and assessment of contraception and pregnancy status are described in study inclusion criteria. Discussion of pregnancy prevention includes advising a participant who was born female and who reports no current sexual activity that could lead to that participant becoming pregnant to have a plan to begin adequate birth control. This plan would be put to use if, during the study, the participant becomes sexually active in a way that could lead to that participant becoming pregnant.

9.2.1 Use of screening results from another HVTN study

If a participant screens for an HVTN study at the same HVTN CRS but then does not join that study, screening results from that effort may be applied to the screening for this protocol, as long as the screening was done under participant consent, the participant has signed a consent form to begin screening for this study, and the tests were conducted within the time periods specified in the eligibility criteria (see Sections 7.1 and 7.2).

9.3 Enrollment and vaccination visits

Enrollment is simultaneous with first vaccination. The time interval between randomization and enrollment should not exceed 4 working days. The HVTN CRS registers the participant by scheduling the day 0 visit (enrollment) via the Web-based randomization system, and requests the randomization assignment. Circumstances may require a participant's enrollment visit to be changed. This may exceed the 4-day randomization time limit.

At all vaccination visits, the following procedures are performed before vaccination:

- Abbreviated physical examination, including weight, vital signs, and a symptom-directed evaluation by history and/or appropriate physical exam based on participant self-reported symptoms or complaints;

- Assessment of baseline reactogenicity parameters;

- Assessment of concomitant medications (as described in Section 9.2);
- Assessment of any new or unresolved AEs/intercurrent illnesses; and
- Urine or serum pregnancy test (for participants who were born female).

Following completion of all procedures in the preceding list and results indicate that vaccination may proceed, vaccination is prepared and administered (see Sections 8.3 and 8.4).

Immediately following vaccination, the participant remains in the clinic for observation. An initial reactogenicity assessment is made at a target of 30 minutes after injection, with an acceptable range of 25-60 minutes. Before leaving the clinic, the participant is given the postvaccination symptom log and is instructed on how to complete it. The site will make arrangements to obtain daily reports of reactogenicity events from the participant during the reactogenicity period (as described in Section 9.10).

The following procedures will be performed at all vaccination visits. These procedures may be performed prior to or following vaccination:

- Risk reduction counseling (as described in Section 9.7);
- Pregnancy prevention assessment (as described in Sections 9.2 and 9.8); and
- Assessment of new or unresolved social impacts (site staff will ask participant about the status of any unresolved social impacts and if s/he has experienced any new social impacts as a result of the trial participation).

The following procedure will be performed at all vaccination visits. This procedure will be performed following vaccination:

- Visual Analog Scale (pain assessment)

Additional procedures will be performed at scheduled visits as specified in Appendix G and Appendix H:

- Confirm that participants received HIV test results from previous visit. If not, provide test results and post-test counseling as appropriate; and
- Specimen collection (should be completed prior to vaccination)
- Mini Mental State Examination

9.3.1 Procedures for device-only participants

If the participant was not enrolled because the Ichor TDS EP device was applied to the participant's arm but the participant was not able to receive any injection of study product at that visit, other procedures specified for the visit (with the exceptions of pain assessment using the VAS, and postvaccination reactogenicity assessments) should be completed and data collected should be reported to the SDMC using the appropriate CRFs (see Section 11.2.2)

The CRS staff should contact each device-only participant approximately 14 days after initial application of the device in order to assess any new or unresolved AEs that may have occurred in the interim. This contact does not require a clinic visit, unless medically indicated. As the device-only participant was not enrolled in the trial, no further visits or study procedures are required, except for AE reporting of events associated with the application of the EP device, which should be reported to the SDMC on the appropriate CRF. In addition, AEs requiring expedited reporting should be reported to the DAIDS RSC Safety Office as described in Section 11.2.3.

9.4 Follow-up visits

The following procedures are performed at all scheduled follow-up visits:

- Assessment of new or continuing concomitant medications (as described in Section 9.2); and
- Assessment of new or unresolved AEs/intercurrent illnesses.

Additional procedures will be performed at scheduled follow-up visits as specified in Appendix G and Appendix H.

- Risk reduction counseling (as described in Section 9.7);
- Pregnancy prevention assessment (as described in Sections 9.2 and 9.8);
- Assessment of new or unresolved social impacts (site staff will ask participant about the status of any unresolved social impacts and if s/he has experienced any new social impacts as a result of the trial participation);
- Administration of the social impact assessment questionnaire (types of impacts assessed involve personal relationships, health insurance, life insurance, educational or employment opportunities, housing, immigration, or travel);
- Administration of a questionnaire that asks the participant about any HIV testing he or she may have received outside of the study. Participants will also be asked whether they believe they received the active vaccine or the control;
- HIV infection assessment including pre-test counseling. A subsequent follow-up contact is conducted to provide post-test counseling and to report results to participant;
- Confirm that participants received HIV test results from previous visit. If not, provide test results and post-test counseling as appropriate;
- Abbreviated physical examination including weight, vital signs, and a symptom-directed evaluation by history and/or appropriate physical exam based on participant self-reported symptoms or complaints;

- Complete physical examination, including weight, vital signs, and clinical assessments of head, ears, eyes, nose, and throat; neck; lymph nodes; heart; chest; abdomen; extremities; neurological function; and skin;
- Administration of acceptability questionnaire
- Specimen collection;
- Clinical laboratory tests including:
  - CBC with differential and platelet count,
  - T-cell subsets,
  - Chemistry panel (see Section 9.2), and
  - Urine dipstick (urinalysis if appropriate; see Section 9.9);
- Urine or serum pregnancy test (for participants who were born female); and
- Mini Mental State Examination

9.5 Month 15 health contact

CRS staff will contact study participants at their Month 15 timepoints to collect the information listed below. Clinic visits will only be required if HIV confirmatory testing is necessary (see Section 9.7.1); however, a clinic visit may be arranged for other reasons.

Confirmation of vital status; if deceased, attempt to learn cause and date of death

If participant is alive, record the participant's responses to questions regarding any occurrence of the following events since the last HVTN study contact:

- Assessment of new or continuing concomitant medications (as described in Section 9.2); and
- Assessment of new or unresolved AEs/intercurrent illnesses
  - Life threatening adverse experiences;
  - Persistent or significant disability/incapacity;
  - Hospitalizations and reasons;
  - Other important medical events that may jeopardize the participant or may require intervention to prevent 1 of the other outcomes listed above;
  - New chronic conditions requiring more than 30 days of medical intervention or medication;
  - AESI
  - New diagnosis of HIV infection; and
  - Pregnancies and outcomes, including congenital anomalies/birth defects.

All such events will be recorded and adverse events will be assessed for relationship to study products. Other questions may be added by the HVTN 087 Protocol Team for exploratory endpoints.

9.6 Annual health contacts

Participants will be contacted annually for a total of 3 years following initial study injection (see Appendix H). At these contacts, CRS staff will collect the information listed below. Clinic visits will only be required if HIV confirmatory testing is necessary (see Section 9.7.1); however, a clinic visit may be arranged for other reasons.

- Confirmation of vital status; if deceased, attempt to learn cause and date of death;
- If participant is alive, record the participant’s responses to questions regarding any occurrence of the following events since the last HVTN study contact:
    - Life-threatening adverse experiences;
    - Persistent or significant disability/incapacity;
    - Hospitalizations and reasons;
    - Other important medical events that may jeopardize the participant or may require intervention to prevent 1 of the other outcomes listed above;
    - New chronic conditions requiring more than 30 days of medical intervention or medication;
    - AESI
    - New diagnosis of HIV infection; and
    - Pregnancies and outcomes, including congenital anomalies/birth defects.

All such events will be recorded, and adverse events will be assessed for relationship to study product(s).

9.6.1 Interim contacts

CRSs may report safety information obtained at a contact other than the annual contact. These contacts are reported as interim visits.

9.7 HIV counseling and testing

HIV counseling will be performed in compliance with the CDC’s guidelines or other local guidelines for HIV counseling and referral. HIV testing will be performed in accordance with the current HVTN HIV testing algorithm following enrollment.

Participants will be counseled routinely during the trial on the avoidance of HIV infection and on the potential negative social impacts of testing antibody positive due to the vaccine. They will also be counseled on the risks of HIV antibody testing outside of the HVTN CRSs and will be discouraged from doing so during study participation and/or during any period of vaccine-induced positive serology.

Study staff will take particular care to inform study participants of the likelihood of routine HIV testing being offered or performed outside the study CRS at emergency rooms, clinics, and medical offices. Such testing has become more likely due to the CDC's revised guidelines for HIV counseling and testing, as well as policy changes in many countries to make HIV testing more frequent and routine. CRS staff should inform participants of their right to opt out of HIV testing outside the study site. CRS staff should inform study participants if local and/or state policies and regulations permit medical providers to perform HIV testing without first informing patients. If this is the case, then CRS staff should advise study participants that they may decline testing preemptively. CRS staff should inform participants if positive results must be reported to local public health authorities. CRS staff should also inform participants of the need to maintain study blinding by getting HIV testing only at the study CRS. CRS staff should provide participants with CRS contact information and should encourage participants to ask medical providers to contact the CRS. The CRS can verify that the participant is in an HIV vaccine clinical trial and should only be tested at the study CRS.

Potential participants identified as being HIV infected during screening are not enrolled. All participants who become HIV infected during the study will be terminated from this study. Potential and enrolled participants identified as HIV infected will be referred for medical treatment, counseling, and management of the HIV infection. These individuals may also be referred to appropriate ongoing clinical trials or observational studies.

9.7.1 Distinguishing intercurrent HIV infection from vaccine-induced positive serology

The study products may elicit an antibody response to HIV proteins. Therefore, vaccine-induced positive serology may occur in this study. Several precautionary measures will be taken to distinguish intercurrent HIV infection from vaccine-induced positive serology. These precautionary measures include:

- Participants will have physical examinations at visits specified in Appendix G. Signs or symptoms of an acute HIV infection syndrome, an intercurrent illness consistent with HIV-1 infection, or probable HIV exposure would prompt a diagnostic workup per the HVTN algorithm for Recent Exposure/Acute Infection Testing to determine HIV infection.

- HIV testing will be performed at multiple timepoints throughout the study (see Appendix E and Appendix F). The Laboratory Program (or approved diagnostic laboratory) will follow the HVTN HIV testing algorithm (as described in the *HVTN Site Lab Reference Manual*), which is able to distinguish vaccine-induced antibody responses from actual HIV infections.

- All participants can receive HIV-1 diagnostic testing from the site following their last scheduled visit until they are told that they did not receive an HIV vaccine or that they do not have vaccine induced seropositivity.

- All participants who received vaccine product and who have vaccine-induced positive or indeterminate HIV-1 serology (as measured by the standard anti-HIV antibody screening tests) at or after the study is unblinded will be offered poststudy HIV-1 diagnostic testing (per the HVTN poststudy HIV-1 testing algorithm) periodically and free of charge as medically/socially indicated (approximately every 6 months).

9.8 Contraception status

Contraception status is routinely assessed and documented at clinic visits, for a participant who was born female and who is sexually active in a way that could cause that participant to become pregnant. Prior to enrollment and throughout the study, staff will ask participants to verbally confirm their use of adequate contraceptive methods. A participant who was born female and is sexually active in a way that could cause that participant to become pregnant should be reminded at all scheduled clinic visits of the importance of using contraception and should be referred to specific counseling, information, and advice as needed. (Specific contraception requirements are listed in Section 7.1.) This reminder should be documented in the participant's study record.

Self-reported infertility—including having reached menopause (no menses for 1 year) or having undergone hysterectomy, bilateral oophorectomy, or tubal ligation—must be documented in the participant's study record.

9.9 Urinalysis

Dipstick testing may be performed in the clinic or the lab, as long as the required elements (glucose, protein, and hemoglobin) are tested. The examination is performed on urine obtained by clean catch.

If the screening dipstick is transiently abnormal due to menses or infection, document this issue in the participant's source documentation. For infection, provide appropriate treatment and/or referral. Following resolution, repeat the dipstick and, if within the eligibility limits specified in the protocol, the participant may be enrolled.

Follow-up urinalysis should be deferred if a participant is menstruating, but should be performed as soon as possible. If a follow-up dipstick is abnormal due to a participant's menstrual period, document in the comment section of the CRF and repeat the dipstick once the participant is no longer menstruating. A micro-urinalysis is not required.

9.10 Assessments of reactogenicity

For all participants, baseline assessments are performed before and reactogenicity assessments are performed after each vaccination. All reactogenicity symptoms are followed until resolution and graded according to the *Division of AIDS Table for Grading the Severity of Adult and Pediatric Adverse Events* (DAIDS AE Grading Table), Version 1.0, December 2004 (Clarification August 2009).

The reactogenicity assessment period is 3 full days following each HIV-MAG vaccine/placebo injection and 7 full days following the VSV HIV *gag* vaccine/placebo injection per the assessment schedule shown in Table 9-1. Participants are instructed to record symptoms using a postvaccination symptom log and to contact the site daily during the assessment period. Clinic staff will follow new or unresolved reactogenicity symptoms present at the end of the assessment period to resolution. Participants are instructed to contact the clinic for events that arise during the period between vaccination and the next scheduled visit. In general, a participant who self-reports any

postvaccination reaction greater than mild is seen by a clinician within 48 hours after onset, unless the reaction is improving and/or has completely resolved.

Reactogenicity events are reported using CRFs that correspond to the time of assessment in Table 9-1. Reactogenicity assessments include assessments of systemic and local symptoms, vaccine-related lesions, and lymph nodes. Events not listed on a CRF, or with an onset after the reactogenicity assessment period, or those meeting SAE/adverse events requiring expedited reporting to DAIDS criteria, are recorded on an adverse experience log form.

Table 9-1 Schedule of reactogenicity assessments

Day	Time	Performed by
0[a]	Reactogenicity baseline: before vaccination	HVTN CRS staff
	VAS immediate: post vaccination	HVTN CRS staff
	VAS 5-7 minutes: post vaccination	HVTN CRS staff
	VAS 25-60 minutes: post vaccination	HVTN CRS staff
	Reactogenicity early: post vaccination	HVTN CRS staff
1	Reactogenicity between 12:00 AM and 11:59 PM day 1	HVTN CRS staff or participant
2	Reactogenicity between 12:00 AM and 11:59 PM day 2	HVTN CRS staff or participant
3[b]	Reactogenicity between 12:00 AM and 11:59 PM day 3	HVTN CRS staff or participant
4-7[b]	Reactogenicity between 12:00 am and 11:59 pm days 4-7, **for VSV HIV *gag* vaccine/placebo only (VAC 4)**	HVTN CRS staff or participant

[a] Day of vaccination

[b] New or unresolved reactogenicity symptoms present at the end of the assessment period are followed until resolution

9.10.1 Assessment of systemic and local symptoms

Systemic symptoms include increased body temperature, malaise and/or fatigue, myalgia, headache, chills, arthralgia, nausea, and vomiting. Local symptoms include pain and/or tenderness proximal to the injection site. The daily maximum severity reached for each symptom during the assessment period is reported.

Body temperature is measured by oral or infrared thermometry and reported in degrees Celsius. If temperature is measured in Fahrenheit, the conversion to Celsius should be documented in the participant's chart note. A measurement is taken once daily during the assessment period and should be repeated if participant is feeling feverish.

9.10.2 Assessment of injection site

Typical injection site reactions are erythema/induration/swelling/edema. The maximum horizontal and maximum vertical measurements for all injection site reactions are recorded.

All injection site reactions are monitored until resolution. Areas greater than 25 cm^2 are followed daily; otherwise, the frequency of follow-up is based on clinician judgment.

9.10.3 Assessment of lymph nodes

This assessment is required only when reactogenicity assessments are performed by HVTN CRS staff, not by the participant.

Only the proximally draining lymph nodes are assessed (eg, axillary nodes on the same side of the body for injections given in the deltoid). Lymph nodes are first evaluated for enlargement and tenderness. If they are found to be enlarged, measurements are taken to determine the size (widest diameter) of the enlarged node(s).

9.11 Assessment of neurological safety

As a precaution, specific inclusion and exclusion criteria have been included in this protocol to minimize the chances of enrolling anyone with pre-existing neurological or neuropsychiatric disease that could be confused with a neurological adverse effect related to rVSV vaccination. The Mini Mental State Examination [47] will also be used to monitor participant safety. Any participant who develops symptoms or findings suggestive of possible encephalitis or a neurological adverse event related to rVSV vaccination should be referred appropriately to a neurologist or other physician for consultation and care. The site should request permission from the participant for access to medical records related to the evaluation. Information about participants who are evaluated or referred for neurological symptoms or findings that could be related to rVSV vaccination should be reported by phone to the SDMC Clinical Affairs staff within 24 hours and followed by the customary email and fax transmissions.

9.12 Assessment for generalized rVSV infection

Participants who report symptoms of a systemic viral syndrome such as fever, chills, myalgias, and nausea up to 7 days post-vaccination at visit 11 with VSV HIV *gag* vaccine or placebo will be assessed with urine, blood, and saliva collection for rVSV viral infectivity assay. The assays will be performed on frozen, batched samples (see Section 10.8.1).

9.13 Visit windows and missed visits

Visit windows are defined in *HVTN* 087 *Study Specific Procedures*. For a visit not performed within the window period, a Missed Visit form is completed. If the missed visit is one that required safety assessments or local safety labs, HVTN CRS staff should attempt to bring the participant in for an interim visit as soon as possible.

Procedures performed at an interim visit are usually toxicity/safety assessments (including local safety labs) and HIV testing. With the exception of HIV testing, these procedures are performed only if they were required at the missed visit or if clinically indicated. HIV testing may be performed as deemed appropriate by the study staff. Blood samples for immunogenicity assays are not typically collected at interim visits.

If a missed visit required vaccination, please refer to Section 7.3.3 and Section 7.3.4 for resolution.

9.14 Early termination visit

In the event of early participant termination, site staff should consider if the following assessments are appropriate: a final physical examination, clinical laboratory tests (including urine dipstick, CBC with differential, platelet count, and chemistry panel), pregnancy testing, social impact assessment, and HIV test.

9.15 Pregnancy

If a participant becomes pregnant during the course of the study, no more injections of study product will be given but remaining visits and study procedures should be completed unless medically contraindicated. If the participant terminates from the study prior to the pregnancy outcome, the site should make every effort to keep in touch with the participant in order to ascertain the pregnancy outcome.

10 Laboratory

10.1 HVTN CRS laboratory procedures

The *HVTN Site Lab Reference Manual* provides further guidelines for operational issues concerning the clinical and processing laboratories. The manual includes guidelines for general specimen collection; special considerations for phlebotomy, specimen labeling, whole blood processing, HIV screening/diagnostic testing, and general screening and safety testing.

Tube types for blood collection are specified in Appendix E and Appendix F. For tests performed locally, the local lab may assign appropriate tube types.

In specific situations, the blood collection tubes may be redirected to another laboratory or may require study-specific processing techniques. In these cases, laboratory special instructions will be posted on the protocol-specific section of the HVTN website.

10.2 Total blood volume

Required blood volumes per visit are shown in Appendix E and Appendix F. Not shown is any additional blood volume that would be required if a safety lab needs to be repeated, or if a serum pregnancy test needs to be performed. The additional blood volume would likely be minimal. The total blood volume drawn for each participant will not exceed 500 mL in any 56-day (8-week) period.

10.3 Primary immunogenicity timepoints

The primary immunogenicity timepoints in this study are at visits 10 (day 98) and 15 (day 182) (ie, 2 weeks after the third and fourth vaccination visits). Endpoint assays for humoral and cellular responses are performed on participants at the primary immunogenicity timepoints and may be performed at baseline. Depending on the number of responders observed, assays for humoral and cellular responses may be performed on participants at other timepoints; the schedule is shown in Appendix E and Appendix F.

10.4 Endpoint assays: cellular

10.4.1 Flow cytometry

Flow cytometry will be used to examine vaccine-specific CD4+ and CD8+ T-cell responses following stimulation of PBMC with synthetic HIV peptides that span the proteins encoded by the vaccine construct. Intracellular cytokine staining (ICS) parameters will include cytokines such as IFN- γ, IL-2, and TNF-α, and may include other cytokines to identify T cells of specific functionality (such as Th2 and Th17). Markers of cytotoxic potential (Granzyme B, perforin and CD57) may also be included. Data will be reported as percentages of CD4+ or CD8+ T cells responding to a specific peptide pool. Additional cell surface markers, cytokines, or functional markers may also be analyzed.

10.4.2 IFN-γ ELISpot

If supplemental cellular immunogenicity data for the study vaccines are needed, IFN-γ ELISpot may be performed. PBMC will be stimulated overnight with synthetic peptide pools that span the proteins encoded by the vaccine constructs. This process will allow ex vivo HIV-specific T-cell data to be assessed by IFN-γ ELISpot as an immunogenicity endpoint. Data will be reported as the number of SFC per 10^6 cells recognizing a specific peptide pool.

10.5 Endpoint assays: humoral

10.5.1 HIV-1 multiplex antibody assay

Total binding IgG (IgG1, IgG2, IgG3, IgG4) and IgA antibodies to HIV-1 HXB2 Gag p55 and HIV-1 6101 Env gp160 will be assessed on plasma samples from study participants taken at the primary immunogenicity timepoint and baseline. Specimens from other timepoints as well as other HIV antigens may also be assayed based on the results of the initial assay.

10.5.2 IL-12 binding antibodies by ELISA

As an exploratory analysis, the induction of binding antibodies to human IL-12 will be assessed by ELISA by Profectus on serum samples collected at baseline and at 2 weeks following the fourth vaccination. Serum collected at 2 weeks following the third vaccination may also be assessed. Binding antibodies to human IL-12 will be monitored in all study participants for safety.

10.5.3 IL-12 neutralizing antibody assay

If anti-human IL-12 binding antibodies are detected by the IL-12 binding Ab ELISA, those samples will be tested for neutralizing activity against human IL-12.

10.6 Innate immunity assays

Innate immunity assays will be performed on samples from participants in Groups 1 and 3.

10.6.1 Soluble factors in serum

Multiplex cytokine bead array and/or enzyme-linked immunosorbent assay (ELISA) may be used to measure soluble cytokines, chemokines, and other immunomodulatory factors in the serum or plasma. Analytes may include IFN-γ, IL-6, TNF- α, IL-10, IP-10, and/or MCP-1. Other analytes may also be included.

10.6.2 Enumerations and phenotyping of cell populations

Phenotyping of DCs, monocytes, NK cells, B cells, T cells or other leukocytes for lineage, maturation and activation markers may be performed on cryopreserved PBMC. Absolute cell counts will be determined by obtaining a CBC including a differential cell count from a clinical laboratory. Trucount tubes will be used when possible for direct

enumeration of major leukocyte populations in the blood, including DC, by flow cytometry. Alternatively, cryopreserved PBMC from the same blood draw may be examined by flow cytometry and absolute counts of cell types of interest will be determined based on the absolute lymphocyte count derived from the CBC. Data will be reported as cell concentrations per µl of blood and as percent of cells positive for each marker at the various timepoints.

10.6.3 RNA gene expression

Whole blood or bulk PBMC will be cryopreserved in an RNA protection reagent. RNA will be isolated and may be used for microarray analysis and/or real-time PCR. Signatures of gene expression changes for different cell types will be analyzed over time after vaccination. Data will be reported as fold change over baseline pre-vaccine expression.

10.6.4 Natural Killer (NK)-cell phenotyping and function

NK-cell phenotyping by flow cytometry may be conducted. Markers will include NK-cell receptors of the KIR, NCR and FcR families. In addition, a flow-based assay assessing NK cell function will be utilized. This assay determines the NK cells' ability to respond to stimuli delivered through the different classes of NK receptors (e.g. KIR, NCR and FcR) by assessing NK cell degranulation (CD107 upregulation) as well as the secretion of cytokines and chemokines (especially MIP-1).

10.7 Genotyping

Molecular human leukocyte antigen (HLA) typing may be performed on enrolled participants using cryopreserved PBMC collected at baseline, initially on specimens from participants who demonstrate vaccine-induced T-cell responses at postvaccination timepoints. Other participants (including control recipients) may be HLA-typed to support future studies of immunological interest at the discretion of the HVTN Laboratory Program. Other markers, single nucleotide polymorphisms (SNPs) linked with genes associated with immune responses or HIV-1 disease progression may also be assessed.

10.8 Exploratory studies

These samples will be used for other testing and research related to furthering the understanding of HIV pathogenesis or vaccines. In addition, cryopreserved samples may be used to perform additional assays to support standardization and validation of existing or newly developed methods.

10.8.1 Vector viral infectivity assay

As an exploratory analysis, the presence of replication competent VSV that may be present in the blood, urine, and saliva will be examined for any participant with symptoms of a systemic viral syndrome, up to 7 days postvaccination with rVSV/placebo. Vero cell cultures will be incubated with participant samples and examined by microscopy for the presence of VSV-induced cytopathic effects. If

cytopathic effects are observed, VSV titrations will be determined by a plaque assay and supernatants of positive samples will be further cultured to examine the induction of cytopathic effects. Nucleotide sequence analysis will be performed to verify the presence of VSV. Vector viral infectivity assays will be performed retrospectively on frozen specimens.

10.9 Other use of stored specimens

The HVTN aims not only to test vaccine candidates but also to continue to explore the correlates of immunity to HIV. In order to do so, the HVTN intends to store blood samples from participants. These samples will be used for other testing and research related to furthering the understanding of virology, immunology, or vaccinology to the extent authorized in each study site's informed consent form, or as otherwise authorized under applicable law. Other testing on specimens will only occur, at a minimum, after review and approval by the HVTN and the IRB of the researcher requesting the specimens.

The protocol sample informed consent form is written so that the participant either explicitly allows or does not allow sample storage for other research when he or she signs the form. Participants who initially agree to other use of their samples may rescind their approval once they enter the study; such participants will still remain in this study. If a participant decides against allowing other research using his or her samples, or at any time rescinds prior approval for such other use, the study site investigator or designee must notify HVTN Regulatory Affairs in writing. In either case, after database lock, the HVTN Laboratory Program will request that the repository destroy all specimens with the participant identification numbers (PTIDs) of all participants who do not agree to other use of their samples. HVTN Core will report the destruction of relevant specimens to the participants' site Principal Investigators (PIs).

Study sites must notify HVTN Regulatory Affairs if institutional or local governmental requirements pose a conflict with or impose restrictions on the use of stored specimens.

10.10 Biohazard containment

As the transmission of HIV and other blood-borne pathogens can occur through contact with contaminated needles, blood, and blood products, appropriate precautions will be employed by all personnel in the drawing of blood and shipping and handling of all specimens for this study, as currently recommended by the CDC and the NIH or other locally appropriate agencies.

All dangerous goods materials, including Biological Substances, Category A or Category B, must be transported according to instructions detailed in the International Air Transport Association Dangerous Goods Regulations.

11 Safety monitoring and safety review

11.1 Safety monitoring and oversight

11.1.1 HVTN 087 PSRT

The HVTN 087 PSRT is composed of the following members:

- DAIDS medical officer representative,
- Protocol chair and cochair,
- Protocol Team leader,
- Core medical monitor, and
- SDMC Clinical Affairs safety associate.

The clinician members of the HVTN 087 PSRT are responsible for decisions related to participant safety.

The Protocol Team clinic coordinator, project manager, vaccine developer representative, clinical trial manager, and others may also be included in HVTN 087 PSRT meetings.

11.1.2 HVTN SMB

The SMB is a multidisciplinary group consisting of biostatisticians, clinicians, and experts in HIV vaccine research that, collectively, has experience in the conduct and monitoring of vaccine trials. Members of the SMB are not directly affiliated with the protocols under review.

The SMB reviews safety data, unblinded as to treatment arm, approximately every 4 months. The reviews consist of evaluation of cumulative reactogenicity events, AE, laboratory safety data, and individual reports of adverse events requiring expedited reporting to DAIDS. To increase the sensitivity for detecting potential safety problems, the SMB will review safety data aggregated across multiple protocols that use the same or similar vaccine candidates. The SMB conducts additional special reviews at the request of the HVTN 087 PSRT.

Study sites will receive SMB summary minutes and are responsible for forwarding them to their local IRB/ECs.

11.1.3 SDMC roles and responsibilities in safety monitoring

The roles and responsibilities of the SDMC in relation to safety monitoring include:

- Maintaining a central database management system for HVTN clinical data;
- Providing reports of clinical data to appropriate groups such as the HVTN 087 PSRT and HVTN SMB (see Section 11.1.2);

- Daily monitoring of clinical data for events that meet the safety pause and HVTN 087 PSRT AE review criteria (see Section 11.4);
- Notifying HVTN CRSs and other groups when safety pauses or planned holds are instituted and lifted (see Section 11.4);
- Querying HVTN CRSs for additional information regarding reported clinical data; and
- Providing support to the HVTN 087 PSRT.

11.2 Safety reporting

11.2.1 Submission of safety forms to SDMC

Sites must submit all safety forms (eg, reactogenicity, adverse experience, urinalysis, local lab results, concomitant medications) before the end of the next business day after receiving the information. The forms should not be held in anticipation of additional information at a later date. If additional information is received at a later date, the forms should be updated and refaxed before the end of the next business day after receiving the new information.

11.2.2 AE reporting

An AE is any untoward medical occurrence in a clinical investigation participant administered a study product/procedure(s) and which does not necessarily have a causal relationship with this treatment. An AE can therefore be any unfavorable and unintended sign (including an abnormal laboratory finding), symptom, or disease temporally associated with the use of an investigational study product/procedure(s), whether or not related to the investigational study product/procedure(s). All AEs are graded according to the *Division of AIDS Table for Grading the Severity of Adult and Pediatric Adverse Events* (DAIDS AE Grading Table), Version 1.0, December 2004 (Clarification August 2009), available on the RSC website at http://rsc.tech-res.com/safetyandpharmacovigilance/, except that unintentional weight loss of less than 10% loss in body weight from baseline is not required to be reported as an adverse event.

All AEs are reported to the SDMC on the appropriate CRF. Clinic staff should evaluate every AE to determine if (1) the AE meets the requirements for expedited reporting to DAIDS (Section 11.2.3) and (2) if the AE meets the criteria for a safety pause/prompt AE review (Section 11.4).

Autoimmune disorders are AEs of special interest (AESI). A sample list of AESI is provided in Appendix J.AESI must be reported during the entire period of participation including during annual health contacts.

Sites are expected to notify SDMC Clinical Affairs staff of any serious safety concern requiring their attention (see Table 11-1). Telephone numbers and email addresses are listed in the Key Resource Guide of the *HVTN 087 Study Specific Procedures*. Concerns requiring immediate attention should be communicated by calling the SDMC Clinical Affairs safety phone.

In the case of email notification, SDMC Clinical Affairs staff will reply during working hours (US Pacific Time) to confirm that the email has been received and reviewed. If email service is not available, the HVTN CRS should notify SDMC Clinical Affairs of the event by telephone, then submit CRFs.

In addition, site investigators are required to submit AE information in accordance with local regulatory agencies' or other local authorities' requirements.

11.2.3 Expedited reporting of adverse events to DAIDS

Requirements, definitions, and methods for expedited reporting of AEs are outlined in Version 2.0 dated January 2010 of *The Manual for Expedited Reporting of Adverse Events to DAIDS* (DAIDS EAE Manual), which is available on the RSC website: http://rsc.tech-res.com/safetyandpharmacovigilance/. The SAE Reporting Category will be used for this study.

The internet-based DAIDS Adverse Event Reporting System (DAERS) must be used for expedited AE reporting to DAIDS. In the event of system outages or technical difficulties, expedited AE reports may be submitted via the DAIDS EAE Form. For questions about DAERS, please contact DAIDS-ES at DAIDS-ESSupport@niaid.nih.gov or from within the DAERS application itself.

Sites where DAERS has not been implemented will submit expedited AE reports by documenting the information on the current DAIDS EAE Form. This form is available on the RSC website: http://rsc.tech-res.com/safetyandpharmacovigilance/. For questions about expedited AE reporting, please contact the RSC (DAIDSRSCSafetyOffice@tech-res.com).

The study products for which expedited reporting are required are:

- HIV-MAG or placebo
- GENEVAX® *IL-12*-4532 or placebo
- VSV_{IN} HIV gag or placebo
- TDS EP device

While the participant is in the main study reporting period (see Section 3), the SAE Reporting Category will be used.

If the participant has completed the main study and is in the "annual health contacts reporting period" (see Section 3) the SUSAR Reporting Category will be used (In addition, please note, per Section 9.5, all adverse events that are serious are collected on the LTFU Event Log and reported to SCHARP.)

After the participant has completed the annual health contacts period and is off study, sites must report SUSARS if the study site staff becomes aware of the events on a passive basis (eg, from publicly available information).

The NIAID/DAIDS will report all unexpected SAEs related to the study products observed in this clinical trial to the FDA in accordance with 21 CFR 312.32 (IND Safety Reports). However, because safety is a primary study endpoint, the Sponsor Medical Officer will not be unblinded to study treatment assignment when there is an assessment of relatedness of the SAE with the study products; and the safety report will be sent to the FDA based on the blinded attribution assessment.

If the PSRT believes unblinding of the site PI to treatment assignment will assist with the clinical management of the SAE, the PSRT will consult the independent HVTN SMB for a recommendation. In the event the HVTN SMB determines that unblinding is indicated, the SMB will inform the site physician of the participant's treatment assignment in such a manner as to maintain the study blind of the PSRT and study team. For additional impact and management of SAEs on the study, refer to Section 11.4.

11.3 Safety reviews

11.3.1 Initial safety evaluation

Groups 1, 2, and 3 will be opened to enrollment simultaneously. Groups 1 and 3 share the same visit schedule and will be randomized together. Group 2 will be randomized separately, so that participants who are not available for the frequent visits required for Groups 1 and 3 may also participate. Enrollment across all participating HVTN clinical research sites (CRSs) will be restricted to a maximum of 1 participant per day until 15 participants have been enrolled overall, in any group(s). Enrollment will then be held until the PSRT reviews available safety, tolerability, and acceptability data through day 14 for those 15 participants. If these data are acceptable, enrollment may then proceed for Groups 1-3. It is possible that these first 15 participants would all be enrolled in Group 2. In that case, the low rate of accrual in Groups 1 and 3 would indicate that those groups are enrolling participants at a very gradual rate.

11.3.2 Safety evaluation for proceeding to Group 4

Prior to enrollment of Group 4, which tests the highest dose of IL-12 pDNA, the PSRT will review all cumulative safety and tolerability data available from at least 24 participants enrolled in Groups 1 and 3 (12 in each group), and all 25 participants in Group 2, through day 14. If these data are acceptable, enrollment may then proceed for Group 4, with a maximum of 1 Group 4 participant per day across all participating HVTN CRSs until 5 participants have been enrolled. Enrollment for Group 4 will then be held until the PSRT reviews available safety, tolerability, and acceptability data through day 14 for those participants. If these data are acceptable, enrollment may then proceed for Group 4.

11.3.3 Safety evaluation prior to VSV boost

Cumulative safety data from the rVSV phase 1 trial, HVTN 090, will be reviewed by the PSRT prior to the initiation of VSV HIV gag boost injections in this study. The dose for use in this study must be considered to have an acceptable safety profile in HVTN 090.

11.4 Safety pause and prompt PSRT AE review

When a trial is placed on safety pause, all enrollment and vaccination with the product related to the event that triggered the pause will be held until further notice. The AEs that will lead to a safety pause or prompt HVTN 087 PSRT AE review are summarized in Table 11-1. Vaccinations may be suspended for safety concerns other than those described in the table, or before pause rules are met, if, in the judgment of the HVTN 087 PSRT, participant safety may be threatened. Criteria for an individual participant's departure from the schedule of vaccinations are listed in Section 7.3.

Table 11-1 AE notification and safety pause/AE review rules

Event and relationship to study products/procedure	Severity	HVTN CRS action	SDMC action
SAE, related	Grade 5 or Grade 4	Phone immediately, email and fax forms immediately[1]	Immediate pause
SAE, not related	Grade 5	Phone immediately, email and fax forms immediately	Immediate HVTN 087 PSRT notification
SAE, related	Grade 3	Email and fax forms immediately	Prompt HVTN 087 PSRT AE review to consider pause
AE[2], related	Grade 4 or 3	Email and fax forms immediately	Prompt HVTN 087 PSRT AE review to consider pause

For all safety pauses, the SDMC Clinical Affairs staff notifies the HVTN 087 PSRT, HVTN Regulatory Affairs, DAIDS Pharmaceutical Affairs Branch (PAB), DAIDS Regulatory Affairs Branch (RAB), DAIDS Safety and Pharmacovigilance Team (SPT), and participating HVTN CRSs. When an immediate safety pause is triggered, the SDMC Clinical Affairs staff also notifies the HVTN SMB.

Once a trial is paused, the HVTN 087 PSRT reviews safety data and decides whether the pause can be lifted or permanent discontinuation of vaccination is appropriate, consulting the SMB if necessary. SDMC Clinical Affairs staff notifies the participating HVTN CRSs, HVTN Regulatory Affairs, DAIDS PAB, DAIDS RAB, and DAIDS SPT of the decision regarding resumption or discontinuation of study vaccinations. Based on the HVTN 087 PSRT assessment, DAIDS RAB notifies the FDA as needed.

If an immediate HVTN 087 PSRT notification or prompt HVTN 087 PSRT AE review is triggered, the SDMC Clinical Affairs staff notifies the HVTN 087 PSRT as soon as possible during working hours (US Pacific Time)—or, if the information was received during off hours, by the morning of the next work day. If a prompt HVTN 087 PSRT AE review cannot be completed within 72 hours of SDMC notification (excluding weekends and US federal holidays), an automatic safety pause occurs.

Each HVTN CRS is responsible for submitting to its IRB/EC and any local regulatory authority protocol-related safety information (such as IND safety reports, notification of vaccine holds due to the pause rules, etc), as required.

In addition, all other AEs are reviewed routinely by the HVTN 087 PSRT (see Section 11.5.2).

[1] Phone numbers and email addresses are listed in HVTN 087 Study Specific Procedures, Key Resource Guide.

[2] Does not include subjective reactogenicity symptoms (injection site pain, tenderness, fatigue/malaise, myalgia, arthralgia, chills, headache, nausea).

11.5 Review of cumulative safety data

Routine safety review occurs at the start of enrollment and then throughout the study.

Reviews proceed from a standardized set of protocol-specific safety data reports. These reports are produced by the SDMC and include queries to the HVTN CRSs. Events are tracked by internal reports until resolution.

11.5.1 Daily review

Blinded daily safety reviews are routinely conducted by the SDMC Clinical Affairs staff for events requiring expedited reporting to DAIDS, and events that meet safety pause criteria or prompt PSRT AE review criteria.

11.5.2 Weekly review

During the injection phase of the trial, the SDMC Clinical Affairs staff and the HVTN 087 PSRT review clinical safety reports on a weekly basis and conduct calls to review the data as appropriate. After the injections and the final 2-week safety visits are completed, less frequent reporting and safety reviews may be conducted at the discretion of the HVTN 087 PSRT. The SDMC Clinical Affairs staff reviews reports of clinical and laboratory AEs. Events identified during the review that are considered questionable, inconsistent, or unexplained are referred to the HVTN CRS clinic coordinator for verification.

11.6 Study termination

This study may be terminated early by the determination of the HVTN 087 PSRT, HVTN SMB, FDA, NIH, Office for Human Research Protections (OHRP), or vaccine developer. In addition, the conduct of this study at an individual HVTN CRS may be terminated by the determination of the local IRB or EC, or of the appropriate local or national regulatory authority.

12 Protocol conduct

This protocol and all actions and activities connected with it will be conducted in compliance with the principles of GCP (ICHe6) and according to DAIDS and HVTN policies and procedures as specified in the HVTN *Manual of Operations* and DAIDS *Clinical Research Policies and Standard Procedures Documents*, including procedures for the following:

- Protocol registration, activation, and implementation;
- Informed consent, screening, and enrollment;
- Study participant reimbursement;
- Clinical and safety assessments;
- Safety monitoring and reporting;
- Data collection, documentation, transfer, and storage;
- Participant confidentiality;
- Study follow-up and close-out;
- Unblinding of staff and participants;
- Quality control;
- Protocol monitoring and compliance;
- Advocacy and assistance to participants regarding negative social impacts associated with the vaccine trial;
- Risk reduction counseling; and
- Specimen collection, processing, and analysis.

Any policies or procedures that vary from DAIDS and HVTN standards or require additional instructions (eg, instructions for randomization specific to this study) will be described in the HVTN 087 *Study Specific Procedures*.

12.1 Social impacts

Participants in this study risk experiencing discrimination or other personal problems, resulting from the study participation itself or from the development of VISP. The HVTN CRS is obliged to provide advocacy for and assistance to participants regarding these negative social impacts associated with the vaccine trial. If HVTN CRS staff have questions regarding ways to assist a participant dealing with a social impact, a designated NIAID or HVTN Core representative can be contacted.

Social harms are tabulated by the SDMC and are subjected to descriptive analysis. The goal is to reduce their incidence and enhance the ability of study staff to mitigate them when possible.

Summary tables of social impact events will be generated weekly and made available for review by the protocol chairs, protocol team leader, and the designated NIAID representative.

12.2 Compliance with NIH guidelines for research involving products containing recombinant DNA

Because this study is evaluating products containing recombinant DNA, it must comply with regulations set forth in the NIH's *Guidelines for Research Involving Recombinant DNA Molecules*. Information about the study must be submitted to site Institutional Biosafety Committees (IBC) and must be approved before participants are enrolled at the respective institution. Investigators at each site are responsible for obtaining IBC approval and periodic review of the research per NIH guidelines *section IV-B07-b-(6)* and *section IV-B-2-b.* IBC review and approval must be documented by the investigator and submitted as part of protocol registration for this trial.

The NIH guidelines also require that human gene transfer trials conducted at or sponsored by institutions that receive NIH funds must be submitted to the NIH Office of Biotechnology Activities (OBA) for review by the Recombinant DNA Advisory Committee (RAC). The NIH guidelines create exceptions to RAC review, but the HVTN 087 Protocol Team determined that the exceptions did not apply. Therefore, the Protocol Team, jointly with Profectus Biosciences, Inc., submitted the application with the study concept proposal for RAC review and responded to RAC comments. The application will follow the guidance provided in the NIH Guidelines.

The HVTN and DAIDS will ensure that reporting requirements to RAC, as outlined in *Appendix M-I-C-1.Initiation of the Clinical Investigation*, *Appendix M-I-C-3.Annual Reports*, and *Appendix M-I-C-4.Safety Reporting* are satisfied per the NIH Guidelines.

12.3 Emergency communication with study participants

As in all clinical research, this study may generate a need to reach participants quickly to avoid imminent harm, or to report study findings that may otherwise concern their health or welfare.

When such communication is needed, the CRS will request that its IRB/EC expedite review of the message. If this review cannot be completed in a timeframe consistent with the urgency of the required communication, the site should contact the participant first, and then notify the IRB/EC of the matter as soon as possible.

13 Version history

The Protocol Team may modify the original version of the protocol. Modifications are made to HVTN protocols via clarification memos, letters of amendment, or full protocol amendments.

The table below describes the version history of, and modifications to, Protocol HVTN 087.

Protocol history and modifications

Date	Protocol version	Protocol modification	Comment
27-DEC-11	Version 1	Original protocol	

14 Document references (other than literature citations)

Other documents referred to in this protocol, and containing information relevant to the conduct of this study, include:

- Assessment of Understanding. Accessible through the HVTN protocol-specific website.
- Current CDC Guidelines. *Revised Recommendations for HIV Testing of Adults, Adolescents, and Pregnant Women in Health-Care Settings.* Available at http://www.cdc.gov/mmwr/PDF/rr/rr5514.pdf.
- *Division of AIDS (DAIDS) Clinical Research Policies and Standard Procedures Documents*. Available at http://www3.niaid.nih.gov/research/resources/DAIDSClinRsrch/
- *Division of AIDS Protocol Registration Manual*. Available at http://www.niaid.nih.gov/LabsAndResources/resources/DAIDSClinRsrch/Documents/prmanual.pdf
- *Division of AIDS Table for Grading the Severity of Adult and Pediatric Adverse Events*. Version 1.0, December 2004 (Clarification August 2009). Available at http://rsc.tech-res.com/safetyandpharmacovigilance/.
- *The Manual for Expedited Reporting of Adverse Events to DAIDS*. Version 2.0, January 2010. Available at http://rsc.tech-res.com/safetyandpharmacovigilance/.
- *HVTN Certificate of Confidentiality*. Accessible through the HVTN website.
- *HVTN 087 Special Instructions*. Accessible through the HVTN protocol-specific website.
- *HVTN 087 Study Specific Procedures.* Accessible through the HVTN protocol-specific website.
- *HVTN Site Lab Reference Manual*. Accessible through the HVTN website.
- *HVTN Manual of Operations*. Accessible through the HVTN website.
- HVTN algorithm for diagnosis of HIV infections. Part of the *HVTN Site Lab Reference Manual* (see above).
- International Conference on Harmonisation (ICH) E6 (R1), *Guideline for Good Clinical Practice*: section 4.8, *Informed consent of trial subjects*. Available at http://www.emea.europa.eu/pdfs/human/ich/013595en.pdf.
- *Participants' Bill of Rights and Responsibilities.* Accessible through the HVTN website.
- *NIH Guidelines for Research Involving Recombinant DNA Molecules*. Available at http://oba.od.nih.gov/rdna/nih_guidelines_oba.html.

- *NIH Policy on Reporting Race and Ethnicity Data: Subjects in Clinical Research*. Available at http://grants1.nih.gov/grants/guide/notice-files/NOT-OD-01-053.html.

- *Pharmacy Guidelines and Instructions for DAIDS Clinical Trials Networks*, July 2008.

- *Requirements for Source Documentation in DAIDS Funded and/or Sponsored Clinical Trials.* Available at http://www3.niaid.nih.gov/research/resources/DAIDSClinRsrch/ClinicalSite.htm

- Title 21, Code of Federal Regulations, Part 50. Available at http://www.access.gpo.gov/nara/cfr/waisidx_08/21cfrv1_08.html.

- Title 45, Code of Federal Regulations, Part 46. Available at http://www.access.gpo.gov/nara/cfr/waisidx_07/45cfrv1_07.html.

See Section 16 for literature cited in this protocol.

15 Acronyms and abbreviations

ADARC	Aaron Diamond AIDS Research Center
AE	adverse event
AESI	adverse events of special interest
ALT	alanine aminotransferase
ANOVA	analysis of variance
AST	aspartate aminotransferase
AVEG	AIDS Vaccine Evaluation Group
BGH	bovine growth hormone
β-HCG	beta human chorionic gonadotropin
BMI	body mass index
CAB	Community Advisory Board
CBC	complete blood count
CDC	US Centers for Disease Control and Prevention
CFR	Code of Federal Regulations
CIOMS	Council for International Organizations of Medical Sciences
CPK	creatine phosphokinase
CRF	case report form
CRPMC	NIAID Clinical Research Products Management Center
CRS*	clinical research site
CT	cytoplasmic tail
CTL	cytotoxic T lymphocyte
DAERS	DAIDS Adverse Event Reporting System
DAIDS	Division of AIDS (US NIH)
DHHS	US Department of Health and Human Services
DSMB	NIAID Data and Safety Monitoring Board
EAE	adverse events requiring expedited reporting to DAIDS
EC	Ethics Committee
EDTA	ethylenediamine tetraacetic acid
ELISA	enzyme-linked immunosorbent assay
ELISpot	enzyme-linked immunospot
EP	electroporation
FDA	US Food and Drug Administration
FHCRC	Fred Hutchinson Cancer Research Center
GCP	Good Clinical Practice
GEE	generalized estimating equation
GLP	good laboratory practice
hCMV	human cytomegalovirus
HCV	hepatitis C virus

HIV	human immunodeficiency virus
HLA	human leukocyte antigen
HSV	herpes simplex virus
HVTN	HIV Vaccine Trials Network
IB	Investigator's Brochure
IBC	Institutional Biosafety Committee
IC	intracranial
ICH	International Conference on Harmonisation
ICS	intracellular cytokine staining
IFN-γ	interferon gamma
IM	intramuscular
IND	Investigational New Drug
IRB	Institutional Review Board
IT	intrathalamic
IUD	intrauterine device
IV	intravenous
LTFU	loss to follow-up
MAR	missing at random
MCAR	missing completely at random
MMR	measles, mumps, and rubella
MTD	maximum tolerated dose
nAb	neutralizing antibody
NHP	nonhuman primate
NIAID	National Institute of Allergy and Infectious Diseases (US NIH)
NIH	US National Institutes of Health
NV	neurovirulence
OBA	NIH Office of Biotechnology Activities
OHRP	US Office for Human Research Protections
OPV	oral polio vaccine
PAB	DAIDS Pharmaceutical Affairs Branch
PBMC	peripheral blood mononuclear cells
PCR	polymerase chain reaction
PFU	plaque-forming unit
PI	Principal Investigator
PID	pelvic inflammatory disease
PSRT	Protocol Safety Review Team
PTE	potential T-cell epitope
qPCR	quantitative polymerase chain reaction
RAB	DAIDS Regulatory Affairs Branch
RAC	NIH Recombinant DNA Advisory Committee
RSC	DAIDS Regulatory Support Center

SAE	serious adverse event
SCHARP	Statistical Center for HIV/AIDS Research and Prevention
sCMV	simian cytomegalovirus
SDMC	statistical and data management center
SFC	spot-forming cells
SIV	simian immunodeficiency virus
SMB	Safety Monitoring Board
SPT	DAIDS Safety and Pharmacovigilance Team
TB	tuberculosis
TDS	TriGrid™ Delivery System (Ichor Medical Systems, Inc.)
TU	transcriptional unit
UW-VSL	University of Washington Virology Specialty Laboratory
VAS	visual analog scale
VISP	vaccine induced seropositivity
VRC	Vaccine Research Center (NIAID)
VSV	vesicular stomatitis virus
WBC	white blood cell
wt	wild type

[*] CRSs were formerly referred to as HIV Vaccine Trial Units (HVTUs). Conversion to use of the term CRS is in process, and some HVTN documents may still refer to HVTUs.

16 Literature cited

1. Council for International Organizations of Medical Sciences(CIOMS). International ethical guidelines for biomedical research involving human subjects. Bull Med Ethics **2002**;17-23.

2. UNAIDS. Ethical considerations in HIV preventive vaccine research. **2002**.

3. The National Commission for the Protection of Human Subjects of Biomedical and Behavioral Research. The Belmont Report: Ethical Principles and Guidelines for the Protection of Human Subjects of Research. **1979**.

4. Amara RR, Villinger F, Altman JD, Lydy SL, O'Neil SP, Staprans SI, Montefiori DC, Xu Y, Herndon JG, Wyatt LS, Candido MA, Kozyr NL, Earl PL, Smith JM, Ma HL, Grimm BD, Hulsey ML, Miller J, McClure HM, McNicholl JM, Moss B, Robinson HL. Control of a mucosal challenge and prevention of AIDS by a multiprotein DNA/MVA vaccine. Science **2001**;292:69-74.

5. Shiver JW, Fu TM, Chen L, Casimiro DR, Davies ME, Evans RK, Zhang ZQ, Simon AJ, Trigona WL, Dubey SA, Huang L, Harris VA, Long RS, Liang X, Handt L, Schleif WA, Zhu L, Freed DC, Persaud NV, Guan L, Punt KS, Tang A, Chen M, Wilson KA, Collins KB, Heidecker GJ, Fernandez VR, Perry HC, Joyce JG, Grimm KM, Cook JC, Keller PM, Kresock DS, Mach H, Troutman RD, Isopi LA, Williams DM, Xu Z, Bohannon KE, Volkin DB, Montefiori DC, Miura A, Krivulka GR, Lifton MA, Kuroda MJ, Schmitz JE, Letvin NL, Caulfield MJ, Bett AJ, Youil R, Kaslow DC, Emini EA. Replication-incompetent adenoviral vaccine vector elicits effective anti-immunodeficiency-virus immunity. Nature **2002**;415:331-5.

6. Luckay A, Sidhu MK, Kjeken R, Megati S, Chong SY, Roopchand V, Garcia-Hand D, Abdullah R, Braun R, Montefiori DC, Rosati M, Felber BK, Pavlakis GN, Mathiesen I, Israel ZR, Eldridge JH, Egan MA. Effect of plasmid DNA vaccine design and in vivo electroporation on the resulting vaccine-specific immune responses in rhesus macaques. J Virol **2007**;81:5257-69.

7. Egan MA, Megati S, Roopchand V, Garcia-Hand D, Luckay A, Chong SY, Rosati M, Sackitey S, Weiner DB, Felber BK, Pavlakis GN, Israel ZR, Eldridge JH, Sidhu MK. Rational design of a plasmid DNA vaccine capable of eliciting cell-mediated immune responses to multiple HIV antigens in mice. Vaccine **2006**;24:4510-23.

8. Kiepiela P, Ngumbela K, Thobakgale C, Ramduth D, Honeyborne I, Moodley E, Reddy S, de Pierres C, Mncube Z, Mkhwanazi N, Bishop K, van der SM, Nair K, Khan N, Crawford H, Payne R, Leslie A, Prado J, Prendergast A, Frater J, McCarthy N, Brander C, Learn GH, Nickle D, Rousseau C, Coovadia H, Mullins JI, Heckerman D, Walker BD, Goulder P. CD8+ T-cell responses to different HIV proteins have discordant associations with viral load. Nat Med **2007**;13:46-53.

9. Sacha JB, Chung C, Rakasz EG, Spencer SP, Jonas AK, Bean AT, Lee W, Burwitz BJ, Stephany JJ, Loffredo JT, Allison DB, Adnan S, Hoji A, Wilson NA, Friedrich TC, Lifson JD, Yang OO, Watkins DI. Gag-specific CD8+ T lymphocytes recognize infected cells before AIDS-virus integration and viral protein expression. J Immunol **2007**;178:2746-54.

10. Aihara H, Miyazaki J. Gene transfer into muscle by electroporation in vivo. Nat Biotechnol **1998**;16:867-70.

11. Gehl J, Mir LM. Determination of optimal parameters for in vivo gene transfer by electroporation, using a rapid in vivo test for cell permeabilization. Biochem Biophys Res Commun **1999**;261:377-80.

12. Gothelf A, Mir LM, Gehl J. Electrochemotherapy: results of cancer treatment using enhanced delivery of bleomycin by electroporation. Cancer Treat Rev **2003**;29:371-87.

13. Cemazar M, Golzio M, Sersa G, Rols MP, Teissie J. Electrically-assisted nucleic acids delivery to tissues in vivo: where do we stand? Curr Pharm Des **2006**;12:3817-25.

14. Favard C, Dean DS, Rols MP. Electrotransfer as a non viral method of gene delivery. Curr Gene Ther **2007**;7:67-77.

15. van Drunen Littel-van den Hurk, Hannaman D. Electroporation for DNA immunization: clinical application. Expert Rev Vaccines **2010**;9:503-17.

16. Liu J, Kjeken R, Mathiesen I, Barouch DH. Recruitment of antigen-presenting cells to the site of inoculation and augmentation of human immunodeficiency virus type 1 DNA vaccine immunogenicity by in vivo electroporation. J Virol **2008**;82:5643-9.

17. Boyer JD, Cohen AD, Ugen KE, Edgeworth RL, Bennett M, Shah A, Schumann K, Nath B, Javadian A, Bagarazzi ML, Kim J, Weiner DB. Therapeutic immunization of HIV-infected chimpanzees using HIV-1 plasmid antigens and interleukin-12 expressing plasmids. AIDS **2000**;14:1515-22.

18. Schadeck EB, Sidhu M, Egan MA, Chong SY, Piacente P, Masood A, Garcia-Hand D, Cappello S, Roopchand V, Megati S, Quiroz J, Boyer JD, Felber BK, Pavlakis GN, Weiner DB, Eldridge JH, Israel ZR. A dose sparing effect by plasmid encoded IL-12 adjuvant on a SIVgag-plasmid DNA vaccine in rhesus macaques. Vaccine **2006**;24:4677-87.

19. Boyer JD, Robinson TM, Kutzler MA, Parkinson R, Calarota SA, Sidhu MK, Muthumani K, Lewis M, Pavlakis G, Felber B, Weiner D. SIV DNA vaccine co-administered with IL-12 expression plasmid enhances CD8 SIV cellular immune responses in cynomolgus macaques. J Med Primatol **2005**;34:262-70.

20. Schnell MJ, Buonocore L, Whitt MA, Rose JK. The minimal conserved transcription stop-start signal promotes stable expression of a foreign gene in vesicular stomatitis virus. J Virol **1996**;70:2318-23.

21. Johnson JE, Schnell MJ, Buonocore L, Rose JK. Specific targeting to CD4+ cells of recombinant vesicular stomatitis viruses encoding human immunodeficiency virus envelope proteins. J Virol **1997**;71:5060-8.

22. Rose NF, Marx PA, Luckay A, Nixon DF, Moretto WJ, Donahoe SM, Montefiori D, Roberts A, Buonocore L, Rose JK. An effective AIDS vaccine based on live attenuated vesicular stomatitis virus recombinants. Cell **2001**;106:539-49.

23. Geisbert TW, Daddario-Dicaprio KM, Lewis MG, Geisbert JB, Grolla A, Leung A, Paragas J, Matthias L, Smith MA, Jones SM, Hensley LE, Feldmann H, Jahrling PB. Vesicular stomatitis virus-based ebola vaccine is well-tolerated and protects immunocompromised nonhuman primates. PLoS Pathog **2008**;4:e1000225.

24. Van Rompay KK, Abel K, Earl P, Kozlowski PA, Easlick J, Moore J, Buonocore-Buzzelli L, Schmidt KA, Wilson RL, Simon I, Moss B, Rose N, Rose J, Marthas ML. Immunogenicity of viral vector, prime-boost SIV vaccine regimens in infant rhesus macaques: attenuated vesicular stomatitis virus (VSV) and modified vaccinia Ankara (MVA) recombinant SIV vaccines compared to live-attenuated SIV. Vaccine **2010**;28:1481-92.

25. Flanagan EB, Zamparo JM, Ball LA, Rodriguez LL, Wertz GW. Rearrangement of the genes of vesicular stomatitis virus eliminates clinical disease in the natural host: new strategy for vaccine development. J Virol **2001**;75:6107-14.

26. Gherardi MM, Ramirez JC, Esteban M. Interleukin-12 (IL-12) enhancement of the cellular immune response against human immunodeficiency virus type 1 env antigen in a DNA prime/vaccinia virus boost vaccine regimen is time and dose dependent: suppressive effects of IL-12 boost are mediated by nitric oxide. J Virol **2000**;74:6278-86.

27. Yamanaka H, Hoyt T, Yang X, Golden S, Bosio CM, Crist K, Becker T, Maddaloni M, Pascual DW. A nasal interleukin-12 DNA vaccine coexpressing Yersinia pestis F1-V fusion protein confers protection against pneumonic plague. Infect Immun **2008**;76:4564-73.

28. Jacobson MA, Sinclair E, Bredt B, Agrillo L, Black D, Epling CL, Carvidi A, Ho T, Bains R, Girling V, Adler SP. Safety and immunogenicity of Towne cytomegalovirus vaccine with or without adjuvant recombinant interleukin-12. Vaccine **2006**;24:5311-9.

29. Hirao LA, Wu L, Khan AS, Hokey DA, Yan J, Dai A, Betts MR, Draghia-Akli R, Weiner DB. Combined effects of IL-12 and electroporation enhances the potency of DNA vaccination in macaques. Vaccine **2008**;26:3112-20.

30. Graham BS, Koup RA, Roederer M, Bailer RT, Enama ME, Moodie Z, Martin JE, McCluskey MM, Chakrabarti BK, Lamoreaux L, Andrews CA, Gomez PL, Mascola JR, Nabel GJ. Phase 1 Safety and Immunogenicity Evaluation of a Multiclade HIV-1 DNA Candidate Vaccine. J Infect Dis **2006**;194:1650-60.

31. Klinman DM, Klaschik S, Tross D, Shirota H, Steinhagen F. FDA guidance on prophylactic DNA vaccines: Analysis and recommendations. Vaccine **2009**.

32. Johnson JE, Coleman JW, Kalyan NK, Calderon P, Wright KJ, Obregon J, Ogin-Wilson E, Natuk RJ, Clarke DK, Udem SA, Cooper D, Hendry RM. In vivo biodistribution of a highly attenuated recombinant vesicular stomatitis virus expressing HIV-1 Gag following intramuscular, intranasal, or intravenous inoculation. Vaccine **2009**;27:2930-9.

33. Vasan S, Hurley A, Schlesinger SJ, Hannaman D, Gardiner DF, Dugin DP, Boente-Carrera M, Vittorino R, Caskey M, Andersen J, Huang Y, Cox JH, Tarragona-Fiol T, Gill DK, Cheeseman H, Clark L, Dally L, Smith C, Schmidt C, Park HH, Kopycinski JT, Gilmour J, Fast P, Bernard R, Ho DD. In vivo electroporation enhances the immunogenicity of an HIV-1 DNA vaccine candidate in healthy volunteers. PLoS One **2011**;6:e19252.

34. Dolter KE, Evans CF, Ellefsen B, Song J, Boente-Carrera M, Vittorino R, Rosenberg TJ, Hannaman D, Vasan S. Immunogenicity, safety, biodistribution and persistence of ADVAX, a prophylactic DNA vaccine for HIV-1, delivered by in vivo electroporation. Vaccine **2011**;29:795-803.

35. Scofield RH, Harley JB. Autoantigenicity of Ro/SSA antigen is related to a nucleocapsid protein of vesicular stomatitis virus. Proc Natl Acad Sci U S A **1991**;88:3343-7.

36. Huang SC, Pan Z, Kurien BT, James JA, Harley JB, Scofield RH. Immunization with vesicular stomatitis virus nucleocapsid protein induces autoantibodies to the 60 kD Ro ribonucleoprotein particle. J Investig Med **1995**;43:151-8.

37. Hardgrave KL, Neas BR, Scofield RH, Harley JB. Antibodies to vesicular stomatitis virus proteins in patients with systemic lupus erythematosus and in normal subjects. Arthritis Rheum **1993**;36:962-70.

38. Routsias JG, Sakarellos-Daitsiotis M, Detsikas E, Tzioufas AG, Sakarellos C, Moutsopoulos HM. Antibodies to EYRKK vesicular stomatitis virus-related peptide account only for a minority of anti-Ro60kD antibodies. Clin Exp Immunol **1994**;98:414-8.

39. Rose NF, Roberts A, Buonocore L, Rose JK. Glycoprotein exchange vectors based on vesicular stomatitis virus allow effective boosting and generation of neutralizing antibodies to a primary isolate of human immunodeficiency virus type 1. J Virol **2000**;74:10903-10.

40. Symmons DP. Review of UK data on the rheumatic diseases--8. SLE. Br J Rheumatol **1991**;30:288-90.

41. Agresti A, Coull BA. Approximate is better than "exact" for interval estimation of binomial proportions. Am Stat **1998**;52:199-256.

42. Bechhofer RE, Santner TJ, Goldsman DM. Design and analysis of experiments for statistical selection, screening, and multiple comparisons. New York: John Wiley & Sons, **1995**.

43. Hudgens MG. Estimating cumulative probabilities from incomplete longitudinal binary responses with application to HIV vaccine trials. Statistics in Medicine **2003**;22:463-79.

44. Lachenbruch PA. Comparisons of two-part models with competitors. Stat Med **2001**;20:1215-34.

45. Hughes JP. Mixed effects models with censored data with application to HIV RNA levels. Biometrics **1999**;55:625-9.

46. Rotnitzky A, Robins J. Analysis of semi-parametric regression models with non-ignorable non-response. Stat Med **1997**;16:81-102.

47. Folstein MF, Folstein SE, Mchugh PR. Mini-Mental State - Practical Method for Grading Cognitive State of Patients for Clinician. Journal of Psychiatric Research **1975**;12:189-98.

Appendix A Sample informed consent form

Title: A phase 1 trial to evaluate the safety, tolerability, and immunogenicity *of* an IL-12 pDNA enhanced HIV-1 multiantigen pDNA vaccine delivered intramuscularly with electroporation, with an HIV-1 rVSV vaccine boost, in healthy HIV-uninfected **adult participant**

Short title: HVTN 087

Site: [Insert site name]

Thank you for your interest in our research study. Please read this consent form or ask someone to read it to you. If you decide to join the study, we will ask you to sign this form. We will offer you a copy to keep. We will ask you questions to see if we have explained everything clearly. You can also ask us questions about the study. This will help you decide whether or not to join, or whether to continue in the study.

Research is not the same as treatment or medical care. The purpose of a research study is to answer scientific questions.

About the study

The HIV Vaccine Trials Network (HVTN) and [Insert site name] are conducting a research study to test two HIV vaccines. HIV is the virus that causes AIDS.

About 100 people will take part in this study at multiple sites. The researcher in charge of this study at this clinic is [Insert name of site PI]. The US National Institutes of Health (NIH) is paying for the study.

The study tests two vaccines, another study product called an adjuvant, and an experimental procedure called electroporation (EP), to try to improve the body's response to one of the vaccines. We will define these terms in a later section.

1. We are doing this study to answer several questions.

- Are the study products safe to give to people?
- Are people able to take the study products without becoming too uncomfortable?
- Are people able to have electroporation with study products without becoming too uncomfortable?
- How do people's immune systems respond to the study products? (Your immune system protects you from disease.)
- Do different doses of the study adjuvant have different effects?

2. The study vaccines cannot give you HIV.

The study vaccines are not made from actual HIV. It is impossible for the study vaccines to give you HIV. Also, they cannot cause you to give HIV to someone else.

3. We do not know if the study vaccines will decrease, increase, or not change your chance of becoming infected with HIV if you are exposed to the virus.

Sites: Any change to the language in this section requires approval from HVTN Regulatory Affairs.

Several studies have tested whether HIV vaccines can reduce the risk of getting HIV from another person. In some studies, people who got the vaccine seemed to have the *same* risk of getting HIV as people who did not get the vaccine. In one study, people who got the vaccine seemed to have a *lower* risk of getting HIV than people who did not get the vaccine. In another study, some men who got the vaccine had a *higher* risk of getting HIV than men who did not get the vaccine.

This study differs from the studies in which people who got vaccine had a higher or lower risk of getting HIV. The study staff can tell you about the differences.

We do not know whether the vaccines in this study will affect your risk of getting HIV from another person. The risk could be higher, lower, or unchanged. It's very important to avoid exposure to HIV during and after the study. We will tell you how to avoid HIV.

4. These study products are experimental.

The study products are called HIV-MAG vaccine, VSV HIV *gag* vaccine, and *IL-12* pDNA adjuvant. From here on, we will call them HIV-MAG vaccine and VSV vaccine or "the study vaccines" and *IL-12* or "the study adjuvant". The study vaccines are experimental HIV vaccines. That means we do not know whether they will be safe to use in people, or whether they will work to prevent HIV infection. These vaccines are used only in research studies.

The study products are provided by Profectus Biosciences, Inc.

This is the first time the study vaccines will be given together to people. They have been tested together in mice, rabbits, and monkeys without causing health problems. Even if something looks like it is safe or works in animals, that may not be true for people.

The HIV-MAG vaccine and *IL-12* pDNA are currently being tested together with EP in another study of up to 60 people. They are being given to people already infected with HIV. As of July 2011, 3 people in that study have been given the HIV-MAG and *IL-12* pDNA, using the same EP device that will be used in this study. In yet another study, *IL-12* has been tested in 30 people with a different EP device. In addition 146 people have received *IL-12* without EP. Before this study begins, the VSV vaccine will have been tested in another study, including 10 people who got the vaccine at the same dose being tested in this study. Based on the safety results and responses to the study products seen in these studies, researchers feel it is appropriate to continue studying these products in a larger group of people, and to test them together.

HIV-MAG vaccine is a DNA vaccine which contains DNA made in the laboratory. DNA is a natural substance in the body that tells the body to make proteins. Proteins are natural substances that the body uses to build and maintain itself as well as protect itself against disease. The DNA in the study vaccine tells the body to make a few proteins that are found in HIV called Env, Gag, Pol, Nef, Tat and Vif. Your body's immune system may

respond to these proteins by making cells that recognize and fight against these types of HIV protein.

The idea behind this vaccine is to show your body's immune system some of the HIV proteins so that your body will recognize and fight real HIV if your body ever sees HIV. This is the way other vaccines work to fight other diseases.

Sometimes vaccines work better when they are combined with another substance that helps to alert the immune system. These substances are called adjuvants. In this study, the HIV-MAG vaccine will be tried by itself and also in combination with an adjuvant called *IL-12*. It is an experimental adjuvant used only in research studies. The study adjuvant will be tested at 3 doses. The study adjuvant is made of DNA that will tell your body to make IL-12, a normal protein in the body that helps immune cells work together and makes them multiply. The study adjuvant is given with the HIV-MAG vaccine to help the body's immune system better respond to the study vaccine.

The VSV vaccine is made from a virus called vesicular stomatitis virus (VSV) serotype Indiana.

In nature, VSV is carried by insects and transmitted to animals by insect bites. Infected animals may get a rash or sores at the location of the insect bite, around the mouth, or on their hooves, which go away within 10-12 days. Infected livestock and wild animals may then infect each other. Farmers, veterinarians and other people may catch VSV from living or working closely with infected animals, or from working with VSV in a laboratory. In North America human infection with VSV is rare. When humans are infected with VSV, they can have symptoms of fever, muscle aches, headaches, fatigue and generally feeling unwell or in some cases may experience no symptoms of infection at all.

The VSV in the study vaccine has been weakened with genetic changes so that it does not cause the health problems seen in animals. It is extremely unlikely that someone who receives the study vaccine can pass VSV to someone else.

A virus distantly related to VSV has caused encephalitis in people. Encephalitis is a brain disease which can include changes in mental state, such as confusion, drowsiness, seizures, and loss of consciousness. There is only one known case of someone with encephalitis that was likely caused by VSV serotype Indiana. Encephalitis is not at all expected with the weakened virus used to make this vaccine. But just to be safe, we will check your mental status several times during this study.

The VSV HIV *gag* vaccine carries a gene which tells the body to make a protein called Gag that is found in HIV. Your body's immune system may respond to the Gag protein by making cells that recognize Gag, which could fight against Gag found in HIV. This may strengthen the immune response against Gag that was started by the HIV-MAG vaccine.

5. The HIV-MAG vaccine and study adjuvant are given using electroporation in both arms.

So far, experimental DNA vaccines in people have produced mostly weak immune responses. One of the questions researchers have about DNA vaccines is how to make those immune responses stronger. One possibility is to try adjuvants.

Another possibility is to try to get more of the DNA from the vaccine into the body's cells so the DNA can do its work. One way of doing this is to use an electric pulse to briefly open tiny pores in the cells. The DNA can enter the cells through these pores. This method, called electroporation (EP), has been used for many years in the laboratory to get DNA or other substances into cells. Recently, a study showed that EP increased immune responses to another experimental DNA vaccine. Giving the study products with EP in both arms will also help get the products into more cells.

The EP device used in this study is called the TriGrid™ Delivery System (TDS) EP device. It was developed by Ichor Medical Systems, Inc. From now on we will call it "the TDS device". EP in people is an experimental procedure, and the TDS device is an experimental device. The TDS device is only used in people in research studies.

The TDS device gives an electrical pulse into the same muscle where the injection is given. This electrical pulse is delivered to the muscle through 4 needles at the same time as the study products are given. The TDS device has been tested in more than 80 people in other studies with other DNA vaccines. There were no serious side effects.

Joining the study

6. It is completely up to you whether or not to join the study.

Take your time in deciding. If it helps, talk to people you trust, such as your doctor, friends or family. If you decide not to join this study, or if you leave it after you have joined, your other care at this clinic and the benefits or rights you would normally have will not be affected.

If you join this study, you may not be allowed to join other HIV vaccine or HIV prevention studies now or in the future. Also during the study, you should not donate blood or tissue.

If you choose not to join this study, you could join another study if one is available and you are eligible. If you are interested, we can tell you about other HIV vaccine and prevention studies that we know of.

Site: Remove item 7 if you use a separate screening consent that covers these procedures.

7. If you decide to join the study, we will screen you to see if you are eligible.

Screening involves a physical exam, HIV test and health history. A physical exam may include, but is not limited to:

- Checking your weight, temperature and blood pressure

- Looking in your mouth and throat
- Listening to your heart and lungs
- Feeling your abdomen (stomach and liver)
- Pinching the skin of your upper arm and measuring it
- Doing a brief neurological exam to check your strength, senses, and brain function

We will also do blood and urine tests. These tests tell us about some key aspects of your health, such as how healthy your kidneys, liver, and immune system are. We will also test you for these sexually transmitted diseases: Hepatitis B, Hepatitis C, and Syphilis. We will ask you about medications you are taking. We will ask you about behaviors that might put you at risk for getting HIV. If you were born female, we will test you for pregnancy.

We will review the screening results with you, and offer you counseling and referral if you need medical care. The screening results may show you are not eligible to join the study, even if you want to. You cannot be in another research study where you receive a study product and be enrolled in this study.

8. If you were born female and could become pregnant, you must agree to use birth control to join this study.

Site: List approved birth control methods here if you do not want to hand out the separate Approved Birth Control Methods sheet.

You must agree to use effective birth control from three weeks before your first injection until your last clinic visit. We will talk to you about effective birth control methods. They are listed on a handout that we will give to you. *Site: Delete the preceding sentence if you list approved birth control methods in this consent form.* If you join the study, we will test you for pregnancy at some visits, including before each study injection.

Being in the study

If you are eligible to join the study after screening, and still want to participate, here is what will happen:

9. You will come to the clinic for scheduled visits about 17 times over one year if you are in Groups 1 and 3, or about 11 times over one year if you are in Groups 2 and 4.

Site: Insert number of visits and range of visit lengths. (There is site-specific variation in screening protocols and in the number of possible follow-up visits between protocol-mandated visits.)

Visits can last from [#] to [#] hours.

You may have to come for more visits if you have a laboratory or health issue.

We may contact you after the main study ends (eg, to tell you about the study results).

10. After you finish your clinic visits, we will contact you to ask about your health.

After the clinic visits are completed, we will contact you 3 months later and then once each year to check on your health. These health contacts will continue until 3 years after you received your first study injection.

We will talk more about this part of the study in Section 26 of this form.

11. We will give you [*Site:* Insert compensation] for each study visit you complete.

This amount is to cover the costs of [*Site:* Insert text]

Site: Insert any costs to participants (eg, birth control costs for female participants who could become pregnant).

Payments you receive for being in the study may be taxable. This happens if we pay you more than $600 between January 1 and December 31 of the same year. The clinic staff may need to ask you for your Social Security number for tax reasons.

12. We will give you either the study vaccines or a placebo.

Not everyone in this study will get the study vaccines. Some people will get a *placebo*, a substance that does not contain vaccine. We will compare the results from people who got the placebo with results from people who got the study vaccines. In this study, the placebo is sterile salt water.

You have about a 9 in 10 chance of receiving the study vaccines.

Site: Modify the randomization metaphor in the below paragraph as appropriate to your local culture.

Whether you get the study vaccines or the placebo is completely random, like rolling dice.

The reason we are testing the study vaccines is because we do not know whether they work or are safe. That means we do not know whether it is better to get the vaccines or to get the placebo. In either case, you need to take steps to protect yourself from HIV infection.

The clinic staff have no say in whether you get the study vaccines or the placebo. They will not know which one you are getting, and neither will you. Only the pharmacist at your site will have this information while the study is going on, and he or she will keep it a secret.

You will have to wait until all participants complete the main part of the study to find out whether you got the study vaccines or the placebo. This could be several years. But, if you have a serious medical problem and need to know what you got before the end of the study, we can tell you.

13. We will give you the study products on a schedule.

You will be in one of 4 groups. You will get injections 4 times during the study. At the first three injection visits you will get 2 injections in the muscle of your upper arm with EP, 1 in each arm. At the fourth injection visit you will also get 2 injections in the muscle of your upper arm, 1 in each arm, with a needle and syringe, but without EP.

Injection Schedule

	First Injection Visit (given with EP)	Month 1 (given with EP)	Month 3 (given with EP)	Month 6
Group 1	HIV-MAG or Placebo	HIV-MAG or Placebo	HIV-MAG or Placebo	VSV or Placebo
Group 2 (lower dose *IL-12*)	HIV-MAG+*IL-12* or Placebo	HIV-MAG+*IL-12* or Placebo	HIV-MAG +*IL-12* or Placebo	VSV or Placebo
Group 3 (higher dose *IL-12*)	HIV-MAG+*IL-12* or Placebo	HIV-MAG+*IL-12* or Placebo	HIV-MAG+*IL-12* or Placebo	VSV or Placebo
Group 4 (highest dose *IL-12*)	HIV-MAG+*IL-12* or Placebo	HIV-MAG+*IL-12* or Placebo	HIV-MAG+*IL-12* or Placebo	VSV or Placebo

For the first 3 injection visits you will receive the injections with EP. The TDS device is pressed against your upper arm firmly. You will feel some pressure against your arm. Once the device is set in place, the study staff will activate the device and you will hear two clicks. At this point the needles are inserted into your arm. For most people this does not hurt. It feels more like somebody flicked your arm. After the needles have been inserted, there will be a 3-4 second delay. The study products are being injected into your arm during this time.

After the injection, a very small amount of electricity is sent to the muscle. You will feel some pain or discomfort. The level of pain varies from person to person. The intensity of that feeling dies down within a minute or two. After that, your arm may be sore for a day or two. This procedure will be done once in each upper arm.

In a previous study of the TDS device in healthy volunteers, some people who received EP stated that they felt only a little discomfort while others said that it was very painful. However, 32 out of 32 volunteers returned for all of their scheduled injections.

At each injection visit, you will have to wait in the clinic for about a half hour after the procedure is done to see if there are any problems. Then for that night and for three more days, you will need to write down your symptoms and tell us how you are feeling. For the

fourth injection we will ask you to keep track of your symptoms and report them for 7 days. Contact the clinic staff if you have any issues or concerns after receiving an injection. If you have a problem, we will continue to check on you until it goes away.

14. In addition to giving you the study products, we will perform these procedures:

- Regular HIV testing, as well as counseling on your results and on how to avoid getting HIV;
- Physical exams;
- Collection of blood and urine samples;
- Pregnancy tests if you were born female;
- Questions about your health, including medications you may be taking;
- Personal questions about your HIV risk, including sexual behavior and drug use;
- Questions about any personal problems or benefits you may have from participating in the study;
- Questions to check your mental status; and
- We will review the results of these procedures and tests with you at your next visit, or sooner if necessary. We will also offer you counseling and referral for needed care.

Site: Paste table of procedures in this section or distribute it as a separate sheet if it is helpful to your study participants.

15. We will test your samples for reactions to the study products.

In this study, we will need to take blood from you with a needle on several occasions. The amount will depend on the lab tests we need to do. It will be some amount between 10 mL and 220 mL (2 teaspoons to 1 cup). Your body will make new blood to replace the blood we take out.

Site: You may want to add a sentence to the end of the previous paragraph contextualizing the blood volumes described (eg, "To compare, people who donate blood in the US can give a total of about 500 mL in an 8-week period."). Modify the example for cultural relevance and alter blood volumes as necessary.

We will use some of your samples to see if you have side effects from the study products. We will share these results with you.

If you have a fever and viral symptoms within a week after receiving the study injection at 6 months, we will collect blood, urine and saliva samples. If we need to collect saliva, we will ask you to avoid smoking, eating, or drinking anything but water, for an hour before saliva collection, if possible. We will ask you to spit into a container. We can give

you chewing wax to help you produce more saliva, if needed. We want to collect about 3 mL, which is less than a teaspoon.

We also will send your samples (without your name) to a lab to see how your immune system responds to the study products. This may include genetic tests. These tests are for research purposes only. The lab will not give the results to you or this clinic, and the results will not become part of your study record.

After this testing, we will continue to store your samples in case we need to repeat any tests for this study.

16. When we take samples from you for this study, we take extra samples in case we have to repeat tests. If the samples are no longer needed for this study, the HVTN wants to keep them for use in other studies.

These other studies are likely to be about HIV and the immune system. However, they could also help researchers understand other diseases.

Below we will ask if you agree to donate your extra samples combined with limited information. It is your decision. What you decide will not affect your study participation or any care you receive here. If you do not agree, the HVTN will make sure that your samples are destroyed when they are no longer needed for this study. If you agree, there is no limit on how long your samples will be stored. You can change your mind at any time and your samples will be destroyed.

The HVTN will not sell your samples or information.

What information might be shared with the samples? We will not share any information that would make it easy for anyone to identify you. However, some information may be personal, such as your race, ethnicity, sex, and health, including HIV status. Other information may be what product you received and how your body responded to the product.

What type of studies might be done with the extra samples and information? We cannot guess exactly how your extra samples and information will be used. To use them, any researcher must have his or her institutional review board (IRB) or ethics committee (EC) review the use of the samples. The IRB/EC protects the rights and well-being of research participants.

The studies may include genetic testing. Your genes are passed down from your mother and father. They affect how you look and how your body works. Limited genetic testing involves only some of your genes, not all of your genes (your genome). The researchers will not look at all of your genes, only the genes related to the immune system and diseases.

If you agree, your extra samples and information could also be used for genome wide studies. In these studies, researchers will look at all of your genes (your genome). The researchers compare the genomes of many people, looking for common patterns of genes that could help them understand diseases.

The researchers may put the information from the genome wide studies into a protected database so that other researchers can access it. Usually, no one would be able to look at your genome and link it to you as a person. However, if another database exists that also has information on your genome and your name, someone might be able to compare the databases and identify you. The risk of this is very small.

Will I see the results of the studies? The researchers will not report their results to you, this clinic, or your doctor. The results will not appear in your medical record. These other studies will not benefit you personally and they are not necessary for your medical care. Instead, the studies might help the public through new scientific discoveries.

17. We will do our best to protect your private information.

US sites: Check HIPAA authorization for conflicts with this section.

Your study records and samples will be kept in a secure location. We will label all of your samples and most of your records with a code number, not your name or other personal information. However, it is possible to identify you, if necessary. We will not share your name with the lab that does the tests on your samples, or with anyone else who does not need to know your name.

Clinic staff will have access to your study records. Your records may also be reviewed by groups who watch over this study to see that we are protecting your rights, keeping you safe, and following the study plan. These groups include:

- The US National Institutes of Health and its study monitors
- The US Food and Drug Administration
- [Insert name of local IBC],
- Profectus Biosciences, Inc. and people who work for them;
- The HVTN and people who work for them,
- The HVTN Safety Monitoring Board or the NIAID Data and Safety Monitoring Board, and
- The US Office for Human Research Protections

All reviewers will take steps to keep your records private.

We cannot guarantee absolute privacy. At this clinic, we have to report the following information:

Site: Include any public health or legal reporting requirements. Bulleted examples should include all appropriate cases (reportable communicable disease, risk of harm to self or others, etc.).

- [Item 1]

- [Item 2]
- [Item 3]

We have a Certificate of Confidentiality from the US government, to help protect your privacy. With the certificate, we do not have to release information about you to someone who is not connected to the study, such as the courts or police. Sometimes we can't use the certificate. Since the US government funds this research, we cannot withhold information from it. Also, you can still release information about yourself and your study participation to others.

Researchers who use your stored samples and limited information for other research will also do their best to protect your private information. The samples and limited information they receive will be labeled with a code number. They will not have your name or any personal information. Any reviewers of those studies will take steps to keep your records private.

The results of this study, and other studies that use the samples or information you agree to donate, may be published. No publication will use your name or identify you personally.

After the study is completed, information collected from the study may be made publically available to other researchers. If this is done, your name, personal information, and the code number used to identify you in our records will not be made available.

18. We may stop your injections or take you out of the study at any time.

This may happen if:

- you do not follow instructions,
- the researcher thinks that staying in the study might harm you,
- you get HIV,
- you enroll in a different research study where you receive another study product, or
- the study is stopped for any reason.

We may stop your injections or take you out of the study even if you want to continue and even if you were scheduled for additional injections. If we stop your injections, we may ask you to stay in the study to complete other study procedures.

19. If you become pregnant during the study, we will continue with some procedures but not injections.

We will do this for as long as it is safe for you and your developing baby. If you leave the study while you are still pregnant, we will contact you after your due date to ask some questions about your pregnancy and delivery.

20. If you get infected with HIV during the study, we will help you get care and support.

You will not be able to stay in this study. We will counsel you about your HIV infection and about telling your partner(s). We will tell you where you can get support and medical care, and about other studies you may want to join. We will not provide or pay for any of your HIV care directly. ***Site: Modify the preceding sentence as appropriate.***

Risks

21. There are risks to being in this study.

This section describes the risks and restrictions we know about. There may also be unknown risks, even serious ones. We will tell you if we learn anything new that may affect your willingness to stay in the study.

Risks of routine medical procedures:

In this study, we will do some routine medical procedures. These are taking blood and giving injections. These procedures can cause bruising, pain, fainting, soreness, redness, swelling, itching, muscle damage, and (rarely) infection where the needle was inserted. Taking blood can cause a low blood cell count (anemia), making you feel tired.

General risks of vaccines:

Rarely, a vaccine can cause an allergic reaction, including a rash, hives, or difficulty breathing. **Allergic reactions can be life-threatening.** You should tell us if you have ever had a bad reaction to any injection or vaccine.

All vaccines can cause fever, chills, rash, aches and pains, nausea, headache, dizziness, and feeling tired. Most people can still do their planned activities after getting a vaccine. Rarely, people experience side effects that limit their normal activities or make them go to the doctor.

Very rarely, a vaccine causes an autoimmune disease in a person, or makes an autoimmune disease worse. An autoimmune disease happens when your immune system attacks your own body, instead of attacking an infection. We do not know the risk of an autoimmune disease with this vaccine. We do know that a protein in VSV matches part of a protein in the body. It is possible that participants who receive the study vaccine may develop antibodies against this protein in the body. In theory, this could cause an autoimmune disease. However, from studies in animals and humans so far, there is no direct evidence that VSV infection causes autoimmune disease.

Risks of DNA vaccines and the study adjuvant:

We do not know all the risks of the HIV-MAG study vaccine because it has not been given to people before. Possible risks related to DNA vaccines include: muscle damage at the site of the injection, the production of antibodies which might react with normal body tissues and cause an autoimmune disease, and insertion of the vaccine DNA into the body's DNA. This could lead to cancer or unknown side effects. We think the risk of these things happening is low. More than 1000 people have been given DNA vaccines being tested against HIV and none of these things has happened so far.

Since 1995, thousands of people have received experimental DNA vaccines for diseases such as hepatitis, human papilloma virus (HPV, also known as genital warts), and HIV. In these people, the DNA vaccines also have not caused serious side effects.

We expect the risks of the DNA vaccine in this study to be similar to those of other DNA vaccines. However, there may be new side effects that we don't know about.

In earlier studies of the study adjuvant, there were no severe reactions or serious health problems related to the study adjuvant. We do not know if participants in this study will have similar side effects to those seen in earlier studies.

Risks of bupivacaine:

The HIV-MAG vaccine and *IL-12* adjuvant contain bupivacaine. Bupivacaine helps the study products get into the muscle. It is an anesthetic similar to the numbing medicine used by dentists. In much higher doses, bupivacaine can cause serious problems with the nervous system and heart. Other possible side effects include nausea, vomiting, or chills. However, in this study we will be using doses about 10 times lower than the dose that would have these effects.

Side effects of injections with electroporation:

An injection with the TriGrid™ Delivery System (TDS) EP device will cause brief muscle contractions during the procedure. In previous studies using electroporation, people felt initial pain that ranged from mild to severe. For most people, the pain eased quickly. Electroporation can also cause soreness, bruising, redness, swelling, itching, or hardness/stiffness in the upper arm where you got the injection. Minor damage to muscle cells is also possible. An ordinary injection with needle and syringe can also have these side effects. On rare occasions, the device may cause infection at the part of your body where you got the injection.

Having the procedure or thinking about it may cause some stress and anxiety. If you feel anxious, please tell us and we will try to help you.

We do not know if EP will change the risks for any of the study products. We do not know all the risks of EP with the TDS device because it has only been used with about 80 people before this study.

Risks of the VSV vaccine:

The VSV in the study vaccine has been weakened with genetic changes so that it does not cause illness in animals. However, it is still possible in theory, but very unlikely, that the study vaccine could cause symptoms that people get from VSV infection, including mouth sores or encephalitis. We do not know all the risks of the VSV vaccine because it has only been used in about 50 people before this study.

Personal problems/discrimination/testing HIV antibody positive:

About 10 to 20% of HVTN participants report personal problems or discrimination because of joining an HIV vaccine study. Family or friends may worry, get upset or angry, or assume that you are infected with HIV or at high risk and treat you unfairly as a

result. Rarely, a participant has lost a job because the study took too much time away from work, or their employer thought they had HIV.

The study vaccines may cause you to test positive on some types of HIV tests. This means that after you get the study vaccines, a routine HIV test done outside this clinic may say you have HIV, even if you don't. For this reason, you should plan to get HIV tests only at this clinic during the study. Our tests can tell the difference between true HIV infection and a positive result that is caused by the study vaccines.

If you receive a positive test result caused by the study vaccines at any time, we can provide you with free HIV testing for as long as you need it. If this happens, we do not know how long you will test positive due to the study vaccines. If you receive a positive HIV test result and we determine it is because you have HIV, we will refer you for follow-up care.

It is unlikely, but you could test negative at the end of the study and positive some time later, even though you don't have HIV. This could happen if different HIV tests come into use. We will give you a phone number to call for more information.

If someone believes you are infected with HIV even if you are not, you could face discrimination and other problems. For example, you could be denied medical or dental care, employment, insurance, a visa, or entry into the military. If you do have a positive HIV antibody test caused by the study, you will not be able to donate blood or organs. Your family and friends may treat you differently. We will give you a brochure that tells you more about testing HIV positive because of an HIV vaccine, and how you can avoid some of these problems.

Site: Modify the preceding paragraph if applicable.

Embarrassment/anxiety:

You may feel embarrassed when we ask about your HIV risks, such as having sex and using drugs. Also, waiting for your HIV test results or other health test results could make you feel anxious. You could feel worried if your test results show that you are infected with HIV. If you feel embarrassed or anxious, please tell us and we will try to help you.

Risks of disclosure of your personal information:

We will take several steps to protect your personal information. Although the risk is very low, it is possible that your personal information could be given to someone who should not have it. If that happened, you could face discrimination, stress, and embarrassment. We can tell you more about how we will protect your personal information if you would like it.

Unknown risks:

We do not know if the study vaccines will increase, decrease, or not change your risk of becoming infected with HIV if exposed. If you get infected with HIV, we do not know how the study vaccines might affect your HIV infection or how long it takes to develop AIDS.

We do not know if getting these study vaccines will affect how you respond to any future approved HIV vaccine. It could be that a future HIV vaccine may not work as well for you because you got the study vaccines. Currently, no HIV vaccine has been approved for use.

We do not know how the study vaccines will affect a pregnant participant or a developing baby.

Benefits

22. The study may not benefit you.

We do not know whether getting the study vaccines might benefit you in any way. However, being in the study might still help you in some ways. The counseling that you get as part of the study may help you avoid getting HIV. The lab tests and physical exams that you get while in this study might detect health problems you don't yet know about.

This study may help in the search for a vaccine to prevent HIV. However, if the study vaccines later become approved and sold, there are no plans to share any money with you. You will also not receive any money if you decide to donate your extra samples and limited information for other research, even if this research leads to a new product or discovery.

Your rights and responsibilities

23. If you join the study, you have rights and responsibilities.

As a participant, you have many rights that we respect. You also have responsibilities. We will give you the Participant's Bill of Rights and Responsibilities (PBORR). It describes your rights and responsibilities as a study participant.

Leaving the study

24. Tell us if you decide to leave the study.

You are free to leave the study at any time and for any reason. Your care at this clinic and your legal rights will not be affected, but it is important for you to let us know.

We will ask you to come back to the clinic one last time for a physical exam, and we may ask to take some blood and urine samples. We will also ask about any personal problems or benefits you have experienced from being in the study. We believe these steps are important to protecting your health, but it is up to you whether to complete them.

Injuries

25. If you get sick or injured during the study, contact us immediately.

Your health is important to us. We will help you get the medical care you need.

When someone gets sick or injured in an HVTN study, the HVTN decides whether the injury is probably related to the study products/procedures. If the HVTN decides it was more likely due to the study products or procedures than any other cause, then the HVTN and/or the companies who provide the study products for the trial will use their funds to pay for treatment. If the HVTN decides otherwise, then you and your health insurance *(Sites: insert locale- appropriate medical insurance language in the preceding paragraph)* would be responsible for treatment costs. You may disagree with the decision the HVTN makes about your injuries. At your request the HVTN will ask experts who are not connected with the HVTN to review its decision.

In this study, Profectus Biosciences, Inc. will pay the reasonable cost of medical expenses that arise from injuries caused by the study products, including the TriGrid Delivery System EP device.

For injuries caused by study procedures, other than electroporation, the HVTN has limited funds to cover the cost of medical treatment.

No matter what, you still have the right to use the court system to address payment for your injuries if you are not satisfied.

Some injuries are not physical. For example, someone might be harmed psychologically or emotionally by being in an HIV vaccine study. Or they might lose wages from injuries because they could not go to work. No funds have been set aside to pay for nonphysical injuries, even if they are related to participation in the study.

Annual health contacts

26. After your clinic visits end, we will contact you once a year until 3 years after your first injection.

We will contact you by phone or email *[Site: Modify mode of contact as appropriate]* once a year to ask questions about your health. If you prefer to answer these questions in person, an appointment with the study clinic can be arranged.

If we have any concerns about your health, we may need to have additional contact with you. You are also welcome to contact us at any time if you have concerns about your health related to your study participation.

If we ask you to come to the clinic, we will give you [Site: Insert compensation amount] for each visit. This amount is to cover the costs of [Site: Insert text].

If someone outside this study clinic told you that you are infected with HIV, we will ask you to come back to the clinic for another HIV test. We will draw about 15 mL (1 tablespoon) of blood. We may ask you to come back more than once for this testing.

Because we will want to contact you once a year, please tell us if your address, email, or phone number changes, if you are moving away, or if you do not want us to contact you anymore.

You can tell us at any time that you don’t want any more annual health contacts. If you do so, you will not lose any benefits or rights you would normally have.

All other information that is discussed earlier in this consent also applies to the annual health contacts.

Questions

27. If you have questions or problems at any time during your participation in this study, use the following important contacts.

If you have questions about this study, contact
[name and telephone number of the investigator or other study staff].

If you have any symptoms that you think may be related to this study, contact
[name and telephone number of the investigator or other study staff].

If you have questions about your rights as a research participant, or problems or concerns about how you are being treated in this study, contact
[name/title/phone of person on IRB or other appropriate organization].

If you want to leave this study, contact
[name and telephone number of the investigator or other study staff].

Your permissions and signature

28. In Section 16 of this form, we told you about possible other uses of your extra samples and limited information, outside this study. Please write your initials or make your mark in the box next to the option you choose.

☐ I agree to donate my extra samples combined with limited information for other studies related to HIV, the immune system, and other diseases. This may include limited genetic testing.

OR

☐ I agree to the option above and also to donate my extra samples combined with limited information for use in genome wide studies. I understand my genome and limited information may be put into a protected genome wide database.

OR

☐ I do not agree to donate my extra samples combined with limited information for use in other studies.

29. If you agree to join this study, you will need to sign below. Before you sign this consent form, make sure of the following:

- You have read this consent form, or someone has read it to you.
- You feel that you understand what the study is about and what will happen to you if you join. You understand what the possible risks and benefits are.

- You have had your questions answered and know that you can ask more.
- You agree to join this study.

You will not be giving up any of your rights by signing this consent form.

Participant's name (print)	Participant's signature or mark	Date	Time
Study staff conducting consent discussion (print)	Study staff signature	Date	Time

For participants who are unable to read or write, a witness should also complete the signature block below:

Witness's name (print)#	Witness's signature	Date	Time

Witness is impartial and was present for the consent process.

Appendix B Approved birth control methods (for sample informed consent form)

You should not become pregnant during the study because we do not know how the study vaccines could affect the developing baby.

If you were born female and are sexually active in a way that could lead you to get pregnant, you must agree to use effective birth control, starting at least 3 weeks before you get your first injection of study vaccines or placebo and continuing until your last clinic visit as specified in the informed consent.

Effective birth control means using any of the following methods every time you have sex:

- Birth control drugs that prevent pregnancy—given by pills, shots, patches, vaginal rings, or inserts under the skin;
- Male or female condoms, with or without a cream or gel that kills sperm;
- Diaphragm or cervical cap with a cream or gel that kills sperm;
- Intrauterine device (IUD); or
- Any other contraceptive method approved by the researchers.

You do not have to use birth control if:

- You are only having sex with a partner or partners who have had a vasectomy. (We will ask you some questions to confirm that the vasectomy was successful.);
- You have reached menopause, with no menstrual periods for one year;
- You have had a hysterectomy (your uterus removed);
- You have had your ovaries removed;
- You have a tubal ligation (your “tubes tied”) or confirmed successful placement of a product that blocks the fallopian tubes;
- You are having sex only with a female partner or partners;
- You only have oral sex; or,
- You are sexually abstinent (no sex at all).

Remember: If you are having sex, you need to use male or female condoms to protect yourself from HIV infection.

Appendix C HVTN VISP registry consent

Site: US sites use the following VISP consent language.

The HIV Vaccine Trials Network (HVTN) would like your permission to enter your name and link it to information about you in a computer registry (the "VISP registry"). By having your name and vaccine study information in the VISP registry, trained HVTN staff can quickly help you if you have problems with VISP.

About VISP

The body makes antibodies to prevent infection. Most vaccines cause the body to make antibodies as a way of preventing infection. Your body may make antibodies to HIV because you received an HIV vaccine. Some HIV test results could come back positive even if you are not infected with HIV. This is called a VISP (vaccine-induced seropositive) test result. We do not know who will have VISP test results or how long these test results may last.

People with VISP test results need specific HIV tests. These tests can tell whether a test result is VISP or a real HIV infection. These people may need help explaining their VISP situation if someone outside the study wants to test them for HIV. VISP test results may cause problems in several areas like insurance, job applications, the military, prison, visa applications, emigration/immigration, and blood and tissue donation.

We are asking you for your permission to enter your name in the registry now in case you have VISP test results later. The registry will not be used for any other purpose.

What are the benefits of the registry?

Your study site will help you with problems related to VISP test results. If you are unable to go to your original study site, an HVTN counselor will help you with these problems. The HVTN counselor will need to verify your study participation and if you received an HIV vaccine. The registry gives the HVTN counselor quick access to this information.

If you choose not to have your name entered in the registry, HVTN counselors still will do their best to help you. However, it will take longer to get that information. If your study site is no longer doing HIV vaccine studies, your records may be stored securely off site. It is possible your records may not be found.

What information does the registry contain and how is it protected?

The registry contains the following information:

- Your participant ID (the code used for you instead of your name at your study site)
- The study network and study you were in
- The site where you began the study

- The date you began the study
- Your date of birth or age
- If you received an HIV vaccine that may cause you to test VISP

We are asking for your permission to enter your name into the registry and link it to the information above.

The registry will NOT contain:

- Your HIV test results
- Your phone number or any other way to contact you

Any other personal information that you discuss with the HVTN counselor will be kept separate from the registry. We will keep your name in the registry until you tell us you want it removed.

All people who work with your registry information sign agreements to keep the information confidential.

The registry is a secured computer database. It can only be accessed with a password.

What are the risks?

The only risk to having your name entered and linked to the other pieces of information in the registry is that someone who is not authorized might see your information. The risk of this happening is low because of the security protections in place. However, we cannot guarantee this will never happen.

What if I have more questions about the registry?

Please talk to your study site or call the phone number they provided if you have any questions about the registry now or in the future.

If I agree now, can I change my mind later?

Yes. You can contact your study site or call the number you have been given anytime to tell us that you would like your name to be deleted from the registry. We will send you confirmation that we deleted it. Your decision will not affect your participation in the main HIV vaccine study.

By signing this form, you do not give up any legal rights.

Please write your initials or make your mark in the box next to the option you choose.

☐ I **AGREE** to allow my name to be entered and linked to the information in the HVTN VISP registry.

☐ I **DO NOT AGREE** to allow my name to be entered and linked to the information in the HVTN VISP registry.

Please sign or make your mark below

Participant's name (print)	Participant's signature or mark	Date	Time
Study staff conducting consent discussion (print)	Study staff signature	Date	Time

For participants who are unable to read or write, a witness should complete the signature block below.

Witness's name (print)#	Witness's signature	Date	Time

Witness is impartial and was present for the consent process.

Appendix D Table of procedures (for sample informed consent form)

			Visits (number, months, and time after each injection)															
	Screening	**1st injection**	1 day after 1st injection	3 days after 1st injection	2 weeks after 1st injection	**2nd injection** (Month 1)	2 weeks after 2nd injection	**3rd injection** (Month 3)	1 week after 3rd injection	2 weeks after 3rd injection	**4th injection** (Month 6)	1 day after 4th injection	3 days after 4th injection	1 week after 4th injection	2 weeks after 4th injection	Month 9	Month 12	Month 15*
**Procedure**	Visit 1	Visit 2	Visit 3	Visit 4	Visit 5	Visit 6	Visit 7	Visit 8	Visit 9	Visit 10	Visit 11	Visit 12	Visit 13	Visit 14	Visit 15	Visit 16	Visit 17	Visit 18
Injection		√				√		√			√							
Medical history	√																	
Complete physical	√																√	
Brief physical‡		√	√	√	√	√	√	√	√	√	√	√	√	√	√	√		
Mini Mental State Exam	√										√	√	√	√	√			
Urine test	√				√										√			
Blood drawn	√	√	√	√	√		√		√	√	√	√	√	√	√	√	√	
Pregnancy test (participants born female)	√	√				√		√			√					√		
HIV testing / counseling	√	√								√	√				√	√	√	
Interview / questionnaire	√	√	√	√	√	√	√	√	√	√	√	√	√	√	√	√	√	
Risk reduction counseling	√	√			√	√	√	√		√	√				√	√	√	
Phone contact*																		√

*Clinic visit is not required. However, if someone outside the study told you that you are infected with HIV we will ask you to come back to the clinic for another HIV test.
‡ For participants who report viral symptoms within a week after receiving the study injection at 6 months, we will collect blood, urine, and saliva samples.

Grayed out columns = visits not required for Groups 2 & 4.

Not shown in this table is a time after all study participants have completed their last scheduled visit when you can find out what products you received.

Appendix E Laboratory procedures for Groups 1 and 3

				Tube volume (mL)																		
			Visit:	**1**	**2**	**3**	**4**	**5**	**6**	**7**	**8**	**9**	**10**	**11**	**12**	**13**	**14**	**15**	**16**	**17**	**18**[11]	
			Day:	Screening	D0	D1	D3	D14	D28	D42	D84	D91	D98	D168	D169	D171	D175	D182	D273	D364	D455	
			Month:	visit[4]	M0			M0.5	M1	M1.5	M3	M3.25	M3.5	M6				M6.5	M9	M12	M15	
Procedure	Ship to[1]	Assay location[2,3]	Tube[5]		**VAC1**				**VAC2**		**VAC3**			**VAC4**								**Total**
BLOOD COLLECTION																						
**Screening or diagnostic assays**																						
Screening HIV test	Local lab	Local lab	SST	5	—	—	—	—	—	—	—	—	—	—	—	—	—	—	—	—	—	**5**
HBsAg/anti-HCV/Syphilis	Local lab	Local lab	SST	5	—	—	—	—	—	—	—	—	—	—	—	—	—	—	—	—	—	**5**
HIV in-study diagnostic[10]	UW-VSL	UW-VSL	EDTA	—	—	—	—	—	—	—	—	—	10	—	—	—	—	10	10	20	—	**50**
**Safety labs**																						
CBC/ Diff/ platelets	Local lab	Local lab	EDTA	5	5	5	5	5	—	5	—	5	5	5	5	5	5	5	5	—	—	**70**
Chemistry panel[6]	Local lab	Local lab	SST	5	—	—	—	5	—	5	—	—	5	—	—	—	—	5	5	—	—	**30**
T cell subsets	Local lab	Local lab	EDTA	—	5	—	—	—	—	5	—	—	5	—	—	—	—	5	—	—	—	**20**
**Immunogenicity assays**[7]																						
Genotyping[8]	CSR	FHCRC	ACD	—	20	—	—	—	—	—	—	—	—	—	—	—	—	—	—	—	—	**20**
Cellular assays																						
ICS	CSR	FHCRC	ACD	—	60	—	—	—	—	60	—	—	***60***	—	—	—	—	***60***	—	60	—	**300**
Humoral assays																						
HIV-1 multiplex Ab assay	CSR	Duke	EDTA	—	***10***	—	—	—	—	—	—	—	***10***	—	—	—	—	***10***	—	10	—	**40**
IL-12 binding ab ELISA	CSR	Profectus	SST	—	5	—	—	—	—	—	—	—	5	—	—	—	—	5	—	5	—	**20**
IL-12 neutralizing ab assay	CSR	Profectus	SST	—	5	—	—	—	—	—	—	—	5	—	—	—	—	5	—	5	—	**20**
Innate Immunity Assays																						
Gene Expression	CSR	FHCRC	ACD	—	10	—	—	—	—	—	—	10	—	10	10	10	10	10	—	10	—	**80**
Gene Expression	CSR	FHCRC	Tempus	—	3	3	3	3	—	—	—	3	—	3	3	3	3	3	—	3	—	**33**
TruCOUNT	FHCRC	FHCRC	—	—	y	y	y	y	—	—	—	y	—	y	y	y	y	y	—	—	—	**0**
Serum cytokines	CSR	FHCRC	SST	—	5	5	5	5	—	—	—	5	—	5	5	5	5	5	—	5	—	**55**
**Specimen storage**																						
PBMC	CSR		ACD	—	80	—	—	—	—	80	—	60	80	30	30	30	30	80	—	80	—	**580**
Plasma	CSR		ACD	—	z	—	—	—	—	z	—	z	z	z	z	z	z	z	—	z	—	**0**
Plasma	CSR		EDTA	—	10	—	—	—	—	10	—	—	10	—	—	—	—	10	—	10	—	**50**
Serum	CSR		SST	—	5	—	—	—	—	5	—	5	5	—	—	—	—	5	—	5	—	**30**
**Total**				**20**	**223**	**13**	**13**	**18**	**0**	**170**	**0**	**88**	**200**	**53**	**53**	**53**	**53**	**218**	**20**	**213**	**0**	**1408**
**56-Day total**				**20**	**243**	**256**	**269**	**287**	**287**	**457**	**170**	**258**	**458**	**53**	**106**	**159**	**212**	**430**	**20**	**213**	**0**	
URINE COLLECTION																						
Urinalysis	Local lab	Local lab		X	—	—	—	X	—	—	—	—	—	—	—	—	—	X	—	—	—	
Pregnancy test[9]	Local lab	Local lab		X	X	—	—	—	X	—	X	—	—	X	—	—	—	—	X	—	—	

y = 0.75mL of whole blood will be taken from an ACD tube already drawn during PBMC processing for Trucount; no separate blood draw is needed

z = up to 5mL of plasma will be harvested during ACD blood PBMC processing; no separate blood draw is needed

[1] CSR = central specimen repository

[2] HVTN Laboratory Program includes laboratories at UW-VSL, Duke and FHCRC. UW-VSL = University of Washington Virology Specialty Laboratory (Seattle, Washington, USA); Duke = Duke University Medical Center (Durham, North Carolina, USA); FHCRC = Fred Hutchinson Cancer Research Center (Seattle, Washington, USA)

[3] Non-HVTN Laboratories: Profectus Biosciences, Inc (Tarrytown, New York, USA)

[4] Screening may occur over the course of several contacts/visits up to and including day 0 prior to vaccination.

[5] Local labs may assign appropriate alternative tube types for locally performed tests.

[6] Chemistry panels are defined in Section 9.2 (pre-enrollment) and Section 9.4 (postenrollment).

[7] Immunogenicity assays will be performed at Month 0 (for binding Ab assay), Month 3.5 and Month 6.5. Based on the number of responders observed at these timepoints, lab assays may be performed on all participants for humoral and cellular responses at other timepoints.

[8] Genotyping may be performed on enrolled participants using cryopreserved PBMC collected at baseline, initially in participants who demonstrate vaccine-induced T-cell responses at postvaccination timepoints.

[9] Pregnancy test may be performed on blood specimens.

[10] At an early termination visit for a withdrawn or terminated participant (see Section 9.14), blood should be drawn for HIV diagnostic testing, as shown for visit 12 above.

[11] For information concerning the Month 15 health contact see Section 9.5. Clinic visits are not required, except that any participant reporting a diagnosis of HIV infection will be asked to come to the clinic so that HIV status can be confirmed.

[12] For participants who report symptoms of a systemic viral syndrome after the 4th vaccination, 3mL of EDTA blood, saliva, and urine will be collected at up to 7 days postvaccination for the rVSV viral infectivity assay.

Appendix F Laboratory procedures for Groups 2 and 4

				Tube volume (mL)																		
			Visit:	**1**	**2**	**3**	**4**	**5**	**6**	**7**	**8**	**9**	**10**	**11**	**12**	**13**	**14**	**15**	**16**	**17**	**18**[11]	
			Day:	Screening	D0	D1	D3	D14	D28	D42	D84	D91	D98	D168	D169	D171	D175	D182	D273	D364	D455	
			Month:	visit[4]	M0			M0.5	M1	M1.5	M3	M3.25	M3.5	M6				M6.5	M9	M12	M15	
Procedure	Ship to[1]	Assay location[2,3]	Tube[5]		**VAC1**				**VAC2**		**VAC3**			**VAC4**								**Total**
BLOOD COLLECTION																						
**Screening or diagnostic assays**																						
Screening HIV test	Local lab	Local lab	SST	5	—	—	—	—	—	—	—	—	—	—	—	—	—	—	—	—	—	**5**
HBsAg/anti-HCV/Syphilis	Local lab	Local lab	SST	5	—	—	—	—	—	—	—	—	—	—	—	—	—	—	—	—	—	**5**
HIV in-study diagnostic[10]	UW-VSL	UW-VSL	EDTA	—	—	—	—	—	—	—	—	—	10	—	—	—	—	10	10	20	—	**50**
**Safety labs**																						
CBC/ Diff/ platelets	Local lab	Local lab	EDTA	5	—	—	—	5	—	5	—	—	5	—	—	—	—	5	5	—	—	**30**
Chemistry panel[6]	Local lab	Local lab	SST	5	—	—	—	5	—	5	—	—	5	—	—	—	—	5	5	—	—	**30**
T cell subsets	Local lab	Local lab	EDTA	—	5	—	—	—	—	5	—	—	5	—	—	—	—	5	—	—	—	**20**
**Immunogenicity assays**[7]																						
Genotyping[8]	CSR	FHCRC	ACD	—	20	—	—	—	—	—	—	—	—	—	—	—	—	—	—	—	—	**20**
Cellular assays																						
ICS	CSR	FHCRC	ACD	—	60	—	—	—	—	60	—	—	*60*	—	—	—	—	*60*	—	60	—	**300**
Humoral assays																						
HIV-1 multiplex Ab assay	CSR	Duke	EDTA	—	*10*	—	—	—	—	—	—	—	*10*	—	—	—	—	*10*	—	10	—	**40**
IL-12 binding ab ELISA	CSR	Profectus	SST	—	*5*	—	—	—	—	—	—	—	5	—	—	—	—	*5*	—	5	—	**20**
IL-12 neutralizing ab assay	CSR	Profectus	SST	—	5	—	—	—	—	—	—	—	5	—	—	—	—	5	—	5	—	**20**
**Specimen storage**																						
PBMC	CSR	—	ACD	—	80	—	—	—	—	80	—	—	80	—	—	—	—	80	—	80	—	**400**
Plasma	CSR	—	ACD	—	z	—	—	—	—	z	—	—	z	—	—	—	—	z	—	z	—	**0**
Plasma	CSR	—	EDTA	—	10	—	—	—	—	10	—	—	10	—	—	—	—	10	—	10	—	**50**
Serum	CSR	—	SST	—	5	—	—	—	—	5	—	—	5	—	—	—	—	5	—	5	—	**25**
**Total**				**20**	**200**	**0**	**0**	**10**	**0**	**170**	**0**	**0**	**200**	**0**	**0**	**0**	**0**	**200**	**20**	**195**	**0**	**1015**
**56-Day total**				**20**	**220**	**220**	**220**	**230**	**230**	**400**	**170**	**170**	**370**	**0**	**0**	**0**	**0**	**200**	**20**	**195**	**0**	
URINE COLLECTION																						
Urinalysis	Local lab	Local lab		X	—	—	—	X	—	—	—	—	—	—	—	—	—	X	—	—	—	
Pregnancy test[9]	Local lab	Local lab		X	X	—	—	—	X	—	X	—	—	X	—	—	—	—	X	—	—	

Grayed out columns = visits not required for Groups 2and 4.

z = up to 5mL of plasma will be harvested during ACD blood PBMC processing; no separate blood draw is needed

[1] CSR = central specimen repository

[2] HVTN Laboratory Program includes laboratories at UW-VSL, Duke and FHCRC. UW-VSL = University of Washington Virology Specialty Laboratory (Seattle, Washington, USA); Duke = Duke University Medical Center (Durham, North Carolina, USA); FHCRC = Fred Hutchinson Cancer Research Center (Seattle, Washington, USA)

[3] Non-HVTN Laboratories: Profectus Biosciences, Inc (Tarrytown, New York, USA)

[4] Screening may occur over the course of several contacts/visits up to and including day 0 prior to vaccination.

[5] Local labs may assign appropriate alternative tube types for locally performed tests.

[6] Chemistry panels are defined in section 9.2 (pre-enrollment) and section 9.4(post-enrollment).

[7] Immunogenicity assays will be performed at Month 0 (for binding Ab assay), Month 3.5 and Month 6.5. Based on the number of responders observed at these timepoints, lab assays may be performed on all participants for humoral and cellular responses at other timepoints.

[8] Genotyping may be performed on enrolled participants using cryopreserved PBMC collected at baseline, initially in participants who demonstrate vaccine-induced T-cell responses at postvaccination timepoints.

[9] Pregnancy test may be performed on blood specimens.

[10] At an early termination visit for a withdrawn or terminated participant (see Section 9.14), blood should be drawn for HIV diagnostic testing, as shown for visit 12 above.

[11] For information concerning the Month 15 health contact see Section 9.5. Clinic visits are not required, except that any participant reporting a diagnosis of HIV infection will be asked to come to the clinic so that HIV status can be confirmed.

[12] For participants who report symptoms of a systemic viral syndrome after the 4th vaccination, 3mL of EDTA blood, saliva, and urine will be collected at up to 7 days postvaccination for the rVSV viral infectivity assay.

Appendix G Procedures at HVTN CRS for Groups 1 and 3

Visit:	1[a]	2	3	4	5	6	7	8	9	10	11	12	13	14	15	16	17	18	Post
Day:		D0	D1	D3	D14	D28	D42	D84	D91	D98	D168	D169	D171	D175	D182	D273	D364	D455	
Month:		M0			M0.5	M1	M1.5	M3	M3.25	M3.5	M6			M6.25	M6.5	M9	M12	M15[h]	
**Procedure**	Screening visit	**VAC1**				**VAC2**		**VAC3**			**VAC4**								
**Study procedures[b]**																			
Signed screening consent (if used)	X	—	—	—	—	—	—	—	—	—	—	—	—	—	—	—	—	—	—
Assessment of understanding	X	—	—	—	—	—	—	—	—	—	—	—	—	—	—	—	—	—	—
Signed protocol consents	X	—	—	—	—	—	—	—	—	—	—	—	—	—	—	—	—	—	—
Medical history	X	—	—	—	—	—	—	—	—	—	—	—	—	—	—	—	—	—	—
Complete physical exam	X	—	—	—	—	—	—	—	—	—	—	—	—	—	—	—	—	—	—
Abbreviated physical exam[i]	—	X	X	X	X	X	X	X	X	X	X	X	X	X	X	X	X	—	—
Mini Mental State Examination	X	—	—	—	—	—	—	—	—	—	X	X	X	X	X	—	—	—	—
Risk reduction counseling	X	X	—	—	X	X	X	X	—	X	X	—	—	—	X	X	X	—	—
Pregnancy prevention assessment[c]	X	X	—	—	X	X	X	X	—	X	X	—	—	—	X	X	X	—	—
Behavioral risk assessment	X	—	—	—	—	—	—	—	—	—	—	—	—	—	—	—	—	—	—
Confirm eligibility, obtain demographics, randomize	X	—	—	—	—	—	—	—	—	—	—	—	—	—	—	—	—	—	—
Social impact assessment	—	X	—	—	X	X	X	X	—	X	X	—	—	—	X	X	X	—	—
Social impact assessment questionnaire	—	—	—	—	—	—	—	X	—	—	X	—	—	—	—	—	X	—	—
Outside testing and belief questionnaire	—	—	—	—	—	—	—	—	—	—	X	—	—	—	—	—	X	—	—
Concomitant medications	X	X	X	X	X	X	X	X	X	X	X	X	X	X	X	X	X	—	—
Intercurrent illness/adverse experience[i]	—	X	X	X	X	X	X	X	X	X	X	X	X	X	X	X	X	—	—
HIV infection assessment[d]	X	—	—	—	—	—	—	—	—	X	—	—	—	—	X	X	X	—	—
Confirm HIV test results provided to participant	—	X	—	—	—	—	—	—	—	—	X	—	—	—	—	X	X	—	—
Visual Analog Scale (pain assessment)	—	X	—	—	—	X	—	X		—	X	—	—	—	—	—	—	—	—
Acceptability questionnaires	—	—	—	—	X	—	X	—	—	X	—	—	—	—	—	—	—	—	—
Phone contact	—	—	—	—	—	—	—	—	—	—	—	—	—	—	—	—	—	X	—
**Local lab assessment**																			
Urine dipstick	X	—	—	—	X	—	—	—	—	—	—	—	—	—	X	—	—	—	—
Pregnancy (urine or serum HCG)[e]	X	X	—	—	—	X	—	X	—	—	X	—	—	—	—	X	—	—	—
CBC, differential, platelets	X	X	X	X	X	—	X	—	X	X	X	X	X	X	X	X	—	—	—
Chemistry panel	X	—	—	—	X	—	X	—	—	X	—	—	—	—	X	X	—	—	—
T-cell subsets	—	X	—	—	—	—	X	—	—	X	—	—	—	—	X	—	—	—	—
Syphilis, Hepatitis B, Hepatitis C	X	—	—	—	—	—	—	—	—	—	—	—	—	—	—	—	—	—	—
**Vaccination procedures**																			
Vaccination[f]	—	X	—	—	—	X	—	X	—	—	X	—	—	—	—	—	—	—	—
Reactogenicity assessments[g]	—	X	—	—	—	X	—	X	—	—	X	—	—	—	—	—	—	—	—
**Poststudy**																			
Unblind participant	—	—	—	—	—	—	—	—	—	—	—	—	—	—	—	—	—	—	X

[a] Screening may occur over the course of several contacts/visits up to and including day 0 prior to vaccination.
[b] For specimen collection requirements, see Appendix E.
[c] Pregnancy prevention compliance occurs only with participants who were born female and are capable of becoming pregnant.
[d] Includes pre-test counseling. A subsequent follow-up contact is conducted to provide post-test counseling and to report results to participant.
[e] For a participant who was born female, pregnancy test must be performed on the day of vaccination prior to vaccination. Pregnancy test to determine eligibility may be performed at screening or on day 0 prior to first vaccination. Serum pregnancy tests may be used to confirm the results of, or substitute for, a urine pregnancy test.
[f] Blood draws required at vaccination visits must be performed prior to administration of study product; however, it is not necessary to have results prior to administration. Lab tests may be drawn within the 3 days prior to vaccination.
[g] Reactogenicity assessments performed daily for 3 - 7 days postvaccination (see Section 9.10).
[h] For information concerning the Month 15 health contact, see Section 9.5. Clinic visits are not required expect that any participant reporting a diagnosis of HIV infection will be asked to come to the clinic so that HIV status can be confirmed.
[i] For participants who report symptoms of a systemic viral syndrome after the 4th vaccination, 3mL of EDTA blood, saliva, and urine will be collected at up to 7 days postvaccination for the rVSV viral infectivity assay.

Appendix H Procedures at HVTN CRS for Groups 2 and 4

Visit:	1[a]	2	3	4	5	6	7	8	9	10	11	12	13	14	15	16	17	18	Post
Day:		D0	D1	D3	D14	D28	D42	D84	D91	D98	D168	D169	D171	D175	D182	D273	D364	D455	
Month:		M0			M0.5	M1	M1.5	M3	M3.25	M3.5	M6			M6.25	M6.5	M9	M12	M15[h]	
**Procedure**	Screening visit	VAC1				VAC2		VAC3			VAC4								
**Study procedures[b]**																			
Signed screening consent (if used)	X	—	—	—	—	—	—	—	—	—	—	—	—	—	—	—	—	—	—
Assessment of understanding	X	—	—	—	—	—	—	—	—	—	—	—	—	—	—	—	—	—	—
Signed protocol consents	X	—	—	—	—	—	—	—	—	—	—	—	—	—	—	—	—	—	—
Medical history	X	—	—	—	—	—	—	—	—	—	—	—	—	—	—	—	—	—	—
Complete physical exam	X	—	—	—	—	—	—	—	—	—	—	—	—	—	—	—	—	—	—
Abbreviated physical exam[i]	—	X	—	—	X	X	X	X	—	X	X	—	—	—	X	X	X	—	—
Mini Mental State Examination	X	—	—	—	—	—	—	—	—	—	X	—	—	—	X	—	—	—	—
Risk reduction counseling	X	X	—	—	X	X	X	X	—	X	X	—	—	—	X	X	X	—	—
Pregnancy prevention assessment[c]	X	X	—	—	X	X	X	X	—	X	X	—	—	—	X	X	X	—	—
Behavioral risk assessment	X	—	—	—	—	—	—	—	—	—	—	—	—	—	—	—	—	—	—
Confirm eligibility, obtain demographics, randomize	X	—	—	—	—	—	—	—	—	—	—	—	—	—	—	—	—	—	—
Social impact assessment	—	X	—	—	X	X	X	X	—	X	X	—	—	—	X	X	X	—	—
Social impact assessment questionnaire	—	—	—	—	—	—	—	X	—	—	X	—	—	—	—	—	X	—	—
Outside testing and belief questionnaire	—	—	—	—	—	—	—	—	—	—	X	—	—	—	—	—	X	—	—
Concomitant medications	X	X	—	—	X	X	X	X	—	X	X	—	—	—	X	X	X	—	—
Intercurrent illness/adverse experience[i]	—	X	—	—	X	X	X	X	—	X	X	—	—	—	X	X	X	—	—
HIV infection assessment[d]	X	—	—	—	—	—	—	—	—	X	—	—	—	—	X	X	X	—	—
Confirm HIV test results provided to participant	—	X	—	—	—	—	—	—	—	—	X	—	—	—	—	X	X	—	—
Visual Analog Scale (pain assessment)	—	X	—	—	—	X	—	X		—	X	—	—	—	—	—	—	—	—
Acceptability questionnaires	—	—	—	—	X	—	X	—	—	X	—	—	—	—	—	—	—	—	—
Phone contact	—	—	—	—	—	—	—	—	—	—	—	—	—	—	—	—	—	X	—
**Local lab assessment**																			
Urine dipstick	X	—	—	—	X	—	—	—	—	—	—	—	—	—	X	—	—	—	—
Pregnancy (urine or serum HCG)[e]	X	X	—	—	—	X	—	X	—	—	X	—	—	—	—	X	—	—	—
CBC, differential, platelets; Chemistry panel	X	—	—	—	X	—	X	—	—	X	—	—	—	—	X	X	—	—	—
T-cell subsets	—	X	—	—	—	—	X	—	—	X	—	—	—	—	X	—	—	—	—
Syphilis, Hepatitis B, Hepatitis C	X	—	—	—	—	—	—	—	—	—	—	—	—	—	—	—	—	—	—
**Vaccination procedures**																			
Vaccination[f]	—	X	—	—	—	X	—	X	—	—	X	—	—	—	—	—	—	—	—
Reactogenicity assessments[g]	—	X	—	—	—	X	—	X	—	—	X	—	—	—	—	—	—	—	—
**Poststudy**																			
Unblind participant	—	—	—	—	—	—	—	—	—	—	—	—	—	—	—	—	—	—	X

Grayed out columns = study visits not required for Groups 2 and 4.

[a] Screening may occur over the course of several contacts/visits up to and including day 0 prior to vaccination.
[b] For specimen collection requirements, see Appendix F.
[c] Pregnancy prevention compliance occurs only with participants who were born female and are capable of becoming pregnant.
[d] Includes pre-test counseling. A subsequent follow-up contact is conducted to provide post-test counseling and to report results to participant.
[e] For a participant who was born female, pregnancy test must be performed on the day of vaccination prior to vaccination. Pregnancy test to determine eligibility may be performed at screening or on day 0 prior to first vaccination. Serum pregnancy tests may be used to confirm the results of, or substitute for, a urine pregnancy test.
[f] Blood draws required at vaccination visits must be performed prior to administration of study product; however, it is not necessary to have results prior to administration. Lab tests may be drawn within the 3 days prior to vaccination.
[g] Reactogenicity assessments performed daily for 3 - 7 days postvaccination (see Section 9.10).
[h] For information concerning the Month 15 health contact, see Section 9.5. Clinic visits are not required expect that any participant reporting a diagnosis of HIV infection will be asked to come to the clinic so that HIV status can be confirmed.
[i] For participants who report symptoms of a systemic viral syndrome after the 4th vaccination, 3mL of EDTA blood, saliva, and urine will be collected at up to 7 days postvaccination for the rVSV viral infectivity assay.

Appendix I Procedures at CRS for annual health contacts

Contact[a] Day	728	728
Month	24	36
**Procedures**		
Vital status and health events[b]	X	X

[a] Clinic visits are not required, except that any participant reporting a diagnosis of HIV infection will be asked to come to the clinic so that HIV status can be confirmed.

[b] See section 9.5.

Appendix J Adverse Events of Special Interest (AESI)

AEs of special interest (AESI) for this protocol include but are not limited to autoimmune disorders; representative examples of AESI are listed below. Updates to AESI will be provided as an appendix to the *HVTN 087 Study Specific Procedures*.

Neuroinflammatory disorders

Optic neuritis
Multiple sclerosis
Demyelinating disease
Transverse myelitis
Guillain-Barré syndrome
Myasthenia gravis
Encephalitis
Neuritis
Bell's palsy

Musculoskeletal disorders

Systemic lupus erythematosus
Cutaneous lupus
Sjogren's syndrome
Scleroderma, dermatomyositis
Polymyositis
Rheumatoid arthritis
Juvenile rheumatoid arthritis
Polymyalgia rheumatica
Reactive arthritis
Psoriatic arthropathy
Ankylosing spondylitis
Spondyloarthropathy

Gastrointestinal disorders

Crohn's disease
Ulcerative colitis
Celiac disease

Metabolic diseases

Autoimmune thyroiditis
Grave's or Basedow's disease
Hashimoto thyroiditis
Insulin-dependent diabetes mellitus [IDDM]
Addison's disease
Insulin-dependent diabetes mellitus [IDDM]

Skin disorders

Psoriasis
Vitiligo
Raynaud's phenomenon
Erythema nodosum
Autoimmune bullous skin diseases

Others

Autoimmune hemolytic anemia
Idiopathic thrombocytopenic purpura
Antiphospholipid syndrome
Temporal arteritis
Behcet's syndrome
Pernicious anemia
Autoimmune hepatitis
Primary biliary cirrhosis
Primary sclerosing cholangitis
Autoimmune glomerulonephritis
Autoimmune uveitis
Autoimmune cardiomyopathy
Sarcoidosis
Stevens-Johnson syndrome
Vasculitides
