# Supplementary material for: Safety and tolerability of HIV-1 multiantigen pDNA vaccine given with IL-12 plasmid DNA via electroporation, boosted with a recombinant vesicular stomatitis virus HIV Gag vaccine in healthy volunteers in a randomized, controlled clinical trial
Source: PLoS One. 2018 Sep 20;13(9):e0202753. doi: 10.1371/journal.pone.0202753 (PMC6147413; doi:10.1371/journal.pone.0202753)
Supplement: S1 Methods — (DOCX) [file pone.0202753.s003.docx]

**S1 Methods.** IL-12 neutralizing antibody assessment

IL-12 neutralization assays were performed using the NK-92MI cell line (ATCC), which exhibits a linear secretion of IFN-γ in response to exposure of increasing amounts of IL-12 cytokine. Neutralizing serum to IL-12 will block the IFN-γ secretion in NK-92MI cells, so the IFN-γ signal is reciprocal to the neutralizing activity of the serum. The assay was run on a 96-well plate format. Briefly, test serum (along with reference standard and positive/negative controls) was serially diluted and pre-incubated for 1 hour at RT with IL-12 (12 IU/ml per well). Following the incubation, the serum/IL-12 mixture was added to the NK-92MI cells to a final concentration of 3x10^4^ NK cells/well and 3 IL-12 IU/ml. NK cells were incubated with serum for 18-24 hours at 37°C in a 5%CO_2_ incubator. IFN-γ was detected by performing an IFN-γ ELISA, using anti-IFN-γ capture antibodies (BD Biosciences). The neutralizing serum response was calculated relative to the reference standard dose curve by performing a four-parameter logistic (4PL) nonlinear transformation. By definition, a neutralization titer is the amount of antibody that neutralizes 1 IL-12 IU.

Samples from baseline and from 2 weeks after the third DNA vaccination were measured for IL-12 neutralization antibody responses. The IL-12 neutralization titers at baseline were used to calibrate results for IL-12 neutralization antibodies after three DNA vaccinations. The post-vaccination response to IL-12 was considered to be positive if the neutralization titer was greater than the 97.5th percentile of baseline neutralization titers among all participants, and greater than two times its corresponding baseline neutralization titer. According to these criteria, there was no vaccination-induced IL-12 neutralization antibody response.
