# Supplementary material for: Safety and tolerability of HIV-1 multiantigen pDNA vaccine given with IL-12 plasmid DNA via electroporation, boosted with a recombinant vesicular stomatitis virus HIV Gag vaccine in healthy volunteers in a randomized, controlled clinical trial
Source: PLoS One. 2018 Sep 20;13(9):e0202753. doi: 10.1371/journal.pone.0202753 (PMC6147413; doi:10.1371/journal.pone.0202753)
Supplement: S2 Methods — (DOCX) [file pone.0202753.s004.docx]

**S2 Methods**. Whole blood phenotyping

In brief, a staining cocktail containing the following antibodies was prepared and distributed to the clinical trial sites and stored at 4°C until use (antibodies from BD Biosciences, unless otherwise indicated): CD14–V450, CD19–V450, CD45–AmCyan, CD4–FITC, CD8–PerCP-Cy5.5, CD123–PE, HLA-DR–ECD (Beckman Coulter), CD86–PE-Cy5, CD56–PE-Cy7, CD11c–APC, CD3–Alexa700 and CD16–APC-Cy7. At the time of sample collection, 100 μL of whole blood collected in ACD-containing vacutubes was pipetted into a Trucount tube (BD Biosciences), followed by 100 μL of the staining cocktail. Samples were incubated for 15 minutes before the addition of 1X FACS Lysing solution (BD Biosciences). Samples were then stored and transported at approximately –80°C. The Trucount tubes were then analyzed on a LSRII (BD Biosciences) flow cytometer at a central analysis lab within 4 weeks of preparation. Data was analyzed using FlowJo (Treestar, Inc.). All populations were gated as singlets and CD45^+^. Specific cell populations were identified as follows: CD3 T cells were gated as low side scatter, CD14/CD19^–^, CD56^–^, CD16^–^, and CD3^+^. B cells were gated as low side scatter, CD19^+^, CD3^–^, CD56^–^ and CD16^–^. Natural killer (NK) cells were defined as low side scatter, CD14/CD19^–^, CD3-, CD56^+^ and/or CD16^+^. Granulocytes were identified as high side scatter and CD16^+^.
