# Supplementary figures and images for: Safety and tolerability of HIV-1 multiantigen pDNA vaccine given with IL-12 plasmid DNA via electroporation, boosted with a recombinant vesicular stomatitis virus HIV Gag vaccine in healthy volunteers in a randomized, controlled clinical trial
Source: PLoS One. 2018 Sep 20;13(9):e0202753. doi: 10.1371/journal.pone.0202753 (PMC6147413; doi:10.1371/journal.pone.0202753)

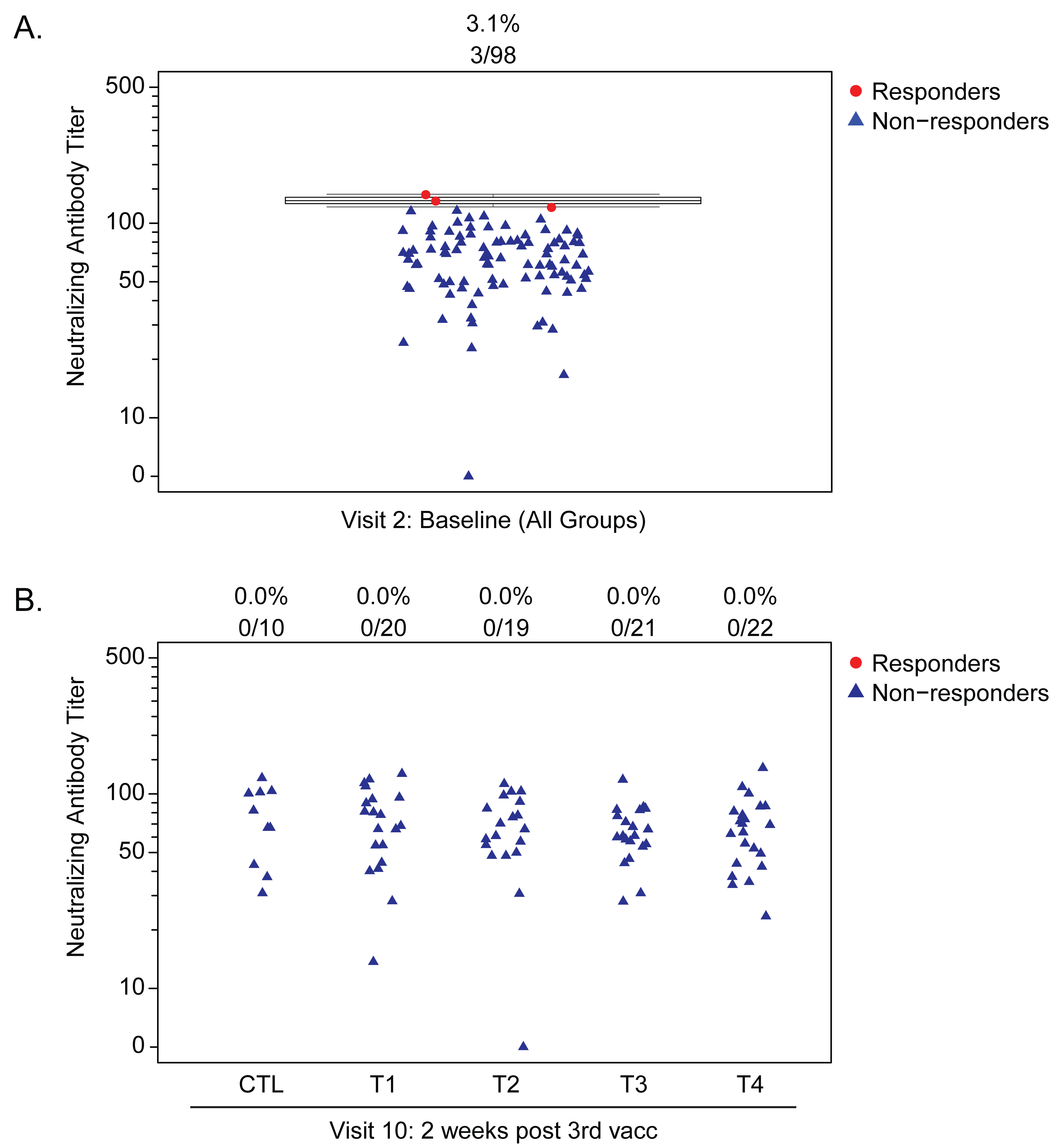

Supplement: S1 Fig — The percentage and frequency of responders is indicated above each plot. Red circles indicate positive responses (responders); blue triangles represent responses below the cutoff for positivity (non-responders). (TIF) [file pone.0202753.s005.tif]
