# Supplementary material for: Safety and tolerability of HIV-1 multiantigen pDNA vaccine given with IL-12 plasmid DNA via electroporation, boosted with a recombinant vesicular stomatitis virus HIV Gag vaccine in healthy volunteers in a randomized, controlled clinical trial
Source: PLoS One. 2018 Sep 20;13(9):e0202753. doi: 10.1371/journal.pone.0202753 (PMC6147413; doi:10.1371/journal.pone.0202753)
Supplement: S1 Table — Details of the decrease in lymphocyte and monocyte counts 1 day after VSV-Gag vaccination (p<0.001 for both groups), which rebounded by 3 days post-vaccination. Days indicated in the table are days following VSV vaccination. (DOCX) [file pone.0202753.s006.docx]

**S1 Table.**

| Subset | Days* | T1 | | | | | T3 | | | | |
| --- | --- | --- | --- | --- | --- | --- | --- | --- | --- | --- | --- |
|  |  | Fold Change | 95% CI | P-value | Overall P-value | Overall Q-value | Fold Change | 95% CI | P-value | Overall P-value | Overall Q-value |
| Lymphocytes | 1 | 0.429 | (0.33, 0.55) | < 0.001 | < 0.001 | < 0.001 | 0.326 | (0.27, 0.4) | < 0.001 | < 0.001 | < 0.001 |
|  | 3 | 0.971 | (0.76, 1.26) | 0.823 |  |  | 0.831 | (0.69, 1) | 0.062 |  |  |
|  | 7 | 1.017 | (0.79, 1.29) | 0.893 |  |  | 0.938 | (0.78, 1.15) | 0.526 |  |  |
|  | 14 | 0.924 | (0.72, 1.17) | 0.535 |  |  | 1.046 | (0.87, 1.26) | 0.649 |  |  |
| CD3+ T cells | 1 | 0.388 | (0.3, 0.49) | < 0.001 | < 0.001 | < 0.001 | 0.298 | (0.25, 0.36) | < 0.001 | < 0.001 | < 0.001 |
|  | 3 | 0.954 | (0.74, 1.2) | 0.703 |  |  | 0.851 | (0.69, 1.05) | 0.121 |  |  |
|  | 7 | 1.038 | (0.81, 1.32) | 0.759 |  |  | 0.958 | (0.78, 1.17) | 0.686 |  |  |
|  | 14 | 0.943 | (0.74, 1.2) | 0.63 |  |  | 1.043 | (0.85, 1.29) | 0.682 |  |  |
| CD4+ T cells | 1 | 0.403 | (0.32, 0.51) | < 0.001 | < 0.001 | < 0.001 | 0.299 | (0.25, 0.36) | < 0.001 | < 0.001 | < 0.001 |
|  | 3 | 0.964 | (0.76, 1.23) | 0.768 |  |  | 0.876 | (0.72, 1.07) | 0.194 |  |  |
|  | 7 | 1.037 | (0.81, 1.32) | 0.765 |  |  | 0.962 | (0.78, 1.17) | 0.71 |  |  |
|  | 14 | 0.947 | (0.74, 1.2) | 0.653 |  |  | 1.047 | (0.85, 1.29) | 0.654 |  |  |
| CD8+ T cells | 1 | 0.341 | (0.27, 0.44) | < 0.001 | < 0.001 | < 0.001 | 0.275 | (0.22, 0.34) | < 0.001 | < 0.001 | < 0.001 |
|  | 3 | 0.907 | (0.71, 1.17) | 0.447 |  |  | 0.793 | (0.65, 0.98) | 0.036 |  |  |
|  | 7 | 1.033 | (0.81, 1.32) | 0.793 |  |  | 0.947 | (0.76, 1.17) | 0.629 |  |  |
|  | 14 | 0.938 | (0.74, 1.2) | 0.608 |  |  | 1.052 | (0.85, 1.29) | 0.64 |  |  |
| B cells | 1 | 0.459 | (0.32, 0.68) | < 0.001 | < 0.001 | 0.005 | 0.349 | (0.28, 0.44) | < 0.001 | < 0.001 | < 0.001 |
|  | 3 | 0.687 | (0.47, 1) | 0.057 |  |  | 0.633 | (0.5, 0.79) | < 0.001 |  |  |
|  | 7 | 0.902 | (0.63, 1.29) | 0.581 |  |  | 0.83 | (0.65, 1.05) | 0.135 |  |  |
|  | 14 | 1.016 | (0.71, 1.48) | 0.931 |  |  | 1.019 | (0.81, 1.29) | 0.871 |  |  |
| NK total | 1 | 0.344 | (0.24, 0.5) | < 0.001 | < 0.001 | < 0.001 | 0.235 | (0.18, 0.3) | < 0.001 | < 0.001 | < 0.001 |
|  | 3 | 1.161 | (0.78, 1.74) | 0.461 |  |  | 0.926 | (0.71, 1.2) | 0.56 |  |  |
|  | 7 | 1.136 | (0.78, 1.66) | 0.503 |  |  | 1.011 | (0.78, 1.32) | 0.935 |  |  |
|  | 14 | 0.897 | (0.63, 1.29) | 0.551 |  |  | 1.049 | (0.81, 1.35) | 0.705 |  |  |
| Monocytes | 1 | 1.42 | (1.1, 1.86) | 0.013 | 0.05 | 0.19 | 1.475 | (1.17, 1.86) | 0.001 | 0.001 | 0.005 |
|  | 3 | 0.972 | (0.74, 1.26) | 0.834 |  |  | 0.967 | (0.78, 1.2) | 0.772 |  |  |
|  | 7 | 1.03 | (0.79, 1.32) | 0.82 |  |  | 0.909 | (0.72, 1.15) | 0.422 |  |  |
|  | 14 | 0.994 | (0.78, 1.29) | 0.96 |  |  | 0.998 | (0.79, 1.26) | 0.989 |  |  |
