# Supplementary material for: Safety and tolerability of HIV-1 multiantigen pDNA vaccine given with IL-12 plasmid DNA via electroporation, boosted with a recombinant vesicular stomatitis virus HIV Gag vaccine in healthy volunteers in a randomized, controlled clinical trial
Source: PLoS One. 2018 Sep 20;13(9):e0202753. doi: 10.1371/journal.pone.0202753 (PMC6147413; doi:10.1371/journal.pone.0202753)
Supplement: S1 Dataset — (ZIP) [file pone.0202753.s007.zip › Supporting Information. HVTN087_datasets/HVTN087_datasets.docx]

**De-identified data underlying the reported findings in HVTN 087:**

Subject_Master.csv : masked participant details, including demographics, treatment group, reasons for discontinuation and study termination.

Vaccination_detail.csv: Received treatment details by study product administration visit.

Reactogenicity_list.csv: all local and systemic reactogenicity events

Reactogenicity_summary.csv: reactogenicity events summarized at the maximum reportable severity grade by participant and visit number

Adverse_Events.csv: all non reactogenicity adverse events

Safety_lab.csv: safety laboratory results and corresponding grades are listed by participant and visit collected.

Social_Impact.csv: social impacts reported

Acceptability.csv: acceptability responses by participant and visit

Pain_Score.csv: pain scores by participant, visit and injection number

Lab_chemistry.csv: cell counts and chemistry values for various laboratory measures

Trucount.csv: cell subset concentrations and percentages at innate immune time points from the Trucount assay
